# Supramolecular Mask-Mediated Regioselective bis-Bingel-Functionalization of M_3_N@I_*h*_‑C_80_ (M = Lu, Sc)

**DOI:** 10.1021/jacs.6c16243

**Published:** 2026-10-16

**Authors:** Valentina Iannace, Clara Sabrià, Sheng Zhu, Olga A. Stasyuk, Miquel Solà, Teodor Parella, Sergio Fernández, Felipe Gándara, Inhar Imaz, Daniel Maspoch, Jianyuan Zhang, Ferran Feixas, Xavi Ribas

**Affiliations:** † Institut de Química Computacional i Catàlisi (IQCC) and Departament de Química, Universitat de Girona, Campus Montilivi, 17003 Girona, Catalonia, Spain; ‡ Department of Chemistry and Chemical Biology, Rutgers, 242612 The State University of New Jersey, Piscataway, New Jersey 08854, United States; § Servei de RMN, Facultat de Ciències, Universitat Autònoma de Barcelona, Campus UAB, 08193 Bellaterra, Catalonia, Spain; ∥ Materials Science Institute of Madrid − CSIC, 28049 Madrid, Spain; ⊥ Catalan Institute of Nanoscience and Nanotechnology (ICN2), CSIC and The Barcelona Institute of Science and Technology, Campus UAB, 08193 Bellaterra, Catalonia, Spain; # ICREA, Pg. Lluís Companys 23, 08010 Barcelona, Catalonia, Spain; ¶ Instituto de Nanociencia y Materiales de Aragon (INMA), CSIC-Universidad de Zaragoza, C/ Pedro Cerbuna 12, 50009 Zaragoza, Spain

## Abstract

Exohedral functionalization of endohedral metallofullerenes (EMFs) is essential for exploiting these intriguing molecules as novel materials in applications such as biomedicine and organic solar cell device design. In the case of trimetallic nitride template EMFs (TNT-EMFs), many functionalization methods effective for empty fullerenes are inaccessible or less efficient due to the presence of the encapsulated cluster. This inner cluster increases the electron density on the carbon cage surface, reducing its reactivity. Moreover, it has been demonstrated that the inner cluster controls both the reactivity and regioselectivity of bare TNT-EMFs. The supramolecular mask strategy (SMS) has yielded promising results in the functionalization of C_60_ and C_70_ and, for the first time, is applied here to endohedral fullerenes to achieve mono- and bis-functionalization of Lu_3_N@I_*h*_-C_80_ and Sc_3_N@I_*h*_-C_80_. Encapsulation of both TNT-EMFs within a tetragonal prismatic cage host is crucial for enhancing their reactivity, enabling bis-functionalization under milder conditions and with shorter reaction times. Comprehensive spectroscopic and spectrometric characterization, combined with molecular dynamics (MD) simulations and density functional theory (DFT) calculations, identified the most favorable and stable bis-adducts by considering the positive alignment between the geometry imposed by the inner cluster and that allowed by the tetragonal prismatic nanocapsule mask. This resulted in preferential formation of five bis-adducts (out of 27 possible), two of them bearing a 120° angle between the addends and aligning with the inner TNT cluster trigonal geometry.

## Graphical abstract

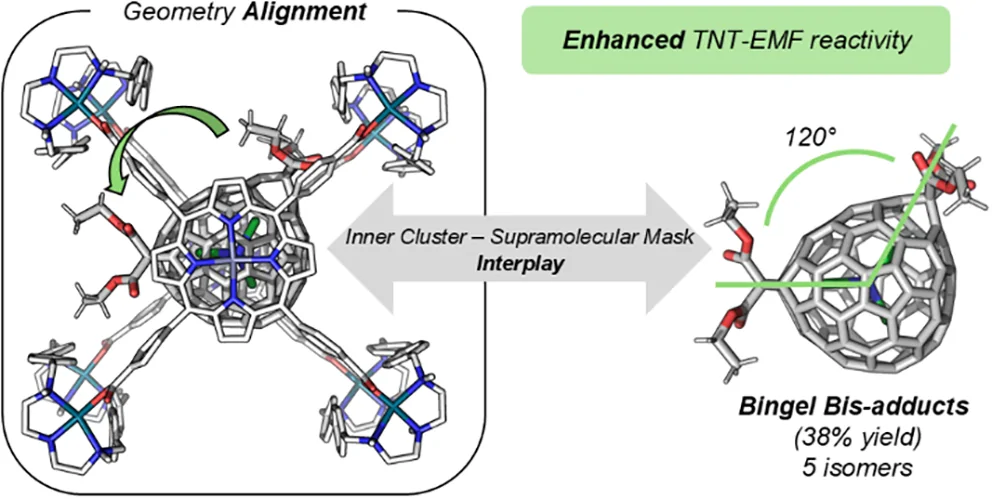

## Introduction

Trimetallic nitride template endohedral metallofullerenes (TNT-EMFs)^1–4^ have received attention in the last decades as novel materials with potential applications in medicine,^5,6^ organic solar cell devices,^7^ and nanomaterials.^8^ Interestingly, the intrinsic properties (magnetic, spectroscopic, and nuclear) of the inner metals are the origin of their suitability for applications. Regarding the carbon cage, as with empty fullerenes, exohedral functionalization^9,10^ is crucial to broaden the scope of potential applications of these interesting molecules, thereby conferring better solubility and chemical stability to the resulting materials.^11^ With its current gram-scale availability,^12^ Sc_3_N@I_*h*_-C_80_ is the most abundant fullerene obtained as a single isomer after C_60_ and C_70_, and, hence, the reported attempts of functionalizing TNT-EMFs have increased.^8^ Within the TNT-EMF family, Lu_3_N@I_*h*_-C_80_ has the second-highest production yield and has already shown a remarkable potential as an X-ray contrast agent, when Gd is mixed in the inner cluster,^13^ and as a component for photovoltaic cells with its PCBM and PCBH derivatives.^7^

One of the most extensively used and studied functionalization reactions for C_60_ and C_70_ fullerenes is the Bingel [2 + 1] cyclopropanation reaction.^14^ In the case of EMF compounds, the presence of the inner cluster increases the complexity of the process due to their electron-rich surface and charge distribution, making them generally less reactive than empty fullerenes, and TNT-EMFs^1^ are even less reactive due to their closed-shell electronic structure. Many functionalization methods that work for classical EMFs and empty fullerenes do not work on TNT-EMFs. In the context of the Bingel reaction, the classical reaction conditions were ineffective for Sc_3_N@I_*h*_-C_80_ ^15^ and Lu_3_N@I_*h*_-C_80_.^16^ Echegoyen and co-workers overcame this problem by adding DMF as a solvent,^17^ obtaining 40% yield of mono-Bingel Lu_3_N@I_*h*_-C_80_C­(CO_2_Et)_2_ (Figure 1a) and a similar yield for mono-Bingel Sc_3_N@I_*h*_-C_80_C­(CO_2_Et)_2_ in shorter reaction times at 60 °C._._ DMF favors this reaction because the stability of the transition state featuring the three-membered ring of the Bingel reaction mechanism is enhanced by the polarity of the solvent.^18^ Another successful strategy to obtain cyclopropanated adducts of M_3_N@I_*h*_-C_80_ (M = Lu, Sc) was reported by Dorn and co-workers via radical-based addition reaction using Mn­(OAc)_3_·2H_2_O in chlorobenzene under reflux^19^ (Figure 1b).

**Figure 1:**
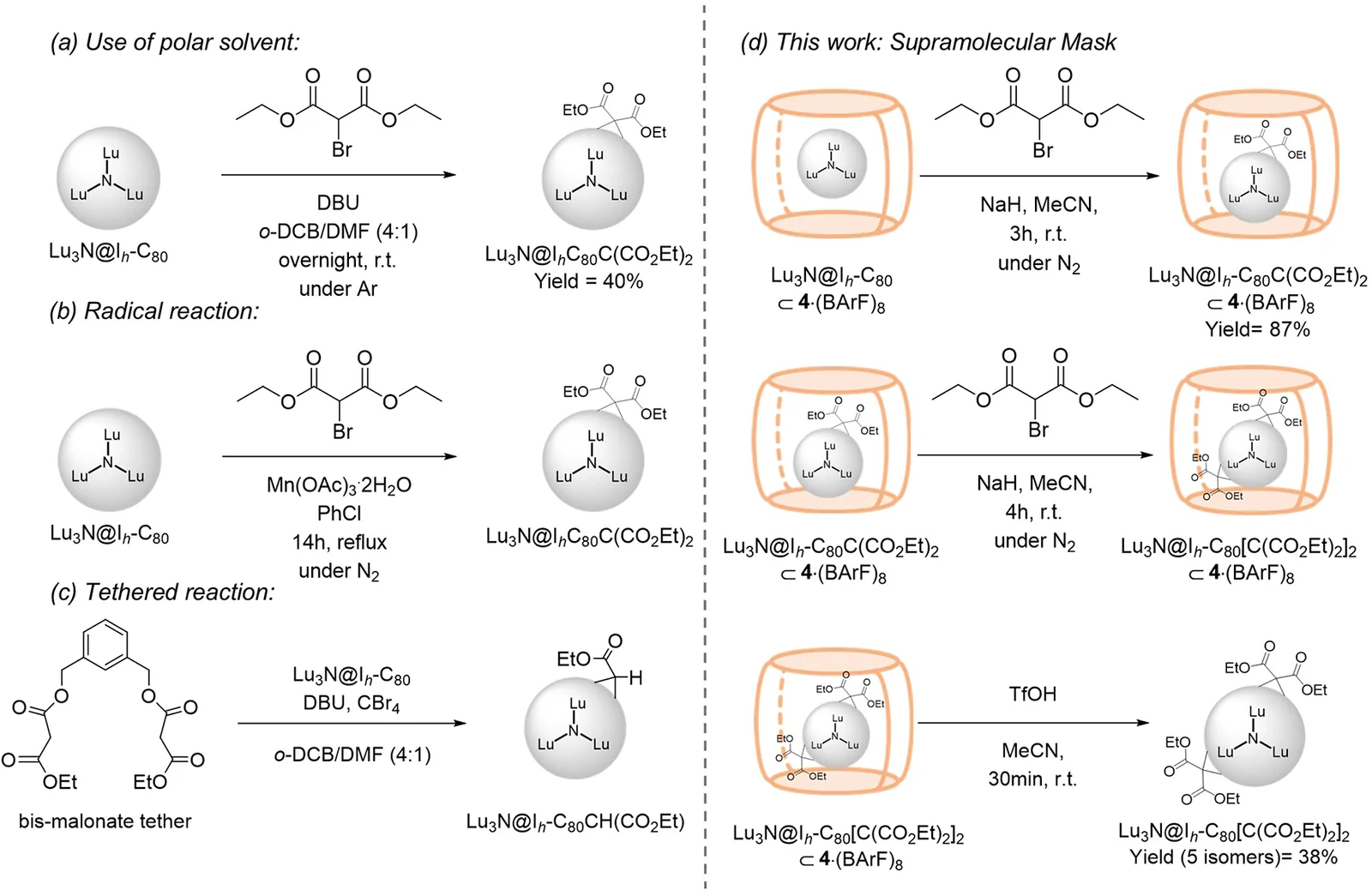
Previous strategies to obtain cyclopropanated mono-adducts of Lu_3_N@I_*h*_-C_80_. (a) Echegoyen’s strategy using DMF and DBU to obtain mono-Bingel adducts.^17^ (b) Mono-adduct obtained via radical-based reaction using Mn­(OAc)_3_, reported by Dorn and Gibson (yield not reported).^19^ (c) Unsuccessful attempt to obtain a tether-directed regio-controlled bis-functionalization^20^ by Echegoyen (yield not reported). (d) (This work) The use of a Supramolecular Mask to enhance the reactivity of encapsulated Lu_3_N@I_*h*_-C_80_ to obtain mono- and bis-adducts (upon guest release).

If the monofunctionalization upon Bingel reaction of TNT-EMFs is challenging, the bis- and polyfunctionalization is even more arduous;^21^ moreover, as in empty fullerenes, regioselectivity issues arise in polyfunctionalization^22^ due to the several possible regioisomers from the high number of different bonds available for the second and subsequent functionalization. Echegoyen and Yamakoshi groups separately reported 1,3-dipolar “Prato” adducts on different TNT-EMFs,^23–26^ which is the most successful polyaddition reported on TNT-EMFs. The sole regioselective Bingel bis-addition was reported by Dorn et al., on a more reactive Sc_3_N@D_3*h*_-C_78_,^27^ and there was no success in the M_3_N@I_*h*_-C_80_ series, even with more exotic approaches, such as the tether-directed^28^ remote functionalization for regioselectivity on the bis-functionalization, in analogy to bare fullerenes^29^ (Figure 1c). Using a dimalonate with a tether spacer between the two malonate moieties,^20^ Echegoyen and co-workers attempted the synthesis of a single bis-adduct of Lu_3_N@I_*h*_-C_80_. Nevertheless, the reaction was unsuccessful, affording only the partially cleaved mono-adduct as product, presumably because of the strong directing effect exerted by the inner cluster, not compatible with the directing effect of the covalent tether-linker (Figure 1c). Notably, the crystallization of EMF multiadducts has been a daunting task, as in the above-mentioned examples, only one crystal structure of a Gd_3_N@I_*h*_-C_80_ Prato bis-adduct was reported,^25^ while other reports use alternative approaches (*e.g*., NMR, theoretical calculation) for structural elucidation.

Our group showed the potential of the supramolecular mask strategy to obtain regiocontrol on the polyfunctionalization of tightly encapsulated fullerenes C_60_ ^30,31^ and C_70_.^32^ Thanks to the four windows of our tetragonal prismatic nanocapsule masks, and in some cases to the use of an additional 10­[CPP] ring to obtain three-shell supramolecular complexes,^33,34^ the functionalization occurs only in the regions of the carbon cage left exposed by the mask. Moreover, regioselective functionalization over encapsulated fullerenes in supramolecular masks can be performed under milder conditions and in polar solvents, where, commonly, bare fullerenes or EMFs are insoluble. However, whether this strategy could be successfully extended to EMFs, whose reactivity and regioselectivity are strongly influenced by the encapsulated inner cluster, remains unexplored.

In this work (Figure 1d), we investigate whether supramolecular confinement can be used to enhance the reactivity of a challenging TNT-EMF substrate, but also to control the geometry of its bis-functionalization, despite the strong influence of the encapsulated inner cluster. We report the mono- and bis-functionalization of Lu_3_N@I_*h*_-C_80_ upon Bingel reaction using a supramolecular mask. The reaction conditions are milder, the reaction times are shorter with respect to the previously reported attempts, and the yields (up to 87%) of the resulting host–guest complex of the mono-adduct are much higher. In addition, starting from the encapsulated mono-adduct, we were able to obtain bis-adducts upon Bingel reaction and guest release in good yield (total yield of regioisomer mixture 38%). The nanocapsule favors the second addition through the windows of the nanocapsule and in correspondence with the metals of the inner cluster, providing control over the geometry of the resulting bis-adducts.

Crystal structures of Lu_3_N@I_*h*_-C_80_⊂**4**·(BArF)_8_ and Lu_3_N@I_*h*_-C_80_C­(CO_2_Et)_2_⊂**4**·(BArF)_8_ were obtained giving information about the geometry match between host and guest, DFT calculations helped in identifying the most stable bis-adducts with a geometry matching with the tetragonal nanocapsule and full-characterization of the mono- and bis-adducts showed the obtention of open [6,6] adducts and helped in understanding the number of bis-adducts obtained. Finally, molecular dynamics (MD) studies aligned with X-ray diffraction (XRD) and reinforced the assignment of the most favorable bis-regioisomers.

## Results and Discussion

Given the reported high affinity of the tetragonal prismatic Pd-based supramolecular nanocapsule **4**·(BArF)_8_ ^35^ for C_60_ and C_70_ fullerenes,^36^ C_84_,^37^ fullertubes,^38^ and U-containing C_80_-based EMFs,^39^ Lu_3_N@I_*h*_-C_80_ encapsulation in **4**·(BArF)_8_ was attempted. The dimensions and shape of its cavity (Figure 2a), with a Zn^...^Zn distance of 14 Å between the two Zn-TCPP caps, and the cage adaptability to different sizes and shapes of the fullerenic guests thanks to its breathability, render it a priori a good host for Lu_3_N@I_*h*_-C_80_.^11,40^ The encapsulation was performed in a mixture of MeCN and *o*-xylene (MeCN 1:4 *o*-xylene, 3 h, r.t.) to ensure the solubility of both host and guest (Figure 2b). The formation of the host–guest complex was confirmed by HR-MS (ESI-QTOF) (Figure 2c), and the final encapsulated product, Lu_3_N@I_*h*_-C_80_⊂**4**·(BArF)_8_, was obtained by crystallization (98% yield) and characterized by NMR (Figures S8–S15).

**Figure 2:**
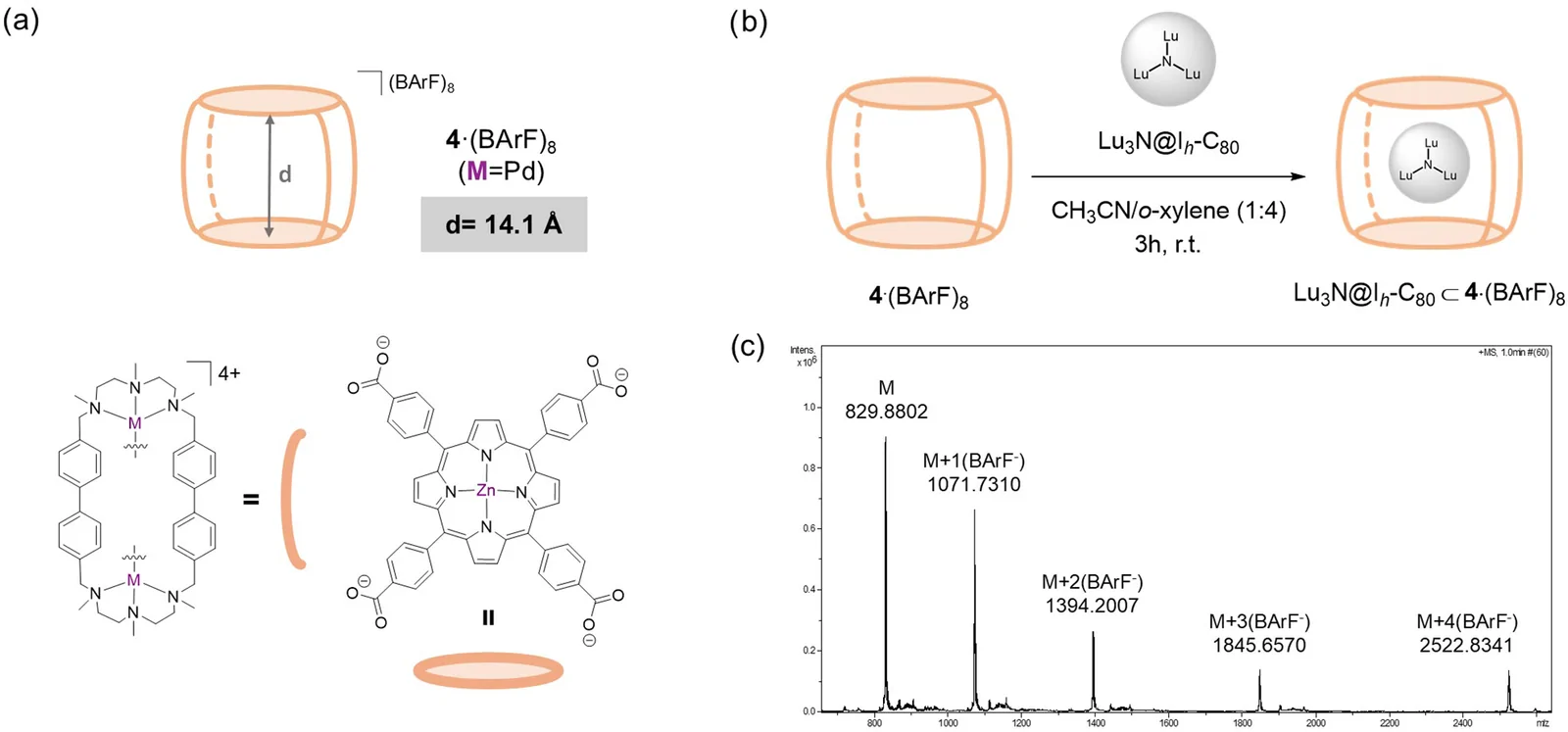
(a) Composition of Pd-based nanocapsule **4**·(BArF)_8_ supramolecular host. (b) Encapsulation of Lu_3_N@I_*h*_-C_80_ in **4**·(BArF)_8_, reaction conditions and (c) HR-MS spectrum of the host–guest complex Lu_3_N@I_*h*_-C_80_⊂**4**·(BArF)_8_, charges from (+8) to (+4). Sample dissolved in MeCN.

Upon slow diethyl ether diffusion in DCM, crystals of the host–guest adduct were obtained, and XRD data for Lu_3_N@I_*h*_-C_80_⊂**4**·(BArF)_8_ were collected using synchrotron radiation. Analysis of the diffraction data reveals that Lu_3_N@I_*h*_-C_80_⊂**4**·(BArF)_8_ crystallizes in the tetragonal P4/*mnc* space group. Owing to the tetragonal symmetry of the crystal, Lu_3_N@I_*h*_-C_80_ is found in four symmetry-related orientations inside the nanocapsule (Figure 3c), each of them featuring a 90° rotation around the crystallographic *c* axis. The N atom is located at the center of the I_*h*_-C_80_ cage, with one of the lutetium atoms lying in the same equatorial plane, parallel to the porphyrin planes. The I_*h*_-C_80_ cage is placed exactly in the center of the nanocapsule (Figure 3b) at a distance of 3.05(2) Å from the porphyrins (Figure 3a), in agreement with the previously reported host–guest complexes between porphyrins and TNT-EMFs.^41^

**Figure 3:**
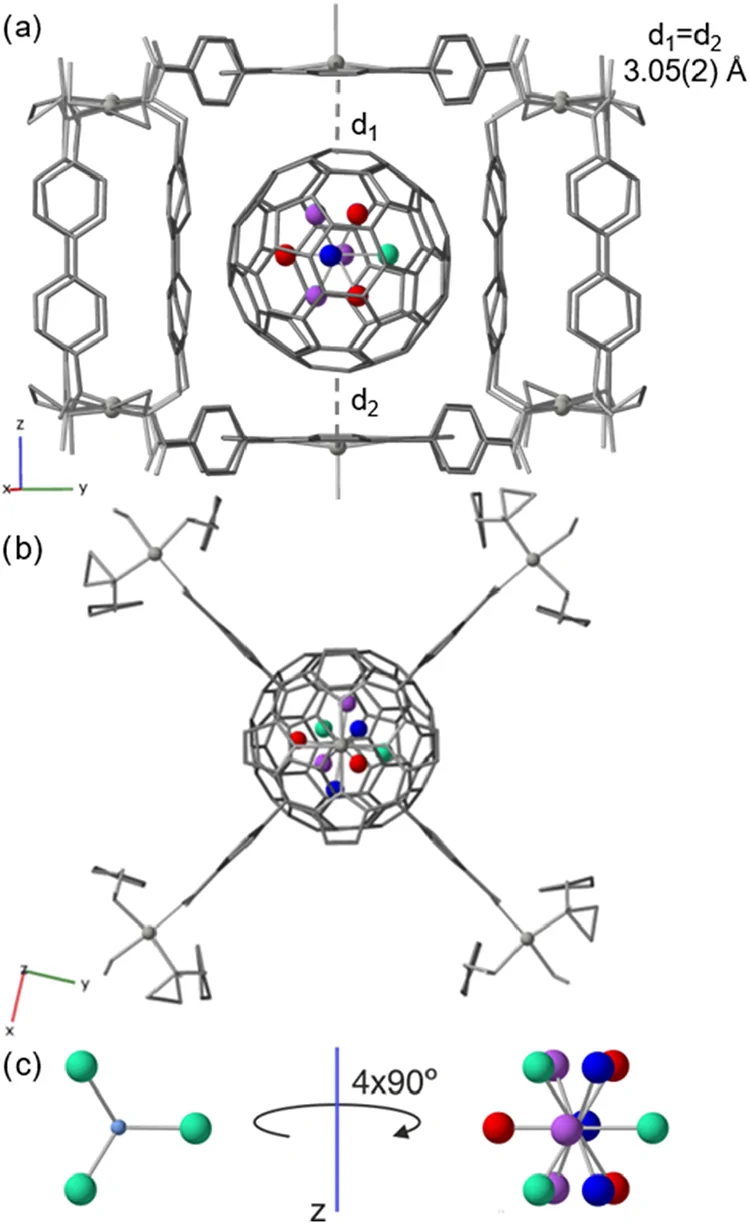
Crystal structure of Lu_3_N@I_*h*_-C_80_⊂**4**·(BArF)_8_. (a) Front view of the host–guest complex depicting the zinc porphyrin-endohedral distance (3.05(2) Å). (b) Top view of the host–guest complex. (c) Four possible orientations of the Lu_3_N inner cluster upon 90° rotation around the *c* axis (blue line), with different colors corresponding to consecutive 90° rotations.

With the Lu_3_N@I_*h*_-C_80_⊂**4**·(BArF)_8_ complex fully characterized, we attempted the Bingel functionalization of the encapsulated Lu_3_N@I_*h*_-C_80_, using diethyl bromomalonate, MeCN as the solvent, and NaH as the base (Figure 4a). The reaction was performed at room temperature, and it was monitored by HR-MS (Figures S25 and S26). Since the solubility of Lu_3_N@I_*h*_-C_80_⊂**4**·(BArF)_8_ is dictated by the cage, the use of a polar solvent such as MeCN is allowed, which should favor the reaction^18^ at milder conditions.

**Figure 4:**
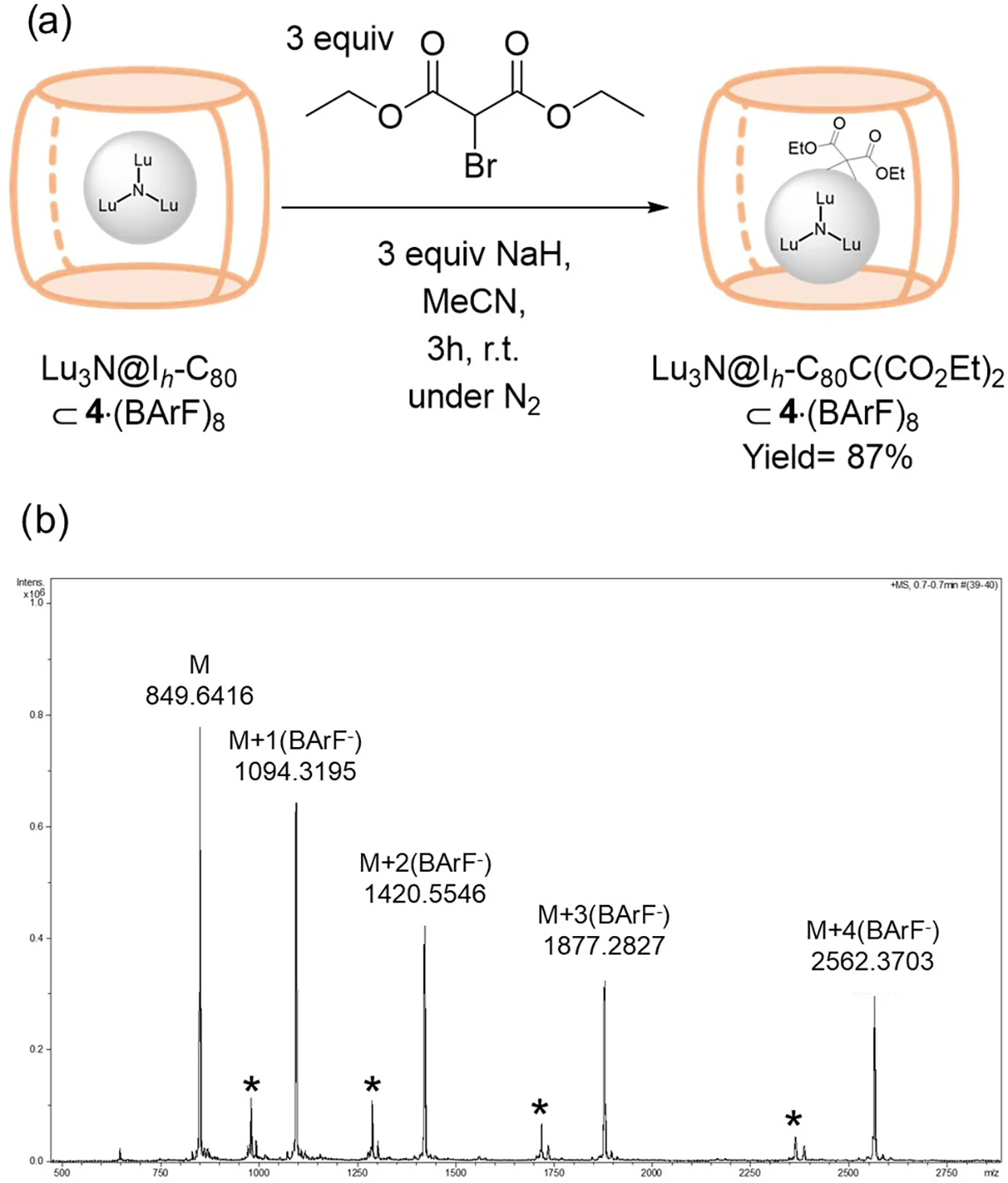
(a) Monofunctionalization reaction conditions. (b) HR-MS spectrum of the host–guest complex obtained, Lu_3_N@I_*h*_-C_80_C­(CO_2_Et)_2_⊂**4**·(BArF)_8_, charges from (+8) to (+4). Peaks labeled with * correspond to the presence of Cl^–^ or Br^–^ as counterion. Sample dissolved in MeCN.

Upon addition of 3 equiv. of diethyl bromomalonate and NaH base, the Lu_3_N@I_*h*_-C_80_C­(CO_2_Et)_2_⊂**4**·(BArF)_8_ is obtained quantitatively in 3 h (Figure 4b). The resulting host–guest complex of the mono-adduct was then obtained upon filtration and crystallization (DCM/diethyl ether) in 87% yield.

The Bingel monofunctionalization process on TNT-EMFs^17,19^ is significantly faster when encapsulated within the supramolecular mask, highlighting the importance of the nanocapsule to stabilize the Lu_3_N@I_*h*_-C_80_ and enhance its reactivity in MeCN. The Lu_3_N@I_*h*_-C_80_C­(CO_2_Et)_2_⊂**4**·(BArF)_8_ host–guest complex was characterized by NMR (Figures S28–S35) techniques, HR-MS (Figures 4b and S27), and X-ray diffraction (Figures 5a–c).

**Figure 5:**
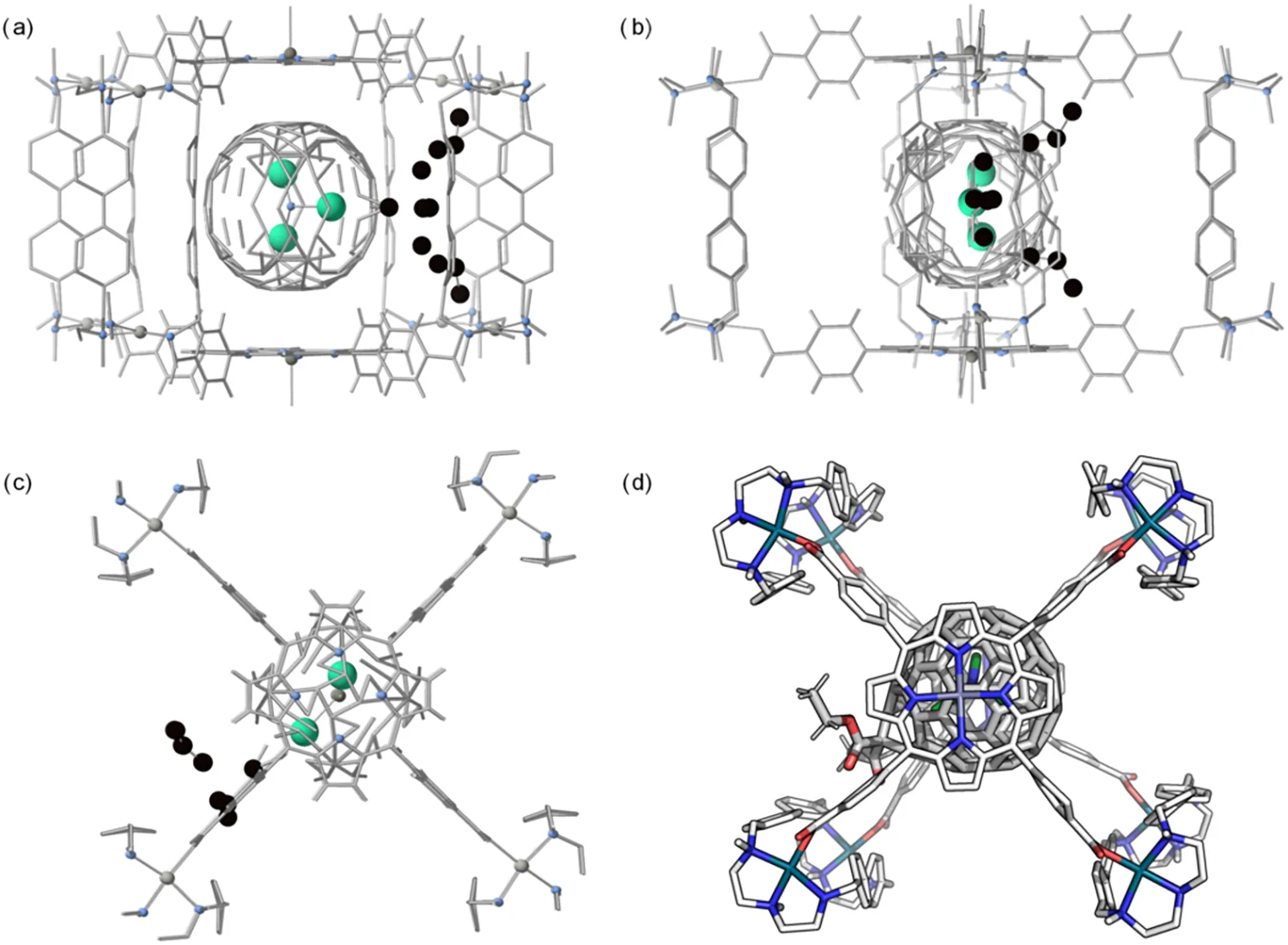
Crystal structure of Lu_3_N@I_*h*_-C_80_C­(CO_2_Et)_2_⊂**4**·(BArF)_8_. (a) Front, (b) side, and (c) apical view of the host–guest complex. Simplification of the crystal structure to show the placement of the functionalized TNT-EMF inside the nanocapsule. (d) Conformation of the mono-adduct inside the nanocapsule obtained by MD simulations showing the same orientation of the functionalization moiety.

The crystal structure of Lu_3_N@I_*h*_-C_80_C­(CO_2_Et)_2_⊂**4**·(BArF)_8_ presents clear similarities with that of Lu_3_N@I_*h*_-C_80_⊂**4**·(BArF)_8_. In both cases, the TNT-EMF is located at the center of the cavity (Figure 5a,b), and the inner cluster is disordered over four symmetry-related orientations obtained by a 90° rotation. Consequently, the functionalization is observed in four equivalent orientations. In all of them, one lutetium atom is oriented toward the site of functionalization, in agreement with reported studies.^19^ Despite the limited resolution and overall XRD data quality, together with the symmetry-imposed disorder affecting the C_80_ cage (which prevents an accurate location of all carbon atoms), the orientation of the functional group can still be reliably extracted. In particular, the distance between the two carbon atoms of the [6,6] bond involved in the functionalization is ∼2.2 Å, in agreement with an open fulleroid structure.^42,43^ Strikingly, the functionalization is pointing toward the aromatic panels of one of the clips to find further stabilization through noncovalent interactions (Figure 5c). The latter interactions were previously characterized for a fully equatorial tetrakis-Bingel-C_60_ adduct encapsulated in **4**·(BArF)_8_,^30^ and the bigger size of the carbon cage (C_80_ compared to C_60_) is deemed crucial to enhance the intensity of these interactions

MD simulations have been carried out for the mono-adduct Lu_3_N@I_*h*_-C_80_C­(CO_2_Et)_2_⊂**4**·Cl_8_, showing the same approximation of the diethyl malonate arms toward the aromatic panels of one clip, confirming the structural data obtained from crystallography (Figure 5d). During the simulation, the monoaddend explores one window while one lutetium atom of the cluster is pointing toward the substituent (Video S1). The expected C_2_ rotation along the Lu–N axis exposes the other two Lu atoms at 120° at the center of the contiguous window of the nanocapsule (despite the MD method used precluding this rotation), and so accessible for a second addition.

To release the mono-adduct from **4**·(BArF)_8_, we first attempted replacement with nonfunctionalized Lu_3_N@I_*h*_-C_80_, relying on the higher affinity of the bare Lu_3_N@I_*h*_-C_80_ for the nanocapsule and the higher solubility of the mono-adduct in toluene. We solubilized the Lu_3_N@I_*h*_-C_80_C­(CO_2_Et)_2_⊂**4**·(BArF)_8_, complex in a mixture of CH_3_CN/toluene (1:1), and an excess (4–20 equiv.) of Lu_3_N@I_*h*_-C_80_ was added at 40 °C. We monitored the exchange by HR-MS (Figure S36), observing the slow replacement of the mono-adduct by Lu_3_N@I_*h*_-C_80_ over time. We obtained almost complete exchange after 7 days. In industrial scale-up, this process can be optimized for continuous functionalization. However, at our experimental scale, considering the large excess of Lu_3_N@I_*h*_-C_80_ needed and the long time required to obtain complete exchange, we decided to release the mono-adduct by disassembling the nanocapsule with acid treatment. Upon addition of triflic acid, **4**·(BArF)_8_ nanocapsule can be fully disassembled straightfowardly,^31^ so mono-adduct Lu_3_N@I_*h*_-C_80_C­(CO_2_Et)_2_ was easily separated (Figure S37). The mono-adduct was characterized by NMR, MALDI-MS, HPLC, and UV–vis (Figures S39–S42), and all the data agreed with the previously reported [6,6] open Lu_3_N@I_*h*_-C_80_C­(CO_2_Et)_2_ reported by Echegoyen.^17^

Our main focus of interest was the regio-controlled synthesis of bis-adducts, since no Bingel bis-addition on M_3_N@I_*h*_-C_80_ has been reported. Starting from the Lu_3_N@I_*h*_-C_80_⊂**4**·(BArF)_8_ complex, we pushed the reaction conditions by increasing the equivalents of diethyl bromomalonate and NaH base, and by increasing the reaction time. However, the excess of base and the long reaction time prompted the decomposition of the supramolecular cage.

For this reason, we switched the strategy and performed the functionalization reaction directly on the encapsulated mono-adduct, Lu_3_N@I_*h*_-C_80_C­(CO_2_Et)_2_⊂**4**·(BArF)_8_ (Figure 6a). Upon addition of diethyl bromomalonate and NaH (6 equiv.) for 4 h (Figure 6a), bis-adducts accumulated as the major product. Notably, it is important to state that the reactivity of more polyfunctionalized TNT-EMF is enhanced; thus, the obtention of tris-adducts is observed by HR-MS (Figure 6b). In comparison, a control experiment was performed, subjecting the mono-adduct Lu_3_N@I_*h*_-C_80_C­(CO_2_Et)_2_ to NaH and an excess of bromomalonate, but no bis-adducts were observed (Figure S91).

**Figure 6:**
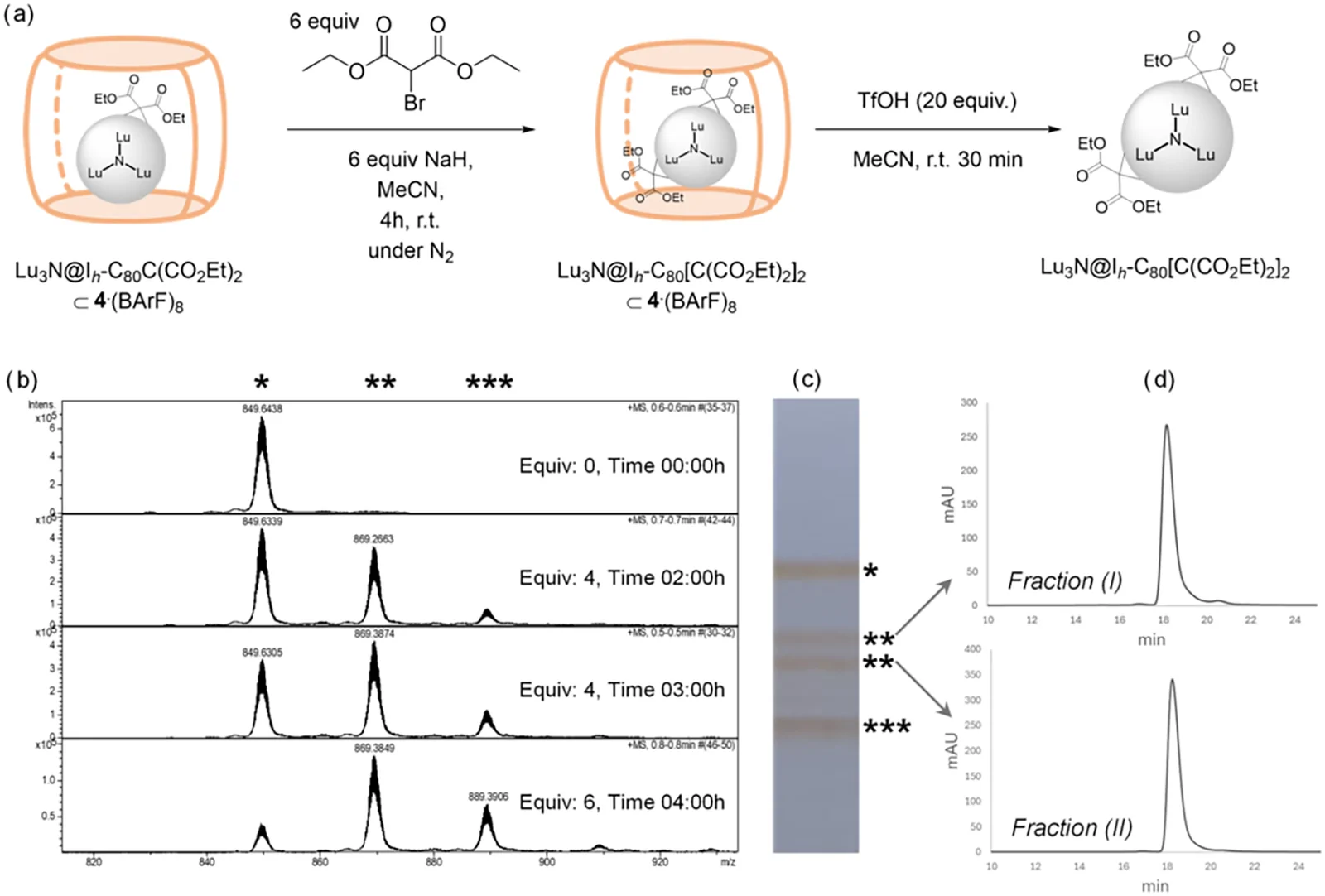
(a) Procedure for the Bingel bis-functionalization and guest release. (b) Monitoring by HR-MS. * Mono adduct, ** bis-adducts, *** tris-adducts charge (+8) peaks. (c) Preparative TLC to separate the adducts extracted from the nanocapsule (toluene 100%). (d) HPLC chromatogram of the bis-adducts two fractions obtained from the preparative TLC (PYE column, *o*-xylene, 0.2 mL/min).

The bis-adducts obtained by our supramolecular mask protocol were accumulated and released upon disassembly of the nanocapsule with triflic acid (Figure 6a). Preparative TLC was used to separate four fractions, one corresponding to unreacted mono-adduct, two fractions (I and II) of bis-adducts, and another fraction corresponding to the tris-adducts (Figure 6c). Bis-adduct Fraction I (yield 18%) and Fraction (II) (yield 20%) afforded a total yield of 38% in remarkably short reaction times (4 h). Two factors may be responsible for the enhanced reactivity of Lu_3_N@I_*h*_-C_80_ inside the nanocapsule: the main one is the possibility to use a polar solvent (MeCN),^17^ which is particularly advantageous for the Bingel reaction due to its ability to stabilize charged intermediates and transition states. Additionally, the donation of electron density from the guest to the porphyrins of the nanocapsule might promote the stabilization of the guest upon encapsulation. To validate this latter hypothesis, cyclic voltammetry studies were performed (Figure S87) on different host–guest complexes. Comparison of empty **4**·(BArF)_8_, C_60_⊂**4**·(BArF)_8_, C_70_⊂**4**·(BArF)_8_, and Lu_3_N@I_*h*_-C_80_⊂**4**·(BArF)_8_ showed that the latter exhibits a slight degree of electron donation to the porphyrins, although the small differences point toward the use of a polar solvent as the main factor for the enhanced reactivity. In addition, the supramolecular mask strategy is geometrically compatible with the strong inner cluster directing effect thanks to the orientation adopted by the mono-adduct in close proximity to one of the clips (see Figure 5), thereby favoring the second addition through the windows of the nanocapsule.

MALDI-MS of fractions I and II confirmed their bis-adduct nature (Figure S60). Then, each fraction was injected into the HPLC using the Nacalai PYE column, and only one peak appeared in both cases (Figure 6d). However, ^1^H NMR of each fraction indicated the presence of a mixture of isomers (Figures S62 and S64), which could not be separated by preparative TLC or HPLC. The ^1^H NMR spectrum for fraction I showed multiplets at 4.63 and 1.49 ppm corresponding to the ethyl moiety, whereas for fraction II, the multiplet signals appeared at 4.58 and 1.46 ppm. COSY NMR spectra did not afford any clue in differentiating the different bis-regioisomers contained in each fraction (Figures S63 and S65).

To have a better estimation of the number of bis-adducts obtained (fractions I and II), we used ^13^C-labeled diethyl malonate (Figure 7). To use this malonate, we must rely on the Bingel-Hirsh reaction with the formation of the diethyl halomalonate *in situ*. We first performed a Bingel-Hirsh reaction with nonlabeled diethyl malonate, I_2_, and NaH, confirming that the same product as in the Bingel reaction was obtained (Figures S79–S81). Then, we synthesized a ^13^C-labeled mono-adduct upon Bingel-Hirsh reaction and characterized it by ^1^H NMR and ^13^C NMR (Figures S84–S86). A similar pattern to the nonlabeled mono-adduct in the ^1^H NMR was observed. On the other hand, the ^13^C NMR spectrum showed a large doublet at 165.08 ppm for the C=O and a large triplet centered at 60.30 ppm (with ^1^J­(CC)=62 Hz) characteristic of an open fulleroid structure^19,42^ (Figure 7a). The latter, together with the XRD data, confirmed the nature of the mono-adduct, *i.e*., the [6,6]-open Lu_3_N@I_*h*_-C_80_C­(CO_2_Et)_2_.

**Figure 7:**
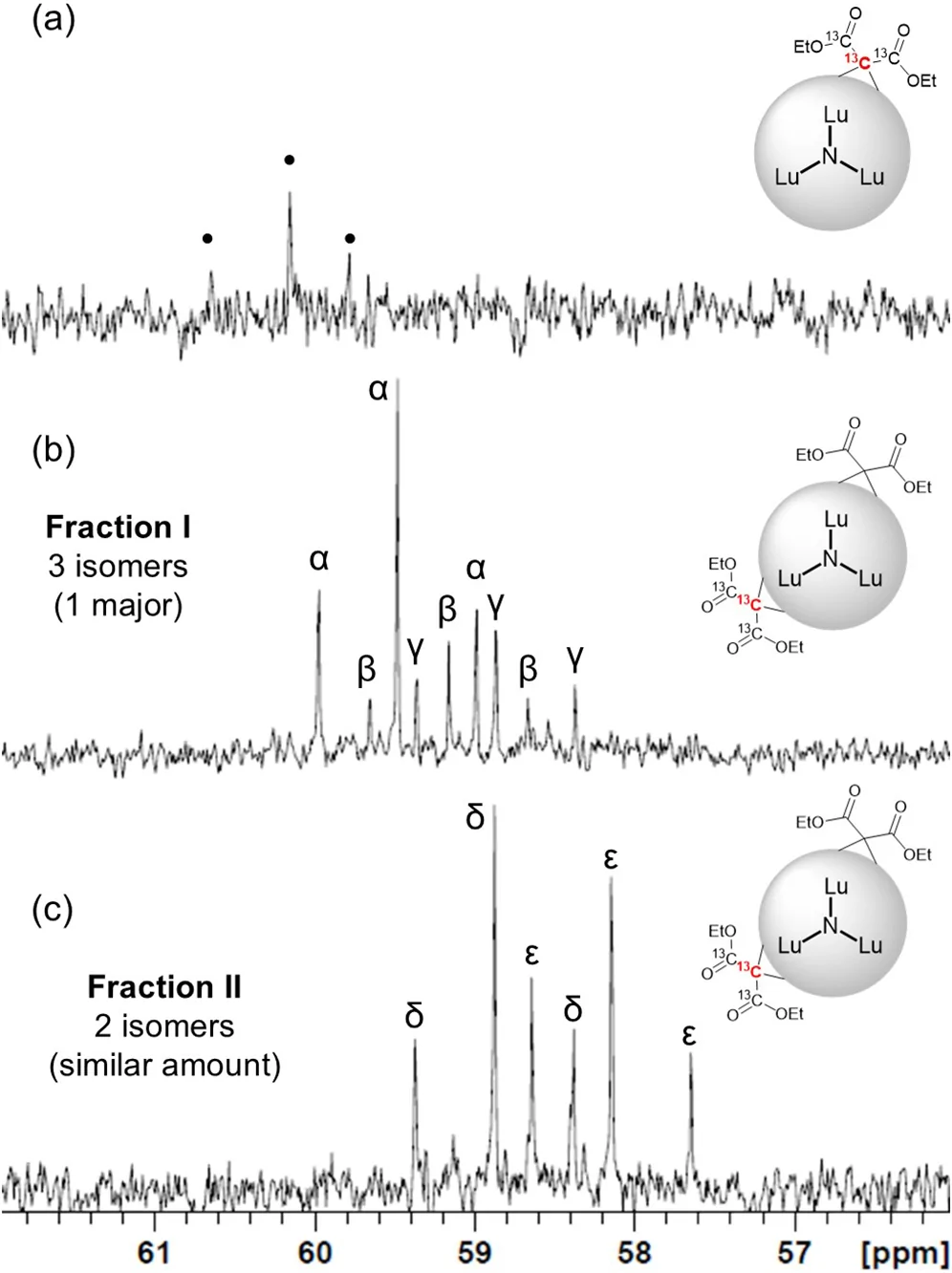
Expanded methano carbon region corresponding to the ^13^C NMR spectra of (a) ^13^C-labeled Lu_3_N@I_*h*_-C_80_C­(CO_2_Et)_2_; (b) fraction I of the single-labeled bis-adducts; and (c) fraction II of the bis-adducts.

Then, starting from the nonlabeled Lu_3_N@I_*h*_-C_80_C­(CO_2_Et)_2_⊂**4**·(BArF)_8_, we synthesized the bis-adducts with the second addend labeled. We envisioned that having one nonlabeled functionalization, we could simplify the pattern observed in the ^13^C NMR spectrum. Also, out of the 27 possible [6,6]-[6,6] bis-adducts of a TNT-EMF with an I_*h*_-C_80_ carbon cage, 18 have a *C*_1_ symmetry,^23^ giving rise to even more complex asymmetric NMR multiplet patterns.

We focused on the –C–^13^C–C– methanobridge signal, which is very characteristic and has a chemical shift around 60 ppm, confirming the obtention of [6,6]-[6,6] open bis-adducts. In the ^13^C NMR of fraction I, we distinguished three different triplets (Figure 7b), one major and two minor (in equal amounts), indicating the presence of 3 isomers. In fraction II, two different isomers in equimolar amounts were observed (Figure 7c). Remarkably, only 5 isomers (3 in major amounts) are obtained out of the 27 possible [6,6]-[6,6] bis-adduct regioisomers thanks to the positive alignment between the directing effect of the inner cluster and the geometry imposed by the supramolecular mask. Yet, a tentative structural assignment based only on the NMR data is not feasible; hence, we resorted to DFT calculations and molecular dynamics (MD) simulations.

DFT calculations indicate that monofunctionalization is slightly more energetically favorable at the [6,6] cyclopenta­[cd]­phenalene-like ring junction than at the [5,6] corannulene-like ring junction. Only open fulleroid structures were computationally obtained for the [6,6] functionalization, in agreement with the experimental data (Figure 8a). In order to give a tentative assignment of the bis-adducts regioisomers experimentally obtained, all possible bis-regioisomers were computationally evaluated (Table S1). The most energetically favorable candidates are shown in Figure 8b, along with the corresponding Schlegel diagram of I_*h*_-C_80_ and the angles between the two addends measured with respect to the N atom at the center of the fullerene cage (CNC angle). The results show that the most stable isomers arise from a secondary functionalization at the 68–69 and 53–54 bonds, forming angles of 118.5° and 118.9°, respectively, relative to the first functionalization at the 1–9 bond. These angles are close to the 120° angle imposed by the inner Lu_3_N cluster. Strikingly, the latter is also consistent with the XRD structure of the mono-adduct, where the malonate addend is stabilized through noncovalent interactions with one of the cage clips. This stabilization directs functionalization toward the 68–69 and 53–54 bonds, oriented at ∼120°, consistent with the position of a second Lu atom of the Lu_3_N cluster and exposed toward the center of the contiguous window of the supramolecular cage (Figure 5).

**Figure 8:**
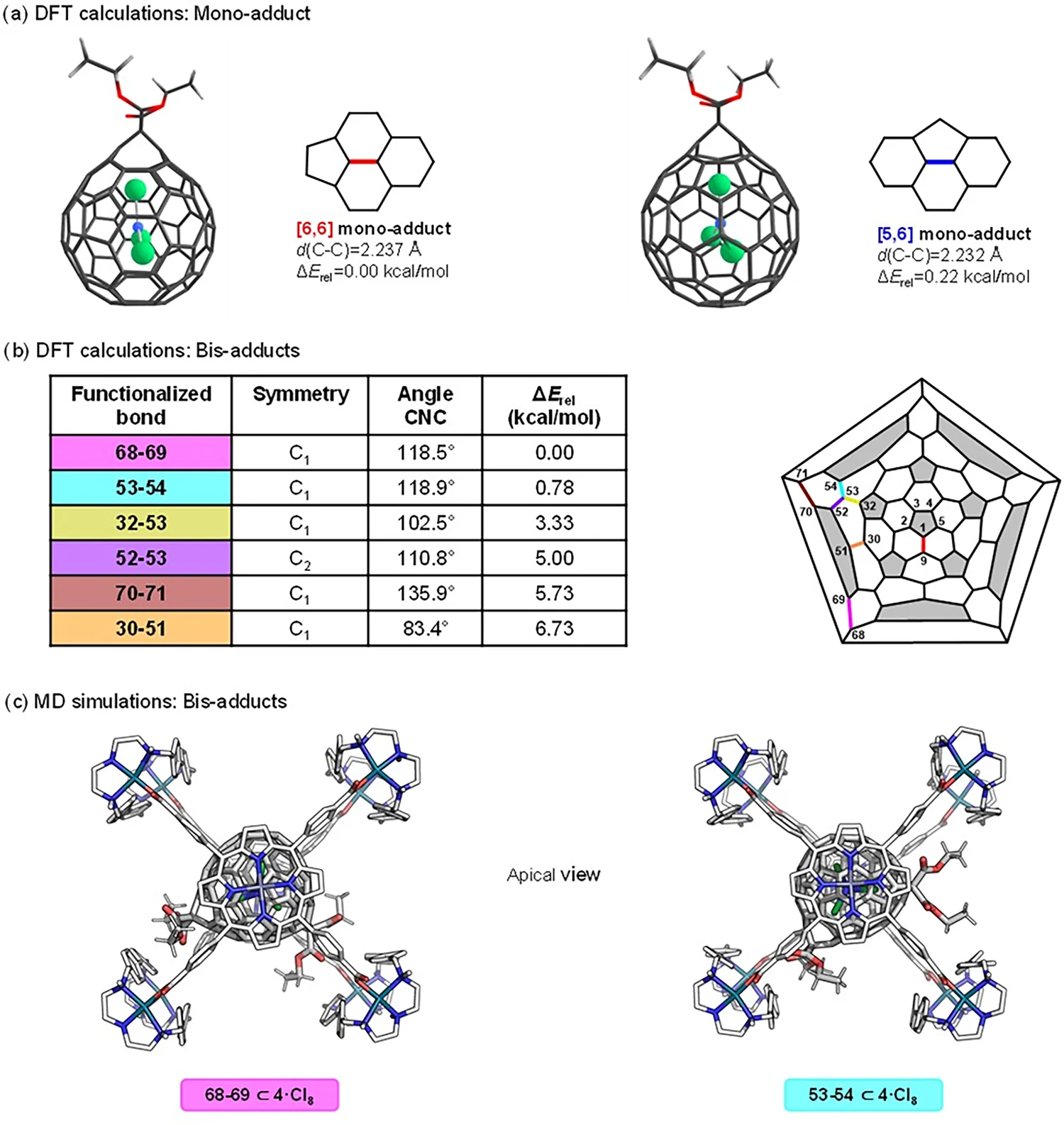
(a) DFT-calculated relative energies of [6,6] and [5,6] mono-adducts, showing the preference for the open [6,6] mono-adduct. (b) Table with relative energies of the 6 most stable bis-adducts and Schlegel diagram of I_*h*_-C_80_, where the bond involved in the first functionalization is highlighted in red, and the bonds of the most stable bis-adducts are color-coded according to the table. (c) MD simulation snapshots of the 68–69 bis-adduct isomer (left) and the 53–54 bis-adduct isomer (right) inside the nanocapsule.

Molecular Dynamics (MD) simulations have been carried out for the two more energetically favored bis-adducts, the 68–69 and the 53–54 (Figure 8c). The two bis-adducts are stable inside the nanocapsule, adopting a single orientation without observing rotation of the bis-adducts (Videos S2 and S3). In the case of bis-adduct 68–69, one substituent is interacting with one clip (as observed in the mono-adduct), and the second one is at the contiguous window also pointing toward a clip; while in 53–54 bis-adduct, the second substituent is placed centered at the contiguous window (Figure 8c). In both cases, the 120° angle among the addends stabilizes the encapsulation of these two bis-isomers.

An assignment of the bis-adducts’ identity, based on the data currently available, would be tentative. However, we can state with reasonable confidence that the two regioisomers on fraction II (δ and ε), obtained in equal amounts, could reasonably be assigned to the 68–69 and 53–54 bis-adducts, featuring a ∼120° angle between the addends. This assignment is supported by several lines of evidence: (a) DFT calculations identify these bis-adducts as the lowest-energy isomers; (b) the orientation of the mono-adduct, as observed in both the XRD structure and MD simulations, promotes secondary functionalization at ∼120°, exposing those bonds toward the gates of the supramolecular mask; (c) MD simulations of both bis-isomers inside the nanocapsule reveal stabilizing host–guest interaction between these isomers and the nanocapsule; and (d) the calculated dipole moments of the 68–69 and 53–54 bis-adducts are very similar, together with comparable CNC angles and relative energies, suggesting that these two regioisomers may correspond to the same chromatographic fraction II (Table S2).

To further investigate the generality of the supramolecular masking strategy, Sc_3_N@I_*h*_-C_80_ was also encapsulated within the nanocapsule and subjected to Bingel functionalization under analogous conditions. The encapsulated Sc_3_N@I_*h*_-C_80_ system showed efficient monofunctionalization, yielding the corresponding mono-adduct (in 92% yield) with shorter reaction times compared to Lu_3_N@I_*h*_-C_80_, in agreement with the higher intrinsic reactivity of Sc_3_N@I_*h*_-C_80_. The ^1^H NMR features of the encapsulated mono-adduct were highly similar to those observed for Lu_3_N@I_*h*_-C_80_, suggesting a comparable orientation within the nanocapsule. However, the higher reactivity of Sc_3_N@I_*h*_-C_80_ resulted in lower iteroselectivity during further functionalization steps, leading to mixtures of higher adducts. These results indicate that the supramolecular masking effect can be extended to different TNT-EMF species while being particularly effective for the less reactive Lu_3_N@I_*h*_-C_80_ system. Further details are provided in the Supporting Information. To assess the generality of this approach, we also explored the Diels–Alder reaction on the Lu_3_N@I_*h*_-C_80_ system with anthracene and pentacene. However, no reaction was observed, suggesting that the nanocapsule-induced enhancement of TNT-EMF reactivity is particularly effective for the Bingel reaction.

## Conclusions

Upon encapsulation in the tetragonal prismatic nanocapsule **4**·(BArF)_8_ supramolecular host, Bingel mono-adducts of M_3_N@I_*h*_-C_80_ (M= Lu, Sc) were obtained in excellent yield, in milder conditions and shorter times compared to reported procedures, thanks to the enhanced reactivity provided by the supramolecular mask. For the first time, we successfully obtained moderate yields of Lu_3_N@I_*h*_-C_80_ Bingel bis-adducts, starting from the host–guest complex Lu_3_N@I_*h*_-C_80_C­(CO_2_Et)_2_⊂**4**·(BArF)_8_. Crystal structure and MD studies of the encapsulated mono-adduct Lu_3_N@I_*h*_-C_80_C­(CO_2_Et)_2_ showed the necessary close interactions of the malonate addend toward one of the cage clips to render accessible bonds at the center of the contiguous gate for the functionalization of the second Bingel addend, aligned with the presence at 120° of a second Lu atom of the Lu_3_N inner cluster. Therefore, the Bingel reaction performed over encapsulated TNT-EMF is governed by the cluster preferred orientation, but it is also permitted by the supramolecular mask geometry. DFT calculations confirmed the preference of [6,6] open Lu_3_N@I_*h*_-C_80_C­(CO_2_Et)_2_ and provided an energetically ordered list of all bis-regioisomers. Based on DFT calculations, MD simulations, and the structural data obtained by X-ray diffraction, we assign two of the five isomers (out of 27 possible), obtained in fraction II in equal amounts, to isomers 68–69 and 53–54, both featuring a 120° angle between the addends.

In summary, we showed that the supramolecular mask approach for bare fullerene functionalization can also be applied to TNT-EMFs. Therefore, the Bingel reaction performed over encapsulated TNT-EMF is governed by the cluster positioning, but the supramolecular mask enhances its reactivity toward Bingel cyclopropanation and adapts to allow alignment with the inner cluster geometry, permitting a partial yet remarkable control over the regioselectivity. This work underlines the potential of supramolecular masks in tackling the challenge of TNT-EMF polyfunctionalization, opening the way to new materials for interesting applications.

## Supplementary Material









## Notes

Materials, instrumentation, experimental procedures, spectroscopic characterization of all compounds, X-ray crystallographic details, DFT computational details, and MD simulation details (PDF)
Lu_3_N@I_*h*_-C_80_C­(CO_2_Et)_2_⊂**4**·(BArF)_8_ (Video S1) (MPG)
Lu_3_N@I_*h*_-C_80_[C­(CO_2_Et)_2_]_2_⊂**4**·(BArF)_8_, 68–69 isomer (Video S2) (MPG)
Lu_3_N@I_*h*_-C_80_[C­(CO_2_Et)_2_]_2_⊂**4**·(BArF)_8_, 53–54 isomer (Video S3) (MPG)

## ∇

Institut Català d’Investigació Química (ICIQ), 43007 Tarragona, Catalonia, Spain

## Abbreviations

[10]­CPP: [10]­cycloparaphenylene
DFT: density functional theory
EMFs: endohedral metallofullerenes
MD: molecular dynamics
SMS: supramolecular mask strategy
TNT-EMFs: trimetallic nitride template endohedral metallofullerenes
