# Supplementary material for: Supramolecular Mask-Mediated Regioselective bis-Bingel-Functionalization of M3N@I h ‑C80 (M = Lu, Sc)
Source: J Am Chem Soc. 2026 Oct 16;148(42):46194–204. doi: 10.1021/jacs.6c16243 (PMC13721153; doi:10.1021/jacs.6c16243)
Supplement: Supplementary file 1 [file ja6c16243_si_001.pdf]

Supporting Information for:

Supramolecular mask-mediated regioselective bis-Bingel-functionalization of $M_3N@I_h$-C_{80} (M=Lu, Sc)

Valentina Iannace,[a] Clara Sabrià,[a] Sheng Zhu,[b] Olga A. Stasyuk,[a,c] Miquel Solà,[a] Teodor Parella,[d] Sergio Fernández,[a] Felipe Gándara,[e] Inhar Imaz,[f] Daniel Maspoch,[f,g] Jianyuan Zhang,*[b] Ferran Feixas,*[a] and Xavi Ribas*[a]

[a]Institut de Química Computacional i Catàlisi (IQCC) and Departament de Química, Universitat de Girona, Campus Montilivi, 17003 Girona, Catalonia (Spain). E-mail: ferran.feixas@udg.edu, xavi.ribas@udg.edu
[b] Department of Chemistry and Chemical Biology, Rutgers, The State University of New Jersey, Piscataway, New Jersey 08854 (United States). E-mail: jy.zhang@rutgers.edu
[c] Present Address: Institut Català d'Investigació Química (ICIQ), 43007 Tarragona, Catalonia (Spain)
[d] Servei de RMN, Facultat de Ciències, Universitat Autònoma de Barcelona, Campus UAB, 08193 Bellaterra, Catalonia, Spain;
[e] Materials Science Institute of Madrid – CSIC, 28049, Madrid (Spain)
[f] Catalan Institute of Nanoscience and Nanotechnology (ICN2), CSIC and The Barcelona Institute of Science and Technology, Campus UAB, 08193 Bellaterra, Catalonia (Spain)
[g] ICREA, Pg. Lluís companys 23, 08010 Barcelona, Catalonia (Spain)

Table of Contents

1. Materials Methods and Instrumentation 3

2. Experimental section 6

2.1. Synthesis and characterization of $M_3N@I_h$-$C_{80}\subset 4\cdot(BArF)_8$ 6

2.1.1. Preparation of $Lu_3N@I_h$-$C_{80}\subset 4\cdot(BArF)_8$ 6

2.1.2. Characterization of $Lu_3N@I_h$-$C_{80}\subset 4\cdot(BArF)_8$ 7

2.1.3. Preparation of $Sc_3N@I_h$-$C_{80}\subset 4\cdot(BArF)_8$ 19

2.1.4. Characterization of $Sc_3N@I_h$-$C_{80}\subset 4\cdot(BArF)_8$ 20

2.2. Mask-mediated synthesis of $M_3N@I_h$-$C_{80}C(CO_2Et)_2\subset 4\cdot(BArF)_8$ 27

2.2.1. Synthesis of $Lu_3N@I_h$-$C_{80}C(CO_2Et)_2\subset 4\cdot(BArF)_8$ via Bingel reaction 27

2.2.2. Characterization of $Lu_3N@I_h$-$C_{80}C(CO_2Et)_2\subset 4\cdot(BArF)_8$ 30

2.2.3. Release and characterization of $Lu_3N@I_h$-$C_{80}C(CO_2Et)_2$ 39

2.2.4. Characterization of $Lu_3N@I_h$-$C_{80}C(CO_2Et)_2$ 41

2.2.5. Synthesis of $Sc_3N@I_h$-$C_{80}C(CO_2Et)_2\subset 4\cdot(BArF)_8$ via Bingel reaction 46

2.2.6. Characterization of $Sc_3N@I_h$-$C_{80}C(CO_2Et)_2\subset 4\cdot(BArF)_8$ 49

2.3. Mask-mediated synthesis of $M_3N@I_h$-$C_{80}[C(CO_2Et)_2]_2\subset 4\cdot(BArF)_8$ via Bingel reaction 56

2.3.1. Synthesis and characterization of $Lu_3N@I_h$-$C_{80}[C(CO_2Et)_2]_2$ via Bingel reaction starting from $Lu_3N@I_h$-$C_{80}\subset 4\cdot(BArF)_8$ 56

2.3.2. Synthesis and characterization of $Lu_3N@I_h$-$C_{80}[C(CO_2Et)_2]_2$ via Bingel reaction starting from $Lu_3N@I_h$-$C_{80}C(CO_2Et)_2$ 58

2.3.3. Synthesis and characterization of $Lu_3N@I_h$-$C_{80}[C(CO_2Et)_2][^{13}C(^{13}CO_2Et)_2]\subset 4\cdot(BArF)_8$ via Bingel-Hirsch Reaction 68

2.3.4. Synthesis and characterization of $Sc_3N@I_h$-$C_{80}[C(CO_2Et)_2][^{13}C(^{13}CO_2Et)_2]\subset 4\cdot(BArF)_8$ via Bingel-Hirsch Reaction 74

2.4. Control experiments with labelled malonate 76

2.4.1. Synthesis and characterization of $Lu_3N@I_h$-$C_{80}C(CO_2Et)_2\subset 4\cdot(BArF)_8$ via Bingel-Hirsch Reaction 77

2.4.2. Synthesis and characterization of $Lu_3N@I_h$-$C_{80}[^{13}C(^{13}CO_2Et)_2]\subset 4\cdot(BArF)_8$ via Bingel-Hirsch Reaction 80

2.5. Cyclic Voltammetry of Host-guest complexes 84

2.6. UV-Vis Spectra of Host-Guest complexes 85

2.7. Control Experiments without the nanocapsule 87

2.8. Stability of the Host-Guest complex under basic conditions 88

3. X-Ray Diffraction 89

4. DFT Calculations 90

5. Molecular Dynamics simulations 96

6. Supporting Videos 99

7. References 100

1. Materials Methods and Instrumentation

Reagents and solvents used were commercially available reagent quality unless indicated otherwise. The synthesis of the nanocapsule has been performed following the reported procedures[1] for **4**·$(BArF)_8$.

NMR data concerning product characterization were collected on Bruker 400 MHz AVANCE spectrometers in $CDCl_3$ or CD_3CN, and calibrated relative to the residual signal of the solvent. ^{13}C NMR experiments on very dilute ^{13}C-labelled samples were collected in a 500 MHz NMR spectrometer equipped with a highly sensitive TCI cryoprobe in long overnight/weekend acquisition periods.

ESI-MS experiments were collected and analysed on Bruker MicroTOF-Q-II using CH_3CN as a mobile phase. MALDI-TOF-MS analysis were collected on an Autoflex maX Bruker Daltonics with a Bruker Smartbeam II (355 nm wavelength) using DCTB/NaTFA as matrix.

HPLC data concerning fullerene adducts identity were collected on Agilent Technologies LC 1200 series instrument equipped with a Cosmosil PYE column (4.6 mm I.D. x 250 mm, particle size: 5 µm, Nacalai Tesque, Inc.) and using toluene as solvent (1 ml/min flow), monitored with a diode array UV-Vis detector at 320 nm to observe the UV-Vis spectra characteristics of each isomer.

Cyclic Voltammetry experiments were carried out inside an N_2-filled glovebox using a CHI620D potentiostat in a single-compartment, three-electrode configuration. A glassy carbon disk working electrode (3 mm diameter), a platinum counter electrode, and a silver wire pseudo-reference electrode were employed. The pseudo-reference electrode was separated from the analyte solution by a porous PTFE fritted salt bridge containing the same electrolyte solution. All potentials were externally calibrated against the ferrocene/ferrocenium ($Fc^{0/+}$) redox couple. Voltammograms were recorded with an initial anodic scan from −0.4 V toward positive potentials vs $Fc^{0/+}$.

X-Ray Diffraction data were collected in ALBA synchrotron.

DFT Calculations

Geometry optimization of the mono- and bis-adducts was performed using the DFT B3LYP functional[2,3] with D3(BJ) dispersion correction[4,5] and def2-SVP basis set.[6,7] For efficient calculations, effective core potentials for Lu Def2-ECP (replacing 28 core electrons)[8] and resolution of identity (RI) approximation were used as implemented in the ORCA 4.1.2 program.[9,10] Solvent effects were accounted using a Conductor-like Polarizable Continuum Model (CPCM) with acetonitrile as a solvent (dielectric constant $\varepsilon = 36.6$).[11] The adducts were treated in the neutral form (charge 0, multiplicity 1). For the assessment of the relative energies of various isomers, single point energy calculations at the B3LYP-D3(BJ)/def2-TZVP level were additionally performed on the B3LYP-D3(BJ)/def2-SVP optimized geometries. Such multi-level approach is common in computational chemistry and allows for large savings in computational resources without significant loss of accuracy. The molecular structures were visualized with Chemcraft 1.8.[12]

MD simulations

The parametrization of cage **4**·Cl_8 used in the molecular dynamic (MD) simulations was derived from our previous work[13]. The force-filed parameters were obtained from a protocol that combines the General Amber Force Field (GAFF)[14] and the Metal Center Parameter Builder (MCPB.py)[15] tools, with crystallographic X-ray data as the starting structure of the nanocapsule. See Supporting Information of reference[13] for a complete description of the force-field parametrization strategy and parameters of **4**·Cl_8. For the endohedral metallofullerenes (EMFs) parameters were obtained in two-step protocol, treating separately the fullerenes and the metal cluster Lu_3N. First, the fullerenes (C_{80}, mono-C_{80} and bis-isomers 68-69 and 53-54) were optimized at the B3LYP/def2-TZVP level with Gaussian 16 package[16] The electrostatic potential was then computed at the same level of theory, and the corresponding atomic charges were derived. This data was used to generate the initial force-filed parameters with the antechamber module of Amber 22[17] following standard GAFF procedures. In parallel, the metal cluster Lu_3N was optimized and its charges computed at the same level of theory as the fullerenes. Force-field parameters were obtained using the MCPB.py workflow. However, initial MD simulations revealed incompatibilities arising from the partial charges assigned to the Lu atoms. For this reason, new atomic charges for the complete EMF system (fullerene + Lu3N) were computed with Gaussian 16. Mulliken charges were adopted and manually reassigned to the previous parameters.

Molecular dynamics simulations were carried out with acetonitrile (MeCN) as solvent and the GPU code (pmemd) of the AMBER 18 package[18]. The system was immersed in a preequilibrated truncated octahedron box with a 10 Å buffer of MeCN molecules using the leap module and neutralized by adding eight explicit Cl^- as a model counterion for $BArF^-$. This simulation setup has been validated in our previous work, where it reproduced experimental encapsulation trends and host-guest dynamics in close agreement with experiment[19]. Then, each complex was submitted to a two-stage geometry optimization approach. The first stage minimizes the positions of solvent molecules and ions imposing positional restraints on the solute by harmonic potential with a force constant of 500 kcal

mol^{-1} Å^{-2}. The second stage corresponds to an unrestrained minimization of all the atoms in the simulation cell. The system was gently heated using six 50 ps steps, where the temperature is increased by 50 K each step (0-300K) under constant volume and periodic boundary conditions. To constraint the covalent bonds containing hydrogens it was employed the SHAKE algorithm. Long-range electrostatic effects were modelled using the particle-mesh Ewald method. An 8 Å cutoff was applied to Lennard-Jones and electrostatic interactions. Harmonic restraints of 10 kcal mol^{-1} were applied to the solute and the Langevin equilibration scheme was used to control and equalize the temperature. The time step was maintained at 2 fs during the heating steps, allowing potential in homogeneities to self-adjust. Then, each system was equilibrated without restrains for 10 ns with a 2 fs time steps at a constant pressure and temperature of 300 K. Each EMF was placed inside the cage as starting point and 2 replicates of 0.5 µs have been carried out for each system.

NCIPLOT was used to calculate the Non-covalent Interaction (NCI) volume between the supramolecular cage **4**·Cl_8 and the two bis-adduct isomers (68-69 and 53-54) along all the simulation time for one MD replicate[20,21]. It has been calculated without taking into account the solvent molecules. The results were visualized with PyMOL using a rainbow scale, where the repulsive interactions are represented in red, the attractive interactions in blue and weak van der Waals interactions in green.

2. Experimental section

2.1. Synthesis and characterization of $M_3N@I_h\text{-}C_{80}\subset 4\cdot(BArF)_8$

2.1.1. Preparation of $Lu_3N@I_h\text{-}C_{80}\subset 4\cdot(BArF)_8$

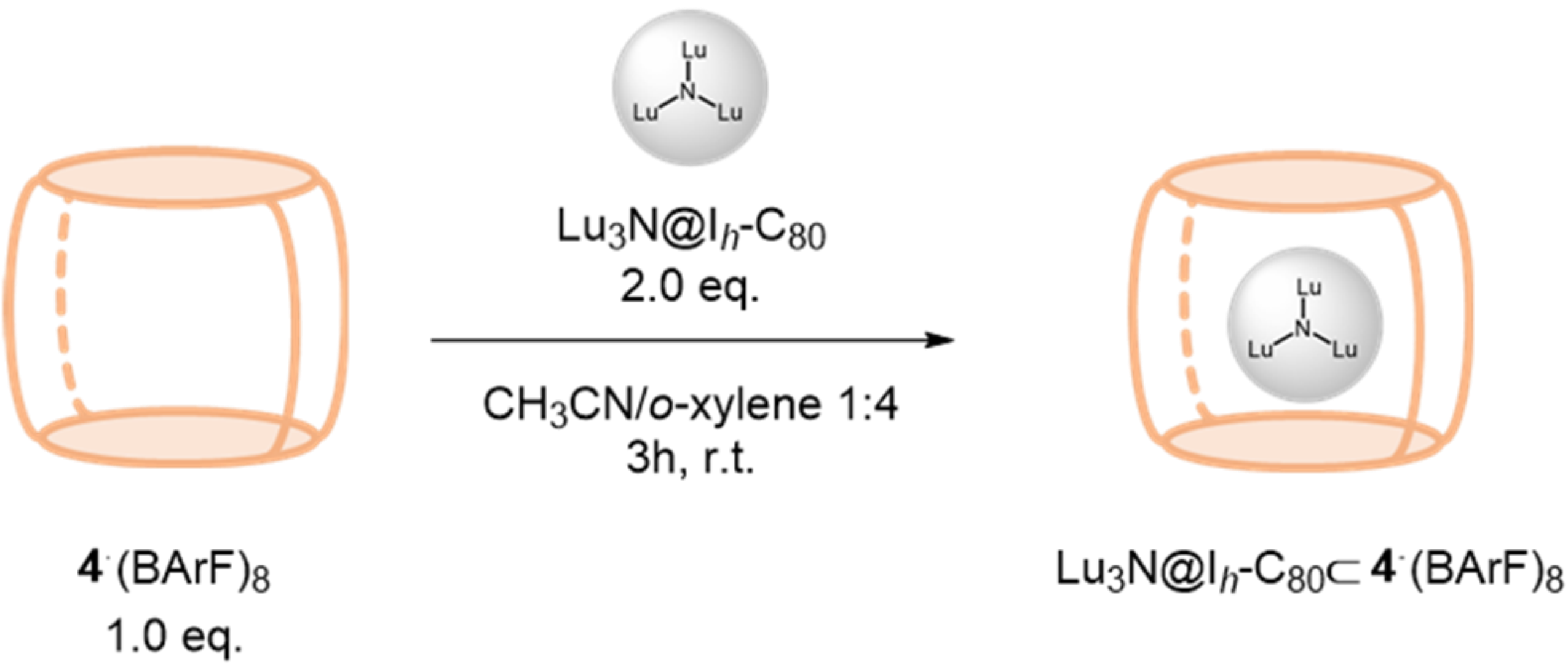

Figure S1. Procedure for the synthesis of $Lu_3N@I_h\text{-}C_{80}\subset$**4**$\cdot(BArF)_8$ host-guest complex.

15 mg of **4**·$(BArF)_8$ nanocapsule (1 equiv.) are dissolved with CH_3CN (1 mL). Then, 2 equivalents of $Lu_3N@I_h\text{-}C_{80}$ (3.73 mg) dissolved in *o*-xylene (4 mL) are added to the nanocapsule solution and stirred at room temperature for 3 h. After this time, the solvent is removed under reduced pressure, CH_3CN is added to solubilize the product, filtering with disk to eliminate the excess of $Lu_3N@I_h\text{-}C_{80}$ (that can be recovered adding *o*-xylene to the disk). The product is dried under reduced pressure and obtained pure by crystallization in DCM/MeCN/Diethyl Ether (See section 4. for details on the X ray diffraction) (Yield=98%).

2.1.2. Characterization of $Lu_3N@I_h$-C_{80}⊂4·(BArF)$_8$

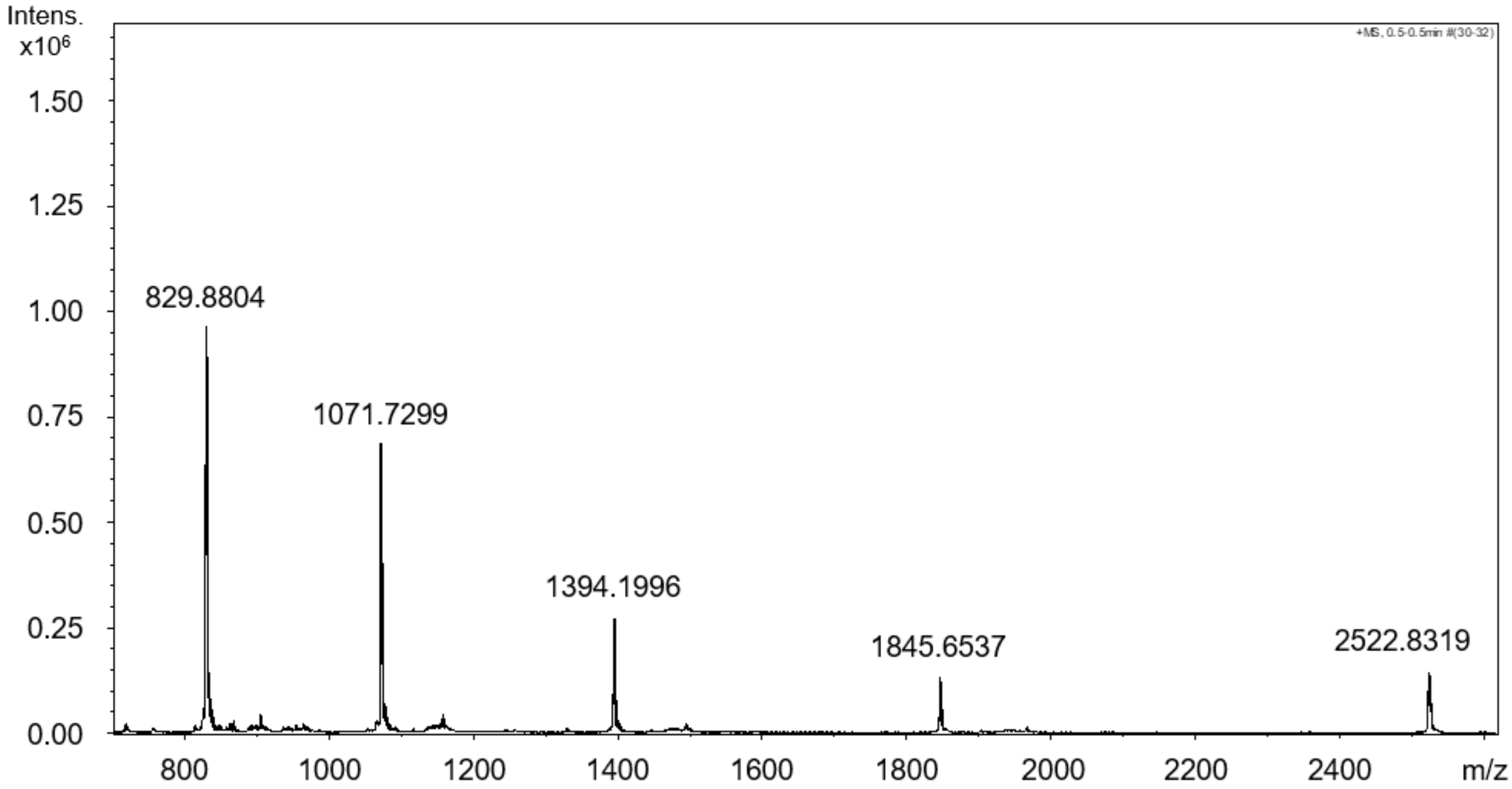

Figure S2. HR-MS spectrum of $Lu_3N@I_h$-C_{80}⊂**4**·(BArF)$_8$. Sample solved in CH_3CN and registered with a Bruker MicroTOF-Q-II mass spectrometer.

HR-MS (m/z):

calculated 829.8912 and found 829.8804 ($Lu_3N@I_h$-C_{80}⊂[**4**·(BArF)$_0$]$^{+8}$);

calculated 1071.7424 and found 1071.7299 ($Lu_3N@I_h$-C_{80}⊂[**4**·(BArF)$_1$]$^{+7}$);

calculated 1394.2107 and found 1394.1996 ($Lu_3N@I_h$-C_{80}⊂[**4**·(BArF)$_2$]$^{+6}$);

calculated 1845.6663 and found 1845.6537 ($Lu_3N@I_h$-C_{80}⊂[**4**·(BArF)$_3$]$^{+5}$);

calculated 2522.8497 and found 2522.8319 ($Lu_3N@I_h$-C_{80}⊂[**4**·(BArF)$_4$]$^{+4}$).

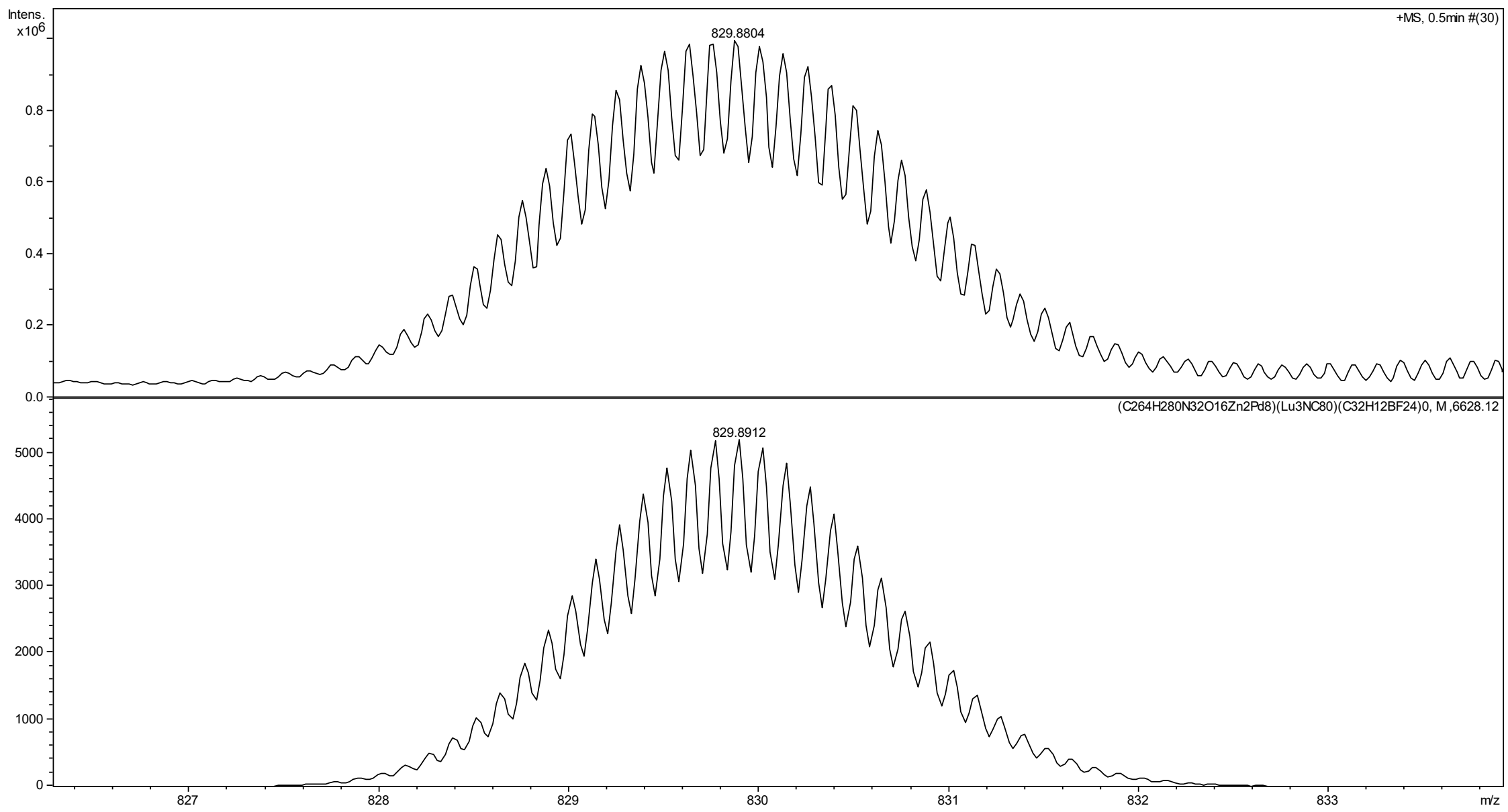

Figure S3. High resolution expanded view of the isotopic envelopes for $Lu_3N@I_h$-C_{80}⊂**4**·$(BArF)_8$. calculated 829.8912 (bottom) and found 829.8804 (top) ($Lu_3N@I_h$-C_{80}⊂[**4**·$(BArF)_0]^{+8}$).

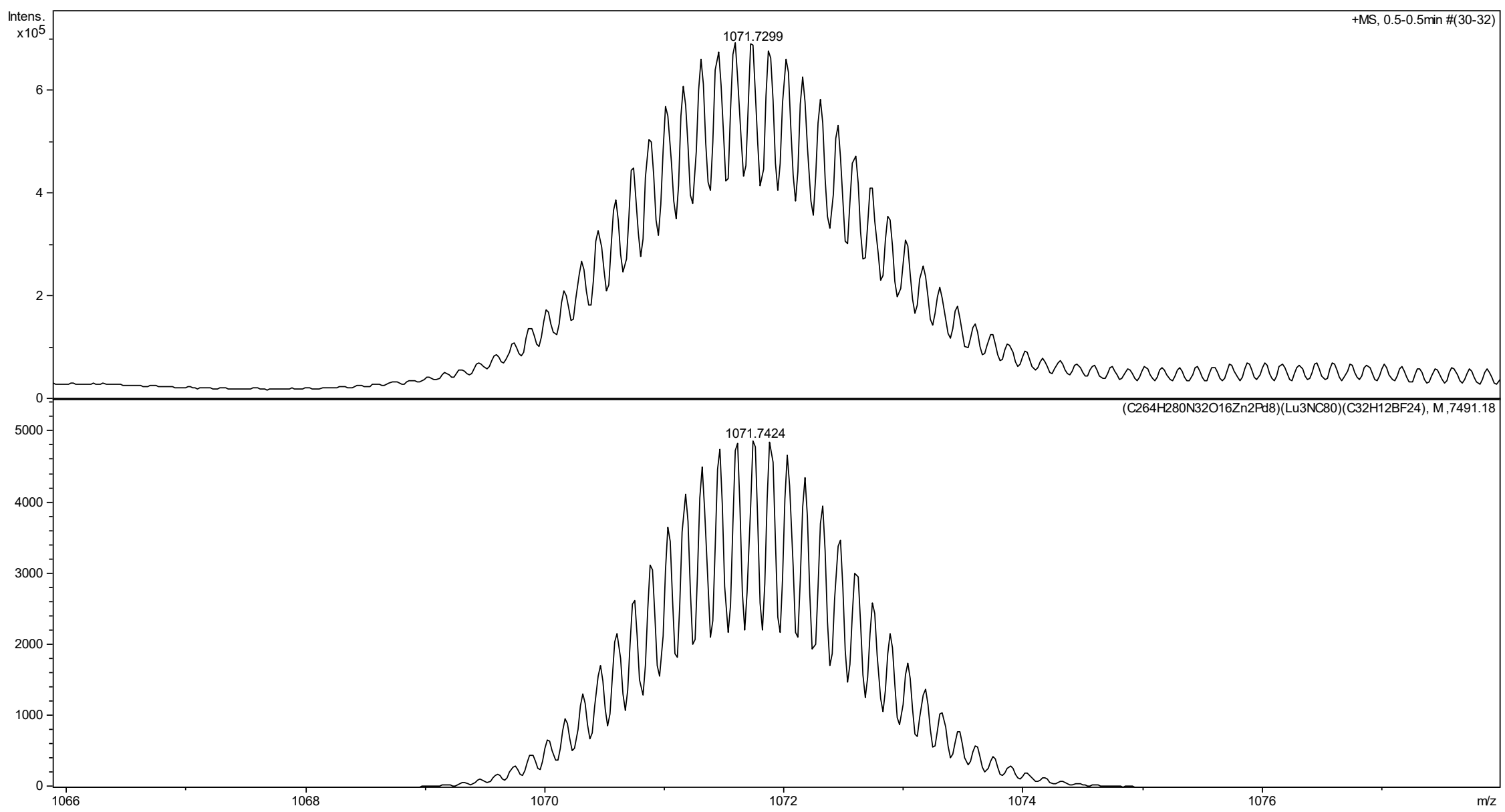

Figure S4. High resolution expanded view of the isotopic envelopes for $Lu_3N@I_h$-C_{80}⊂**4**·$(BArF)_8$. calculated 1071.7424 (bottom) and found 1071.7299 (top) ($Lu_3N@I_h$-C_{80}⊂[**4**·$(BArF)_1]^{+7}$).

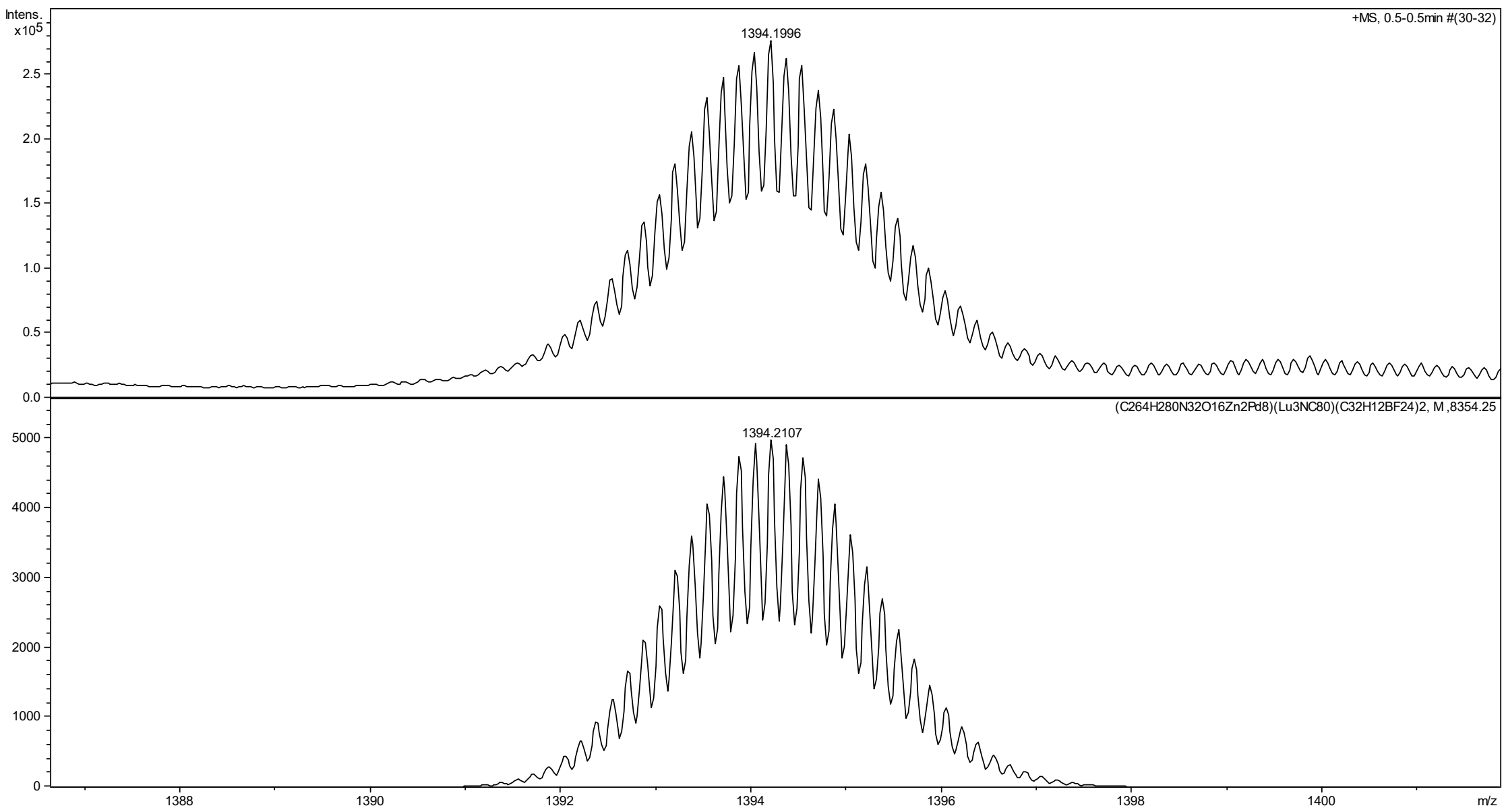

Figure S5. High resolution expanded view of the isotopic envelopes for $Lu_3N@I_h$-C_{80}⊂**4**·(BArF)$_8$. calculated 1394.2107 (bottom) and found 1394.1996 (top) ($Lu_3N@I_h$-C_{80}⊂[**4**·(BArF)$_2$]$^{+6}$).

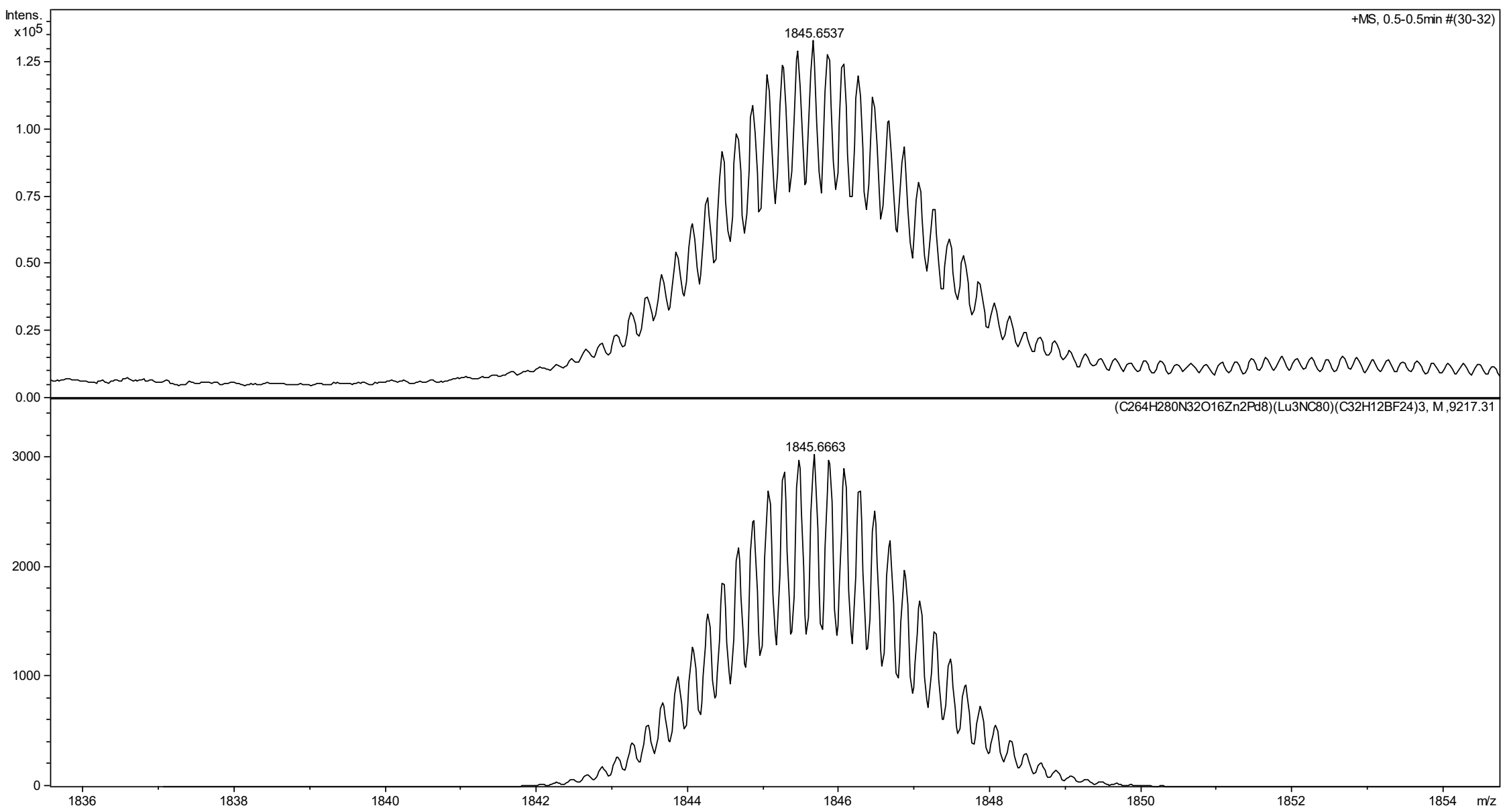

Figure S6. High resolution expanded view of the isotopic envelopes for $Lu_3N@I_h$-C_{80}⊂**4**·(BArF)$_8$. calculated 1845.6663 (bottom) and found 1845.6537 (top) ($Lu_3N@I_h$-C_{80}⊂[**4**·(BArF)$_3$]$^{+5}$).

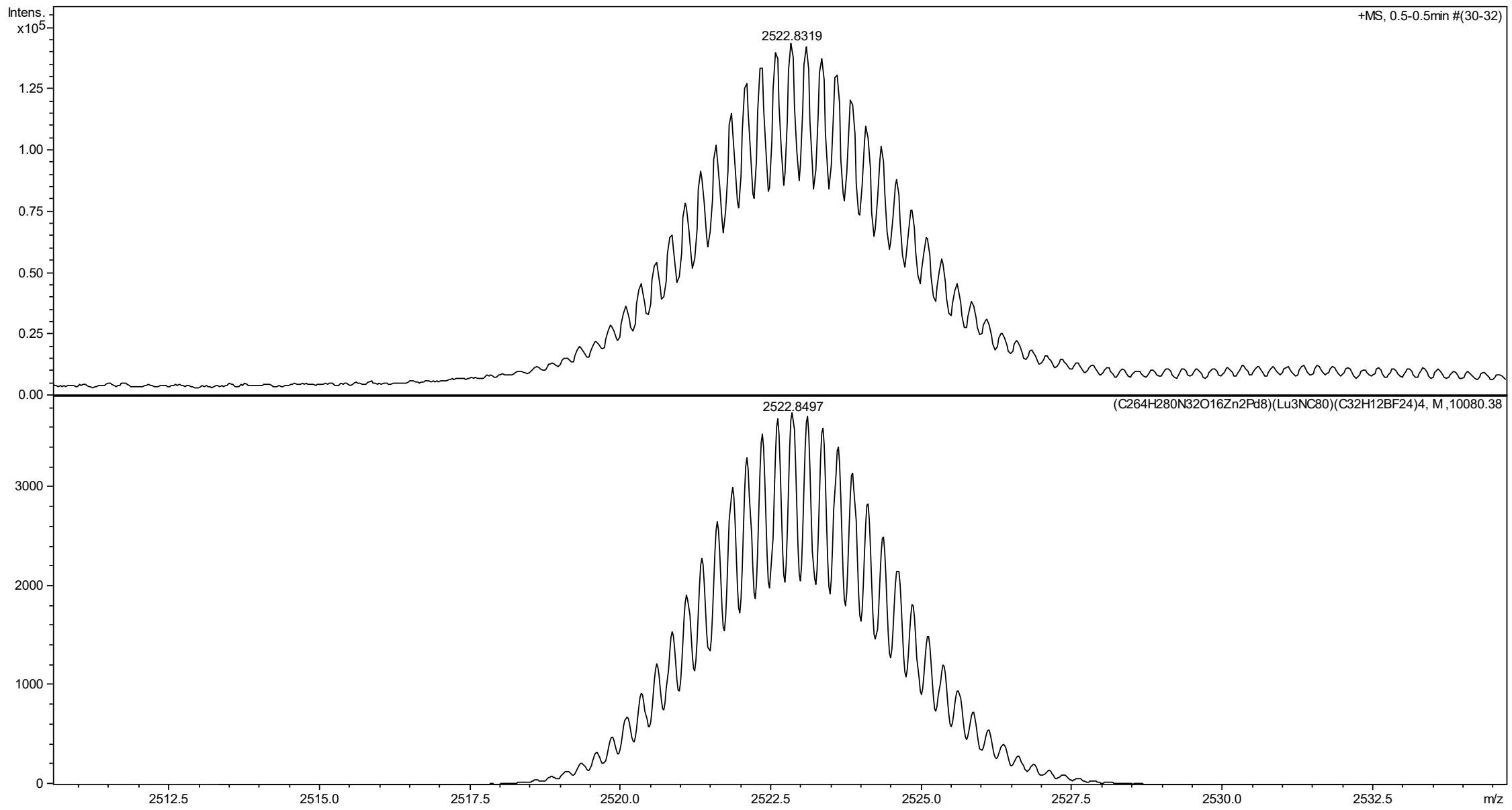

Figure S7. High resolution expanded view of the isotopic envelopes for $Lu_3N@I_h$-C_{80}⊂**4**·$(BArF)_8$. calculated 2522.8497 (bottom) and found 2522.8319 (top) ($Lu_3N@I_h$-C_{80}⊂[**4**·$(BArF)_4$]$^{+4}$).

NMR Characterization

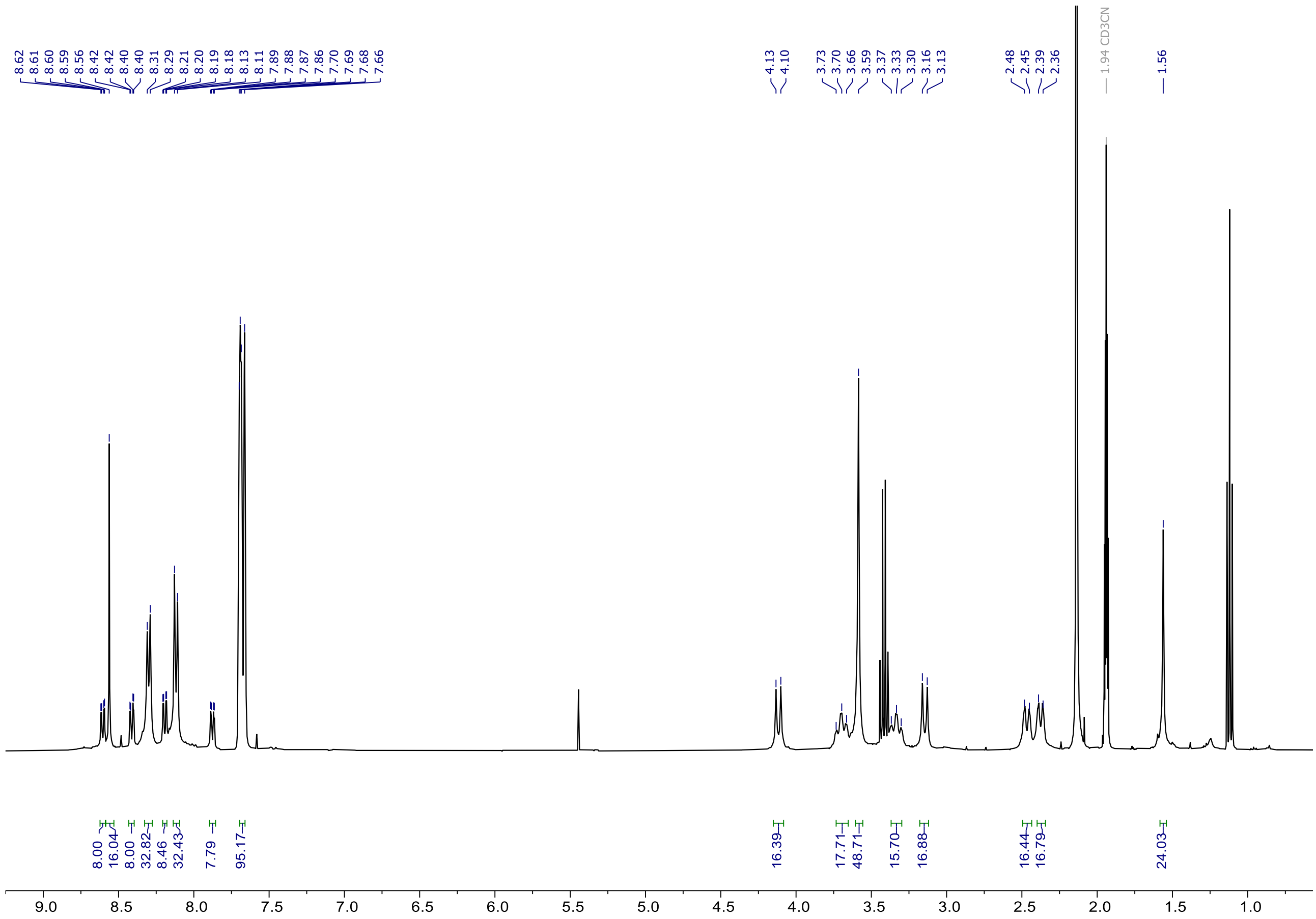

Figure S8. ^{1}H-NMR spectrum of $Lu_3N@I_h$-C_{80}⊂**4**·$(BArF)_8$. Experiment performed in CD_3CN at 298K (400 MHz).

^{1}H-NMR (400 MHz, CD_3CN) δ p.p.m.: 8.61 (dd, *J*=7.7, 1.8 Hz, 8H, arom-porph), 8.56 (s, 16H, pyrrole ring), 8.42 (dd, *J*=8.0, 1.8 Hz, 8H, arom-porph), 8.30 (d, *J*=7.9 Hz, 32H, arom-clip), 8.19 (dd, *J*=7.9, 1.9 Hz, 8H, arom-porph), 8.12 (d, *J*=8.4 Hz, 32H, arom-clip), 7.88 (dd, *J*=7.8, 1.9 Hz, 8H, arom-porph),7.72-7.65 (m, 96H, $BArF^-$), 4.12 (d, *J*=12.9 Hz, 16H, $-CH_2-$), 3.70 (dt, *J*=13.5,7.6 Hz, 16H, $-CH_2-$), 3.59 (s, 48H, $N-CH_3$), 3.33 (dt, *J*=13.5, 7.6 Hz, 16H, $-CH_2-$), 3.14 (d, *J*=13.0 Hz, 16H, $-CH_2-$), 2.47 (d, *J*=12.9 Hz, 16H, $-CH_2-$), 2.37 (d, *J*=12.9 Hz, 16H, $-CH_2-$) 1.56 (s, 24H, $N-CH_3$).

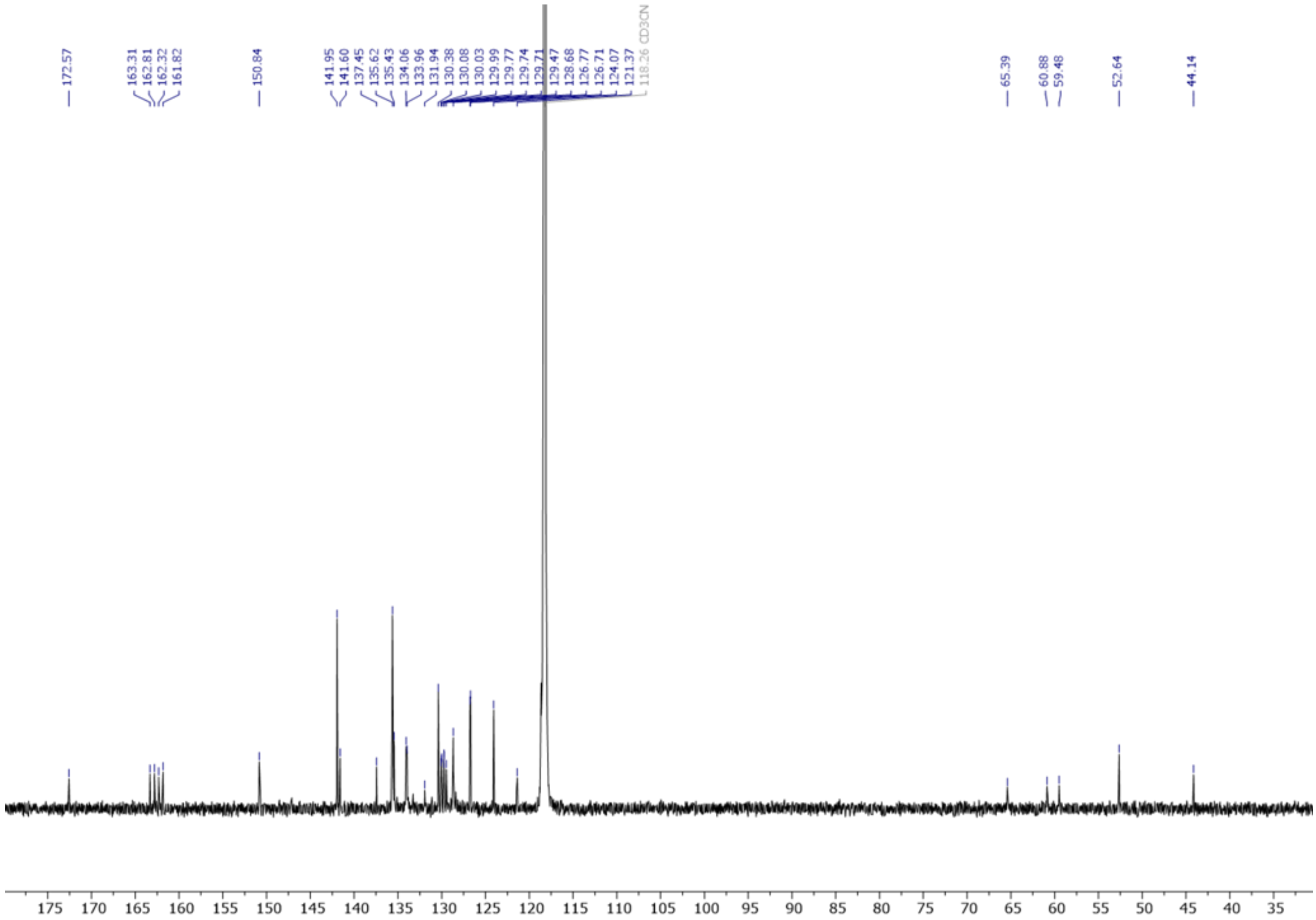

Figure S9. ^{13}C-NMR spectrum of $Lu_3N@I_h$-$C_{80}\subset$**4**·$(BArF)_8$. Experiment performed in CD_3CN at 298K (100 MHz).

^{13}C-NMR (100 MHz, CD_3CN) δ p.p.m.:172.57 (COO^-) 163.31-161.82 ($BArF^-$), 150.84 (pyrrole ring) 135.62 ($BArF^-$),132.13 (arom-porph),134.06 (arom-clip), 133.21 (arom-porph), 131.94 (pyrrole), 128.68 (arom-porph, arom-clip), 128.53 (arom-porph), 65.39 ($-CH_2-$), 60.88 ($-CH_2-$), 59.48 ($-CH_2-$), 52.64 ($-CH_3$), 44.14 ($-CH_3$).

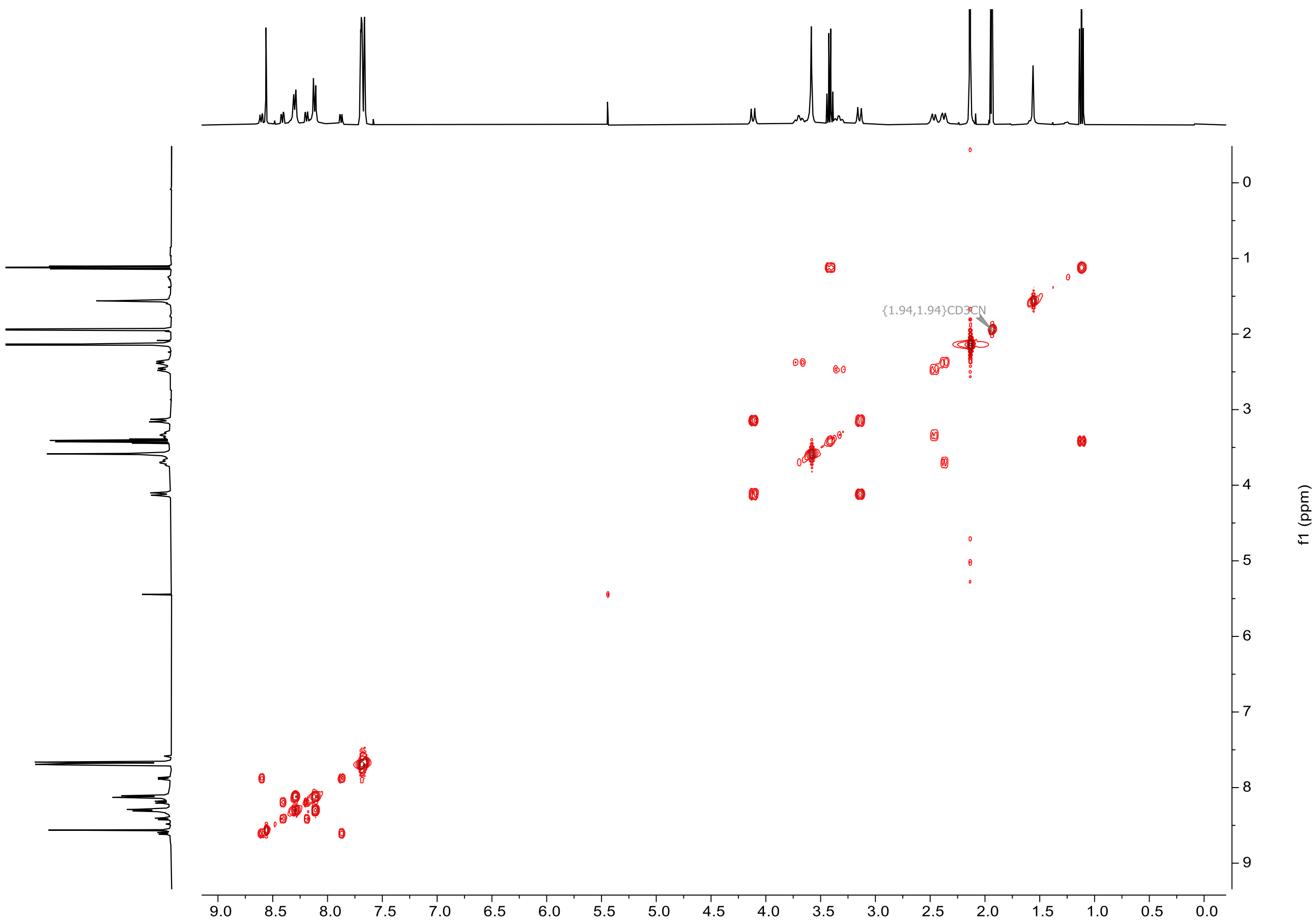

Figure S10. COSY of $Lu_3N@I_h$-C_{80}⊂**4**·$(BArF)_8$. Experiment performed in CD_3CN at 298K (400 MHz).

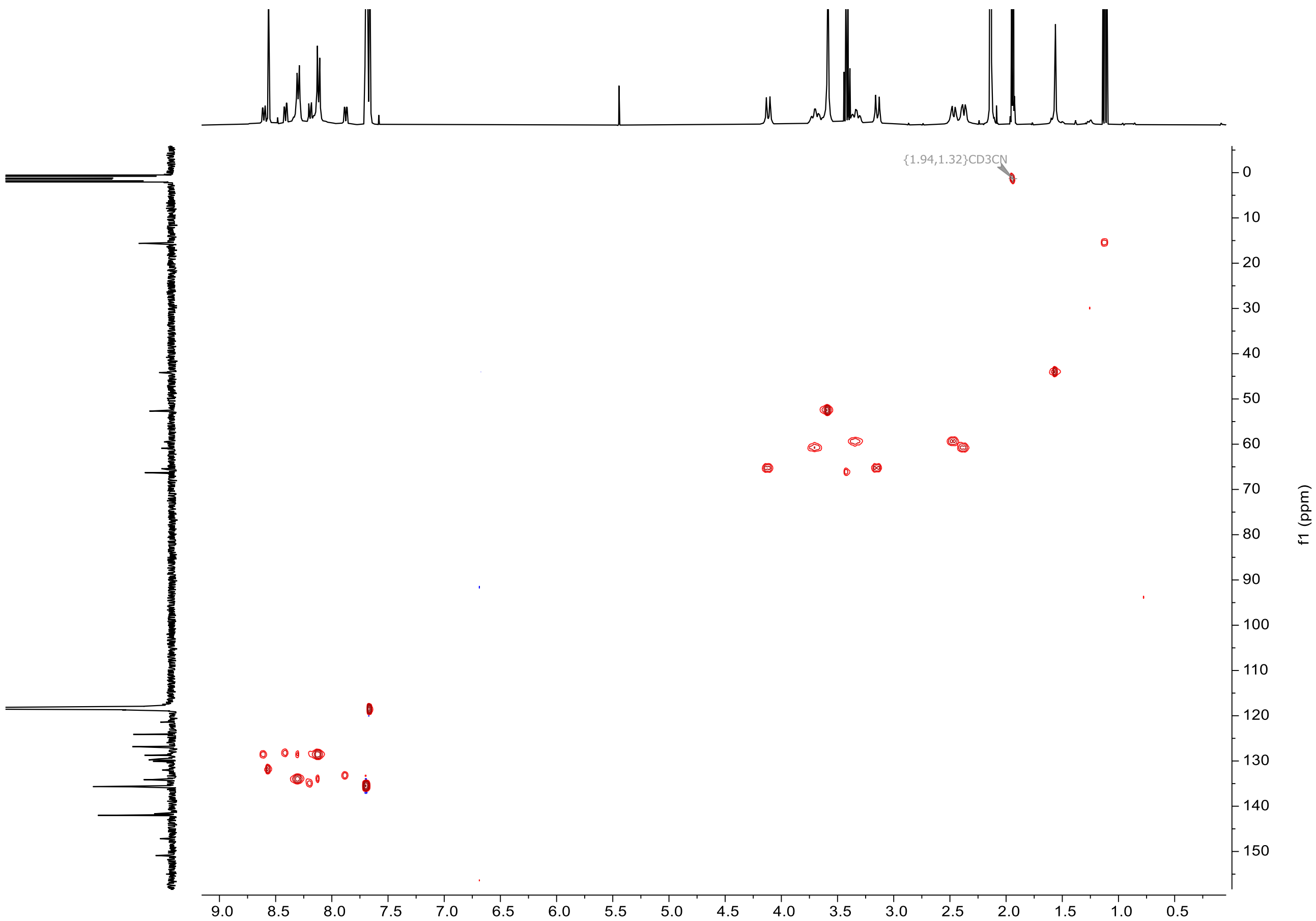

Figure S11. HSQC of $Lu_3N@I_h\text{-}C_{80}\subset$**4**$\cdot(BArF)_8$. Experiment performed in CD_3CN at 298K (400 MHz).

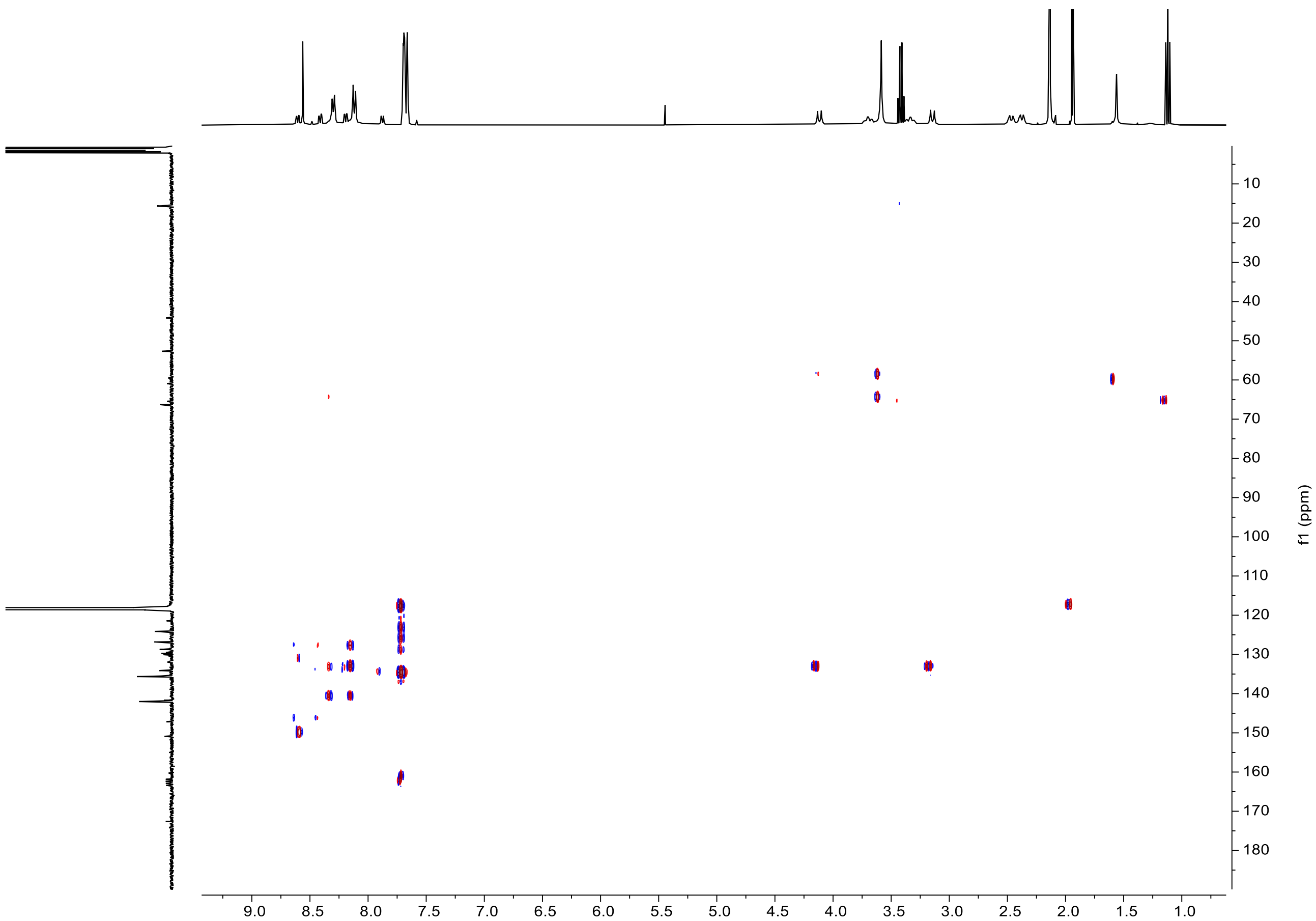

Figure S12. HMBC of $Lu_3N@I_h\text{-}C_{80}\subset$**4**$\cdot(BArF)_8$. Experiment performed in CD_3CN at 298K (400 MHz).

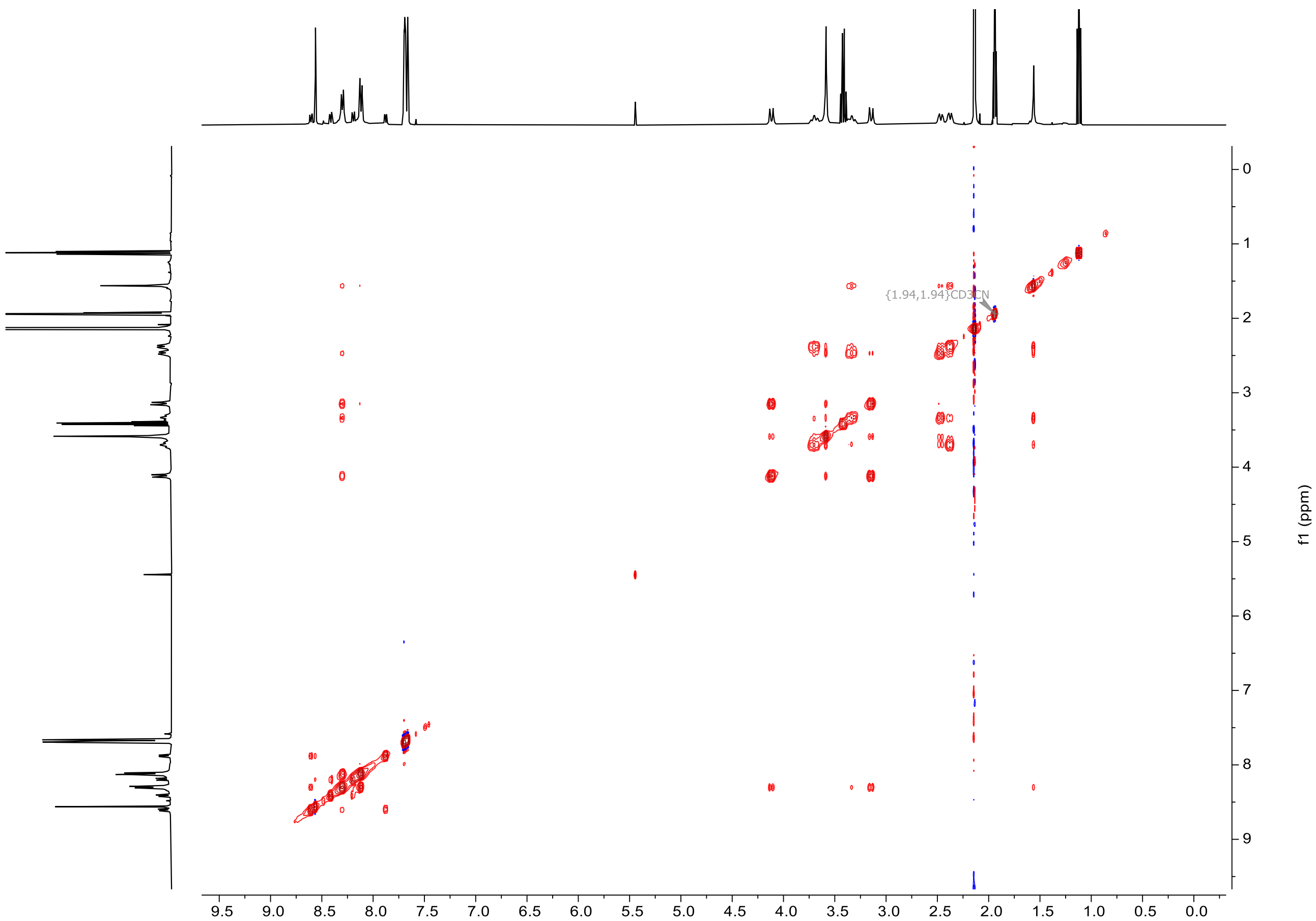

Figure S13. NOESY of $Lu_3N@I_h\text{-}C_{80}\subset\mathbf{4}\cdot(BArF)_8$. Experiment performed in CD_3CN at 298K (400 MHz).

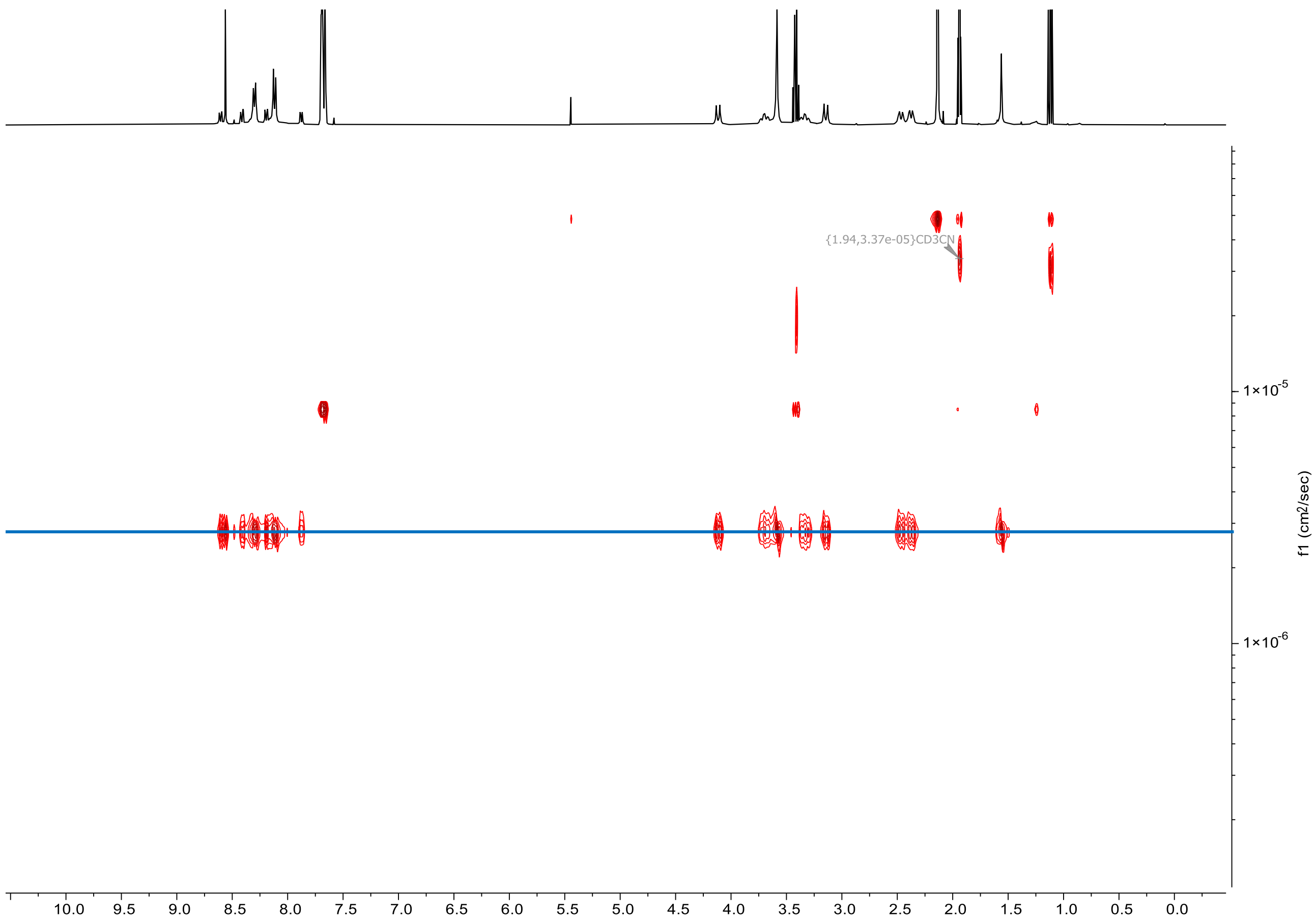

Figure S14. DOSY NMR Spectrum of $Lu_3N@I_h\text{-}C_{80}\subset$**4**$\cdot(BArF)_8$. Experiment performed in CD_3CN at 298K. D= 2.75×10^{-6} cm^2/s.

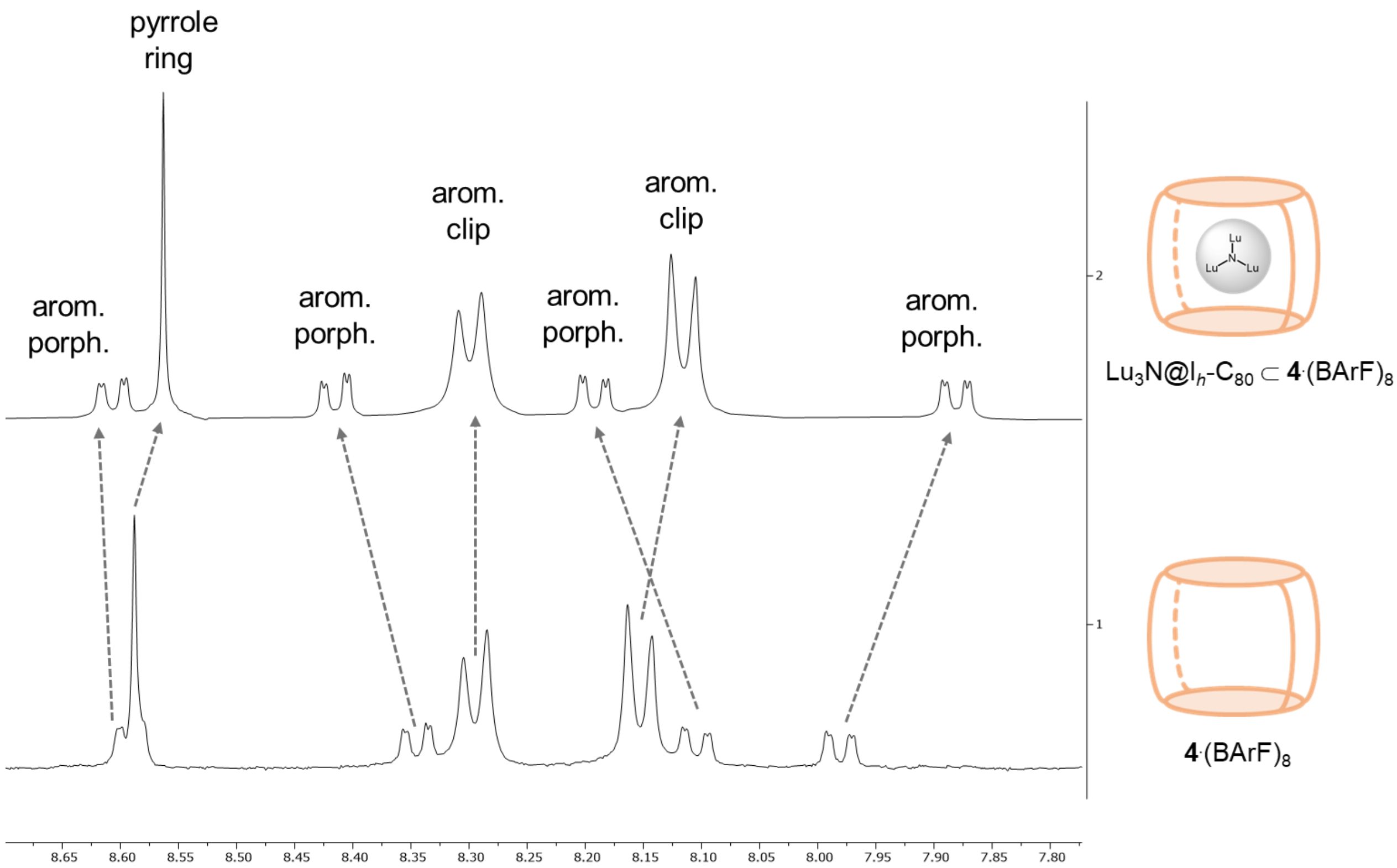

Figure S15. Comparative ^{1}H-NMR spectra (CD_3CN, 400 MHz, 298 K) of $Lu_3N@I_h$-C_{80}⊂**4·**$(BArF)_8$ (up) and empty **4·**$(BArF)_8$ (down). Changes in the chemical shift upon encapsulation, focus on the aromatic region of the spectra.

2.1.3. Preparation of $Sc_3N@I_h\text{-}C_{80}\subset$**4**·$(BArF)_8$

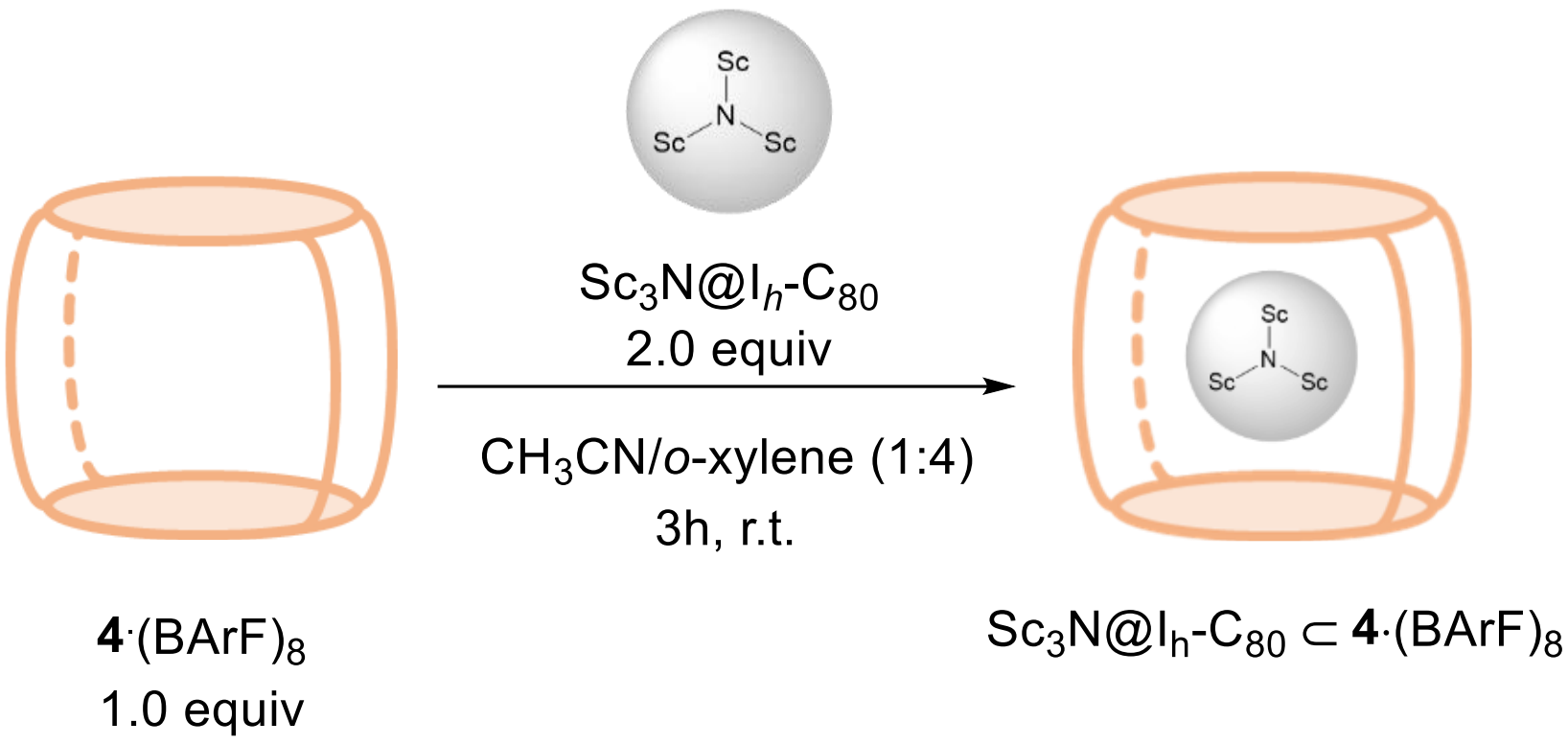

Figure S16. Procedure for the synthesis of $Sc_3N@I_h\text{-}C_{80}\subset$**4**·$(BArF)_8$ host-guest complex.

15 mg of **4**·$(BArF)_8$ nanocapsule (1 equiv.) are dissolved with CH_3CN (1 mL). Then 2 equivalents of $Sc_3N@I_h\text{-}C_{80}$ (2.76 mg) dissolved in *o*-xylene (4 mL) are added to the nanocapsule solution and stirred at room temperature for 3 h. After this time the solvent is removed under reduced pressure, CH_3CN is added to solubilize the product filtering with disk to eliminate the excess of $Sc_3N@I_h\text{-}C_{80}$ (that can be recovered adding *o*-xylene to the disk). The product is dried under reduced pressure and obtained pure by crystallization in DCM/MeCN/Diethyl Ether. (Yield=97%).

2.1.4. Characterization of $Sc_3N@I_h$-C_{80}⊂4·(BArF)$_8$

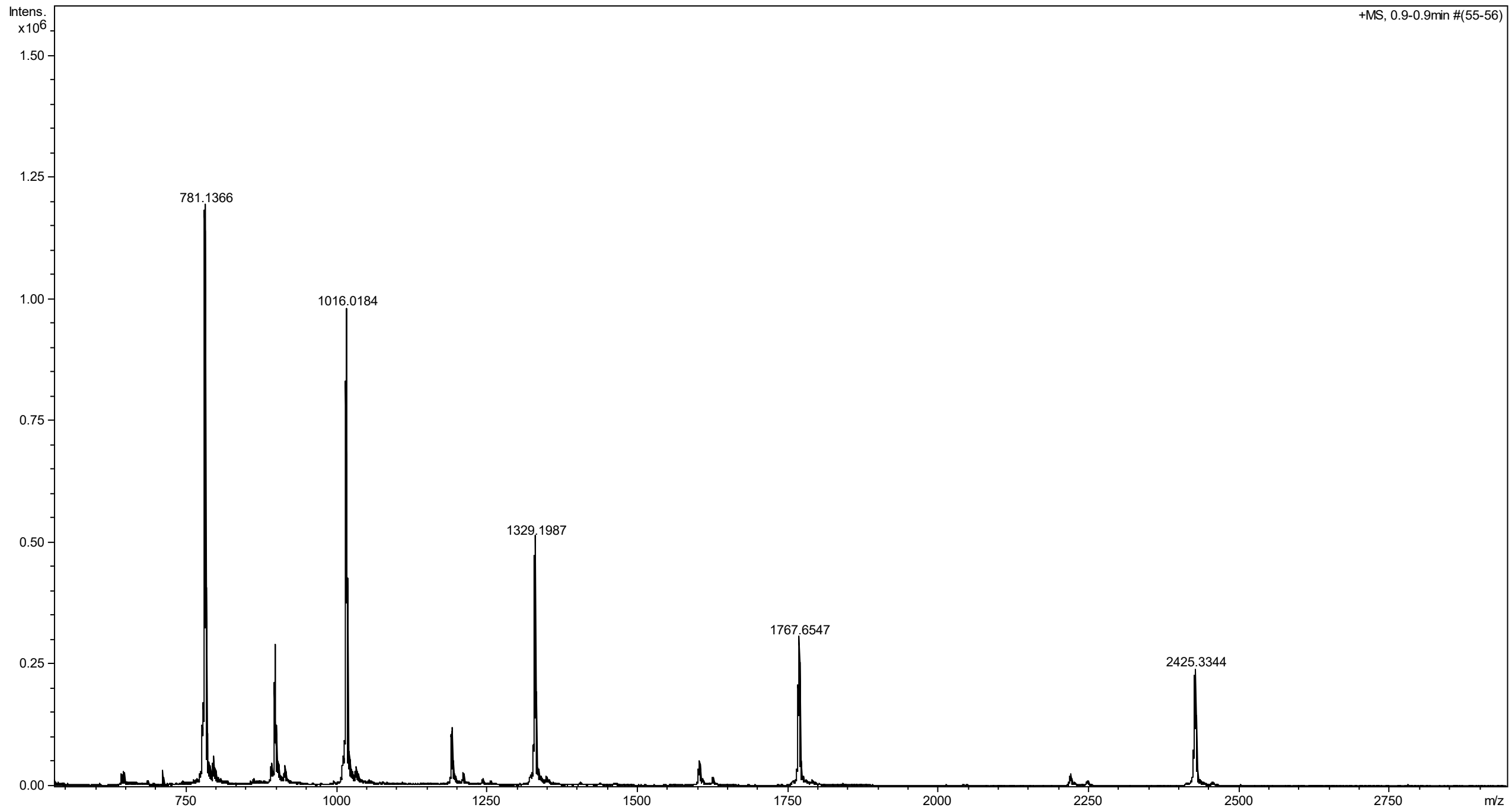

Figure S17. HR-MS spectrum of $Sc_3N@I_h$-C_{80}⊂**4·**(BArF)$_8$. Sample solved in CH_3CN and registered with a Bruker MicroTOF-Q-II mass spectrometer. Extra peaks correspond to the same species with the presence of Cl^- or Br^- as counterion.

<u>HR-MS (m/z):</u>

calculated 781.1469 and found ($Sc_3N@I_h$-C_{80}⊂[**4**·(BArF)$_0$]$^{+8}$);

calculated 1016.0346 and found ($Sc_3N@I_h$-C_{80}⊂[**4**·(BArF)$_1$]$^{+7}$);

calculated 1329.2183 and found ($Sc_3N@I_h$-C_{80}⊂[**4**·(BArF)$_2$]$^{+6}$);

calculated 1767.6754 and found ($Sc_3N@I_h$-C_{80}⊂[**4**·(BArF)$_3$]$^{+5}$);

calculated 2425.3611 and found ($Sc_3N@I_h$-C_{80}⊂[**4**·(BArF)$_4$]$^{+4}$).

NMR Characterization

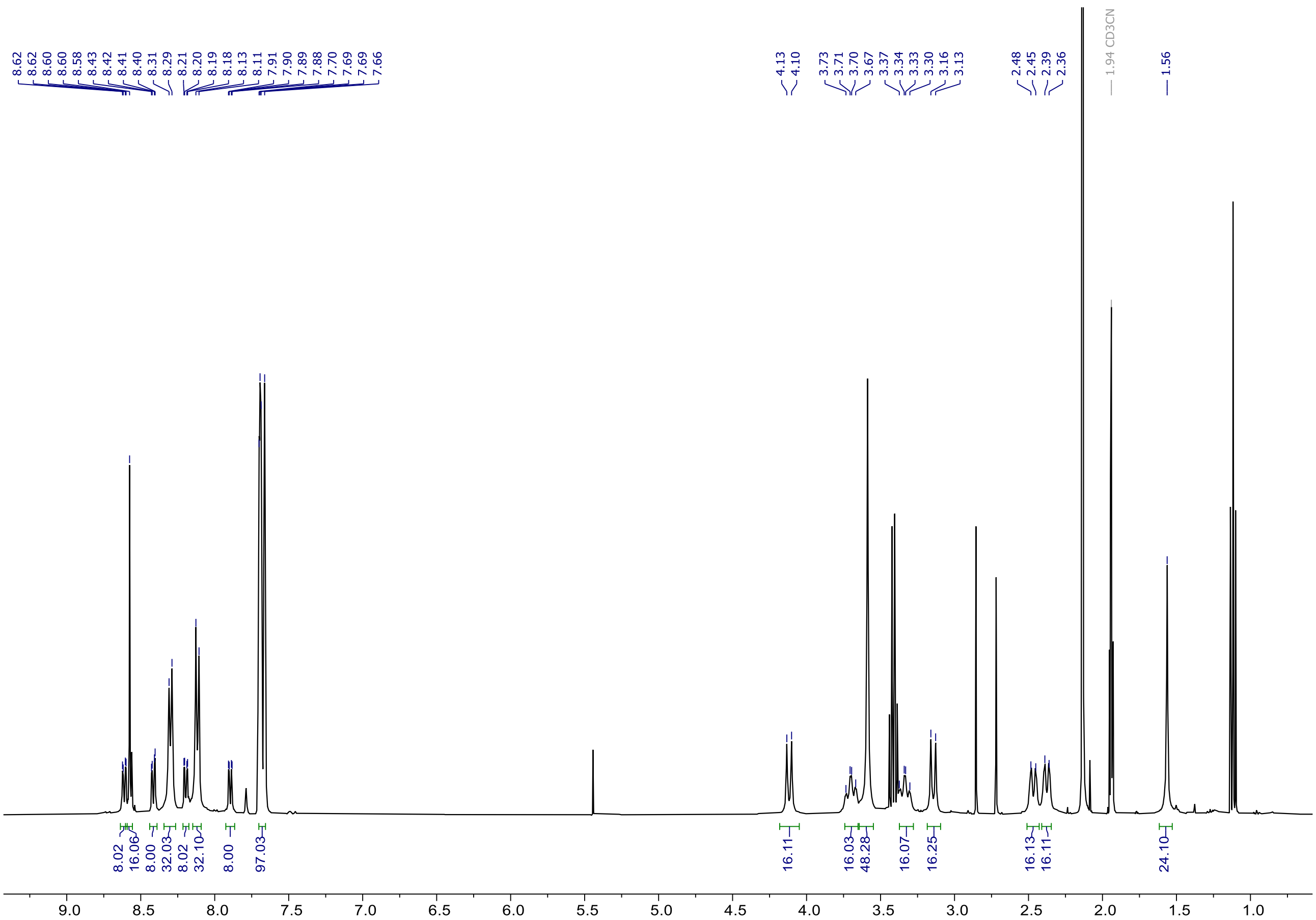

Figure S18. ^{1}H-NMR spectrum of $Sc_3N@I_h\text{-}C_{80}\subset$**4**$\cdot(BArF)_8$. Experiment performed in CD3CN at 298K (400 MHz).

^{1}H-NMR (400 MHz, CD_3CN) δ p.p.m.: 8.61 (dd, *J*=7.8, 1.8 Hz, 8H, arom-porph), 8.58 (s, 16H, pyrrole ring), 8.42 (dd, *J*=7.9, 1.8 Hz, 8H, arom-porph), 8.30 (d, *J*=7.9 Hz, 32H, arom-clip), 8.21 (dd, *J*=7.9, 1.9 Hz, 8H, arom-porph), 8.12 (d, *J*=8.4 Hz, 32H, arom-clip), 7.90 (dd, *J*=7.8, 1.8 Hz, 8H, aromporph), 7.70-7.66 (m, 96H, $BArF^-$), 4.12 (d, *J*=12.9 Hz, 16H, $-CH_2-$), 3.70 (dt, *J*=13.5,7.6 Hz, 16H, $-CH_2-$), 3.67 (s, 48H, $N-CH_3$), 3.34 (dt, *J*=13.5, 7.6 Hz, 16H, $-CH_2-$), 3.15 (d, *J*=13.0 Hz, 16H, $-CH_2-$), 2.47 (d, *J*=13.2 Hz, 16H, $-CH_2-$), 2.38 (d, *J*=11.4 Hz, 16H, $-CH_2-$) 1.56 (s, 24H, $N-CH_3$).

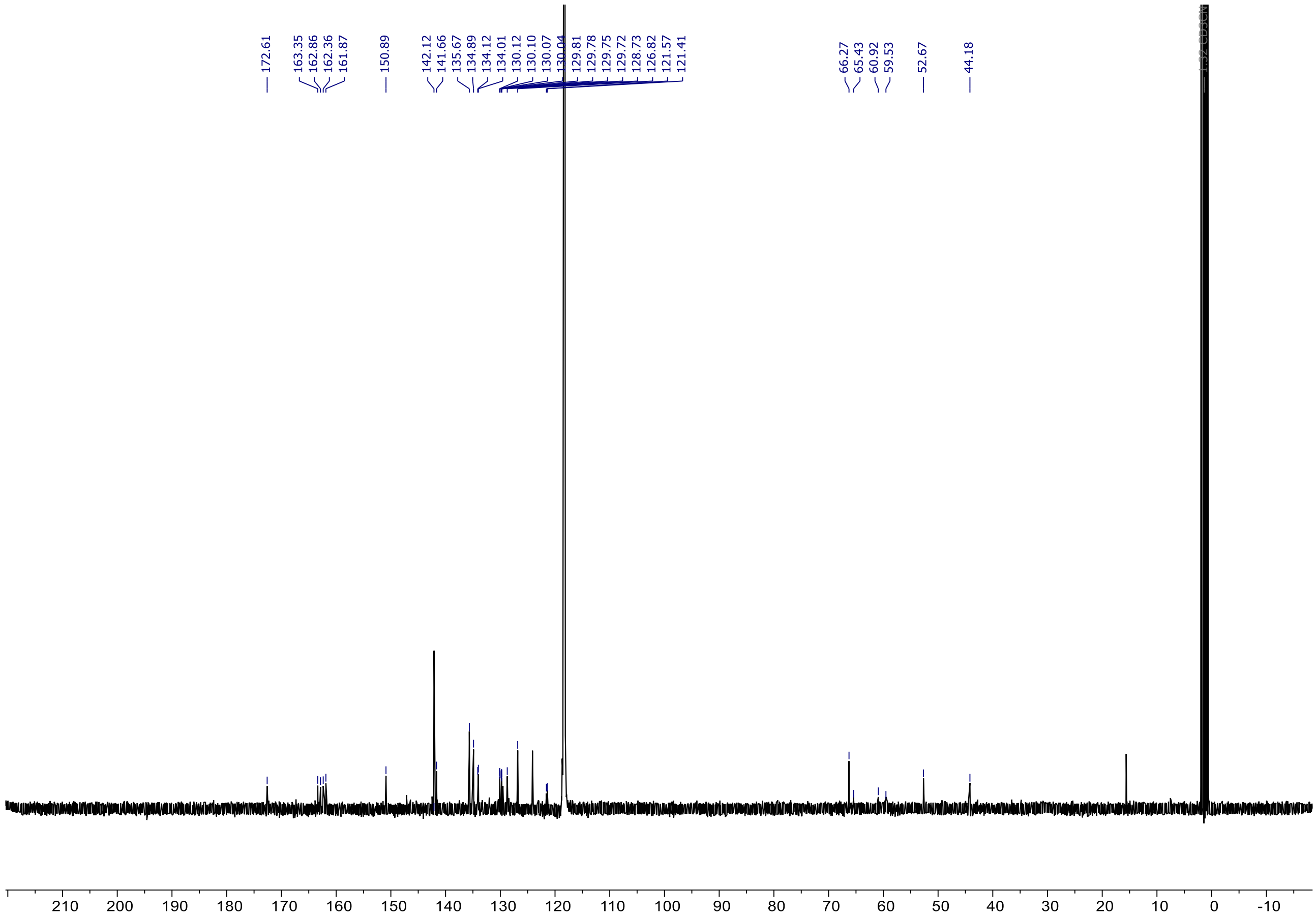

Figure S19. ^{13}C-NMR spectrum of $Sc_3N@I_h$-$C_{80}\subset$**4**·$(BArF)_8$. Experiment performed in CD3CN at 298K (100 MHz).

^{13}C-NMR (100 MHz, CD_3CN) δ p.p.m.:172.61 (COO^-), 163.35-161.87 ($BArF^-$), 150.89 (pyrrole ring) 135.62 ($BArF_-$), 142.50 (arom-porph),135.67 (arom-clip),134.10 (arom-porph), 131.94 (pyrrole), 128.68 (arom-porph, arom-clip), 128.53 (arom-porph), 65.39 ($-CH_2-$), 60.88 ($-CH_2-$), 59.48 ($-CH_2-$), 52.63 ($-CH_3$), 44.14 ($-CH_3$).

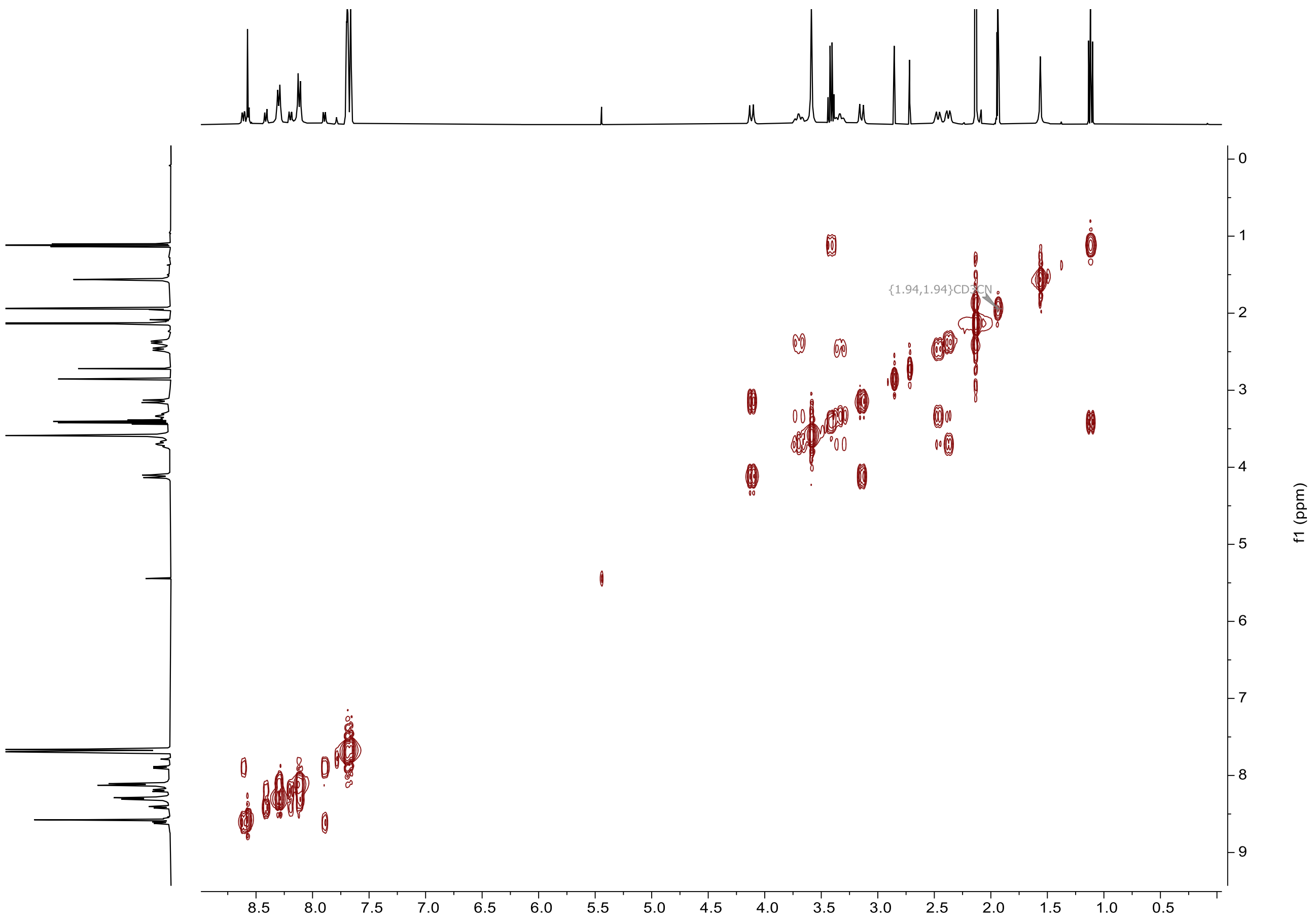

Figure S20. COSY of $Sc_3N@I_h\text{-}C_{80}\subset$**4**·$(BArF)_8$. Experiment performed in CD_3CN at 298K (400 MHz).

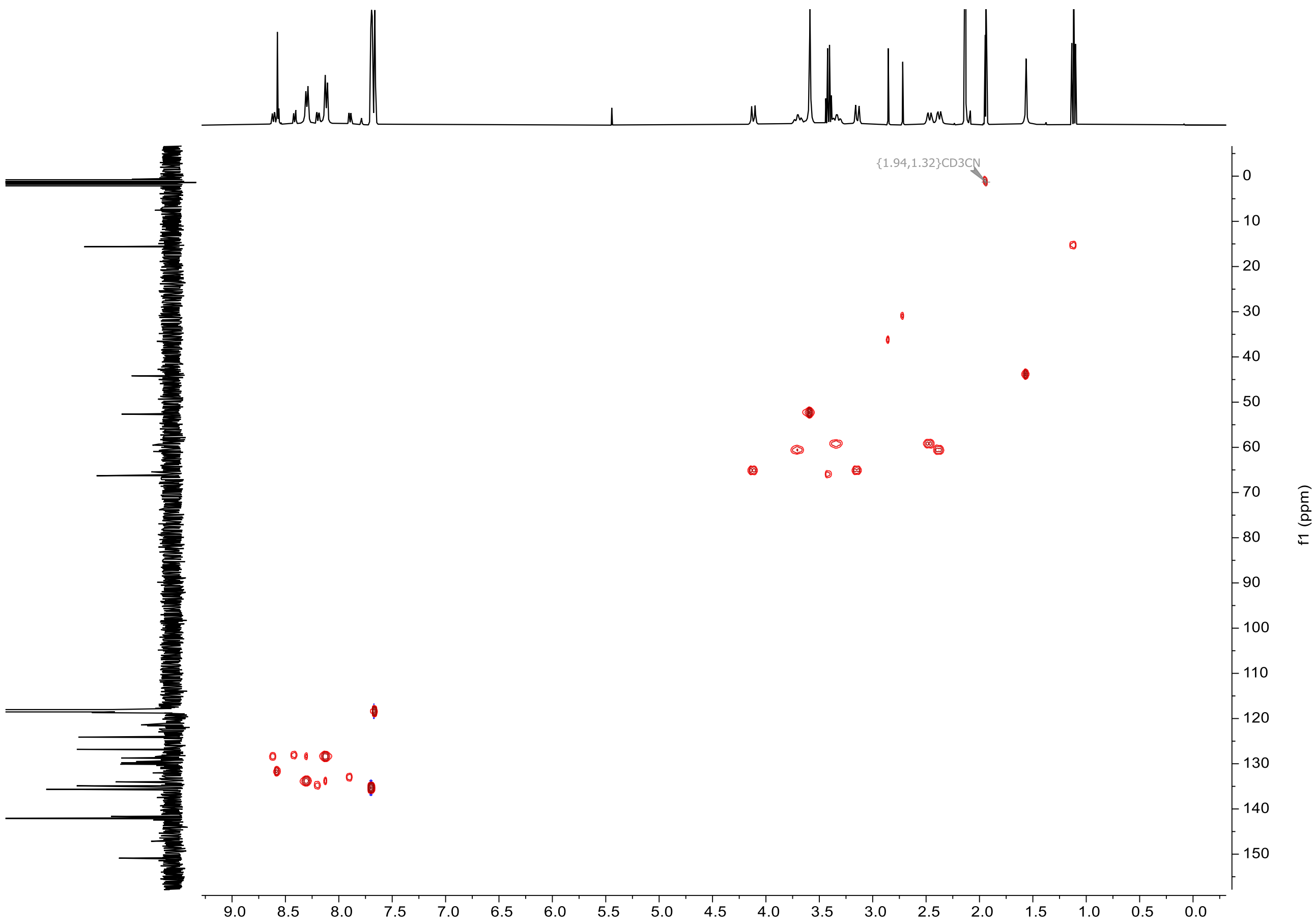

Figure S21. HSQC of $Sc_3N@I_h$-$C_{80}\subset$**4**·$(BArF)_8$. Experiment performed in CD_3CN at 298K (400 MHz).

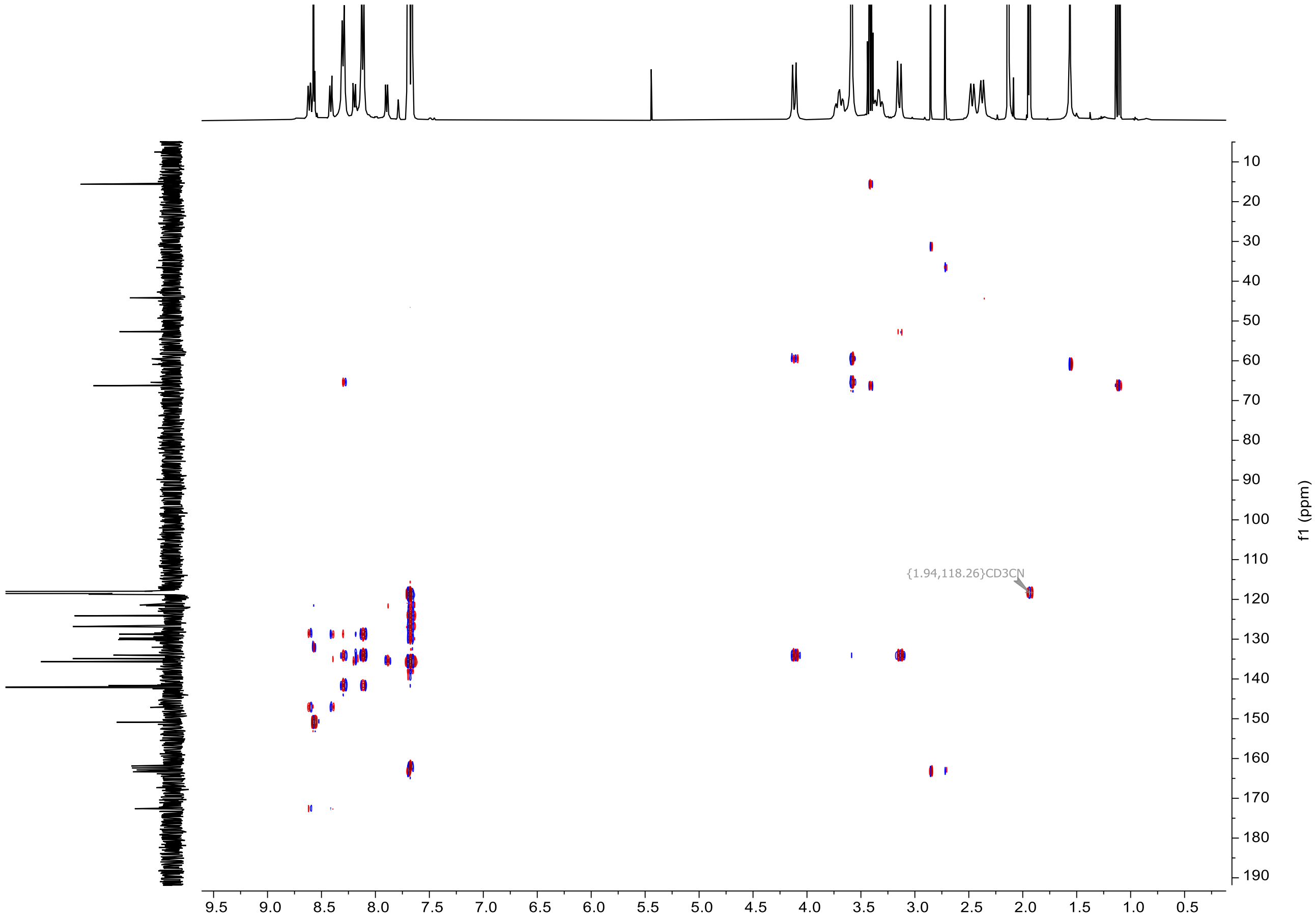

Figure S22. HMBC of $Sc_3N@I_h\text{-}C_{80}\subset$**4**·$(BArF)_8$. Experiment performed in CD3CN at 298K (400 MHz).

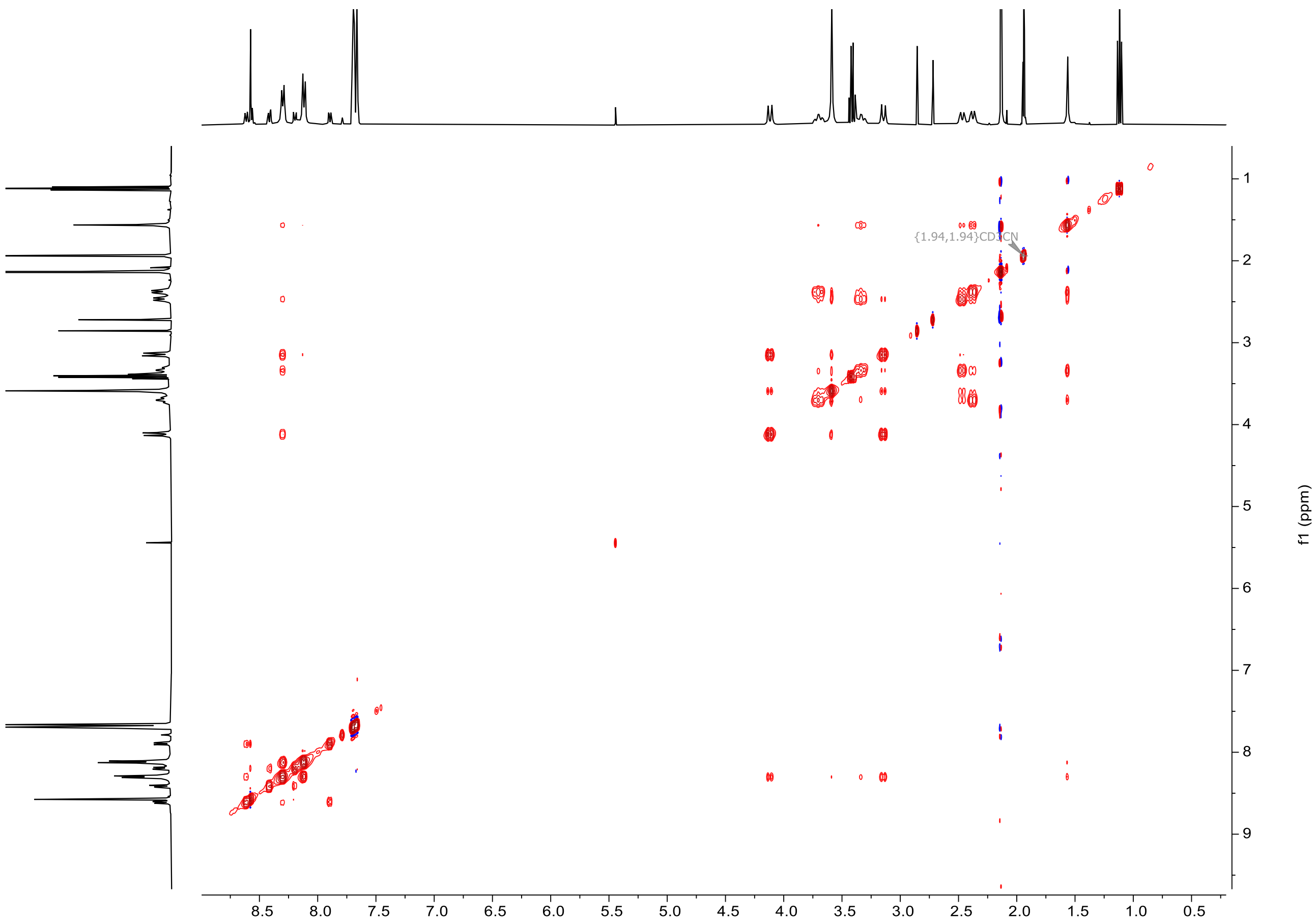

Figure S23. NOESY of $Sc_3N@I_h$-$C_{80}\subset$**4**·$(BArF)_8$. Experiment performed in CD_3CN at 298K (400 MHz).

2.2. Mask-mediated synthesis of $M_3N@I_h$-$C_{80}C(CO_2Et)_2 \subset$4·(BArF)$_8$

2.2.1. Synthesis of $Lu_3N@I_h$-$C_{80}C(CO_2Et)_2 \subset$4·(BArF)$_8$ via Bingel reaction

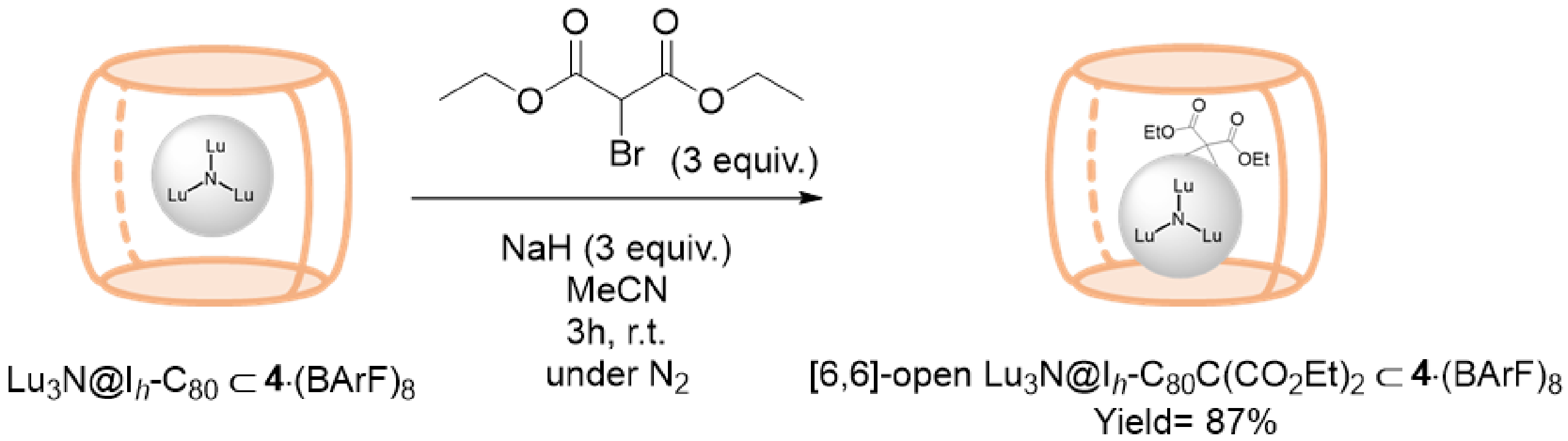

Figure S24. General procedure for Bingel Reaction on $Lu_3N@I_h$-$C_{80} \subset$**4**·(BArF)$_8$.

15 mg of $Lu_3N@I_h$-$C_{80} \subset$**4**·(BArF)$_8$ are totally dissolved in 0.5 mL of MeCN, then 3 equivalents of the diethyl bromomalonate and 3 equivalents of NaH as a base are added sequentially and the reaction is stirred under N_2 at room temperature for 3 hours. Once the $Lu_3N@I_h$-$C_{80} \subset$**4**·(BArF)$_8$ is completely consumed and the $Lu_3N@I_h$-$C_{80}C(CO_2Et)_2 \subset$**4**·(BArF)$_8$ is formed (checked by HR-MS), the reaction is stopped by filtering and removing the solvent under reduced pressure. For the work-up check section 2.2.2. for the isolation of $Lu_3N@I_h$-$C_{80}C(CO_2Et)_2 \subset$**4**·(BArF)$_8$ by crystallization, section 2.2.3.A for the release of $Lu_3N@I_h$-$C_{80}C(CO_2Et)_2$ by exchange with $Lu_3N@I_h$-C_{80} and section 2.2.3.B for the release of $Lu_3N@I_h$-$C_{80}C(CO_2Et)_2$ upon nanocapsule disassembly.

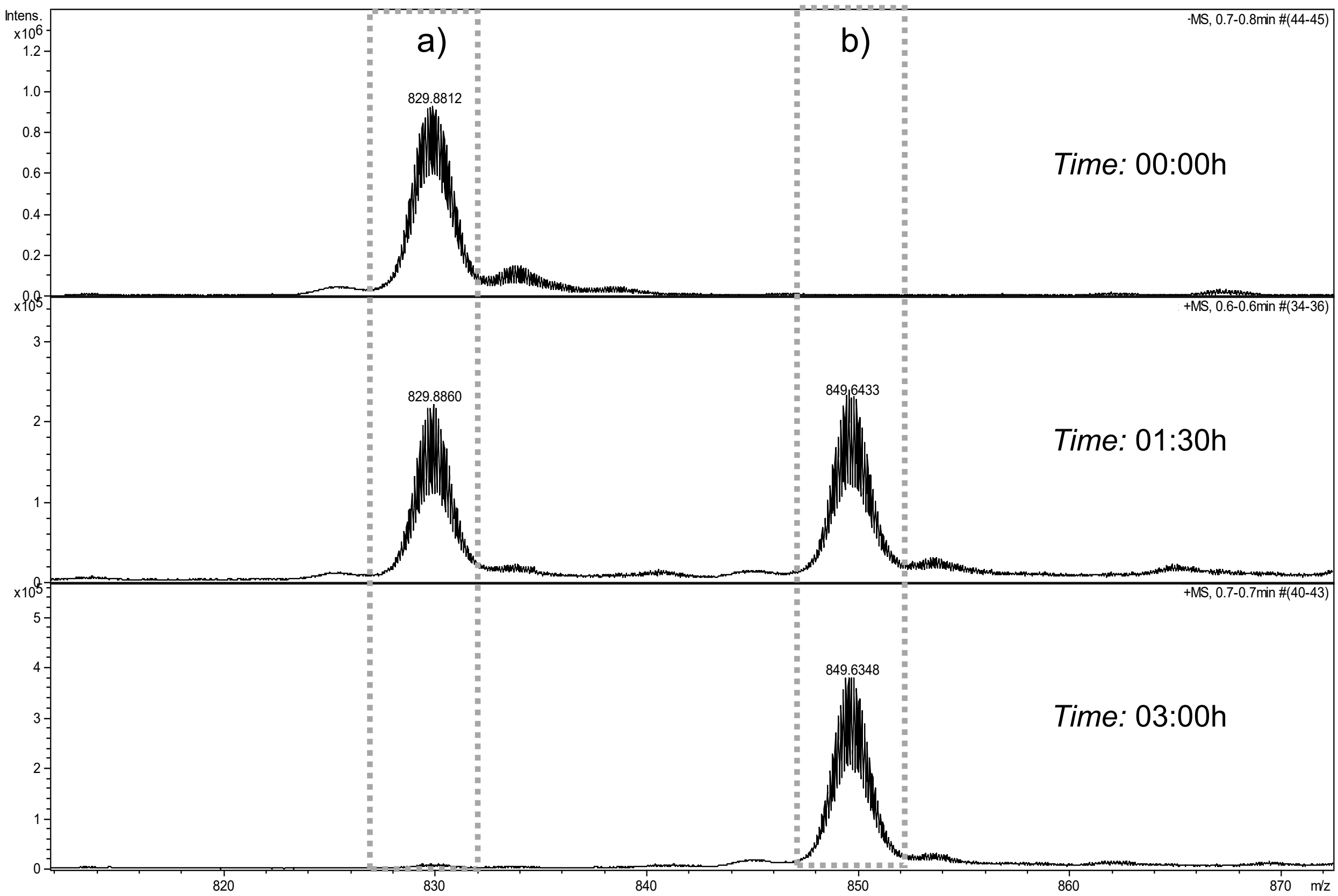

Figure S25. HR-MS monitoring of the $Lu_3N@I_h$-$C_{80}C(CO_2Et)_2$⊂**4**·(BArF)$_8$ formation upon addition of 3 equivalents of diethyl bromomalonate and 3 equivalents of NaH during 3 hours. Focus on the (+8) charge.

a) $Lu_3N@I_h$-C_{80}⊂**4**·(BArF)$_8$ peak disappears upon the additions of the reagents. ($Lu_3N@I_h$-C_{80}⊂[**4**·(BArF)$_0$]$^{+8}$) calculated 829.8912
b) $Lu_3N@I_h$-$C_{80}C(CO_2Et)_2$⊂**4**·(BArF)$_8$ forming during the 3 hours of the reaction. ($Lu_3N@I_h$-$C_{80}C(CO_2Et)_2$⊂[**4**·(BArF)$_0$]$^{+8}$) calculated 849.6485

Sample solved in CH_3CN and registered with a Bruker MicroTOF-Q-II mass spectrometer.

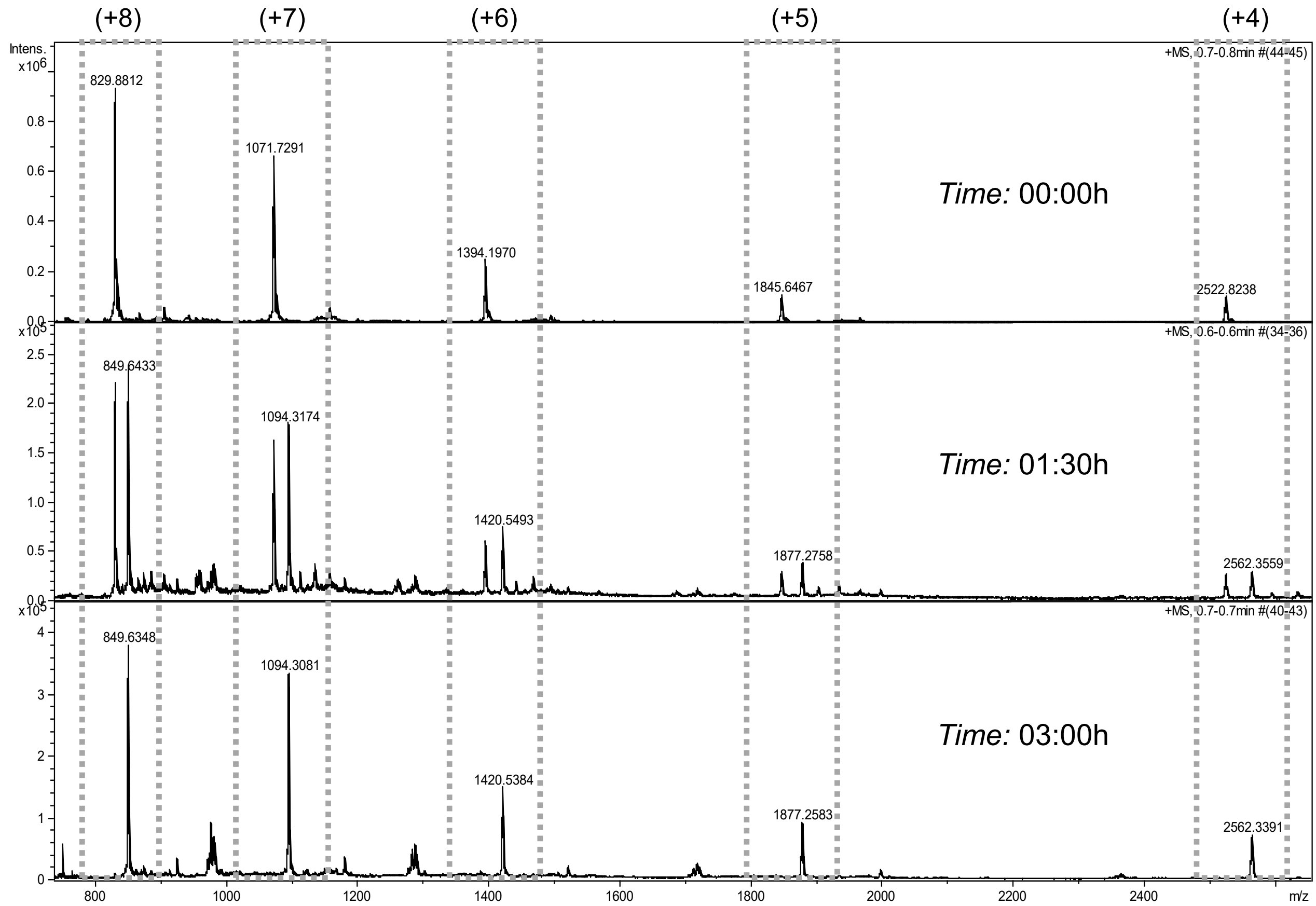

Figure S26. HR-MS monitoring of the $Lu_3N@I_h\text{-}C_{80}C(CO_2Et)_2\subset\mathbf{4}\cdot(BArF)_8$ formation upon addition of 3 equivalents of diethyl bromomalonate and 3 equivalents of NaH for 3 hours. All the charges displayed from (+8) up to (+4) correspond to the presence of $nBArF^-$ (n= 0-4) as counterion. Extra peaks correspond to the same species with the presence of Cl^- or Br^- as counterion.

Sample solved in CH_3CN and registered with a Bruker MicroTOF-Q-II mass spectrometer.

2.2.2. Characterization of $Lu_3N@I_h\text{-}C_{80}C(CO_2Et)_2{\subset}4{\cdot}(BArF)_8$

Crystallization of $Lu_3N@I_h\text{-}C_{80}C(CO_2Et)_2{\subset}4{\cdot}(BArF)_8$

The solid obtained after the Bingel reaction (15 mg) is dissolved in 4 mL of DCM and 200 µL of CH_3CN and then crystallized by slow diethyl ether diffusion (24 mL) in 4 days. The crystals are dried in the vacuum line (Yield: 87%).

For details on the X-Ray diffraction see section 4.

Characterization of $Lu_3N@I_h\text{-}C_{80}C(CO_2Et)_2{\subset}4{\cdot}(BArF)_8$

The formation of the host guest complex with the $Lu_3N@I_h\text{-}C_{80}C(CO_2Et)_2$ adduct inside the nanocapsule **4**·(BArF)$_8$ is characterized by HR-MS and NMR techniques.

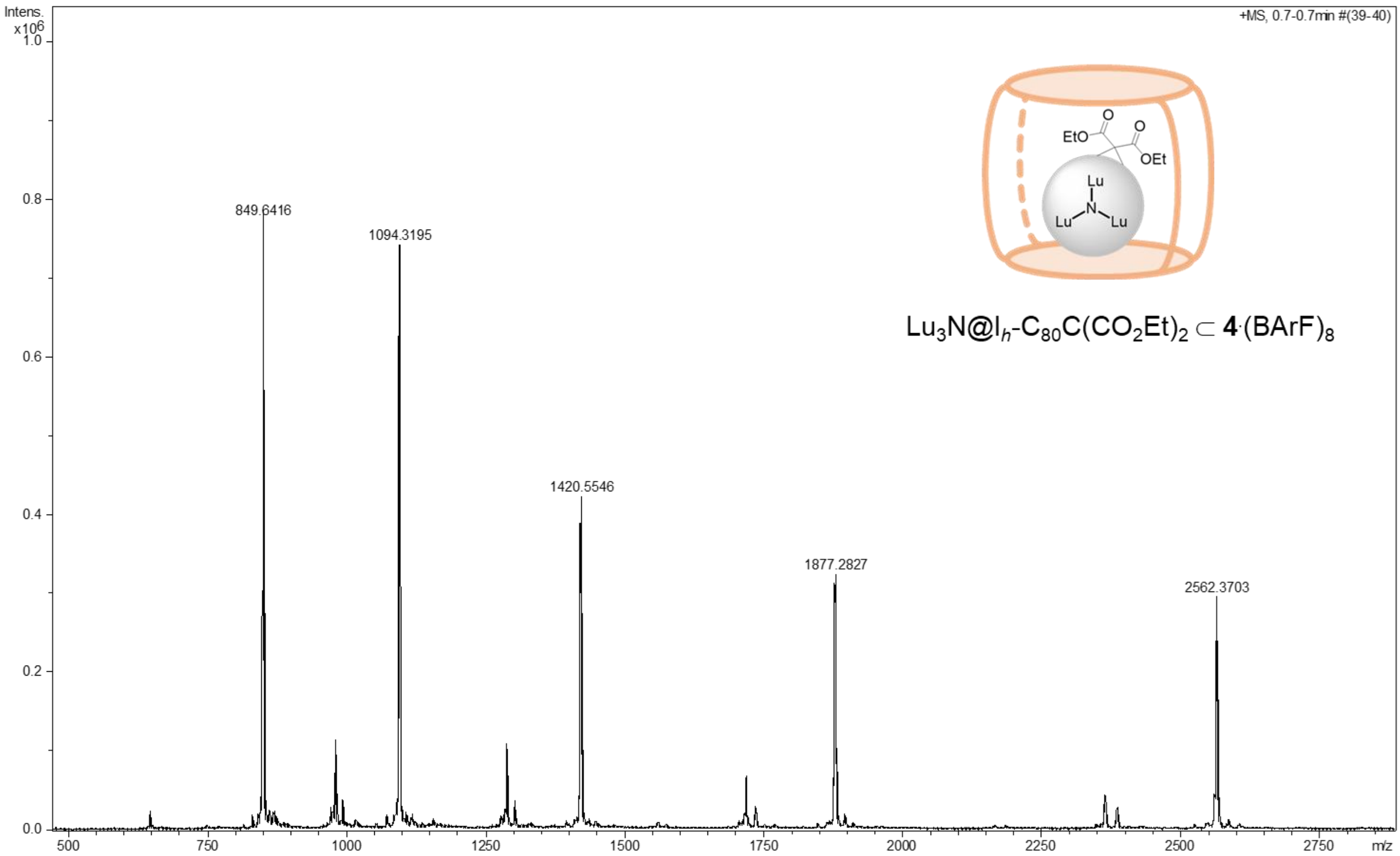

Figure S27. HR-MS spectrum of $Lu_3N@I_h\text{-}C_{80}C(CO_2Et)_2{\subset}$**4·**(BArF)$_8$. Sample solved in CH_3CN and registered with a Bruker MicroTOF-Q-II mass spectrometer. All the charges displayed from (+8) up to (+4) correspond to the presence of nBArF- (n= 0-4) as counterion. Extra peaks correspond to the same species with the presence of Cl- or Br- as counterion.

HR-MS (m/z):

calculated 849.6485 and found 849.6416 ($Lu_3N@I_h\text{-}C_{80}C(CO_2Et)_2{\subset}[$**4**$\cdot(BArF)_0]^{+8}$);

calculated 1094.3222 and found 1094.3195 ($Lu_3N@I_h\text{-}C_{80}C(CO_2Et)_2{\subset}[$**4**$\cdot(BArF)_1]^{+7}$;

calculated 1420.5538 and found 1420.5546 ($Lu_3N@I_h\text{-}C_{80}C(CO_2Et)_2{\subset}[$**4**$\cdot(BArF)_2]^{+6}$;

calculated 1877.2780 and found 1877.2827 ($Lu_3N@I_h\text{-}C_{80}C(CO_2Et)_2{\subset}[$**4**$\cdot(BArF)_3]^{+5}$;

calculated 2562.3643 and found 2562.3703 ($Lu_3N@I_h\text{-}C_{80}C(CO_2Et)_2{\subset}[$**4**$\cdot(BArF)_4]^{+4}$.

NMR Characterization

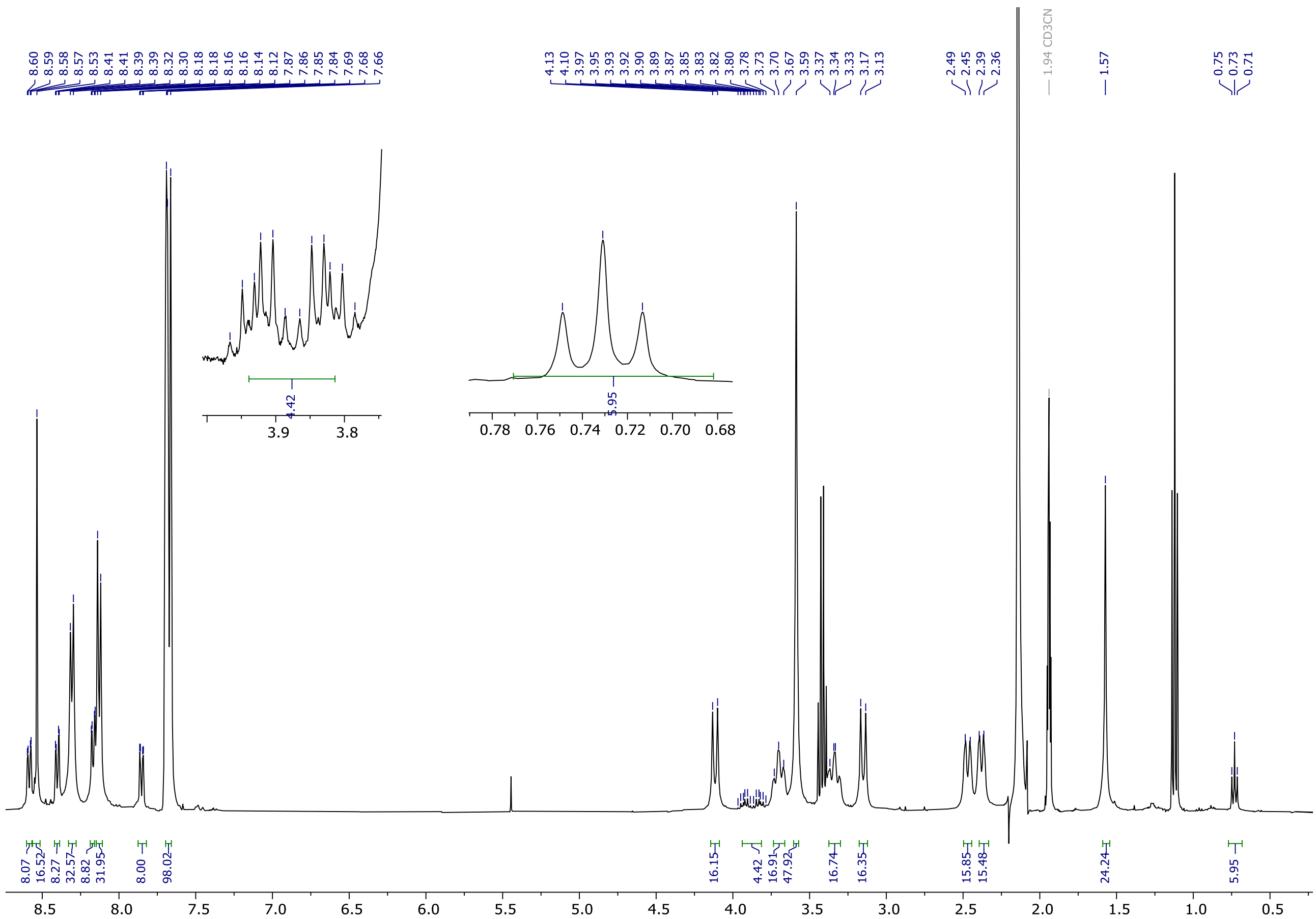

Figure S28. ^{1}H-NMR of $Lu_3N@I_h$-$C_{80}C(CO_2Et)_2$⊂**4**·$(BArF)_8$ (400 MHz, CD_3CN).

<u>^{1}H-NMR</u> (400 MHz, CD_3CN) δ p.p.m.: 8.58 (dd, *J*=7.8, 1.9 Hz, 8H, arom-porph), 8.53 (s, 16H, pyrrole ring), 8.40 (dd, *J*=7.9, 1.8 Hz, 8H, arom-porph), 8.31 (d, *J*=7.9 Hz, 32H, arom-clip), 8.17 (dd, *J*=8.1, 1.8 Hz, 8H, arom-porph), 8.13 (d, *J*=8.1 Hz, 32H, arom-clip), 7.86 (dd, *J*=7.8, 1.9 Hz, 8H, arom-porph), 7.69-7.66 (m, 96H, $BArF^-$), 4.12 (d, *J*=12.9 Hz, 16H), 3.97 – 3.78 (m, 4H), 3.70 (t, *J*= 13.2 Hz, 16H), 3.59 (s, 48H), 3.34 (t, *J*=13.2 Hz, 16H), 3.15 (d, *J*=13.0 Hz, 16H), 2.42 (dd, *J*=36.0, 12.6 Hz, 32H), 1.57 (s, 24H), 0.73 (t, *J*=7.1 Hz, 6H).

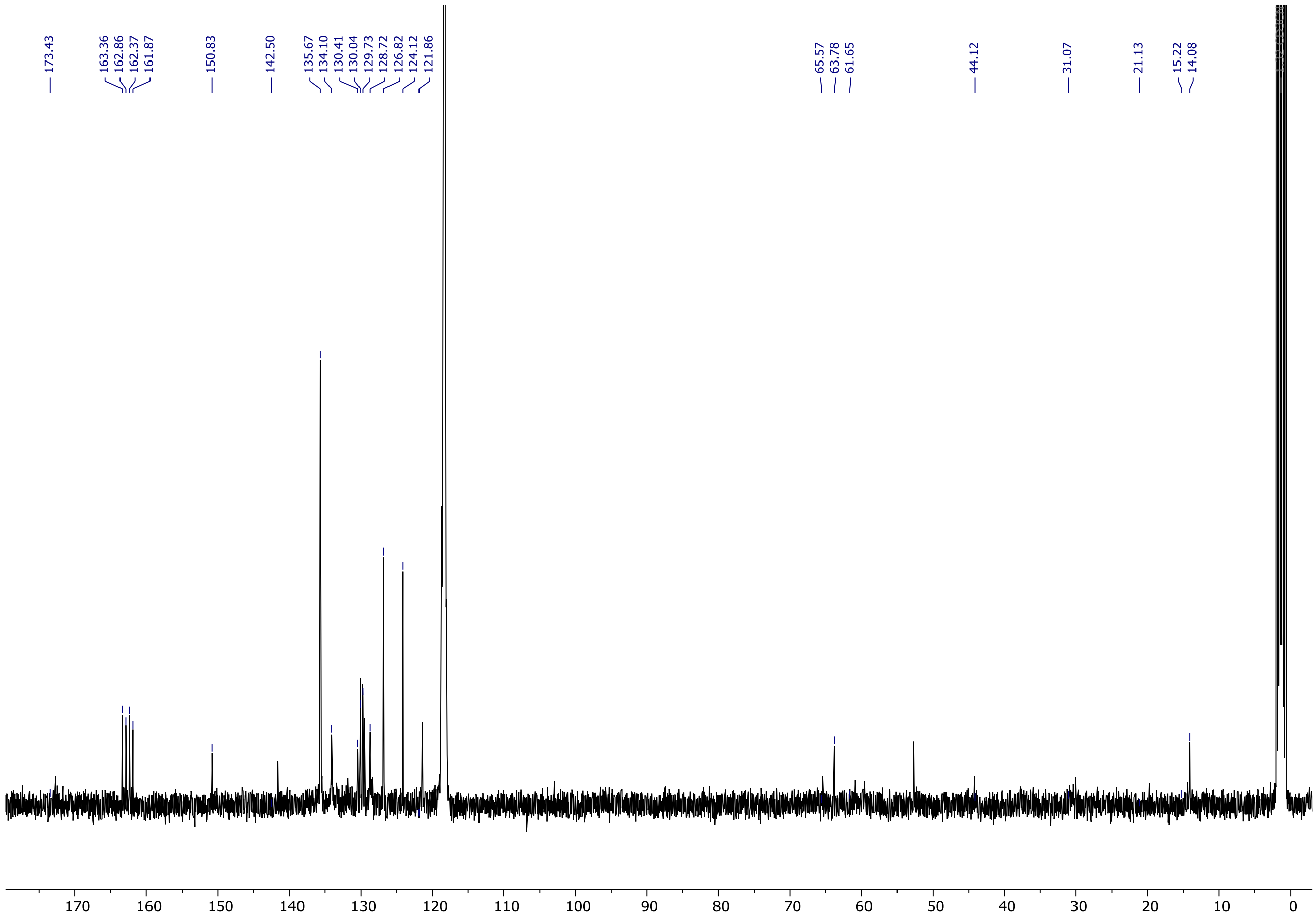

Figure S29. ^{13}C-NMR of $Lu_3N@I_h\text{-}C_{80}C(CO_2Et)_2\subset$**4**$\cdot(BArF)_8$ (100 MHz, CD_3CN).

^{13}C-NMR (100 MHz, CD_3CN) δ p.p.m.: 173.43 (COO^-), 163.36-161.87 ($BArF^-$), 150.83 (pyrrole ring), 142.50 (arom-clip), 135.67 ($BArF^-$), 134.10 (arom-clip), 128.72 (arom-porh), 126.82 (arom-clip), 65.57 ($-CH_2-$ functionalization), 63.78 ($-CH_2-$), 61.65 ($-CH_2-$), 52.38 ($-CH_3$), 44.12 ($-CH_3$), 31.07 ($-CH_3$ functionalization).

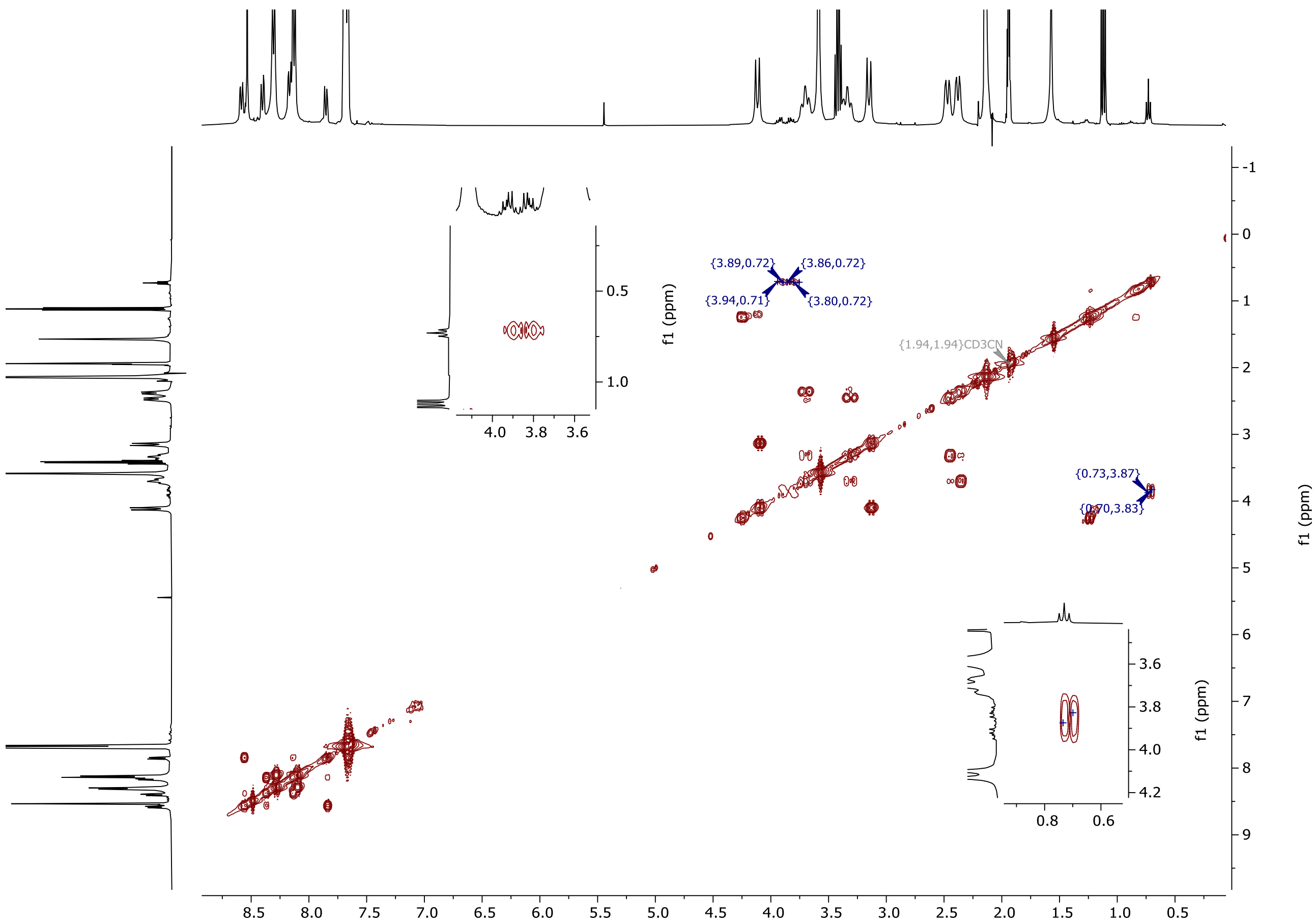

Figure S30. COSY NMR of $Lu_3N@I_h\text{-}C_{80}C(CO_2Et)_2 \subset$**4**$\cdot(BArF)_8$. Experiment performed in CD_3CN at 298 K (400 MHz).

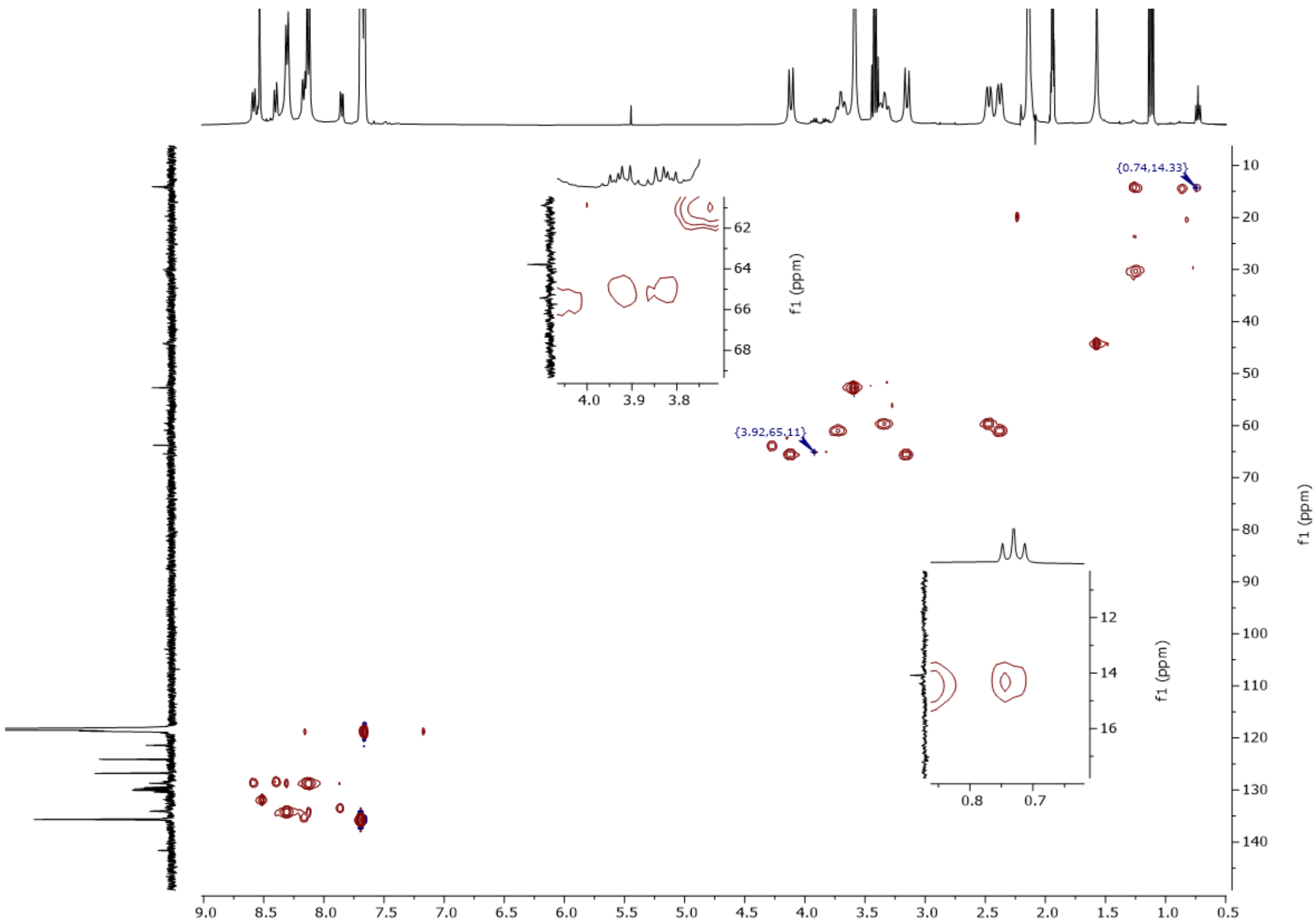

Figure S31. HSQC NMR of $Lu_3N@I_h\text{-}C_{80}C(CO_2Et)_2\subset$**4**$\cdot(BArF)_8$. Experiment performed in CD_3CN at 298 K (400 MHz).

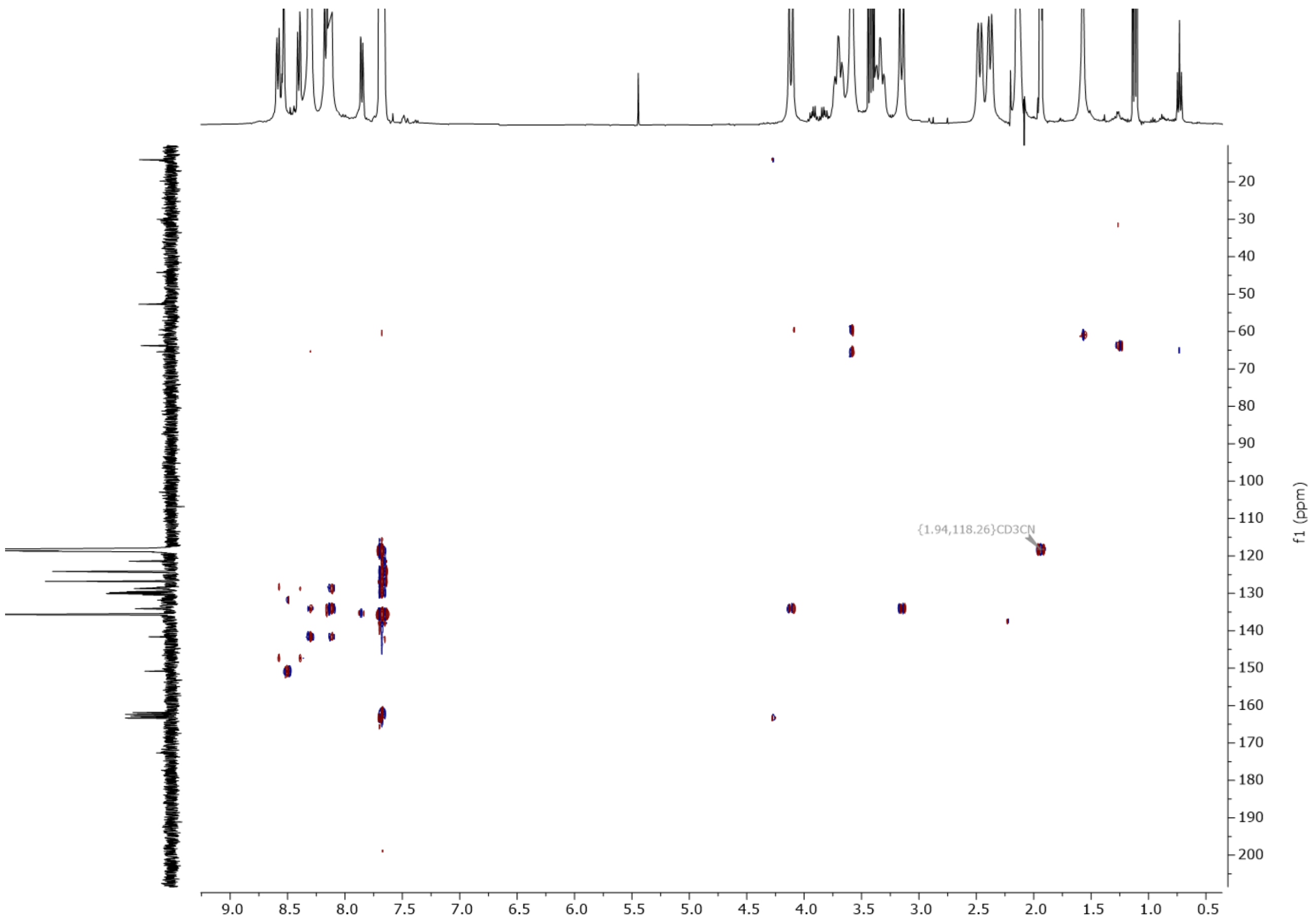

Figure S32. HMBC NMR of $Lu_3N@I_h$-$C_{80}C(CO_2Et)_2 \subset$**4**$\cdot(BArF)_8$. Experiment performed in CD_3CN at 298 K (400 MHz).

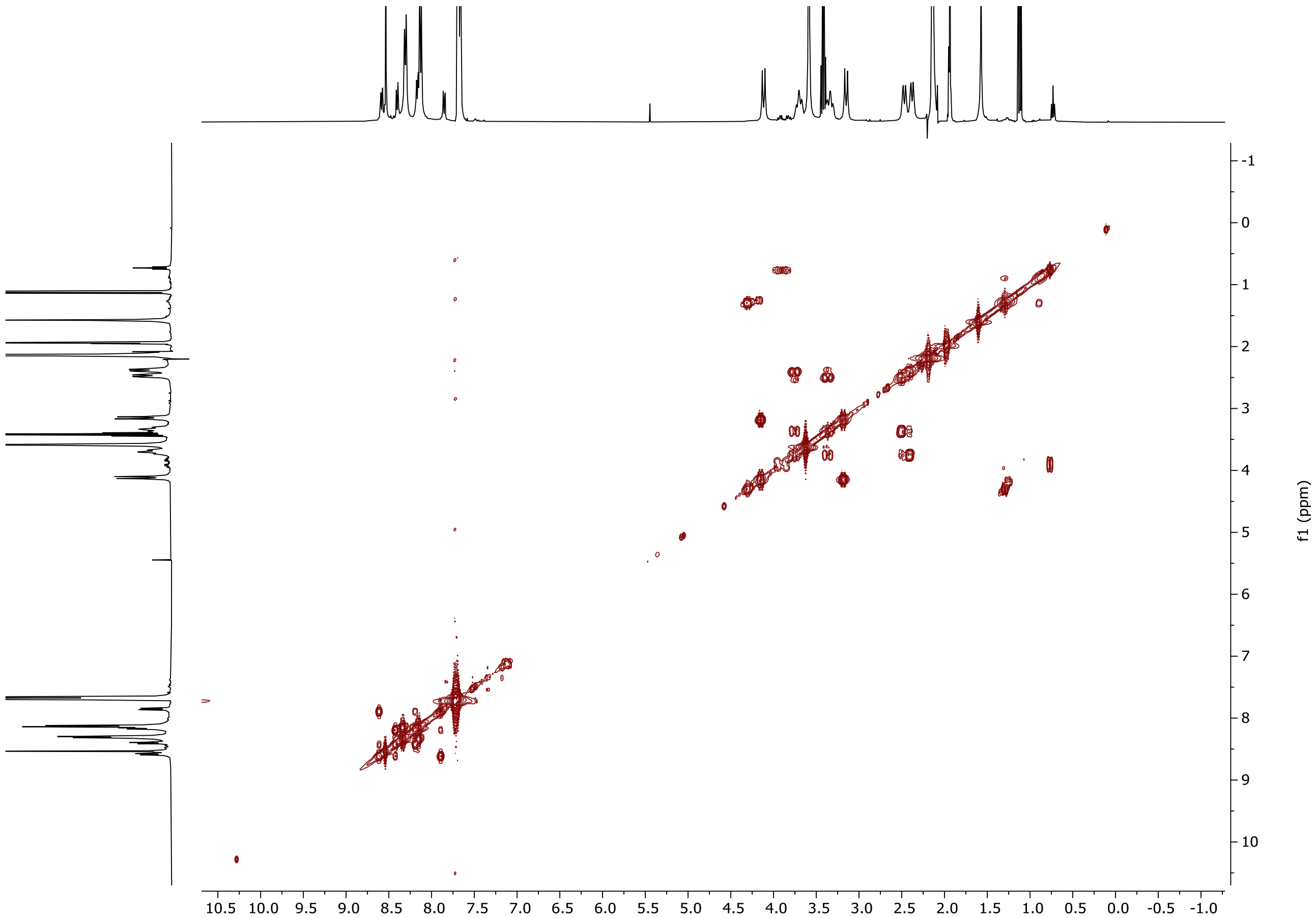

Figure S33. NOESY of $Lu_3N@I_h\text{-}C_{80}C(CO_2Et)_2\subset$**4**$\cdot(BArF)_8$. Experiment performed in CD_3CN at 298 K (400 MHz).

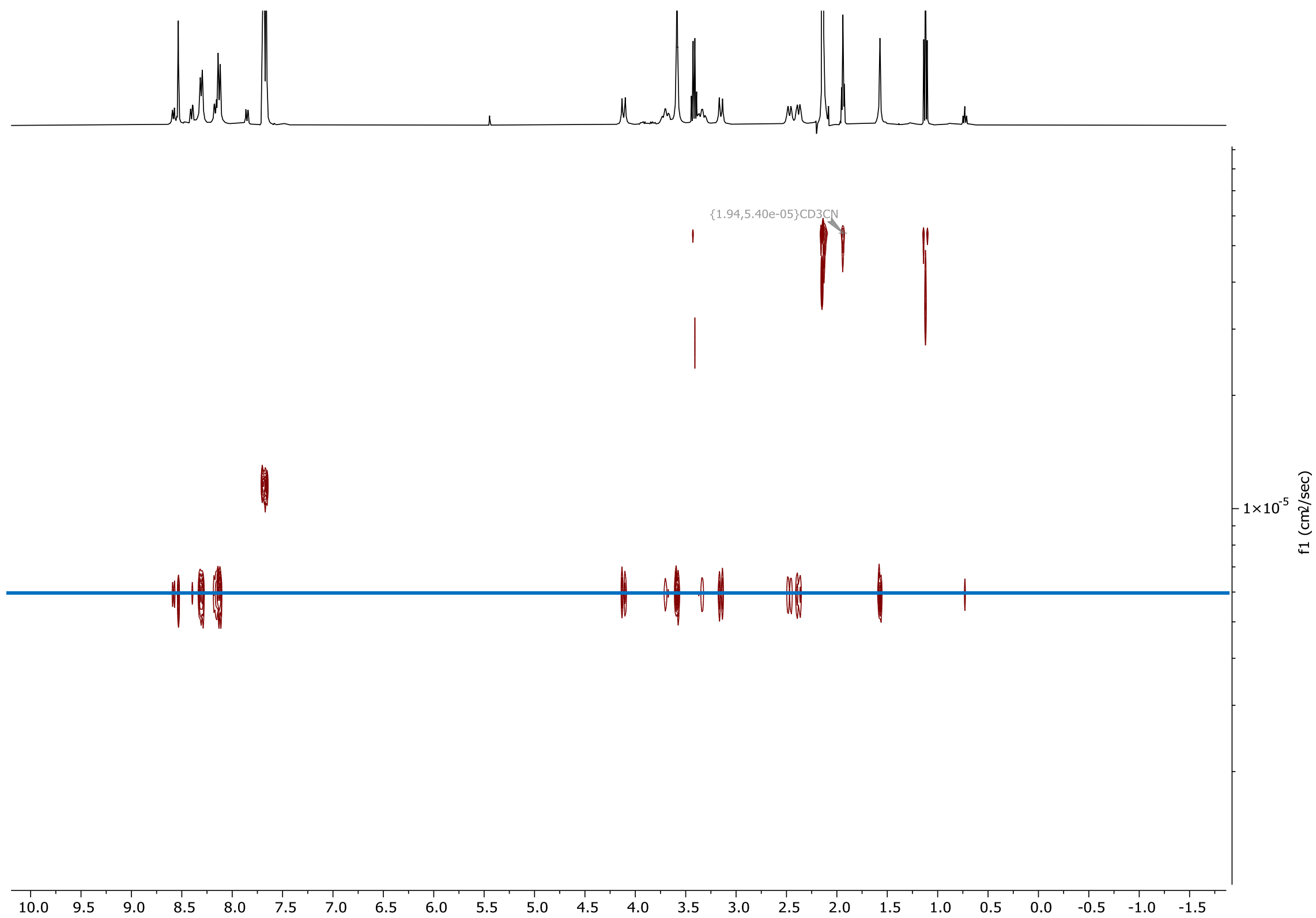

Figure S34. DOSY NMR of $Lu_3N@I_h$-$C_{80}C(CO_2Et)_2$⊂**4**·$(BArF)_8$ (400 MHz, CD_3CN). D =5.9×10^{-6} cm^2/s.

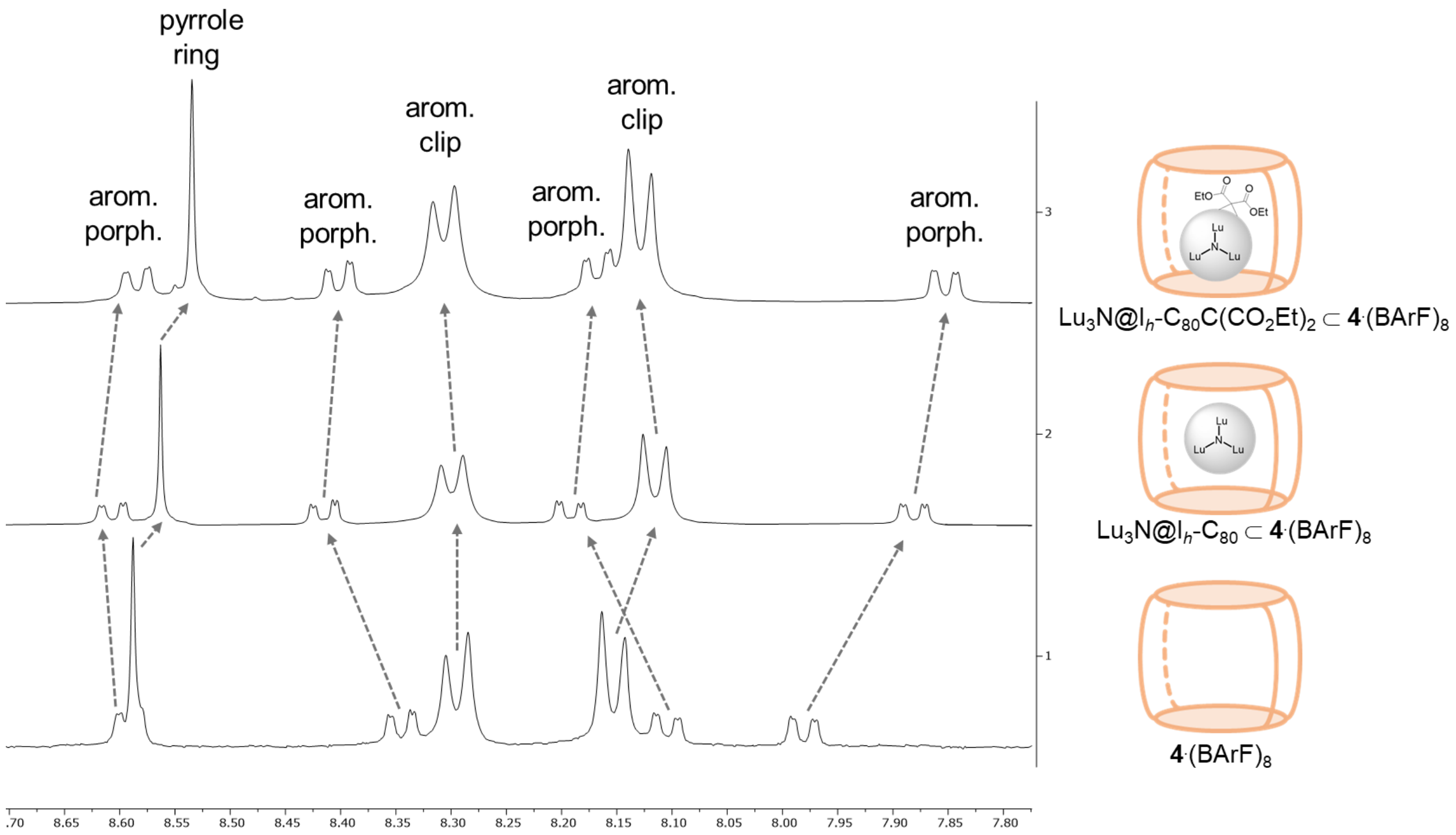

Figure S35. Comparative ^{1}H-NMR spectra (CD_3CN, 400 MHz, 298 K) of $Lu_3N@I_h$-$C_{80}C(CO_2Et)_2$⊂**4**·$(BArF)_8$ (up), $Lu_3N@I_h$-C_{80}⊂**4**·$(BArF)_8$ (middle) and **4**·$(BArF)_8$ (down). Changes in the chemical shift upon encapsulation, focus on the aromatic region of the spectra.

2.2.3. Release and characterization of $Lu_3N@I_h$-$C_{80}C(CO_2Et)_2$

To obtain the release of $Lu_3N@I_h$-$C_{80}C(CO_2Et)_2$ two different strategies have been used, one based on the exchange of the guest for unfunctionalized $Lu_3N@I_h$-C_{80} to obtain reusable host-guest complex (A) and one based on the disassembly of the nanocapsule (B) which is faster and less resources consuming.

A) <u>Exchange of $Lu_3N@I_h$-$C_{80}C(CO_2Et)_2$ for $Lu_3N@I_h$-C_{80} to obtain reusable Host-guest complex</u>

The crude $Lu_3N@I_h$-$C_{80}C(CO_2Et)_2\subset$**4**·$(BArF)_8$ reaction mixture (15 mg) is dissolved in CH_3CN (1 mL) and $Lu_3N@I_h$-C_{80} (4.0 - 20 equivalents) is added as a suspension in Toluene (1mL) (CH_3CN/Toluene 1:1) and stirred at 40°C for 7 days (monitoring by HR-MS). Then, the solution is dried with N_2 gas flow and the $Lu_3N@I_h$-$C_{80}C(CO_2Et)_2$ is obtained in the liquid fraction after $CHCl_3$ addition and filtration. The solid $Lu_3N@I_h$-$C_{80}\subset$**4**·$(BArF)_8$ complex is recovered adding CH_3CN (3x1 mL).

To separate the desired product from the excess of $Lu_3N@I_h$-C_{80} it was performed a preparative TLC (SiO_2) in Toluene 100%.

$Lu_3N@I_h$-$C_{80}C(CO_2Et)_2$ has been characterized by ^{1}H-NMR, HPLC and UV-Vis to confirm the identity, which was also cross-matched with an independently synthesized sample of the product and the data present in literature.[22]

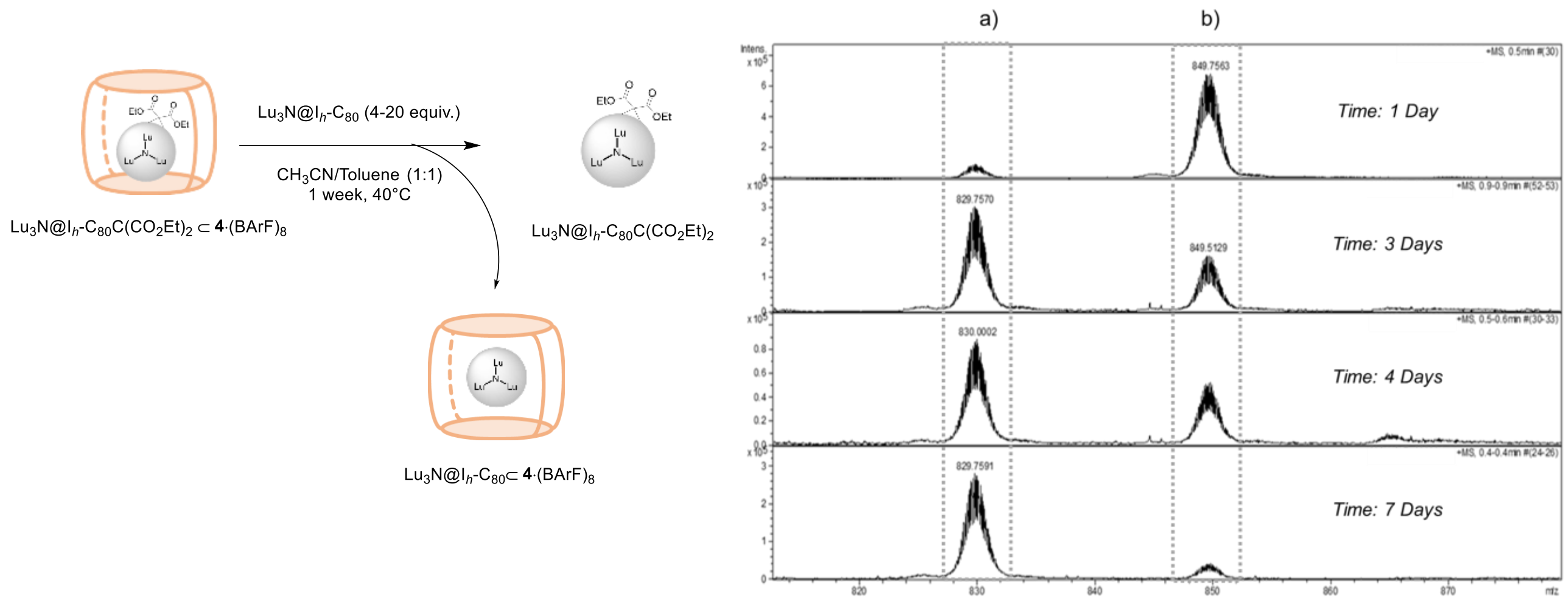

Figure S36. General procedure for the release of $Lu_3N@I_h$-$C_{80}C(CO_2Et)_2$ by exchange with $Lu_3N@I_h$-C_{80}.

Left: Reaction scheme of the exchange process of $Lu_3N@I_h$-$C_{80}C(CO_2Et)_2$ for $Lu_3N@I_h$-C_{80} to obtain reusable $Lu_3N@I_h$-$C_{80}\subset$**4**·$(BArF)_8$.

Right: HR-MS monitoring. Changes in the peaks of (a) $Lu_3N@I_h$-$C_{80}\subset$**4**·$(BArF)_8$ and (b) $Lu_3N@I_h$-$C_{80}C(CO_2Et)_2\subset$**4**·$(BArF)_8$.

Sample solved in CH_3CN and registered with a Bruker MicroTOF-Q-II mass spectrometer.

B) Disassembly of the nanocapsule to obtain $Lu_3N@I_h$-$C_{80}C(CO_2Et)_2$.

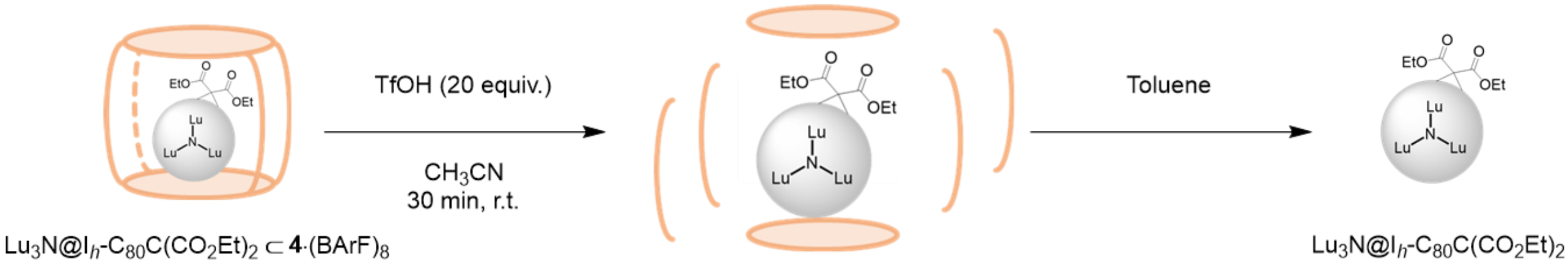

Figure S37. General procedure for the release of $Lu_3N@I_h$-$C_{80}C(CO_2Et)_2$ by **4**·$(BArF)_8$ disassembly.

The crude $Lu_3N@I_h$-$C_{80}C(CO_2Et)_2$⊂**4**·$(BArF)_8$ reaction mixture (15 mg) is dissolved in CH_3CN (1.5 mL) and an excess of TfOH (20 equivalents) is added to afford the disassembly of the nanocapsule. The mixture is stirred at room temperature during 30 min, then, the solution is dried with N_2 gas flow and $Lu_3N@I_h$-$C_{80}C(CO_2Et)_2$ is obtained after Toluene addition and filtration of the solid to remove the broken nanocapsule. Further purification of the $Lu_3N@I_h$-$C_{80}C(CO_2Et)_2$ can be obtained by preparative TLC (SiO_2) in Toluene 100%. $Lu_3N@I_h$-$C_{80}C(CO_2Et)_2$ has been characterized by ^{1}H-NMR, HPLC and UV-Vis to confirm the identity.

2.2.4. Characterization of $Lu_3N@I_h$-$C_{80}C(CO_2Et)_2$

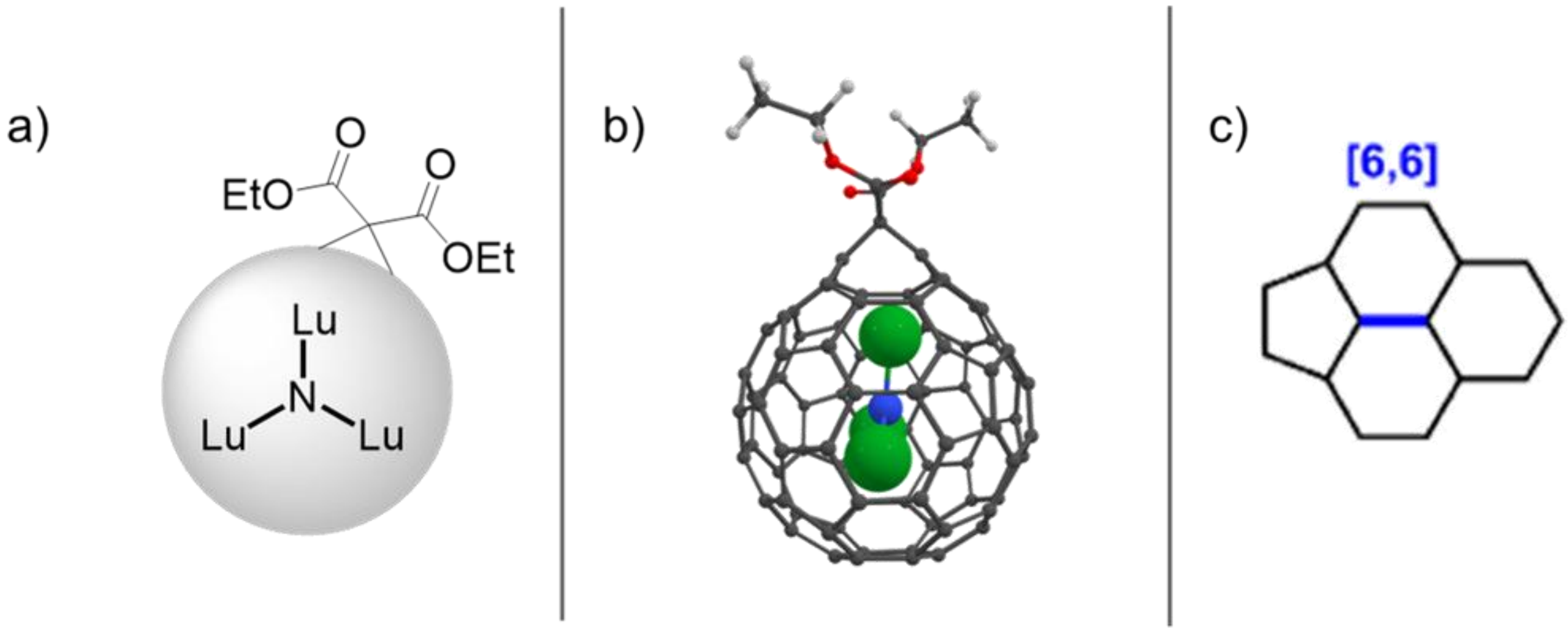

Figure S38. $Lu_3N@I_h$-$C_{80}C(CO_2Et)_2$

a) Representation of $Lu_3N@I_h$-$C_{80}C(CO_2Et)_2$ used in the manuscript and the SI.

b) $Lu_3N@I_h$-$C_{80}C(CO_2Et)_2$, metallofulleroid with [6,6]-open structure with ordered Lu_3N cluster.

O atoms in red, N atom in blue, Lu atoms in green (DFT-optimized structure)

c) [6,6] bonds functionalized upon Bingel reaction.

<u>LDI Mass Spectrum</u>

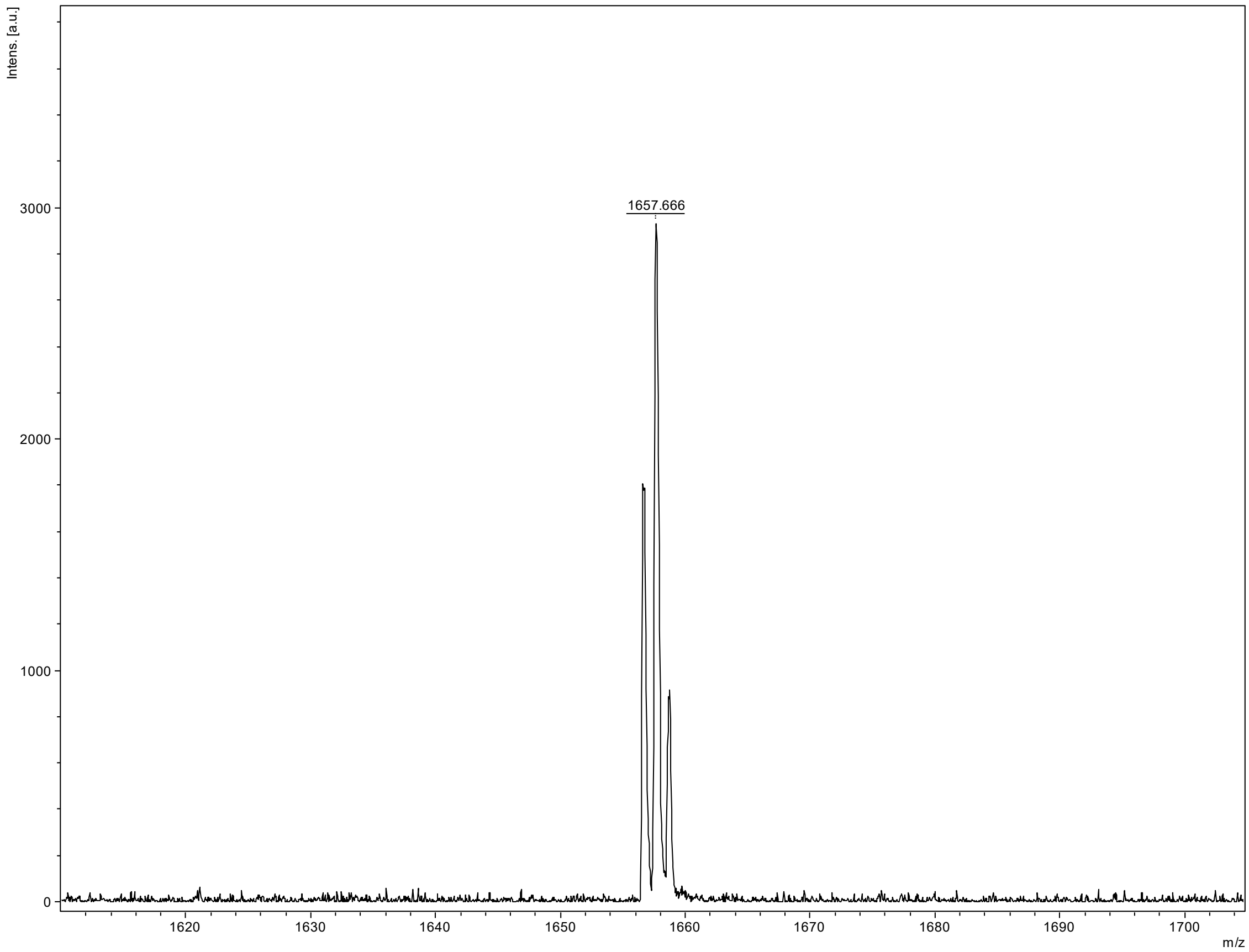

Figure S39. LDI-MS Spectrum of $Lu_3N@I_h\text{-}C_{80}C(CO_2Et)_2$.

Calculated 1657.788 and found 1657.666. Positive mode, using DCTB/NaTFA as matrix.

UV-VIS Spectrum

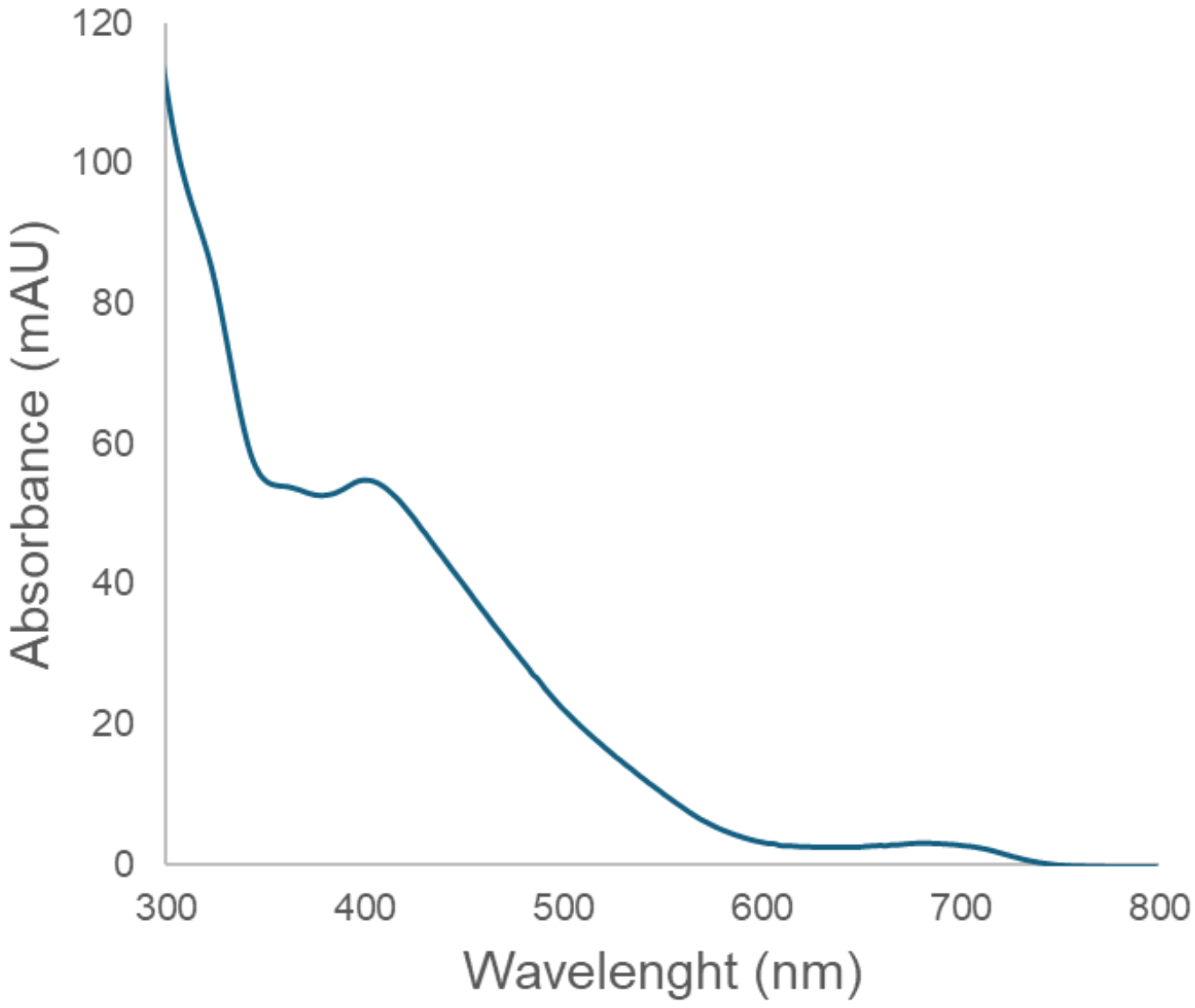

Figure S40. UV-Vis Spectrum of $Lu_3N@I_h\text{-}C_{80}C(CO_2Et)_2$ shows maximum in absorption at 300 nm, 349 nm, 401 nm, 681 nm (*o*-xylene). 5PYE Column (4.6 mm I.D. x 250 mm, particle size: 5 µm, Nacalai Tesque, Inc.). Flow: 0.2 mL/min, Detector: 320 nm.

NMR Characterization

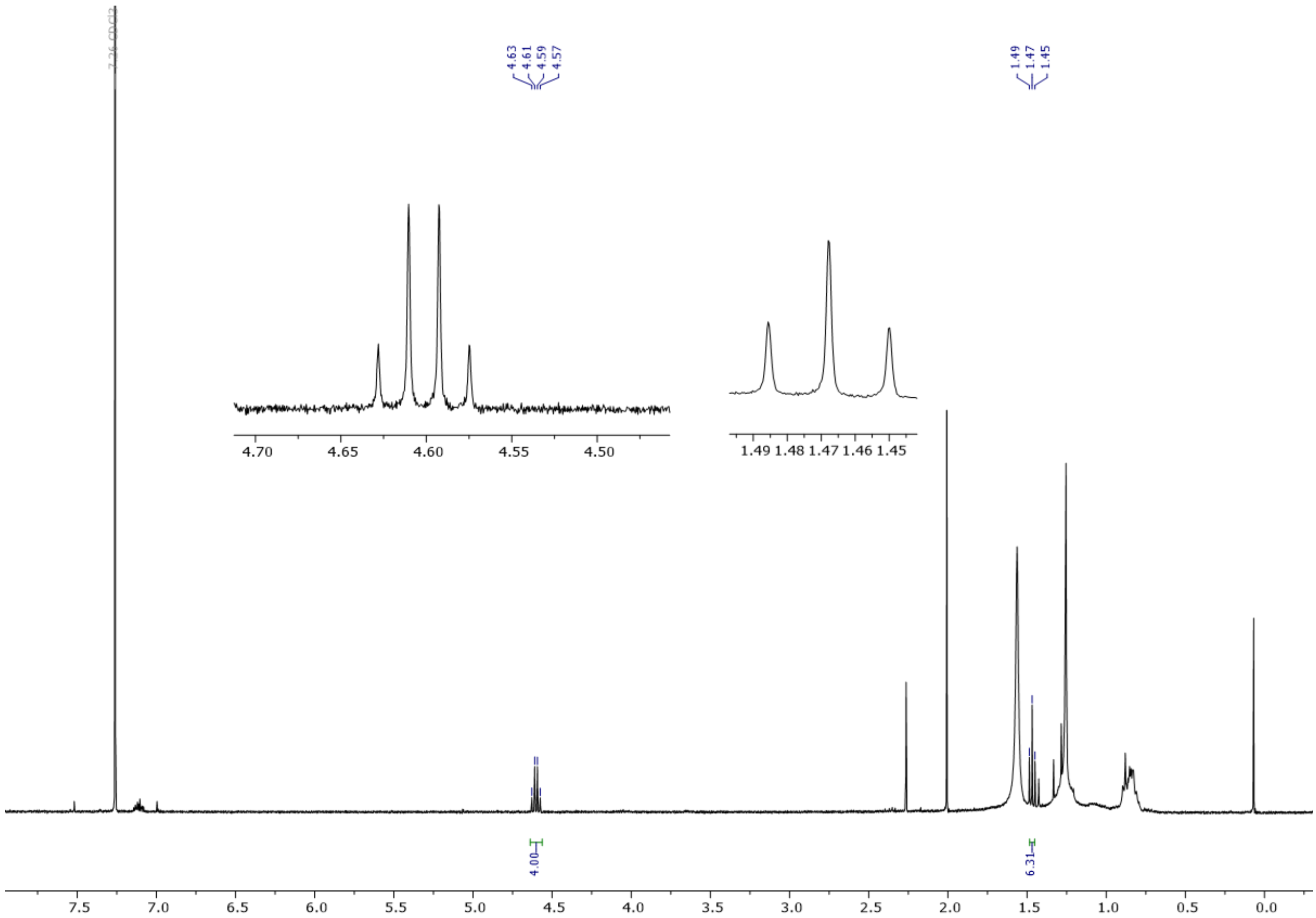

Figure S41. ^{1}H-NMR Spectrum of $Lu_3N@I_h\text{-}C_{80}C(CO_2Et)_2$. Experiment performed in $CDCl_3$ at 298K (400 MHz).

<u>^{1}H-NMR</u> (400 MHz, $CDCl_3$) δ p.p.m.: 4.60 (q, *J*=7.1 Hz, 4H, $-CH_2-$), δ 1.47 (t, *J*=7.1 Hz, 6H, $-CH_3-$).

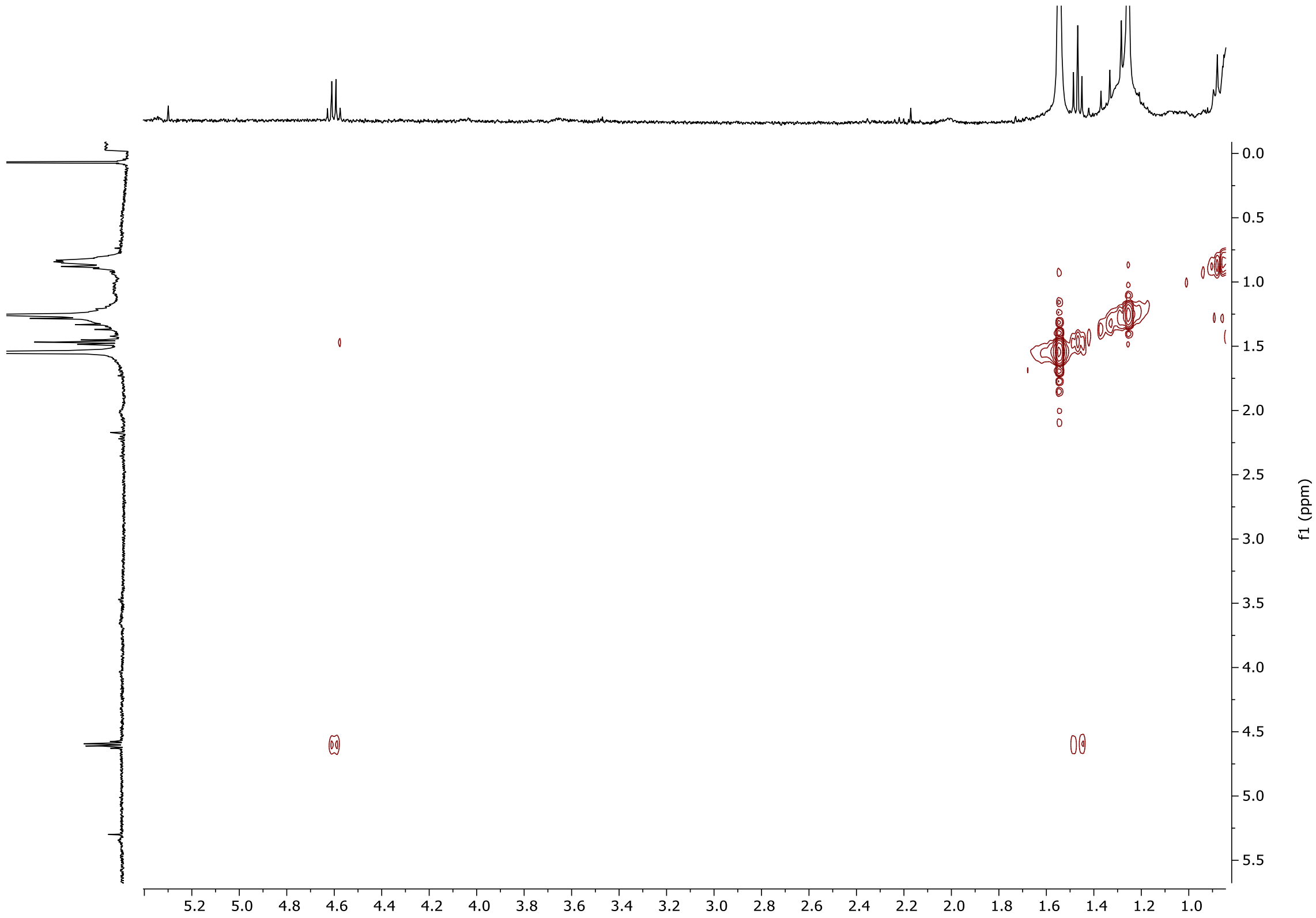

Figure S42. COSY of $Lu_3N@I_h\text{-}C_{80}C(CO_2Et)_2$. Experiment performed in CD_3CN at 298K (400 MHz).

2.2.5. Synthesis of $Sc_3N@I_h$-$C_{80}C(CO_2Et)_2$⊂4·(BArF)$_8$ via Bingel reaction

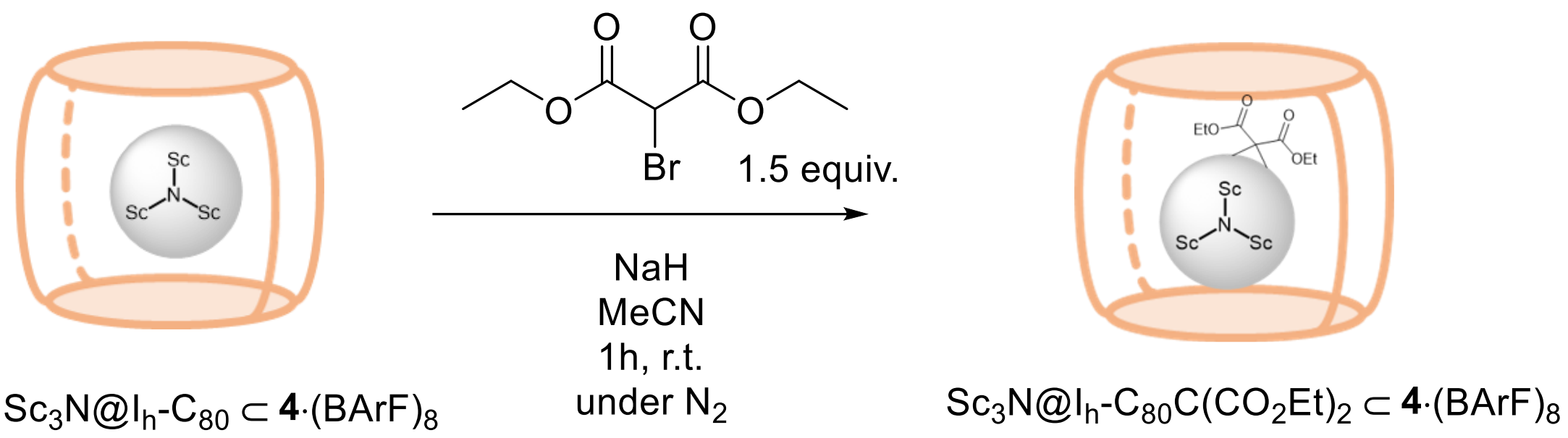

Figure S43. General procedure for Bingel Reaction on $Sc_3N@I_h$-C_{80}⊂**4**·(BArF)$_8$.

15 mg of $Sc_3N@I_h$-C_{80}⊂**4**·(BArF)$_8$ are totally dissolved in 0.5 mL of MeCN, then 1.5 equivalents of the diethyl bromomalonate and 1.5 equivalents of NaH as a base are added sequentially and the reaction is stirred under N_2 at room temperature for 1 hour. Once the $Sc_3N@I_h$-C_{80}⊂**4**·(BArF)$_8$ is consumed and the $Sc_3N@I_h$-$C_{80}C(CO_2Et)_2$⊂**4**·(BArF)$_8$ is formed (checked by HR-MS), the reaction is stopped by filtering and removing the solvent under reduced pressure.

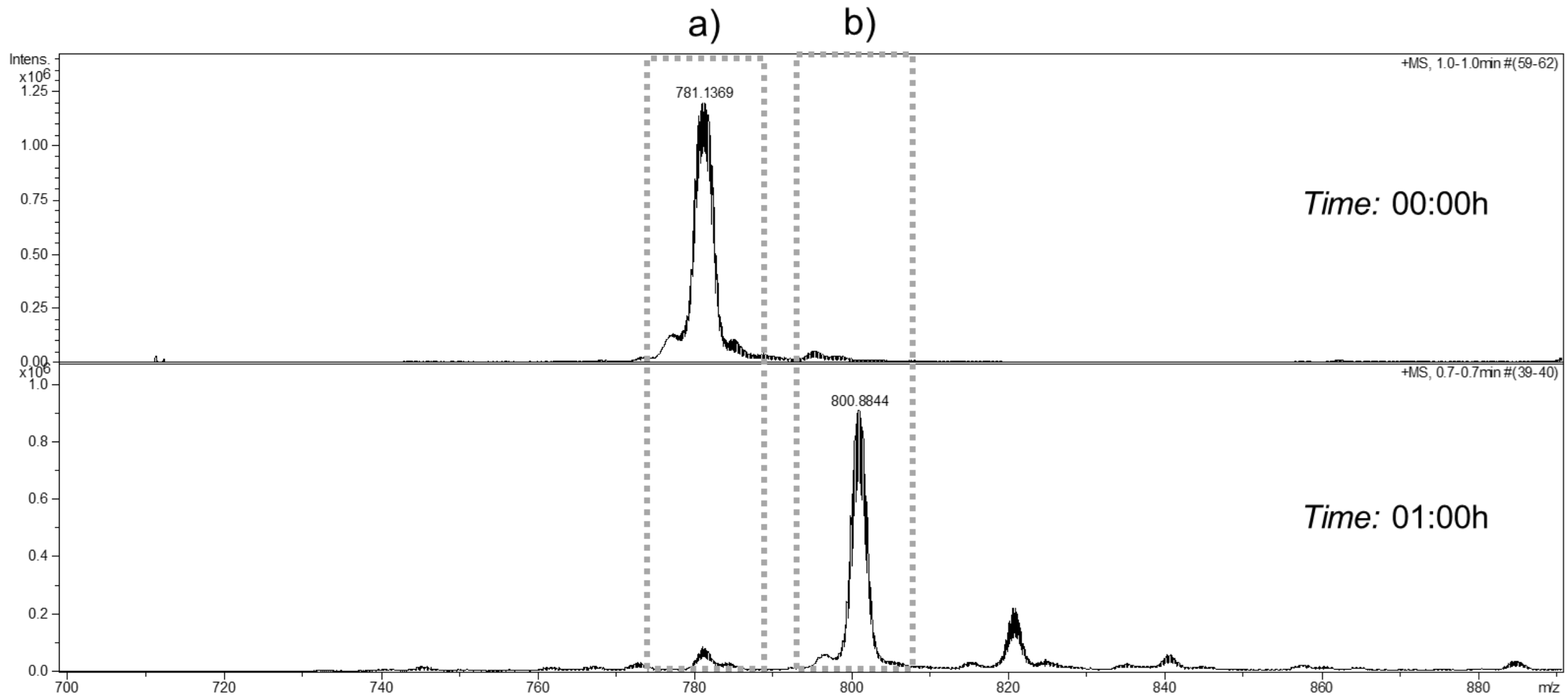

Figure S44. HR-MS monitoring of the $Sc_3N@I_h$-$C_{80}C(CO_2Et)_2$⊂**4**·$(BArF)_8$ formation upon addition of 3 equivalents of diethyl bromomalonate and 3 equivalents of NaH during 1 hour. Focus on the (+8) charge.

a) $Sc_3N@I_h$-C_{80}⊂**4**·$(BArF)_8$ peak disappears upon the additions of the reagents. ($Sc_3N@I_h$-C_{80}⊂[**4**·$(BArF)_0]^{+8}$) calculated 781.1469
b) $Sc_3N@I_h$-$C_{80}C(CO_2Et)_2$⊂**4**·$(BArF)_8$ forming during the 1 hours of the reaction. ($Sc_3N@I_h$-$C_{80}C(CO_2Et)_2$⊂[**4**·$(BArF)_0]^{+8}$) calculated 800.9042

Sample solved in CH_3CN and registered with a Bruker MicroTOF-Q-II mass spectrometer.

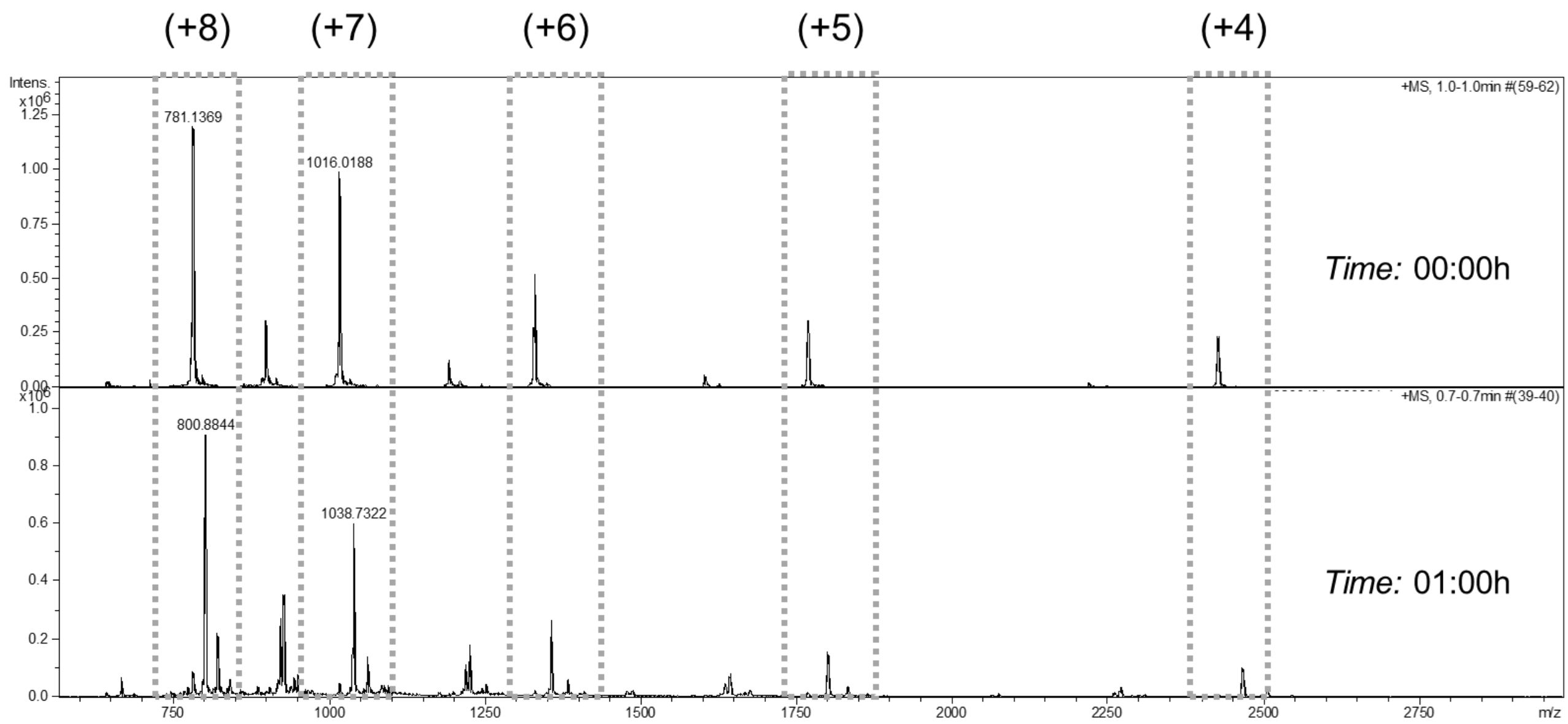

Figure S45. HR-MS monitoring of the $Sc_3N@I_h\text{-}C_{80}C(CO_2Et)_2\subset$**4**·$(BArF)_8$ formation upon addition of 3 equivalents of diethyl bromomalonate and 3 equivalents of NaH in 1 hour. All the charges displayed from (+8) up to (+4) correspond to the presence of $nBArF^-$ as counterion (n= 0-4). Extra peaks correspond to the same species with Cl^- or Br^- as counterion.

Sample solved in CH_3CN and registered with a Bruker MicroTOF-Q-II mass spectrometer.

2.2.6. Characterization of $Sc_3N@I_h$-$C_{80}C(CO_2Et)_2$⊂4·(BArF)$_8$

Crystallization

The solid obtained after the Bingel reaction (15 mg) is dissolved in 4 mL of DCM and 200 µL of CH_3CN and then crystallized by slow diethyl ether diffusion (24 mL) in 4 days. The crystals are dried in the vacuum line (Yield: 92%).

Characterization of $Sc_3N@I_h$-$C_{80}C(CO_2Et)_2$⊂4·(BArF)$_8$

The formation of the host guest complex with the $Lu_3N@I_h$-$C_{80}C(CO_2Et)_2$ adduct inside the nanocapsule **4**·(BArF)$_8$ is characterized by HR-MS and NMR techniques.

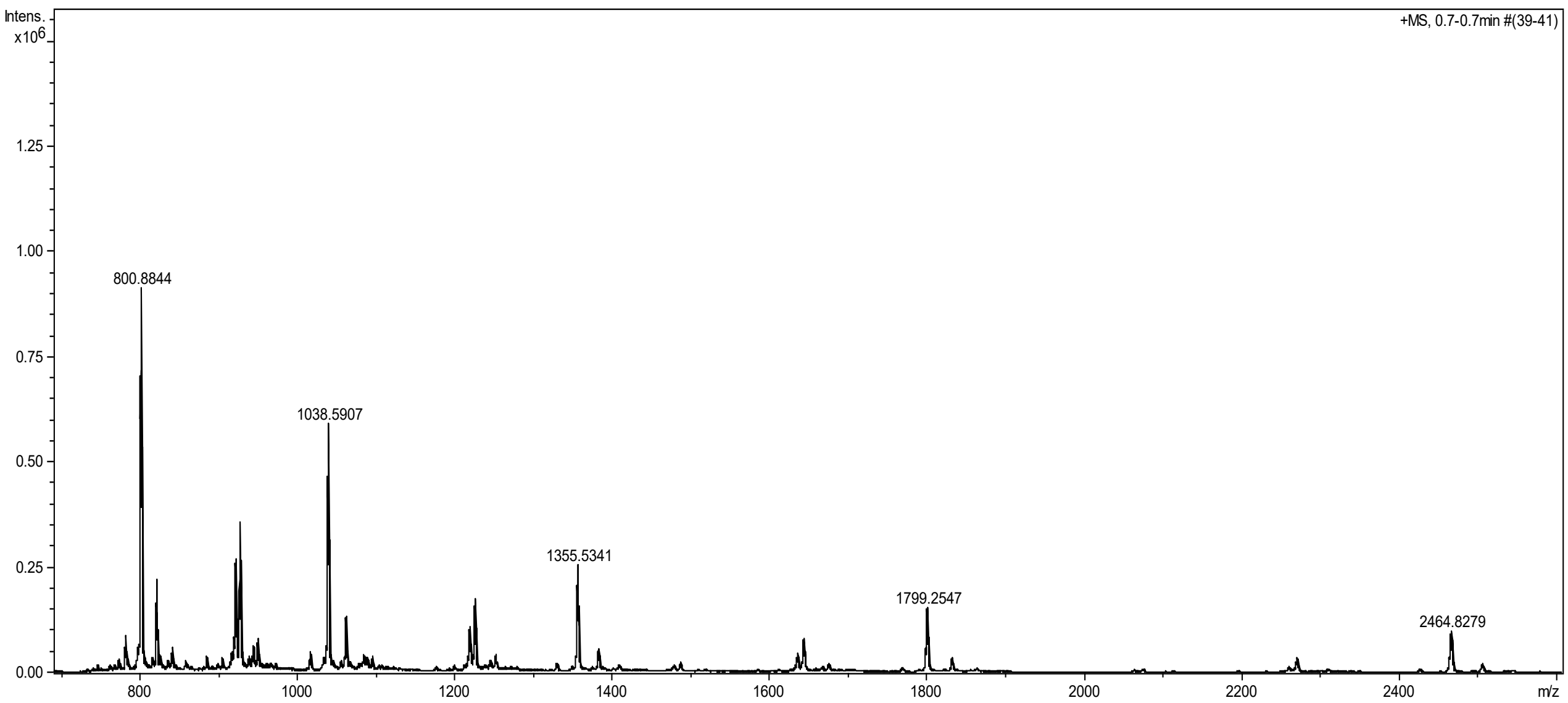

Figure S46. HR-MS spectrum of $Sc_3N@I_h$-$C_{80}C(CO_2Et)_2$⊂**4·**(BArF)$_8$. Sample solved in CH_3CN and registered with a Bruker MicroTOF-Q-II mass spectrometer. All the charges displayed from (+8) up to (+4) correspond to the presence of nBArF$^-$ as counterion (n= 0-4). Extra peaks correspond to the same species with Cl^- or Br^- as counterion.

HR-MS (m/z):

calculated 800.9042and found 800.8844 ($Sc_3N@I_h$-$C_{80}C(CO_2Et)_2$⊂[**4**·(BArF)$_0$]$^{+8}$);
calculated 1038.6144and found 1038.5907 ($Sc_3N@I_h$-$C_{80}C(CO_2Et)_2$⊂[**4**·(BArF)$_1$]$^{+7}$;
calculated 1355.5613and found 1355.5341 ($Sc_3N@I_h$-$C_{80}C(CO_2Et)_2$⊂[**4**·(BArF)$_2$]$^{+6}$;
calculated 1799.2870and found 1799.2547 ($Sc_3N@I_h$-$C_{80}C(CO_2Et)_2$⊂[**4**·(BArF)$_3$]$^{+5}$;
calculated 2464.8756and found 2464.8279 ($Sc_3N@I_h$-$C_{80}C(CO_2Et)_2$⊂[**4**·(BArF)$_4$]$^{+4}$.

NMR Characterization

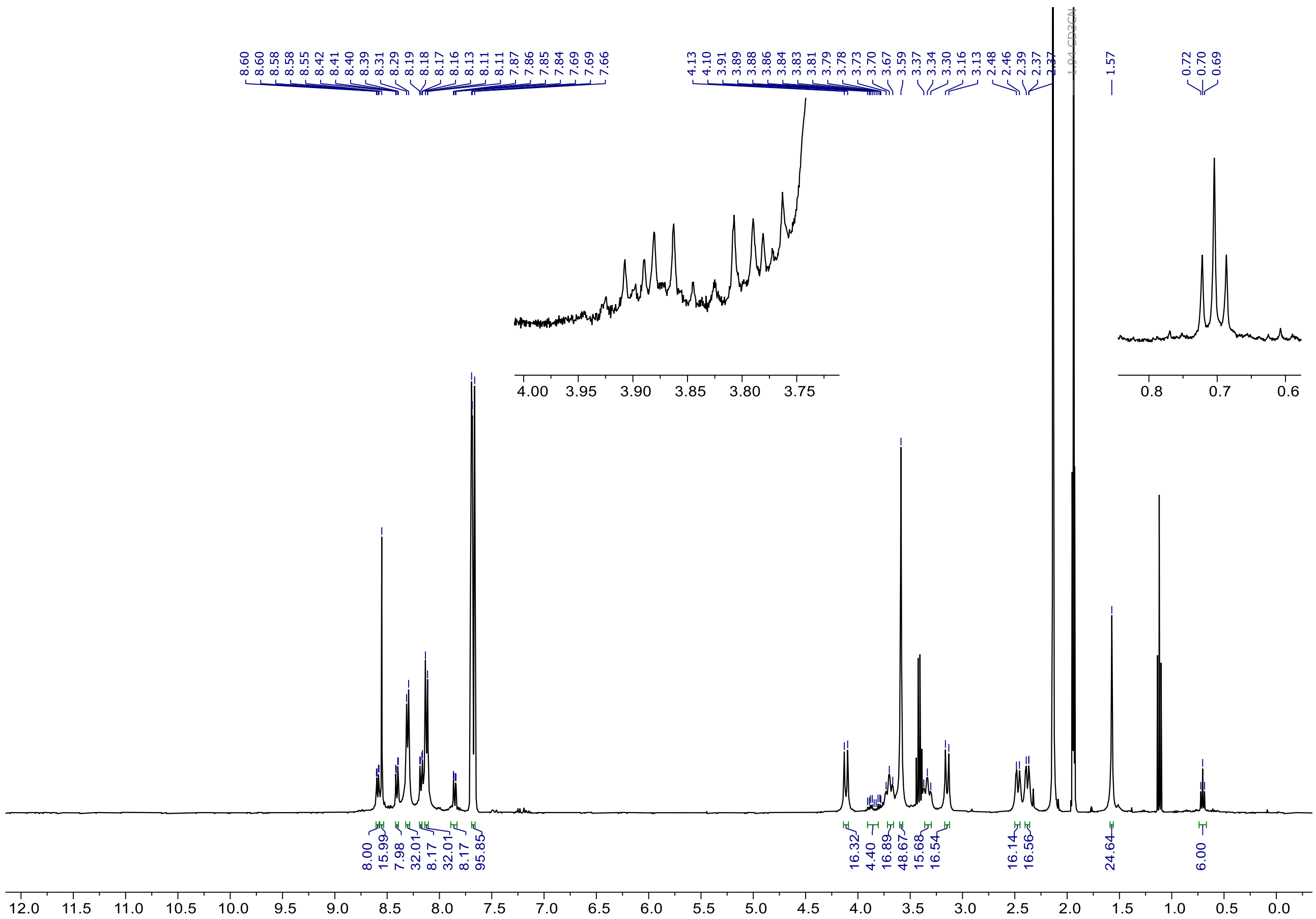

Figure S47. ^{1}H-NMR of $Sc_3N@I_h$-$C_{80}C(CO_2Et)_2$⊂**4**·$(BArF)_8$ (400 MHz, CD_3CN).

^{1}H-NMR (400 MHz, CD_3CN) δ p.p.m.: 8.59 (dd, *J*=7.8, 1.9 Hz, 8H, arom-porph), 8.55 (s, 16H, pyrrole ring), 8.41 (dd, *J*=7.9, 1.8 Hz, 8H, arom-porph), 8.30 (d, *J*=7.9 Hz, 32H, arom-clip), 8.18 (dd, *J*=8.1, 1.8 Hz, 8H, arom-porph), 8.12 (d, *J*=8.1 Hz, 32H, arom-clip), 7.86 (dd, *J*=7.8, 1.9 Hz, 8H, arom-porph), 7.69-7.66 (m, 96H, $BArF^-$), 4.12 (d, *J*=12.9 Hz, 16H), 3.91 – 3.78 (m, 4H), 3.69 (t, *J*= 13.2 Hz, 16H), 3.59 (s, 48H), 3.34 (t, *J*=13.2 Hz, 16H), 3.15 (d, *J*=13.0 Hz, 16H), 2.47 (dd, *J*=36.0, 12.6 Hz, 32H), 1.57 (s, 24H), 0.70 (t, *J*=7.1 Hz, 6H).

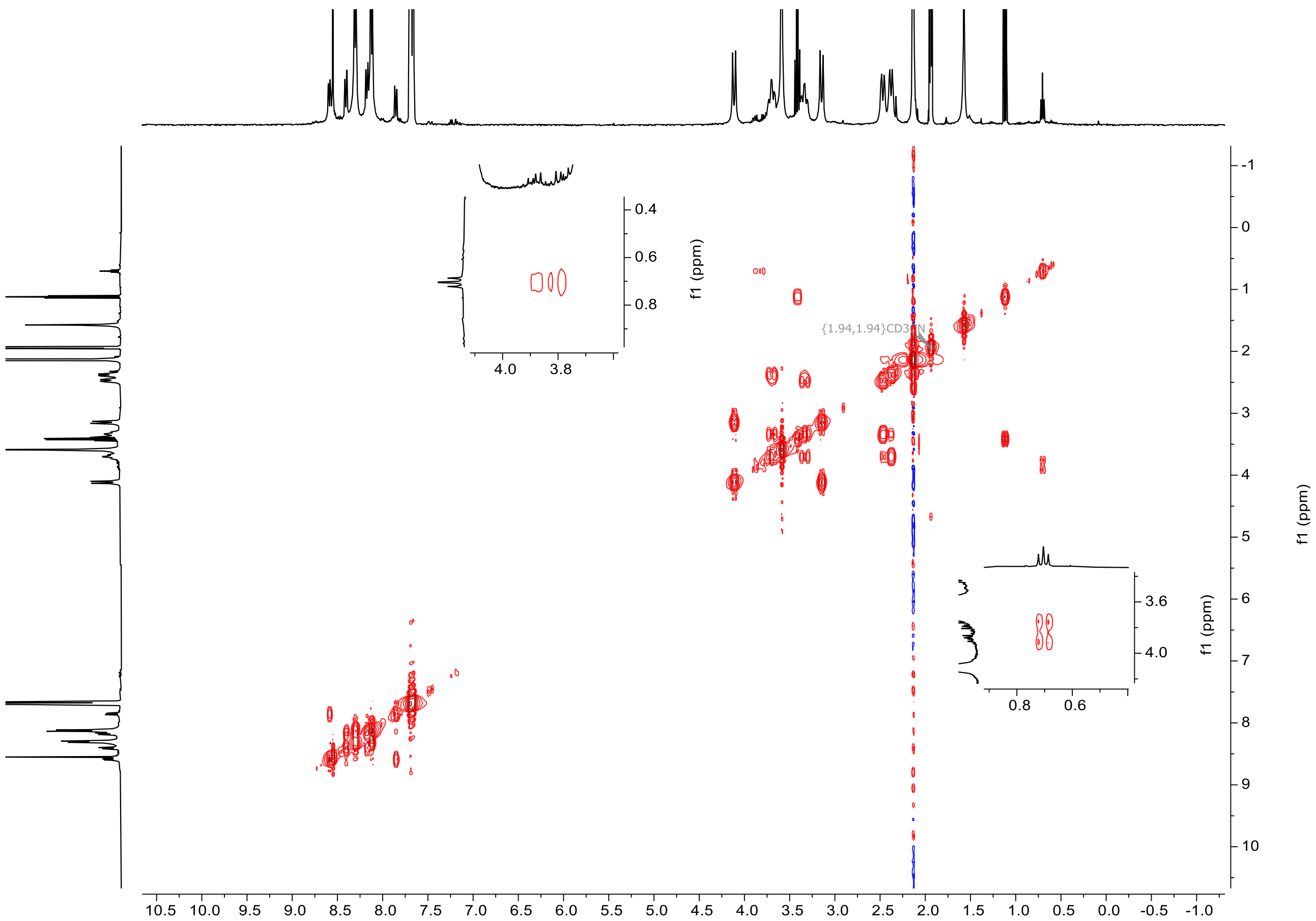

Figure S48. COSY NMR of $Sc_3N@I_h\text{-}C_{80}C(CO_2Et)_2\subset$**4**$\cdot(BArF)_8$. Experiment performed in CD_3CN at 298 K (400 MHz).

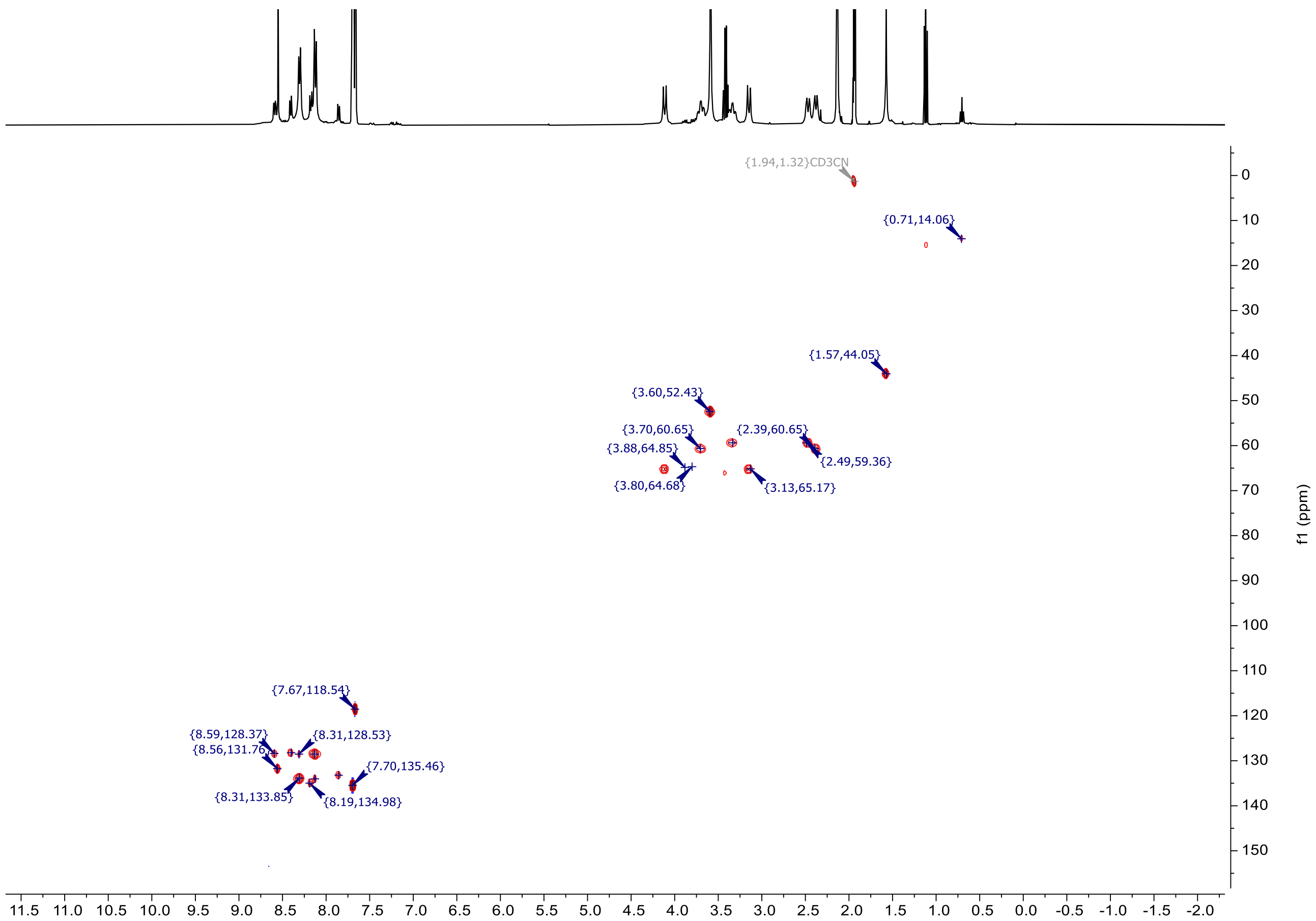

Figure S49. HSQC NMR of $Sc_3N@I_h\text{-}C_{80}C(CO_2Et)_2\subset$**4**·(BArF)$_8$. Experiment performed in CD_3CN at 298 K (400 MHz).

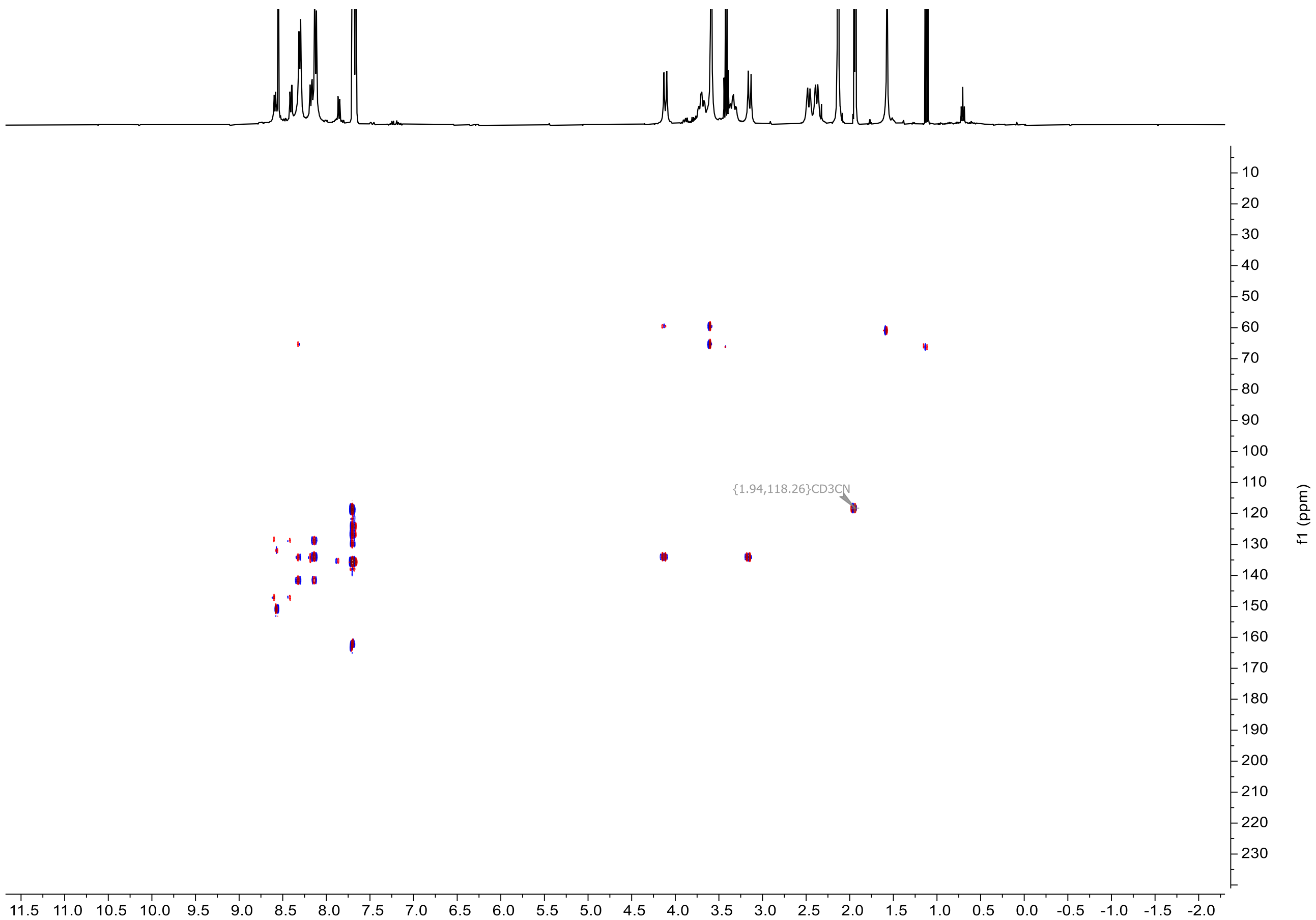

Figure S50. HMBC NMR of $Sc_3N@I_h$-$C_{80}C(CO_2Et)_2\subset$**4**$\cdot(BArF)_8$. Experiment performed in CD_3CN at 298 K (400 MHz).

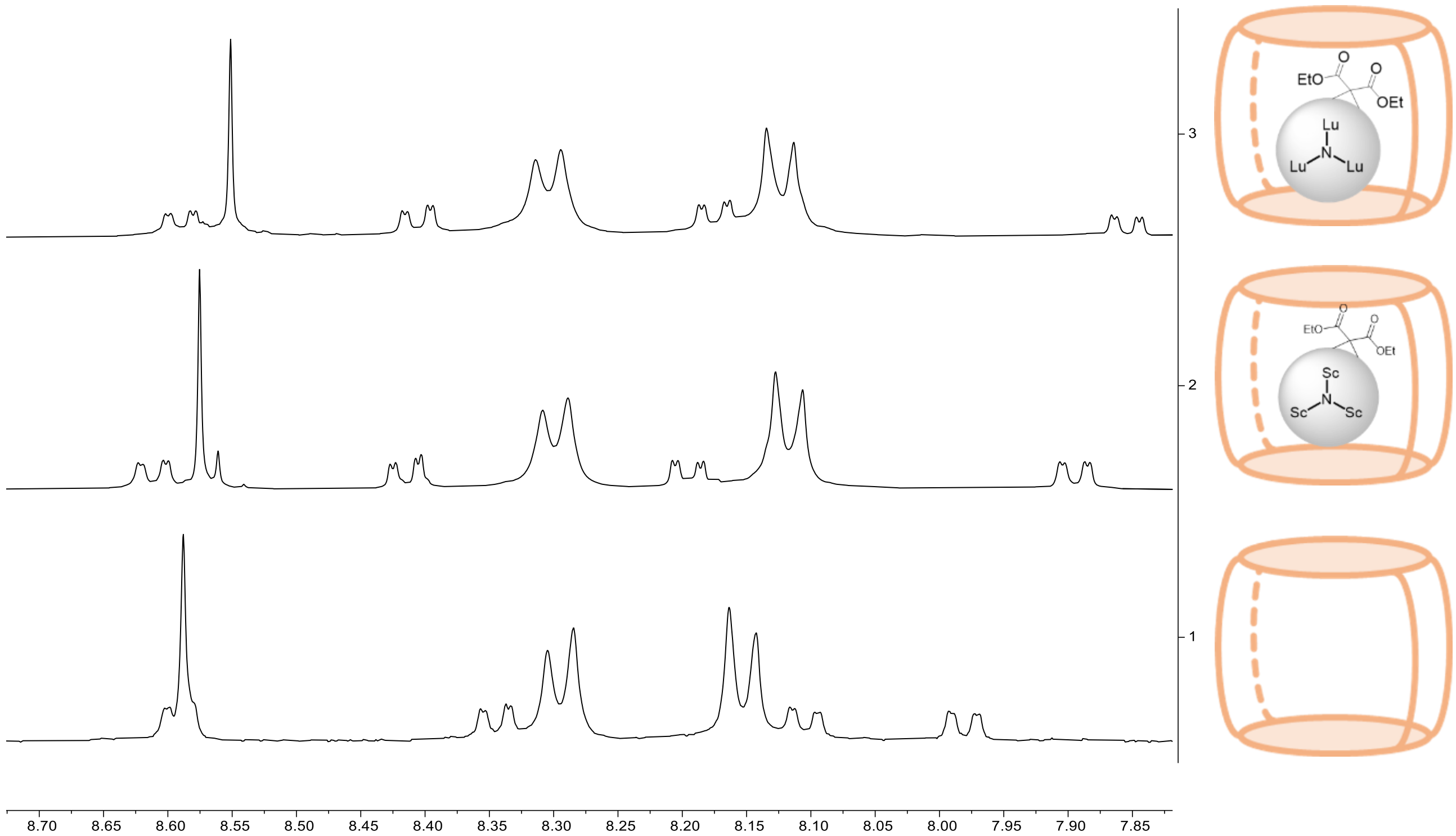

Figure S51. Comparative ^{1}H-NMR spectra (CD_3CN, 400 MHz, 298 K) of $Lu_3N@I_hC_{80}C(CO_2Et)_2\subset$**4**·$(BArF)_8$ (up), $Sc_3N@I_hC_{80}C(CO_2Et)_2\subset$**4**·$(BArF)_8$ (middle) and **4**·$(BArF)_8$ (down). Changes in the chemical shift upon encapsulation, focus on the aromatic region of the spectra.

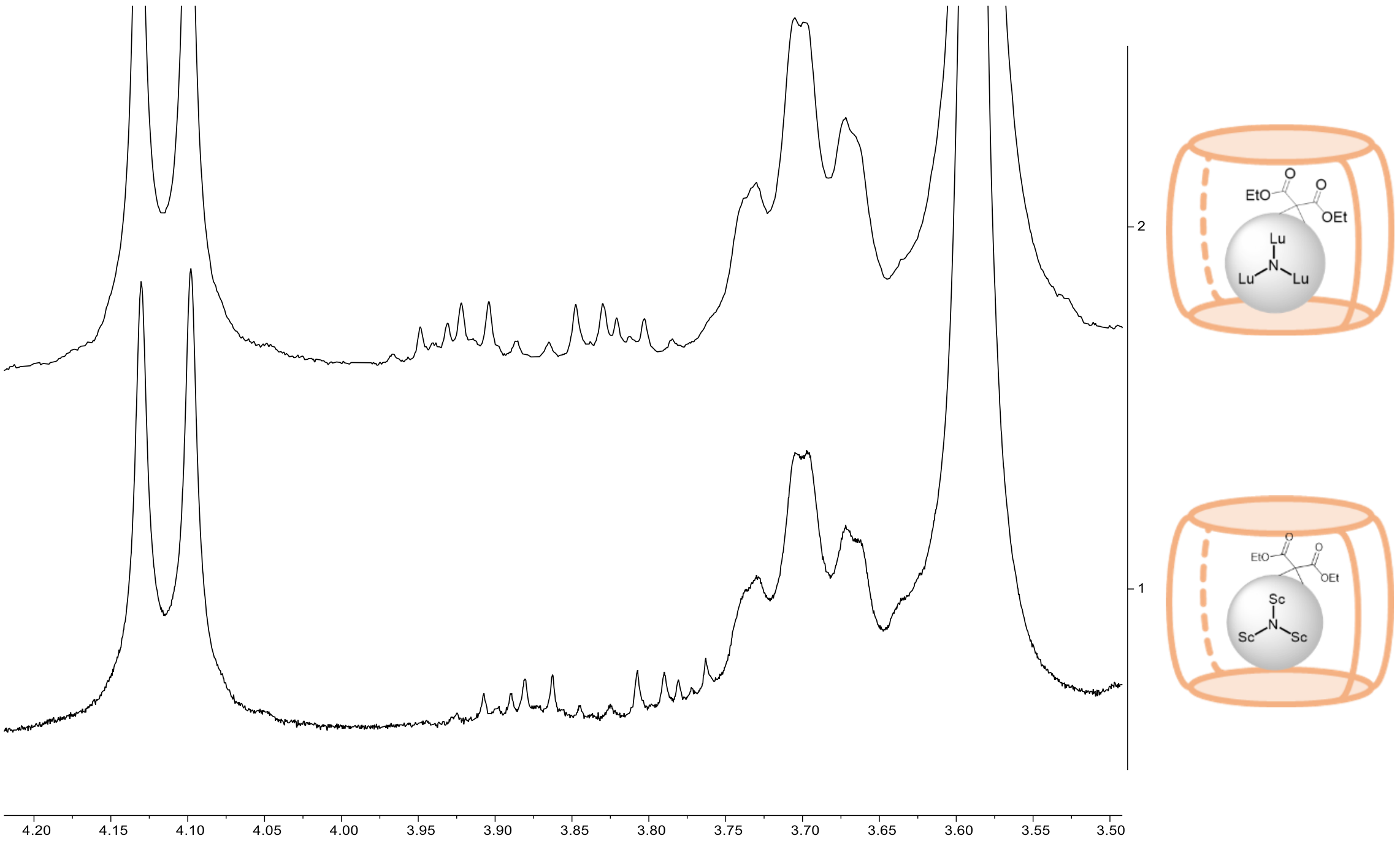

Figure S52. Comparative ^{1}H-NMR spectra (CD_3CN, 400 MHz, 298 K) of $Lu_3N@I_hC_{80}C(CO_2Et)_2\subset$**4**$\cdot(BArF)_8$ (up), $Sc_3N@I_hC_{80}C(CO_2Et)_2\subset$**4**$\cdot(BArF)_8$ (down), showing similarities in the multiplicity and chemical shift of the functionalization moiety.

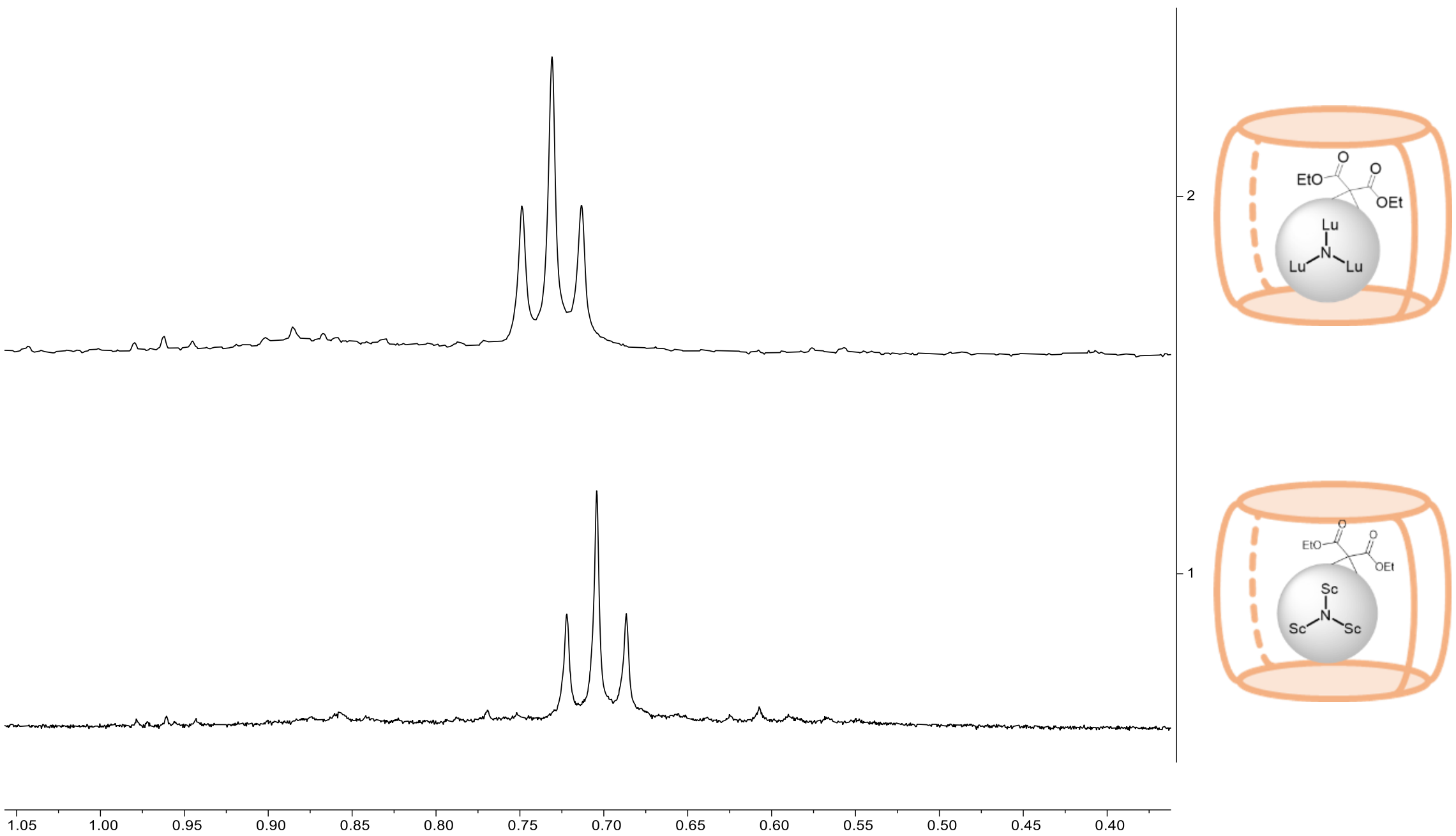

Figure S53. Comparative ^{1}H-NMR spectra (CD_3CN, 400 MHz, 298 K) of $Lu_3N@I_hC_{80}C(CO_2Et)_2\subset$**4**$\cdot(BArF)_8$ (up), $Sc_3N@I_hC_{80}C(CO_2Et)_2\subset$**4**$\cdot(BArF)_8$ (down), showing similarities in the multiplicity and chemical shift of the functionalization moiety.

2.3. Mask-mediated synthesis of $M_3N@I_h$-$C_{80}[C(CO_2Et)_2]_2$⊂4·(BArF)$_8$ via Bingel reaction

2.3.1. Synthesis and characterization of $Lu_3N@I_h$-$C_{80}[C(CO_2Et)_2]_2$ via Bingel reaction starting from $Lu_3N@I_h$-C_{80}⊂4·(BArF)$_8$

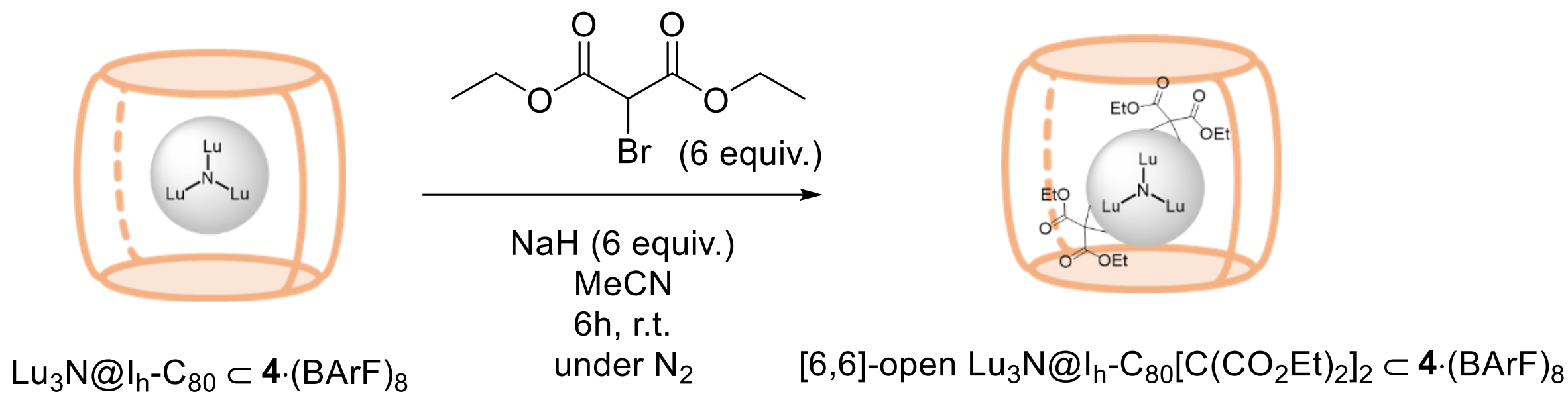

Figure S54. General procedure for Bingel Reaction via supramolecular mask strategy on $Lu_3N@I_h$-C_{80}⊂**4**·(BArF)$_8$ to obtain $Lu_3N@I_h$-$C_{80}[C(CO_2Et)_2]_2$⊂**4**·(BArF)$_8$.

15 mg of $Lu_3N@I_h$-C_{80}⊂**4**·(BArF)$_8$ are totally dissolved in 0.5 mL of MeCN, then 6 equivalents of the diethyl bromomalonate and 6 equivalents of NaH as a base are added sequentially and the reaction is stirred under N_2 at room temperature for 6 hours. Once the $Lu_3N@I_h$-$C_{80}[C(CO_2Et)_2]_2$⊂**4**·(BArF)$_8$ is accumulated as the major product and the $Lu_3N@I_h$-C_{80}⊂**4**·(BArF)$_8$ is mostly consumed (checked by HR-MS), the reaction is stopped by filtering and removing the solvent under reduced pressure. In these reaction conditions the nanocapsule is breaking (as it can be seen in figure S52), giving poor yields of the desired product.

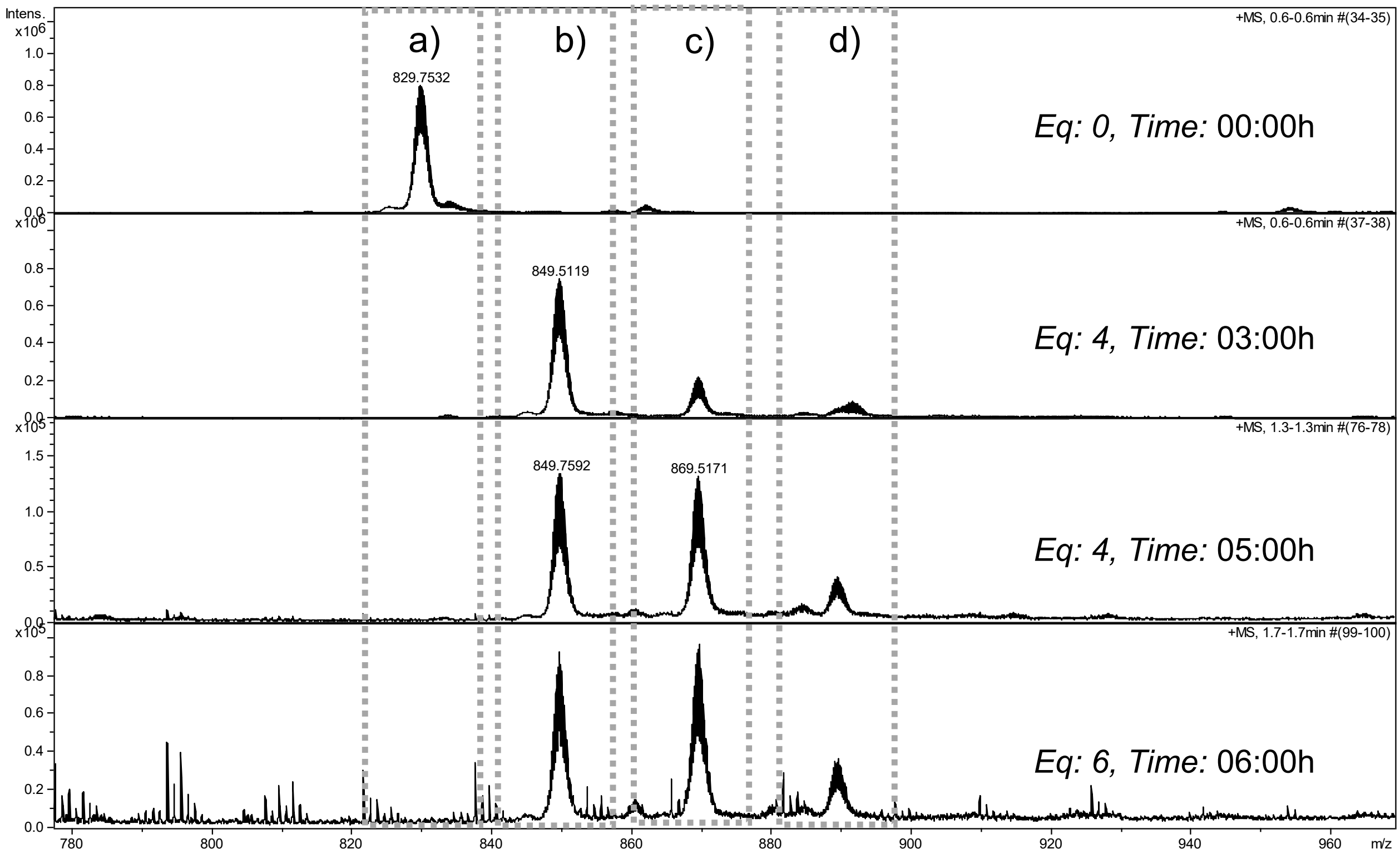

Figure S55. HR-MS monitoring of the $Lu_3N@I_h$-$C_{80}[C(CO_2Et)_2]_2$⊂**4**·$(BArF)_8$ formation upon addition of 6 equivalents of diethyl bromomalonate and 6 equivalents of NaH in 4 hours.

a) Changes in the peak of $Lu_3N@I_h$-C_{80}⊂**4**·$(BArF)_8$

b) Changes in the peak of $Lu_3N@I_h$-$C_{80}C(CO_2Et)_2$⊂**4**·$(BArF)_8$

c) Changes in the peak of $Lu_3N@I_h$-$C_{80}[C(CO_2Et)_2]_2$⊂**4**·$(BArF)_8$

d) Changes in the peak of $Lu_3N@I_h$-$C_{80}[C(CO_2Et)_2]_3$⊂**4**·$(BArF)_8$

2.3.2. Synthesis and characterization of $Lu_3N@I_h$-$C_{80}[C(CO_2Et)_2]_2$ via Bingel reaction starting from $Lu_3N@I_h$-$C_{80}C(CO_2Et)_2$

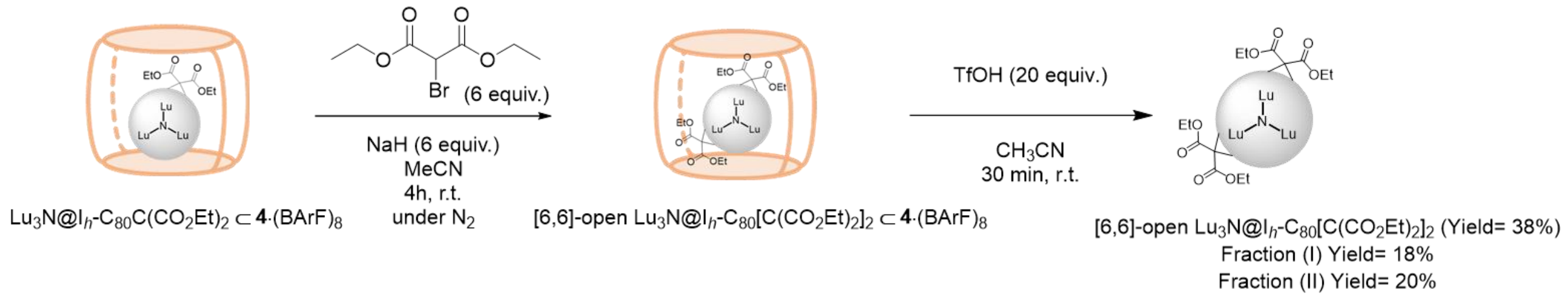

Figure S56. General procedure for Bingel Reaction via supramolecular mask strategy on $Lu_3N@I_h$-$C_{80}C(CO_2Et)_2 \subset \mathbf{4}\cdot(BArF)_8$ to obtain $Lu_3N@I_h$-$C_{80}[C(CO_2Et)_2]_2 \subset \mathbf{4}\cdot(BArF)_8$.

15 mg of $Lu_3N@I_h$-$C_{80}C(CO_2Et)_2 \subset \mathbf{4}\cdot(BArF)_8$ are totally dissolved in 0.5 mL of MeCN, then 6 equivalents of the diethyl bromomalonate and 6 equivalents of NaH as a base are added sequentially and the reaction is stirred under N_2 at room temperature for 4 hours. Once the $Lu_3N@I_h$-$C_{80}[C(CO_2Et)_2]_2 \subset \mathbf{4}\cdot(BArF)_8$ is accumulated as the major product and the $Lu_3N@I_h$-$C_{80}C(CO_2Et)_2 \subset \mathbf{4}\cdot(BArF)_8$ is mostly consumed (checked by HR-MS), the reaction is stopped by filtering and removing the solvent under reduced pressure.

The crude $Lu_3N@I_h$-$C_{80}[C(CO_2Et)_2]_2 \subset \mathbf{4}\cdot(BArF)_8$ reaction mixture is dissolved in CH_3CN (1.5 mL) and an excess of TfOH (20 equivalents) is added to afford the disassembly of the nanocapsule. The mixture is stirred at room temperature during 30 min, then, the solution is dried with N_2 gas flow and $Lu_3N@I_h$-C_{80}-adducts are obtained after Toluene addition and filtration of the solid to remove the broken nanocapsule. Separation of the $Lu_3N@I_h$-$C_{80}[C(CO_2Et)_2]_2$ adducts from the unreacted $Lu_3N@I_h$-$C_{80}C(CO_2Et)_2$ and the undesired $Lu_3N@I_h$-$C_{80}[C(CO_2Et)_2]_3$ adducts is obtained by preparative TLC (SiO_2) in Toluene 100%. Two lines, Fraction (I) and Fraction (II), corresponding to $Lu_3N@I_h$-$C_{80}[C(CO_2Et)_2]_2$ adducts are observed with yield of Fraction (I) 18% and Fraction (II) 20%.

The yield was calculated by NMR using mesitylene as internal standard.

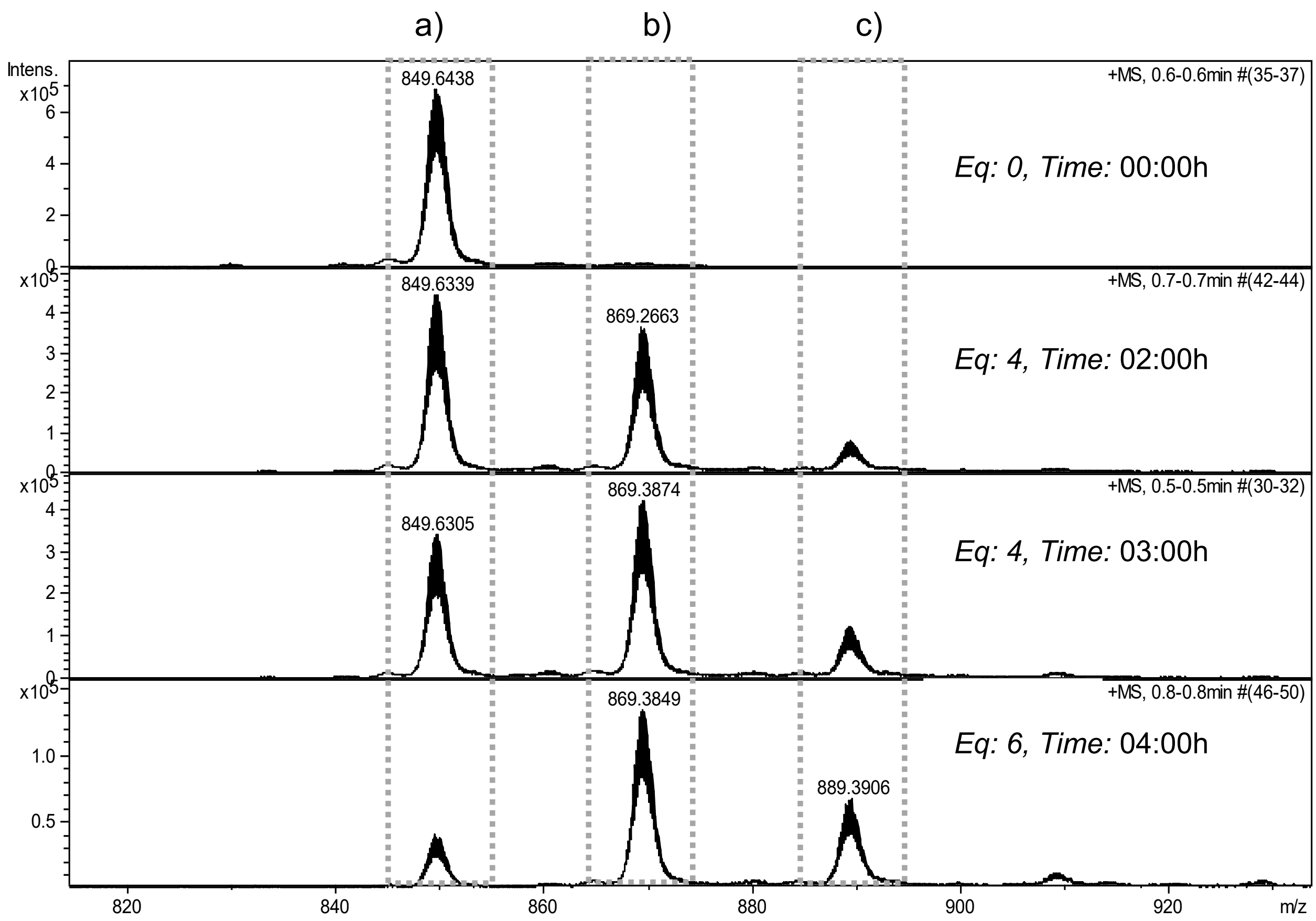

Figure S57. HR-MS monitoring of the $Lu_3N@I_h$-$C_{80}[C(CO_2Et)_2]_2$⊂**4**·$(BArF)_8$ formation upon addition of 6 equivalents of diethyl bromomalonate and 6 equivalents of NaH in 4 hours.

a) Changes in the peak of $Lu_3N@I_h$-$C_{80}C(CO_2Et)_2$⊂**4**·$(BArF)_8$

b) Changes in the peak of $Lu_3N@I_h$-$C_{80}[C(CO_2Et)_2]_2$⊂**4**·$(BArF)_8$

c) Changes in the peak of $Lu_3N@I_h$-$C_{80}[C(CO_2Et)_2]_3$⊂**4**·$(BArF)_8$

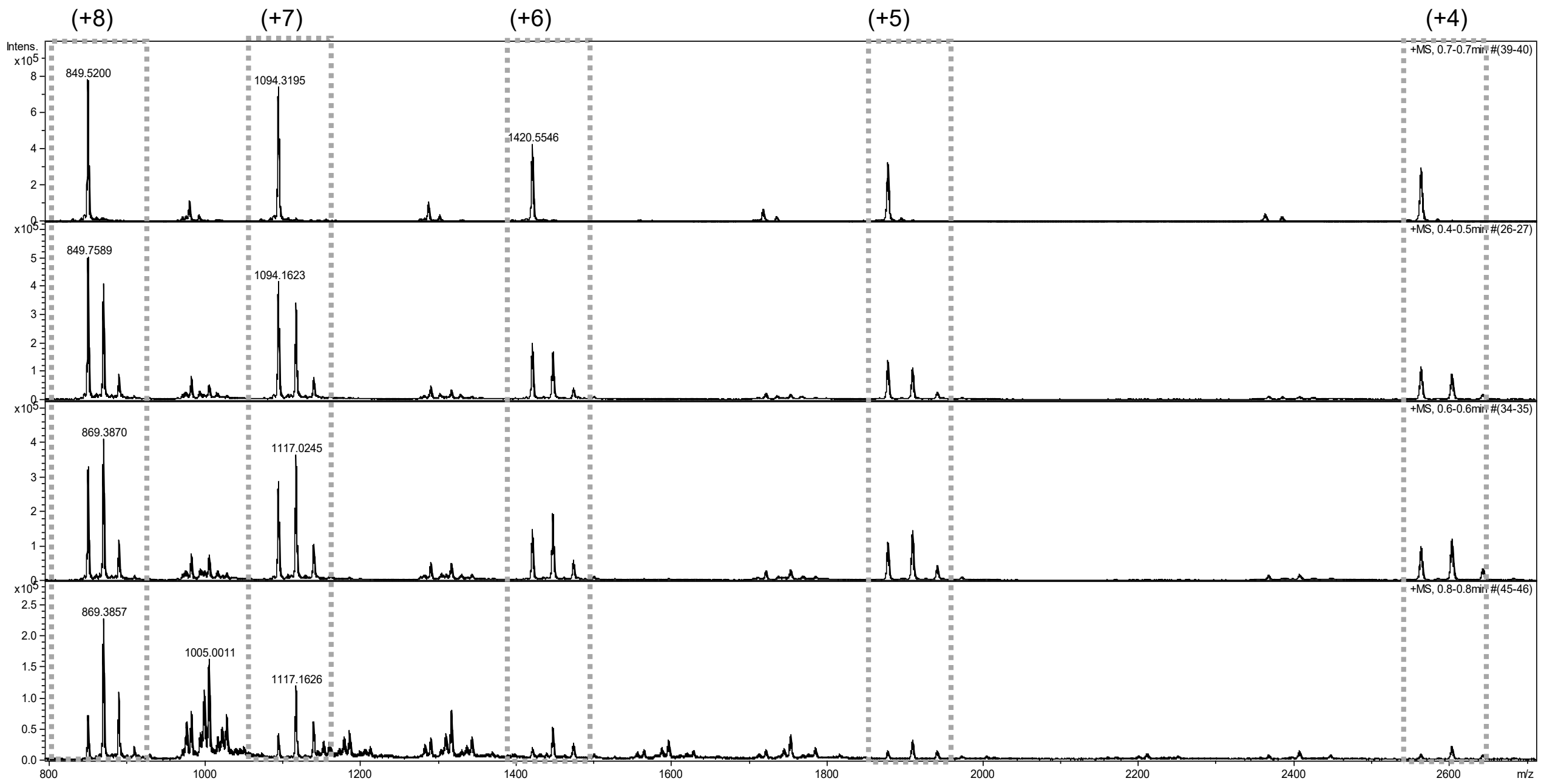

Figure S58. HR-MS monitoring of the $Lu_3N@I_h$-$C_{80}[C(CO_2Et)_2]\subset$**4**·$(BArF)_8$ formation upon addition of 6 equivalents of diethyl bromomalonate and 6 equivalents of NaH in 4 hours. All the charges displayed from (+8) up to (+4) correspond to the presence of $nBArF^-$ counterion (n=0-4). Extra peaks correspond to the same species with the presence of other counter ions such as Br^- and Cl^-.

Sample solved in CH3CN and registered with a Bruker MicroTOF-Q-II mass spectrometer.

Isolation and Characterization of $Lu_3N@I_h$-$C_{80}[C(CO_2Et)_2]_2$

<u>Preparative TLC</u>

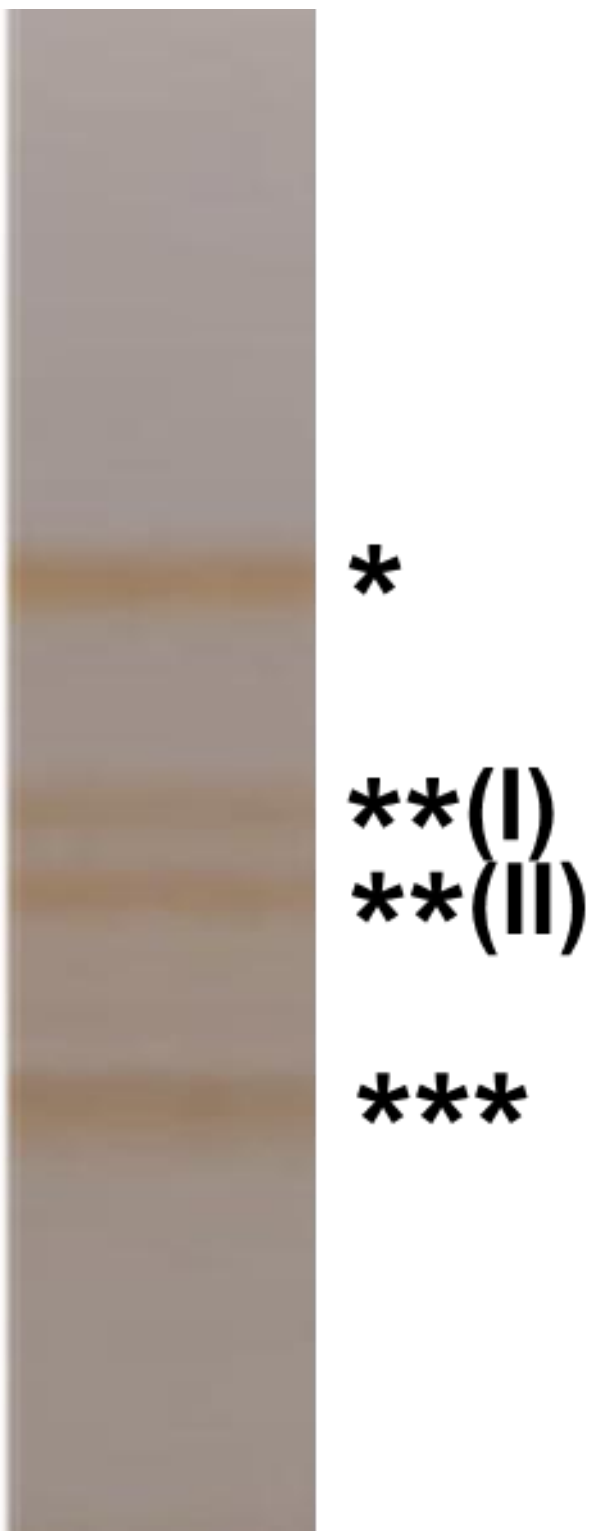

Figure S59. Preparative TLC of crude $Lu_3N@I_h$-$C_{80}[C(CO_2Et)_2]_2$ after breaking the nanocapsule (SiO_2, Toluene).

* $Lu_3N@I_h$-$C_{80}C(CO_2Et)_2]$
**(I) $Lu_3N@I_h$-$C_{80}[C(CO_2Et)_2]_2$ Fraction (I)
**(II) $Lu_3N@I_h$-$C_{80}[C(CO_2Et)_2]_2$ Fraction (II)
*** $Lu_3N@I_h$-$C_{80}[C(CO_2Et)_2]_3$

LDI Mass Spectrum

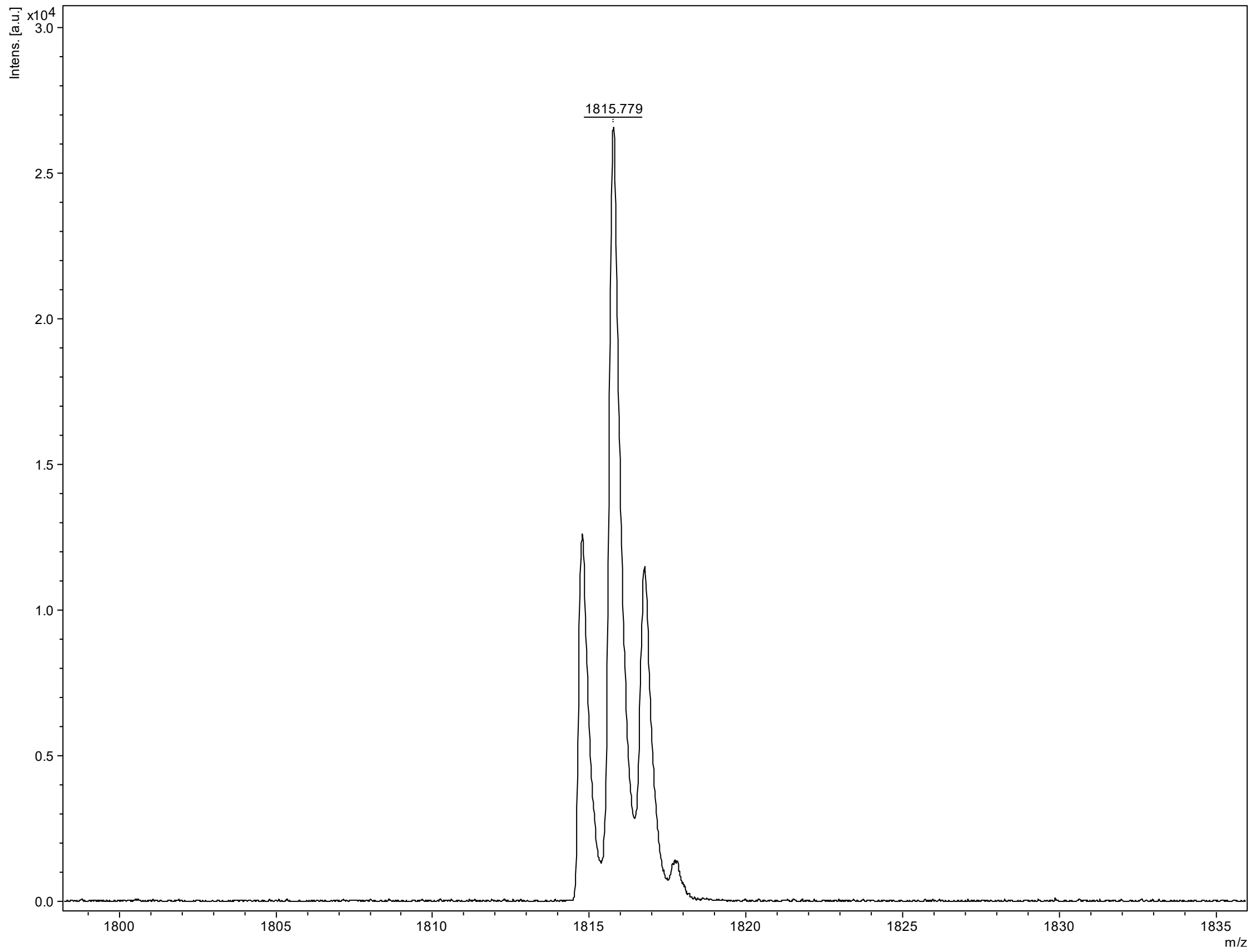

Figure S60. LDI-MS Spectrum of $Lu_3N@I_h$-$C_{80}[C(CO_2Et)_2]_2$. Mixture of Fraction (I) and Fraction (II). Calculated 1816.07 and found 1815.779. Positive mode, using DCTB/NaTFA as matrix.

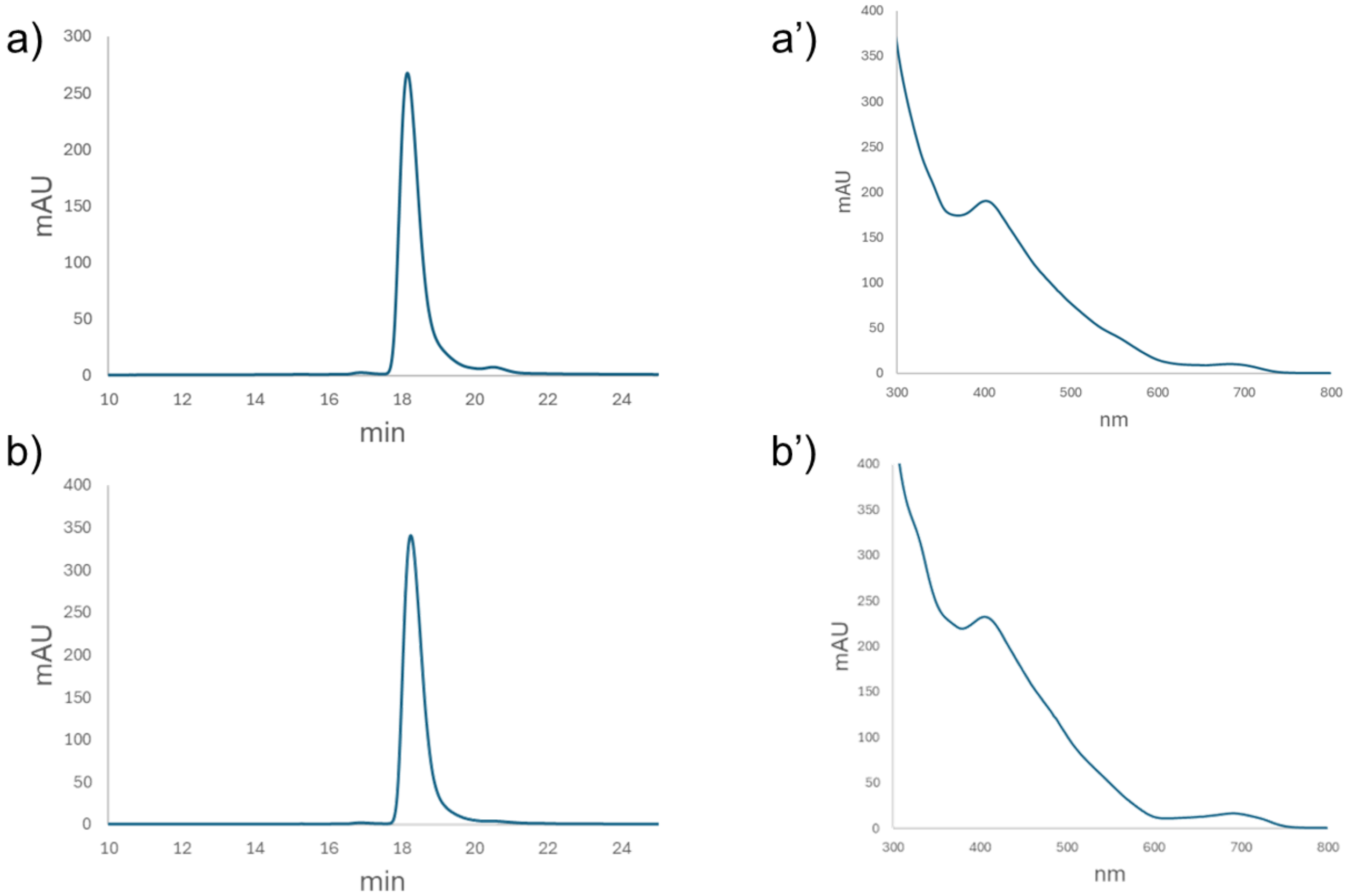

Figure S61. HPLC-DAD (a) Fraction (I) and (b) Fraction (II) of $Lu_3N@I_h\text{-}C_{80}[C(CO_2Et)_2]_2$.

UV-Vis Spectrum (a') Fraction (I) and (b') Fraction (II) of $Lu_3N@I_h\text{-}C_{80}[C(CO_2Et)_2]_2$.

5PYE Column (4.6 mm I.D. x 250 mm, particle size: 5 µm, Nacalai Tesque, Inc.). 100% *o*-xylene. Flow: 0.2 mL/min, Detector: 320 nm.

<u>NMR Characterization</u>

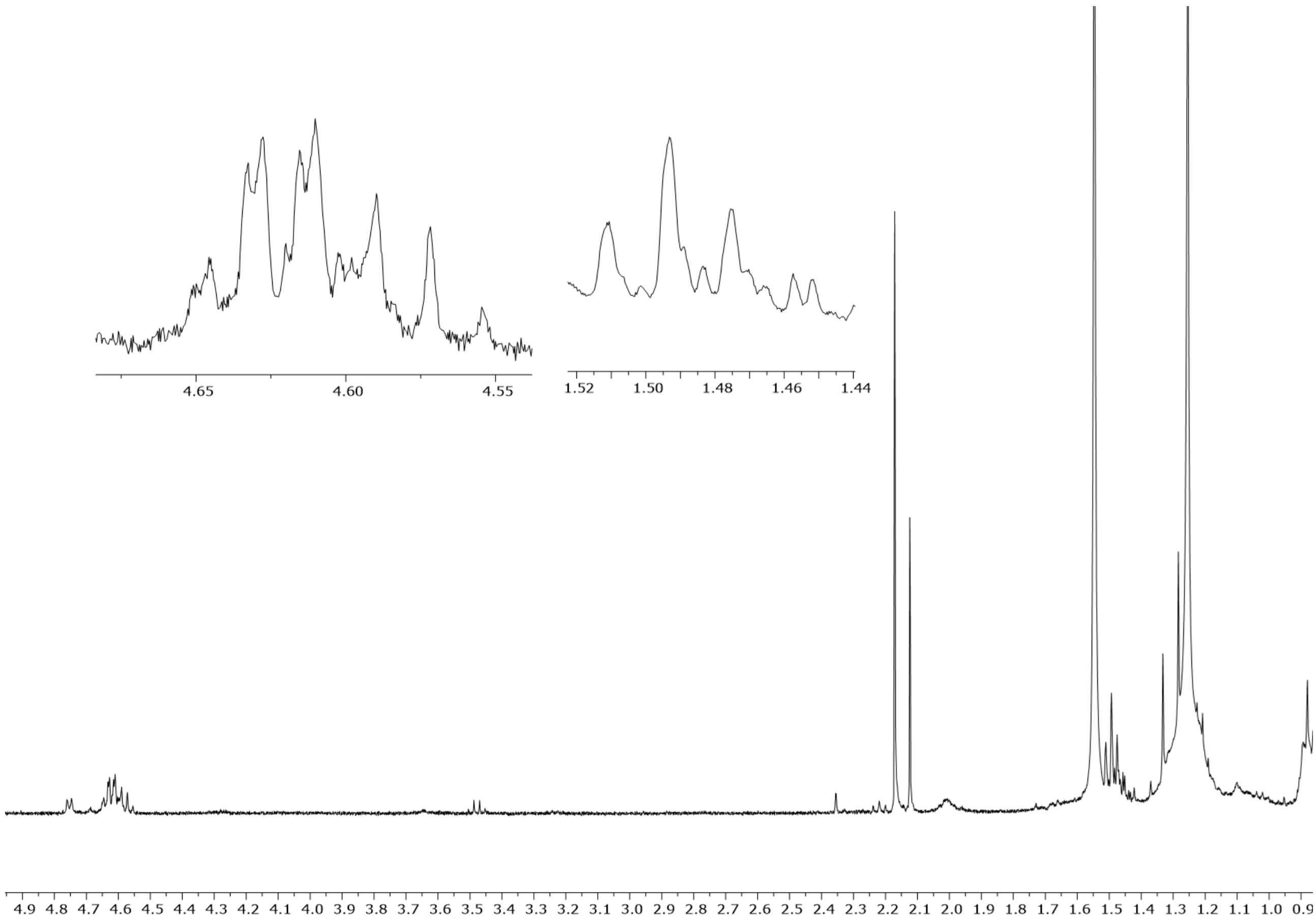

Figure S62. ^{1}H-NMR Spectrum of Fraction (I) of $Lu_3N@I_h$-$C_{80}[C(CO_2Et)_2]_2$. Experiment performed in $CDCl_3$ at 298K (400 MHz).

<u>^{1}H-NMR</u> (400 MHz, $CDCl_3$) δ p.p.m.: 4.63 (m, 4H, $-CH_2-$), δ 1.49 (m, 6H, $-CH_3-$).

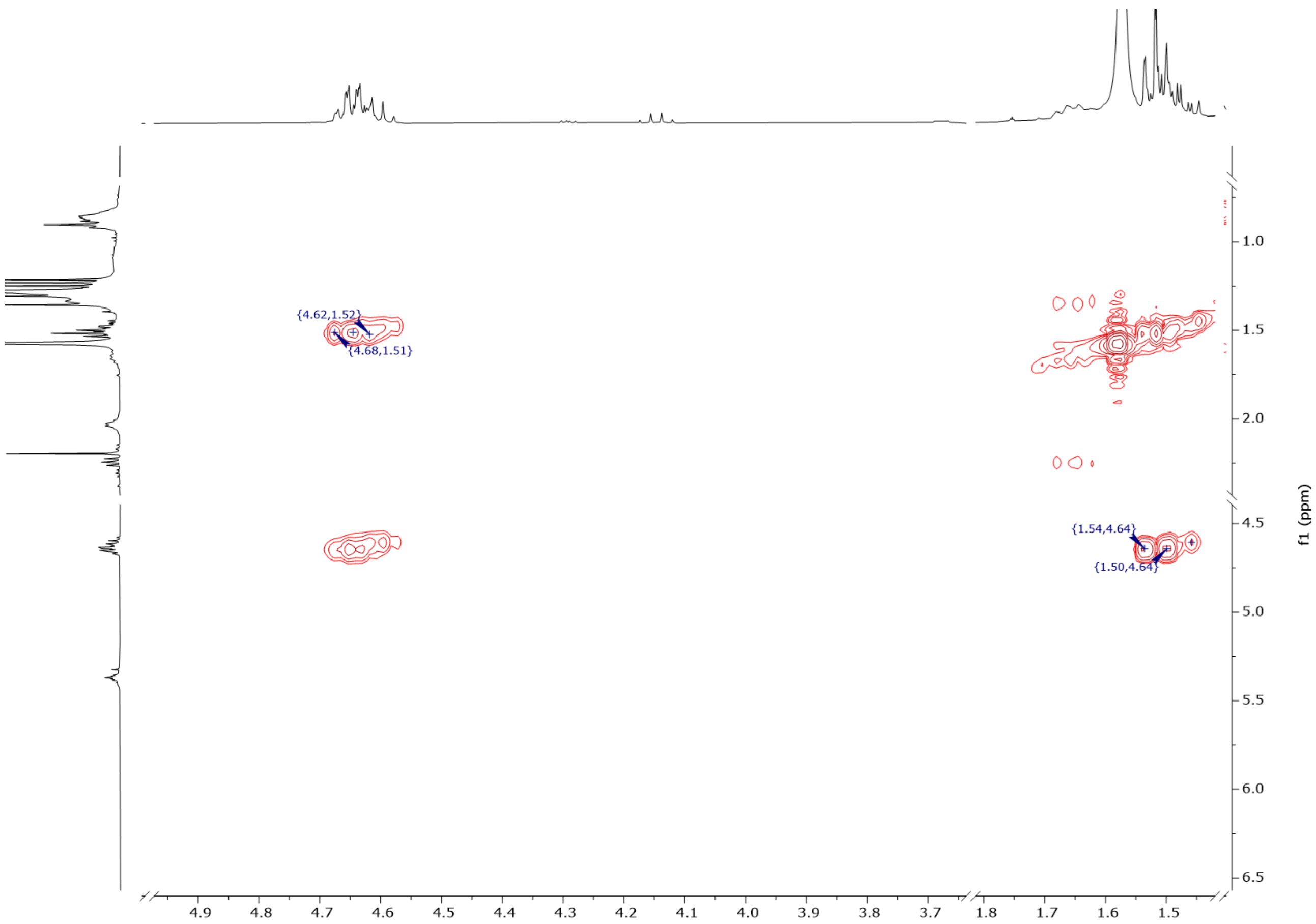

Figure S63. COSY of Fraction (I) of $Lu_3N@I_h$-$C_{80}[C(CO_2Et)_2]_2$. Experiment performed in CD_3CN at 298K (400 MHz).

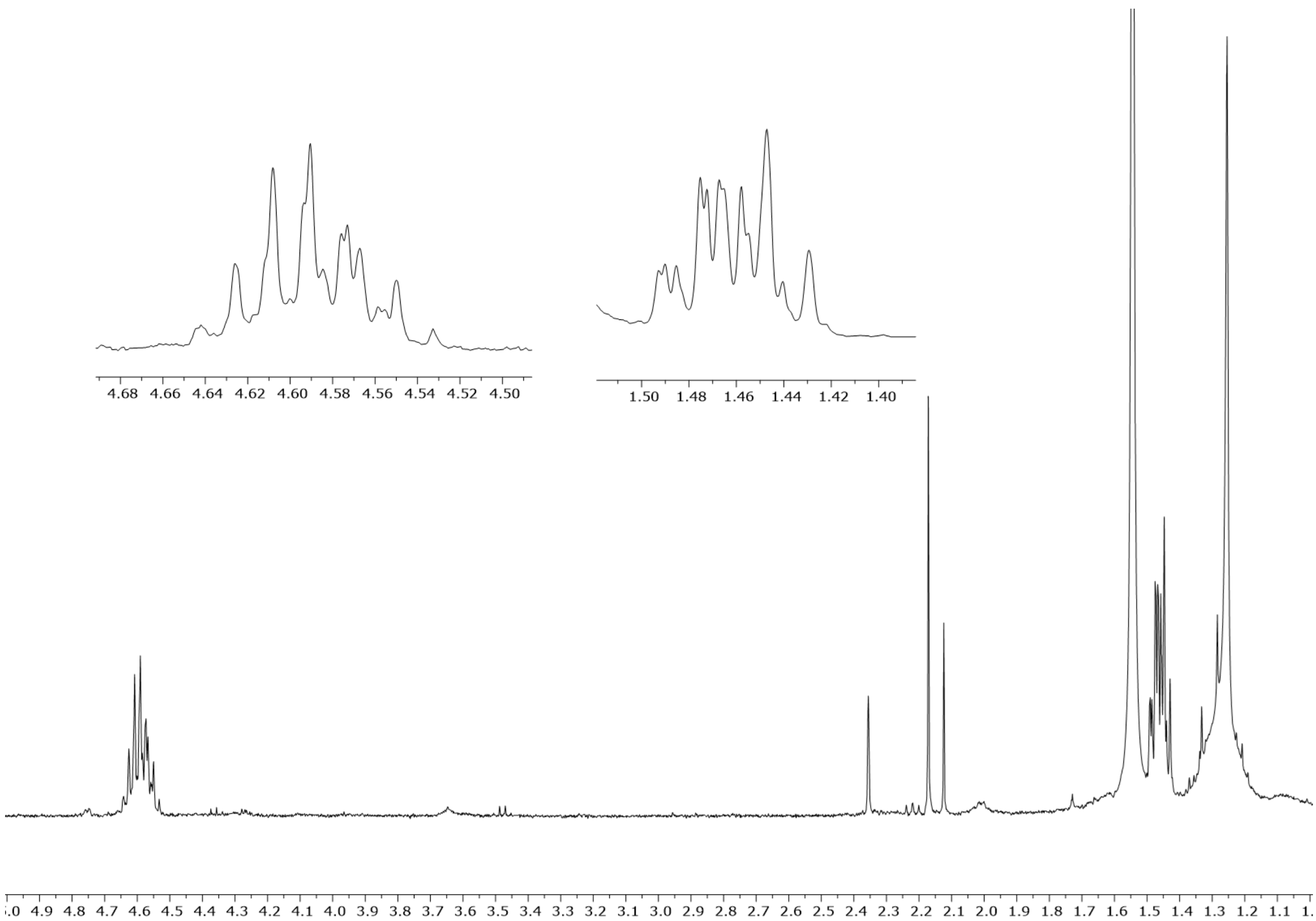

Figure S64. ^{1}H-NMR Spectrum of Fraction (II) of $Lu_3N@I_h$-$C_{80}[C(CO_2Et)_2]_2$. Experiment performed in $CDCl_3$ at 298K (400 MHz).

^{1}H-NMR (400 MHz, $CDCl_3$) δ p.p.m.: 4.58 (m, 4H, -CH_2-), δ 1.46 (m, 6H, -CH_3-).

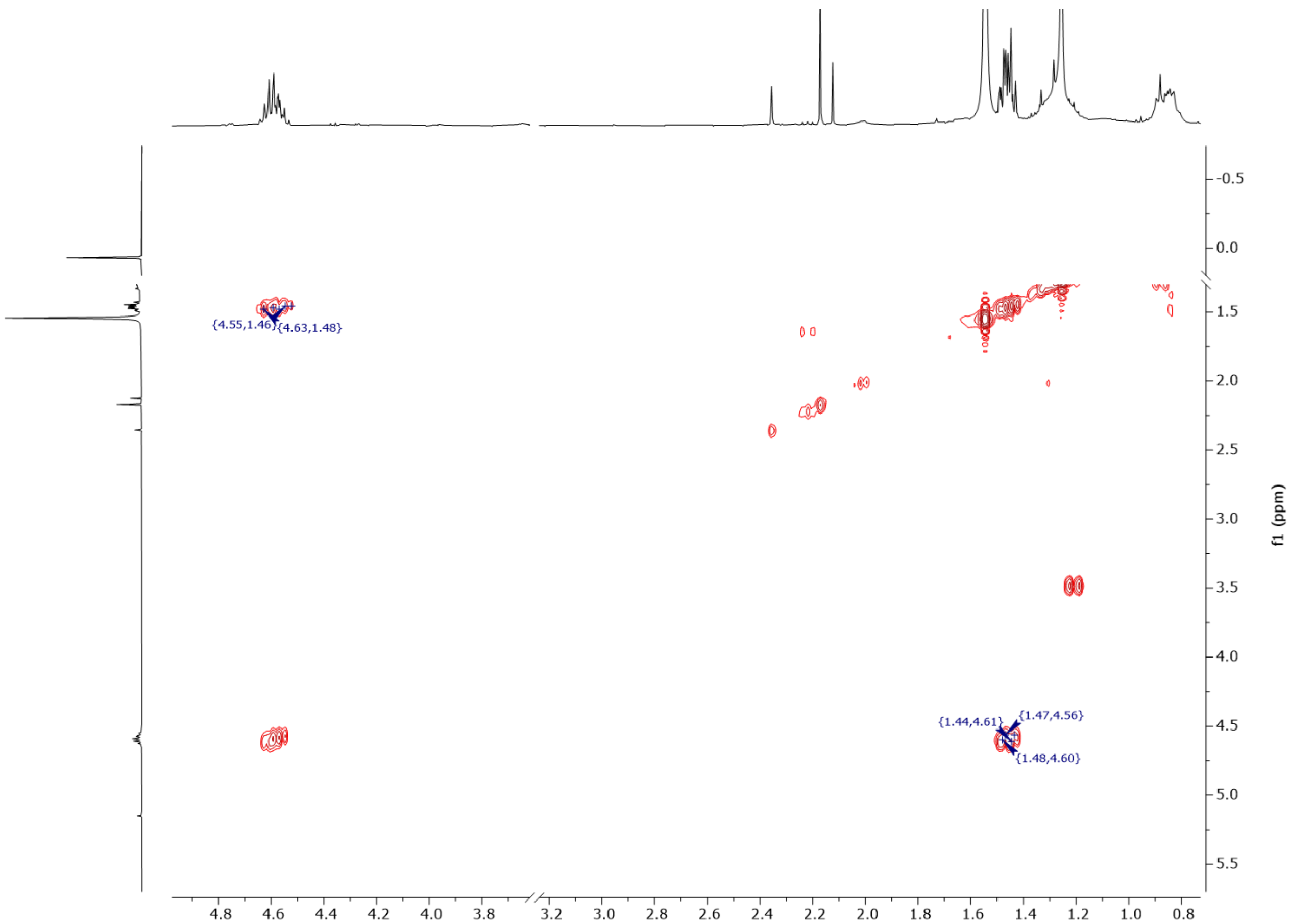

Figure S65. COSY of Fraction (II) of $Lu_3N@I_h\text{-}C_{80}[C(CO_2Et)_2]_2$. Experiment performed in CD_3CN at 298K (400 MHz)

2.3.3. Synthesis and characterization of $Lu_3N@I_h$-$C_{80}[C(CO_2Et)_2][^{13}C(^{13}CO_2Et)_2]\subset$**4**$\cdot(BArF)_8$ via Bingel-Hirsch Reaction

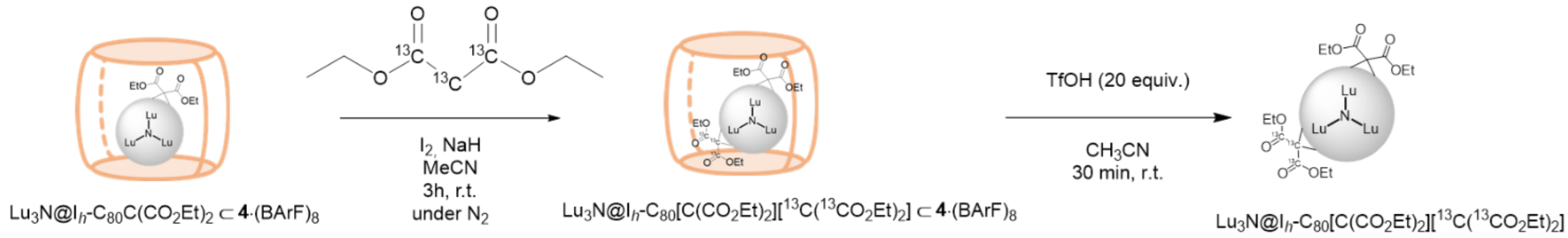

Figure S66. General procedure for Bingel Reaction via supramolecular mask strategy on $Lu_3N@I_h$-$C_{80}[C(CO_2Et)_2]\subset$**4**$\cdot(BArF)_8$ to obtain $Lu_3N@I_h$-$C_{80}[C(CO_2Et)_2][^{13}C(^{13}CO_2Et)_2]\subset$**4**$\cdot(BArF)_8$.

15 mg of $Lu_3N@I_h$-$C_{80}[C(CO_2Et)_2]\subset$**4**$\cdot(BArF)_8$ are totally dissolved in 0.5 mL of MeCN, then 6 equivalents of the diethyl malonate 6 equivalents of I_2 and 6 equivalents of NaH as a base are added sequentially and the reaction is stirred under N_2 at room temperature for 3 hours. Once the $Lu_3N@I_h$-$C_{80}[C(CO_2Et)_2][^{13}C(^{13}CO_2Et)_2]\subset$**4**$\cdot(BArF)_8$ is accumulated as the major product and the $Lu_3N@I_h$-$C_{80}[C(CO_2Et)_2]\subset$**4**$\cdot(BArF)_8$ is consumed (checked by HR-MS), the reaction is stopped by filtering and removing the solvent under reduced pressure.

The crude $Lu_3N@I_h$-$C_{80}[C(CO_2Et)_2][^{13}C(^{13}CO_2Et)_2]\subset$**4**$\cdot(BArF)_8$ reaction mixture is dissolved in CH_3CN (1.5 mL) and an excess of TfOH (20 equivalents) is added to afford the disassembly of the nanocapsule. The mixture is stirred at room temperature during 30 min, then, the solution is dried with N_2 gas flow and $Lu_3N@I_h$-C_{80}-adducts are obtained after Toluene addition and filtration of the solid to remove the broken nanocapsule. Separation of the $Lu_3N@I_h$-$C_{80}[C(CO_2Et)_2][^{13}C(^{13}CO_2Et)_2]$ adducts from the unreacted $Lu_3N@I_h$-$C_{80}C(CO_2Et)_2$ and the undesired $Lu_3N@I_h$-$C_{80}[C(CO_2Et)_2][^{13}C(^{13}CO_2Et)_2]_2$ adducts is obtained by preparative TLC (SiO_2)in Toluene 100%. Two lines Fraction (I) and Fraction (II) corresponding to $Lu_3N@I_h$-$C_{80}[C(CO_2Et)_2][^{13}C(^{13}CO_2Et)_2]$ adducts are observed in the preparative TLC. The fractions are characterized by MALDI-MS, UV-Vis, HPLC, ^{1}H NMR and ^{13}C NMR.

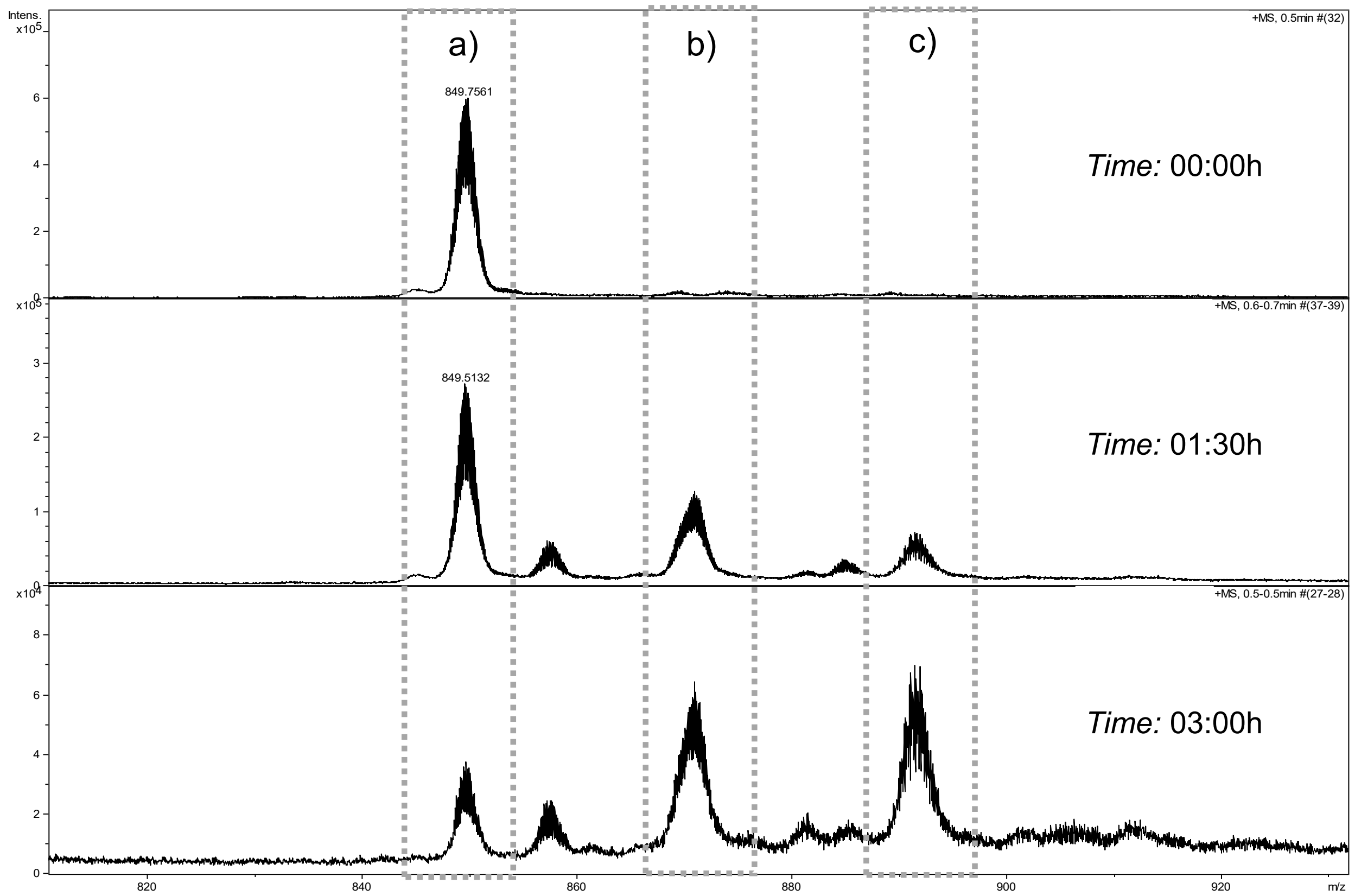

Figure S67. HR-MS monitoring of the $Lu_3N@I_h$-$C_{80}[C(CO_2Et)_2][^{13}C(^{13}CO_2Et)_2] \subset$**4**·$(BArF)_8$ formation upon addition of 6 equivalents of diethyl bromomalonate and 6 equivalents of NaH in 3 hours.

a) Changes in the peak of $Lu_3N@I_h$-$C_{80}[C(CO_2Et)_2]\subset$**4**·$(BArF)_8$
b) Changes in the peak of $Lu_3N@I_h$-$C_{80}[C(CO_2Et)_2][^{13}C(^{13}CO_2Et)_2]\subset$**4**·$(BArF)_8$
c) Changes in the peak of $Lu_3N@I_h$-$C_{80}[C(CO_2Et)_2][^{13}C(^{13}CO_2Et)_2]_2\subset$**4**·$(BArF)_8$

Sample solved in CH_3CN and registered with a Bruker MicroTOF-Q-II mass spectrometer.

NMR Characterization

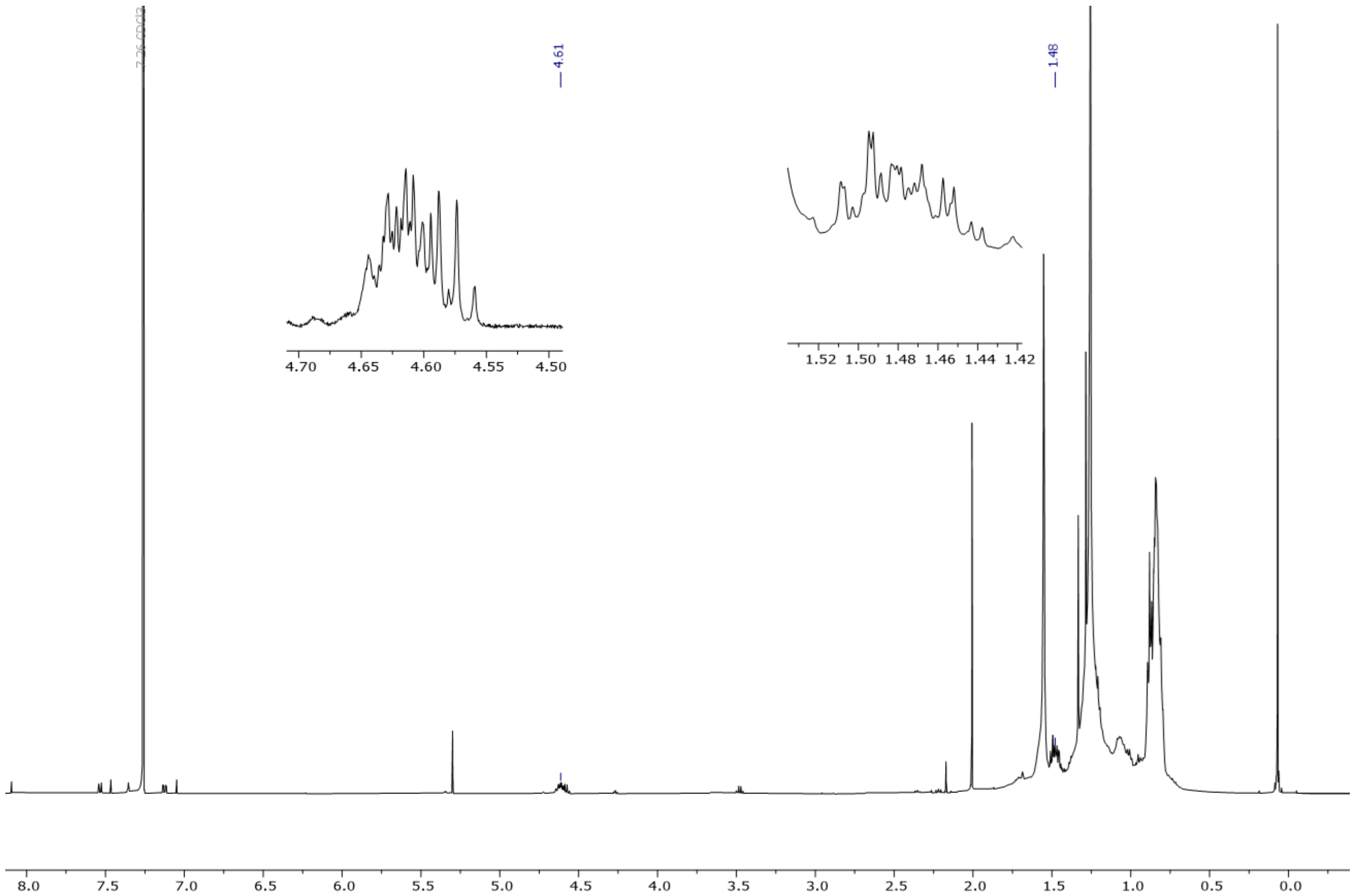

Figure S68.^{1}H-NMR Spectrum of Fraction (I) of $Lu_3N@I_h\text{-}C_{80}[C(CO_2Et)_2][^{13}C(^{13}CO_2Et)_2]$. Experiment performed in $CDCl_3$ at 298K (400 MHz).

^{1}H-NMR (400 MHz, $CDCl_3$) δ p.p.m.: 4.63 (m, 4H, $-CH_2-$), δ 1.49 (m, 6H, $-CH_3-$).

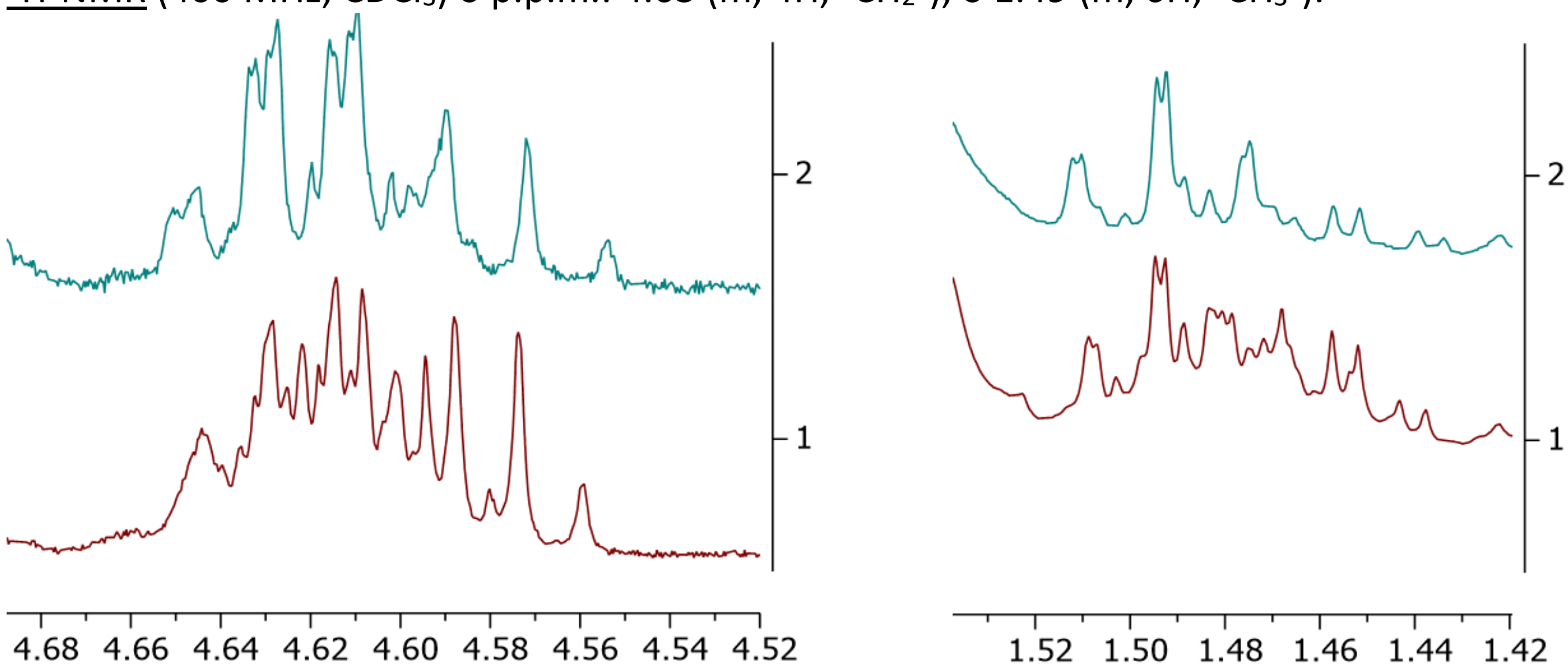

Figure S69. Comparative ^{1}H-NMR spectra ($CDCl_3$, 400 MHz, 298 K) of $Lu_3N@I_h\text{-}C_{80}[C(CO_2Et)_2]_2$ Fraction (I) obtained by Bingel reaction (top) and $Lu_3N@I_h\text{-}C_{80}[C(CO_2Et)_2][^{13}C(^{13}CO_2Et)_2]$ Fraction (I) obtained by Bingel-Hirsh reaction with labelled diethyl malonate (down).

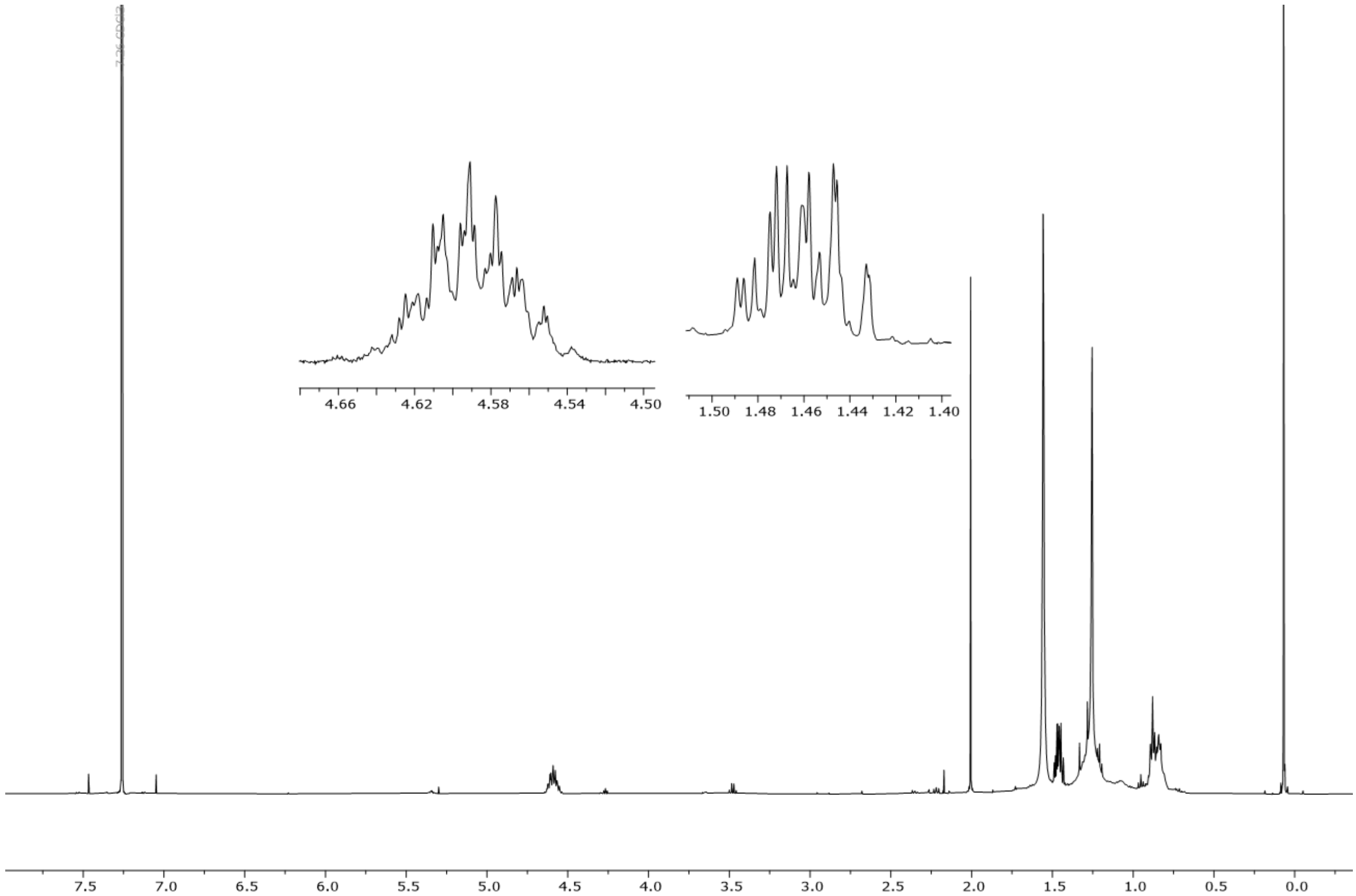

Figure S51. ^{1}H-NMR Spectrum of Fraction (II) of $Lu_3N@I_h\text{-}C_{80}[C(CO_2Et)_2][^{13}C(^{13}CO_2Et)_2]$. Experiment performed in $CDCl_3$ at 298K (400 MHz).

^{1}H-NMR (400 MHz, $CDCl_3$) δ p.p.m.: 4.58 (m, 4H, $-CH_2-$), δ 1.46 (m, 6H, $-CH_3-$).

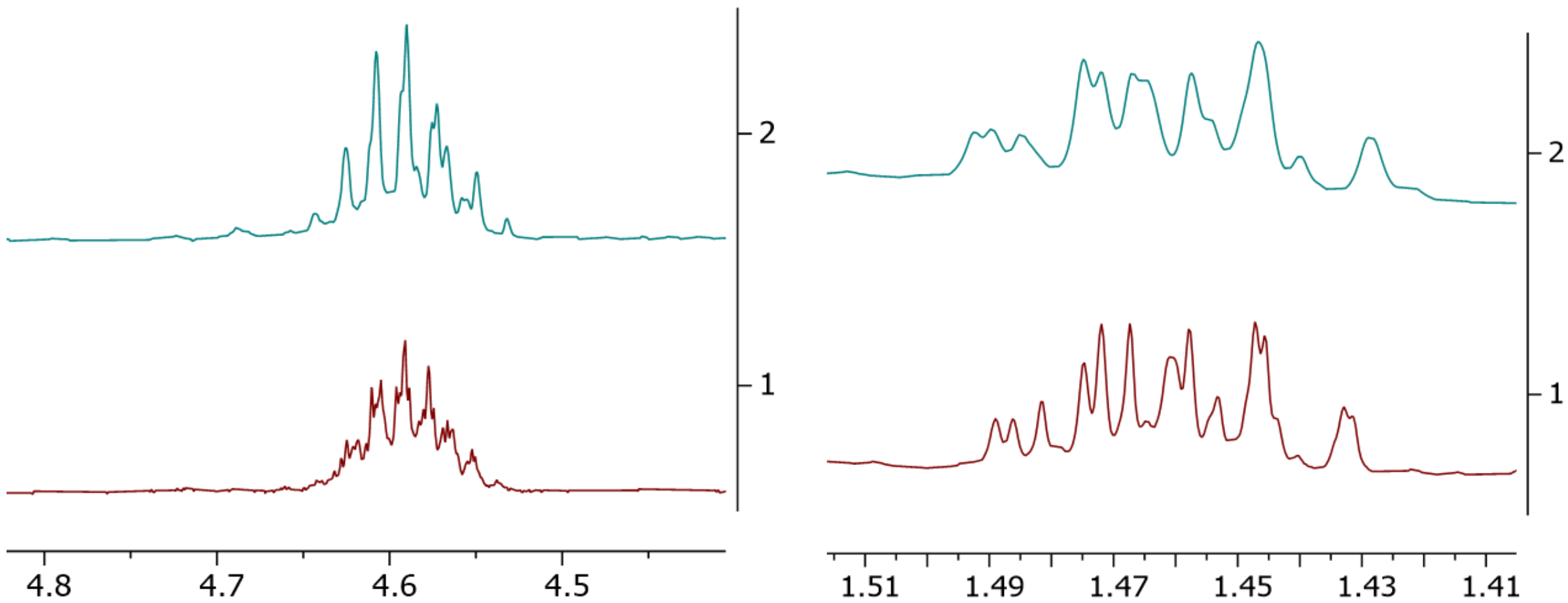

Figure S71. Comparative ^{1}H-NMR spectra ($CDCl_3$, 400 MHz, 298 K) of $Lu_3N@I_h\text{-}C_{80}[C(CO_2Et)_2]_2$ Fraction (I) obtained by Bingel reaction (top) and $Lu_3N@I_h\text{-}C_{80}[C(CO_2Et)_2][^{13}C(^{13}CO_2Et)_2]$ Fraction (I) obtained by Bingel-Hirsh reaction with labelled diethyl malonate (down).

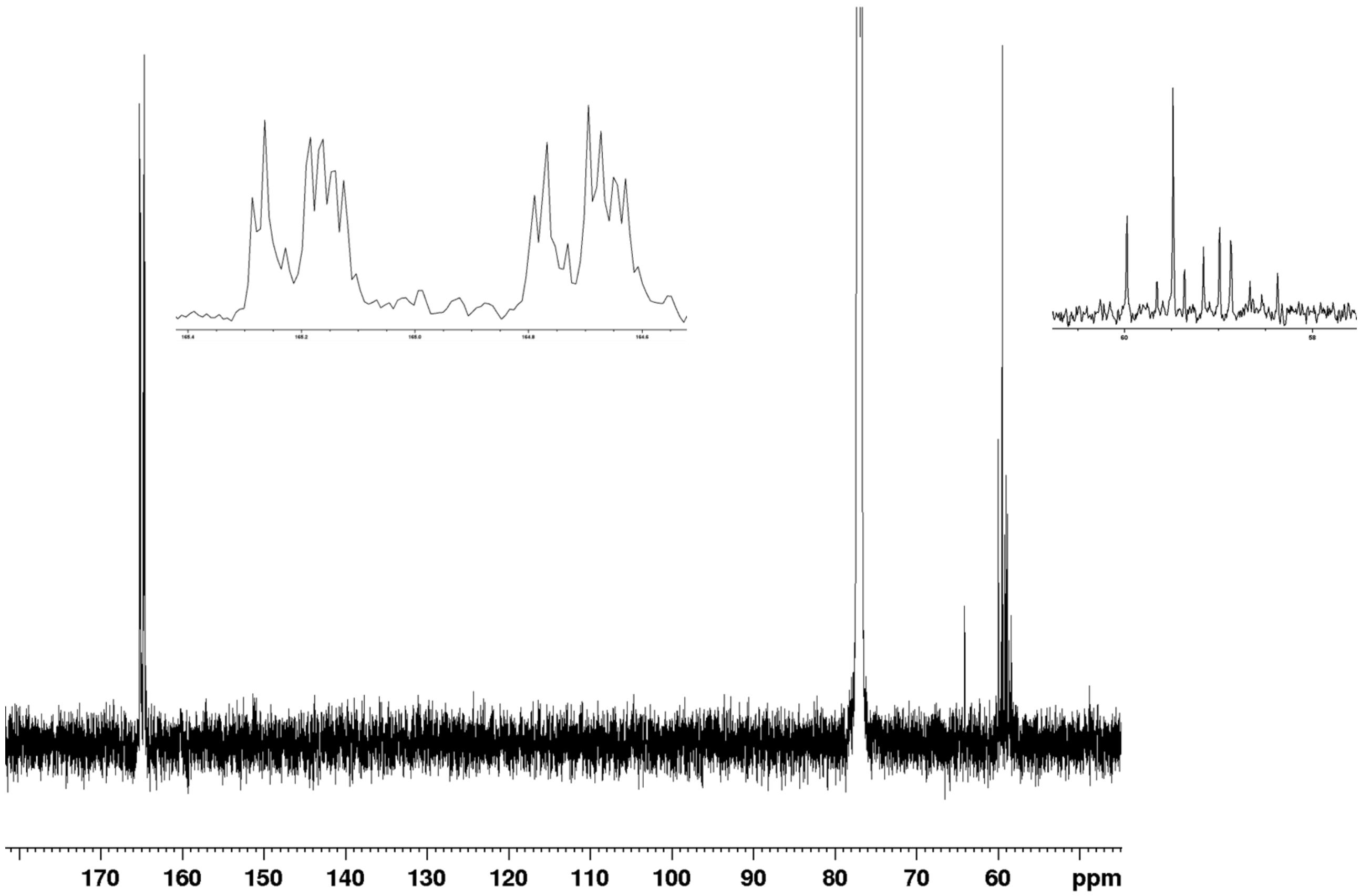

Figure S72. ^{13}C-NMR Spectrum of $Lu_3N@I_h$-$C_{80}[C(CO_2Et)_2][^{13}C(^{13}CO_2Et)_2]$ Fraction (I). Experiment performed in $CDCl_3$ at 298K (100 MHz).

^{13}C-NMR (100 MHz, $CDCl_3$) δ p.p.m.: 165.20 (^{13}C=O, m), 164.71 (^{13}C=O, m), 59.48 (-C-^{13}C-C-, t), 58.99 (-C-^{13}C-C-, t), 58.87 (-C-^{13}C-C-, t).

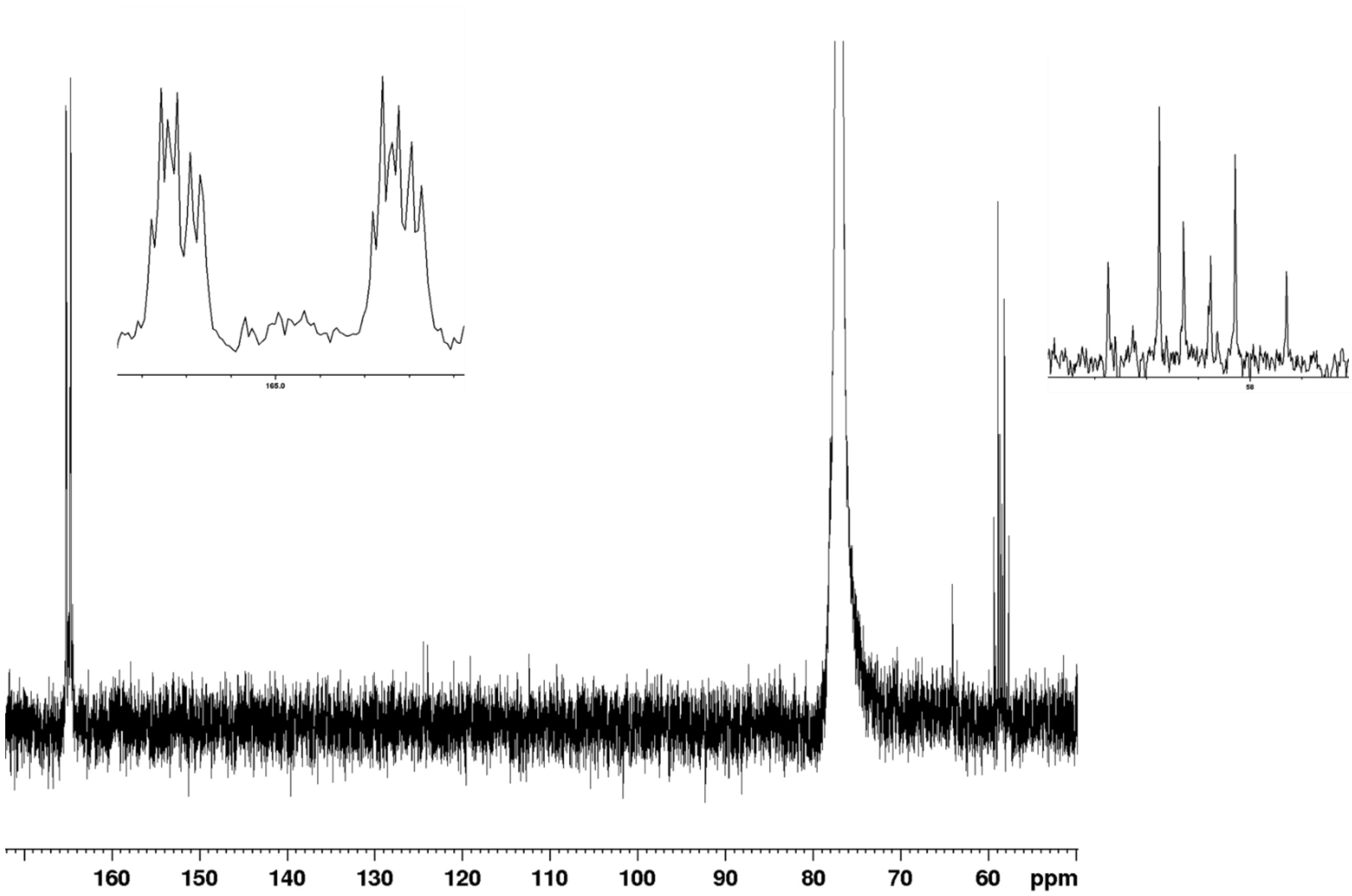

Figure S73. ^{13}C-NMR Spectrum of $Lu_3N@I_h$-$C_{80}[C(CO_2Et)_2][^{13}C(^{13}CO_2Et)_2]$ Fraction (II). Experiment performed in $CDCl_3$ at 298K (100 MHz).

^{13}C-NMR (100 MHz, $CDCl_3$) δ p.p.m.: 165.22 (^{13}C=O, m), 164.72 (^{13}C=O, m), 58.87 (-C-^{13}C-C-, t), 58.15 (-C-^{13}C-C-, t).

2.3.4. Synthesis and characterization of $Sc_3N@I_h$-$C_{80}[C(CO_2Et)_2][^{13}C(^{13}CO_2Et)_2]\subset$**4**$\cdot(BArF)_8$ via Bingel-Hirsch Reaction

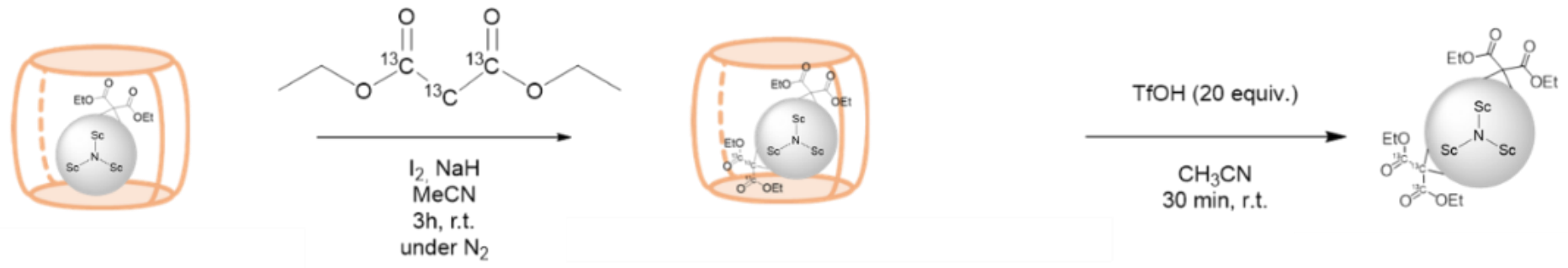

Figure S74. General procedure for Bingel Reaction via supramolecular mask strategy on $Sc_3N@I_h$-$C_{80}[C(CO_2Et)_2]\subset$**4**$\cdot(BArF)_8$ to obtain $Lu_3N@I_h$-$C_{80}[C(CO_2Et)_2][^{13}C(^{13}CO_2Et)_2]\subset$**4**$\cdot(BArF)_8$.

15 mg of $Sc_3N@I_h$-$C_{80}[C(CO_2Et)_2]\subset$**4**$\cdot(BArF)_8$ are totally dissolved in 0.5 mL of MeCN, then 1.5 equivalents of the diethyl malonate 1.5 equivalents of I_2 and 1.5 equivalents of NaH as a base are added sequentially and the reaction is stirred under N_2 at room temperature for 1 hour. Once the $Sc_3N@I_h$-$C_{80}[C(CO_2Et)_2][^{13}C(^{13}CO_2Et)_2]\subset$**4**$\cdot(BArF)_8$ is accumulated as the major product and the $Sc_3N@I_h$-$C_{80}[C(CO_2Et)_2]\subset$**4**$\cdot(BArF)_8$ is consumed (checked by HR-MS), the reaction is stopped by filtering and removing the solvent under reduced pressure.

The crude $Sc_3N@I_h$-$C_{80}[C(CO_2Et)_2][^{13}C(^{13}CO_2Et)_2]\subset$**4**$\cdot(BArF)_8$ reaction mixture is dissolved in CH_3CN (1.5 mL) and an excess of TfOH (20 equivalents) is added to afford the disassembly of the nanocapsule. The mixture is stirred at room temperature during 30 min, then, the solution is dried with N_2 gas flow and $Lu_3N@I_h$-C_{80}-adducts are obtained after Toluene addition and filtration of the solid to remove the broken nanocapsule. Separation of the $Sc_3N@I_h$-$C_{80}[C(CO_2Et)_2][^{13}C(^{13}CO_2Et)_2]$ adducts from the unreacted $Sc_3N@I_h$-$C_{80}C(CO_2Et)_2$ and the undesired $Sc_3N@I_h$-$C_{80}[C(CO_2Et)_2][^{13}C(^{13}CO_2Et)_2]_2$ adducts and multi-adducts is obtained by preparative TLC (SiO_2)in Toluene 100%. Two lines Fraction (I) and Fraction (II) corresponding to $Sc_3N@I_h$-$C_{80}[C(CO_2Et)_2][^{13}C(^{13}CO_2Et)_2]$ adducts are observed in the preparative TLC.

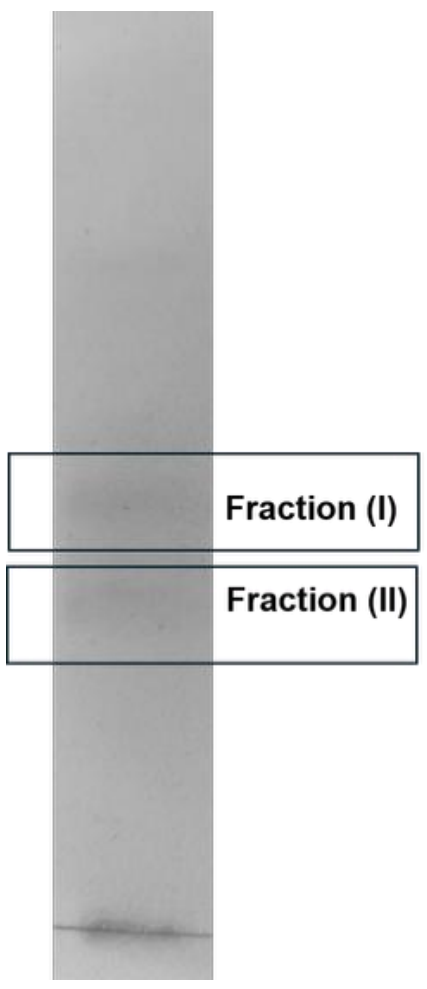

Figure S75. Preparative TLC of $Sc_3N@I_h$-$C_{80}[C(CO_2Et)_2]_2$ after breaking the nanocapsule (SiO_2, Toluene).
$Sc_3N@I_h$-$C_{80}[C(CO_2Et)_2]_2$ Fraction (I)
$Sc_3N@I_h$-$C_{80}[C(CO_2Et)_2]_2$ Fraction (II)

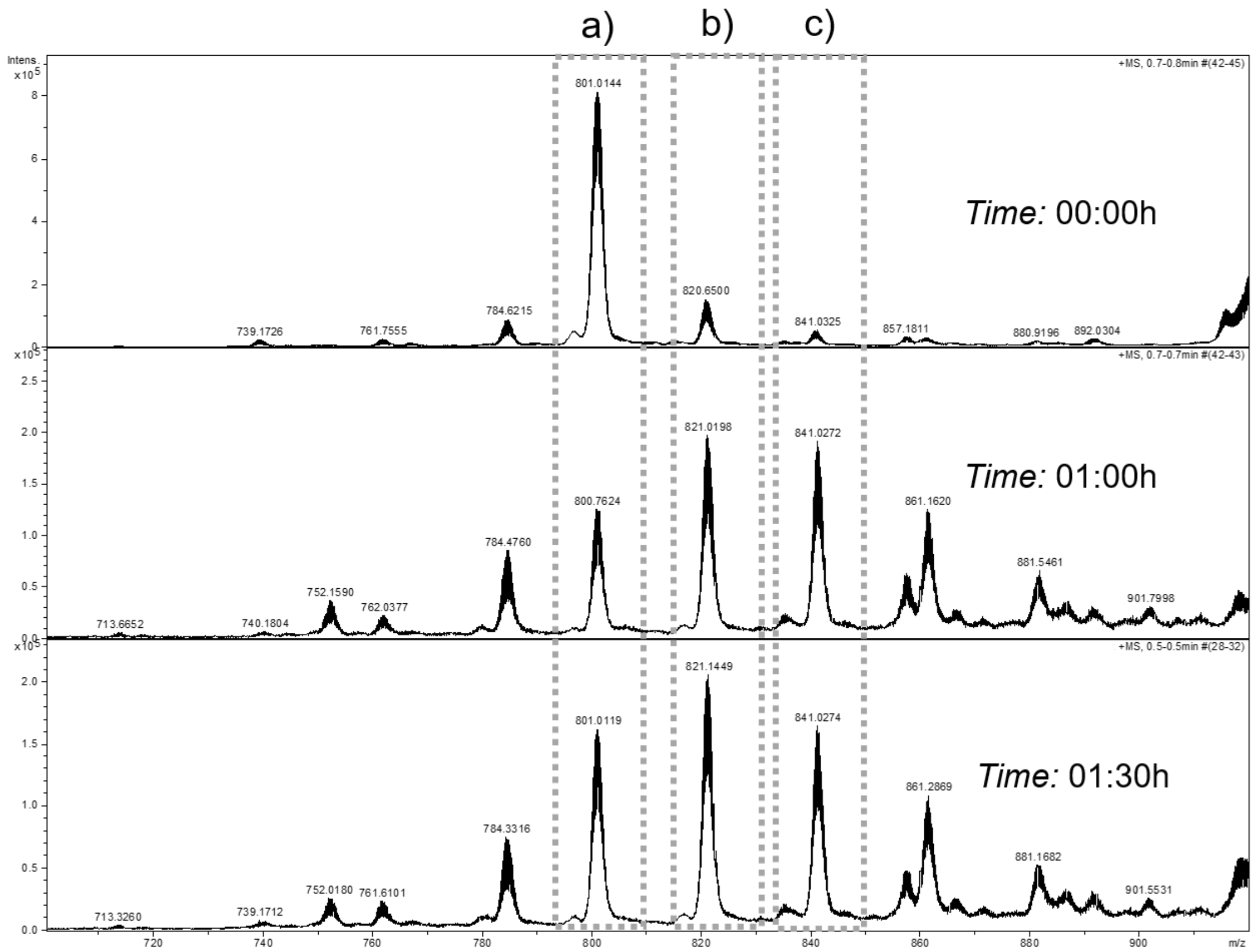

Figure S76. HR-MS monitoring of the $Sc_3N@I_h$-$C_{80}[C(CO_2Et)_2][^{13}C(^{13}CO_2Et)_2] \subset$**4**·$(BArF)_8$ formation upon addition of 1.5 equivalents of diethyl bromomalonate and 1.5 equivalents of NaH in 1 hour.

a) Changes in the peak of $Sc_3N@I_h$-$C_{80}[C(CO_2Et)_2]\subset$**4**·$(BArF)_8$
b) Changes in the peak of $Sc_3N@I_h$-$C_{80}[C(CO_2Et)_2][^{13}C(^{13}CO_2Et)_2]\subset$**4**·$(BArF)_8$
c) Changes in the peak of $Sc_3N@I_h$-$C_{80}[C(CO_2Et)_2][^{13}C(^{13}CO_2Et)_2]_2\subset$**4**·$(BArF)_8$

Sample solved in CH_3CN and registered with a Bruker MicroTOF-Q-II mass spectrometer.

2.4. Control experiments with labelled malonate

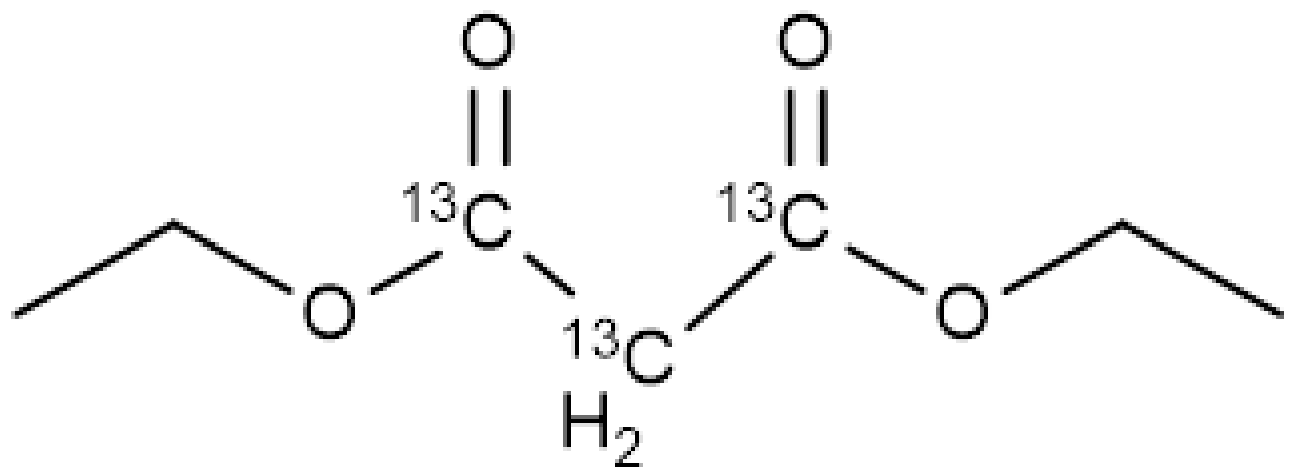

Figure S77. Diethyl malonate-1,2,3-$^{13}C_3$.

To have a better estimation of how many isomers we have in each Fraction we decided to perform some experiments with a labelled malonate. To obtain a cyclopropanated adduct of $Lu_3N@I_h$-C_{80} comparable with the corresponding adduct obtained upon Bingel reaction, we decided to perform a Bingel-Hirsch Reaction[23] using the same supramolecular Mask strategy with **4**·$(BArF)_8$ nanocapsule assuming to obtain comparable adducts differing only in the isotope of 1,2,3-C of the functionalization.

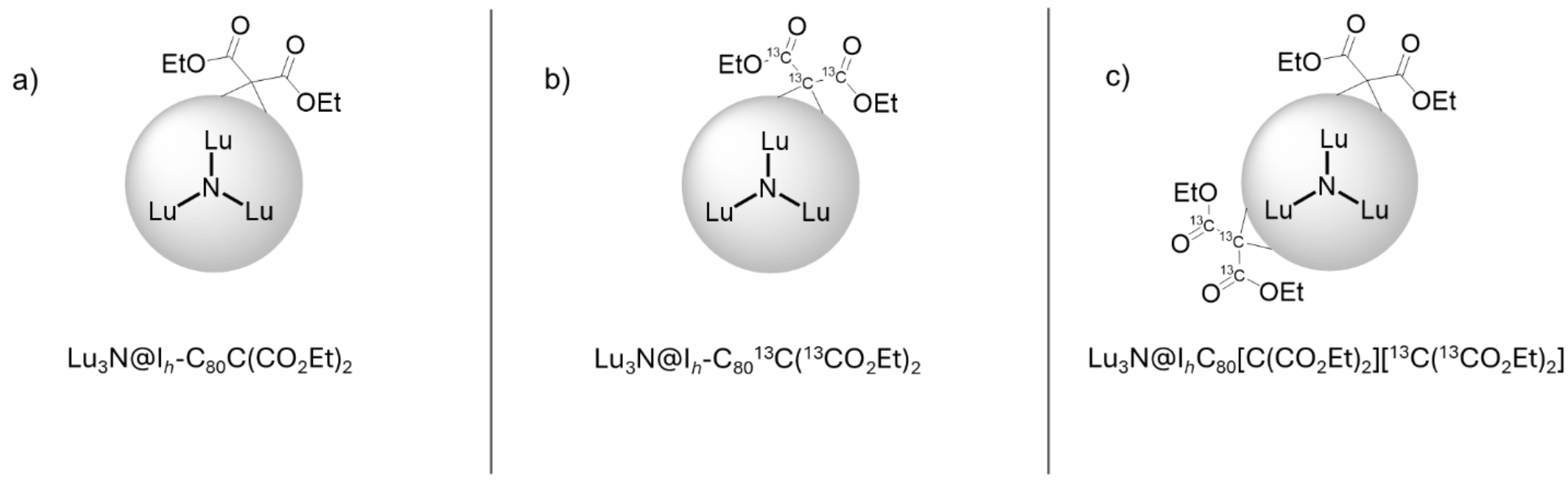

Figure S78. Schematic representation used in the manuscript and the SI for some of the Bingel and Bingel-Hirsch adducts.

a) $Lu_3N@I_h$-$C_{80}C(CO_2Et)_2$

b) $Lu_3N@I_h$-$C_{80}{}^{13}C(^{13}CO_2Et)_2$

c) $Lu_3N@I_h$-$C_{80}[C(CO_2Et)_2][^{13}C(^{13}CO_2Et)_2]$

2.4.1. Synthesis and characterization of $Lu_3N@I_h\text{-}C_{80}C(CO_2Et)_2 \subset \mathbf{4} \cdot (BArF)_8$ via Bingel-Hirsch Reaction

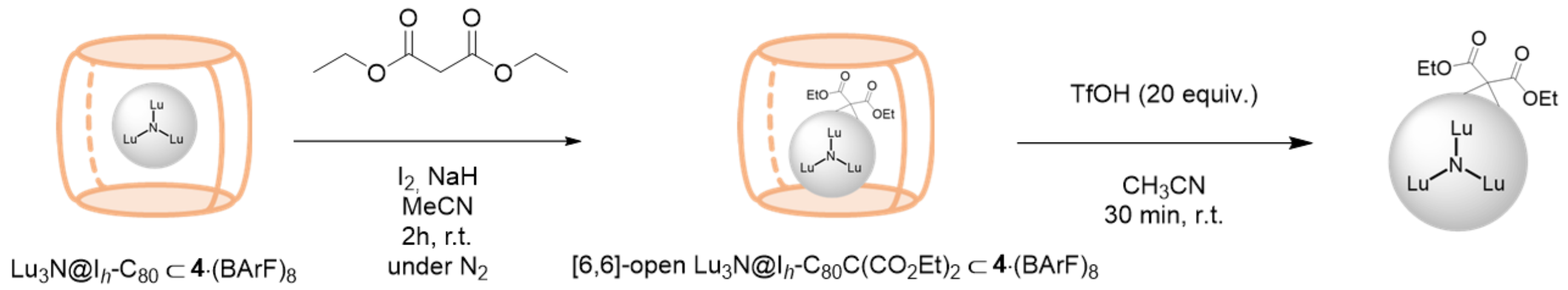

Figure S79. General procedure for Bingel-Hirsch Reaction via supramolecular mask strategy on $Lu_3N@I_h\text{-}C_{80} \subset \mathbf{4} \cdot (BArF)_8$. Experiment done to confirm that the products obtained by Bingel- Hirsch reaction (using Iodine, NaH and Diethyl malonate) are comparable to the products obtained by Bingel reaction (using NaH and Diethyl Bromo Malonate)

15 mg of $Lu_3N@I_h\text{-}C_{80} \subset \mathbf{4} \cdot (BArF)_8$ are totally dissolved in 0.5 mL of MeCN, then 4 equivalents of the diethyl malonate, 4 equivalents of Iodine and 4 equivalents of NaH as a base are added sequentially and the reaction is stirred under N_2 at room temperature for 2 hours. Once the $Lu_3N@I_h\text{-}C_{80} \subset \mathbf{4} \cdot (BArF)_8$ is completely consumed and the $Lu_3N@I_h\text{-}C_{80}C(CO_2Et)_2$ is formed (checked by HR-MS), the reaction is stopped by filtering and removing the solvent under reduced pressure.

The crude $Lu_3N@I_h\text{-}C_{80}C(CO_2Et)_2 \subset \mathbf{4} \cdot (BArF)_8$ reaction mixture is dissolved in CH_3CN (1.5 mL) and an excess of TfOH (20 equivalents) is added to afford the disassembly of the nanocapsule. The mixture is stirred at room temperature during 30 min, then, the solution is dried with N_2 gas flow and $Lu_3N@I_h\text{-}C_{80}C(CO_2Et)_2$ is obtained after Toluene addition and filtration of the solid to remove the broken nanocapsule. Further purification of $Lu_3N@I_h\text{-}C_{80}C(CO_2Et)_2$ can be obtained by preparative TLC in Toluene 100%. $Lu_3N@I_h\text{-}C_{80}C(CO_2Et)_2$ has been characterized by ^{1}H-NMR to confirm the identity in comparison to Mono-*Et*-$Lu_3N@I_h\text{-}C_{80}$ obtained by Bingel reaction.

NMR Characterization

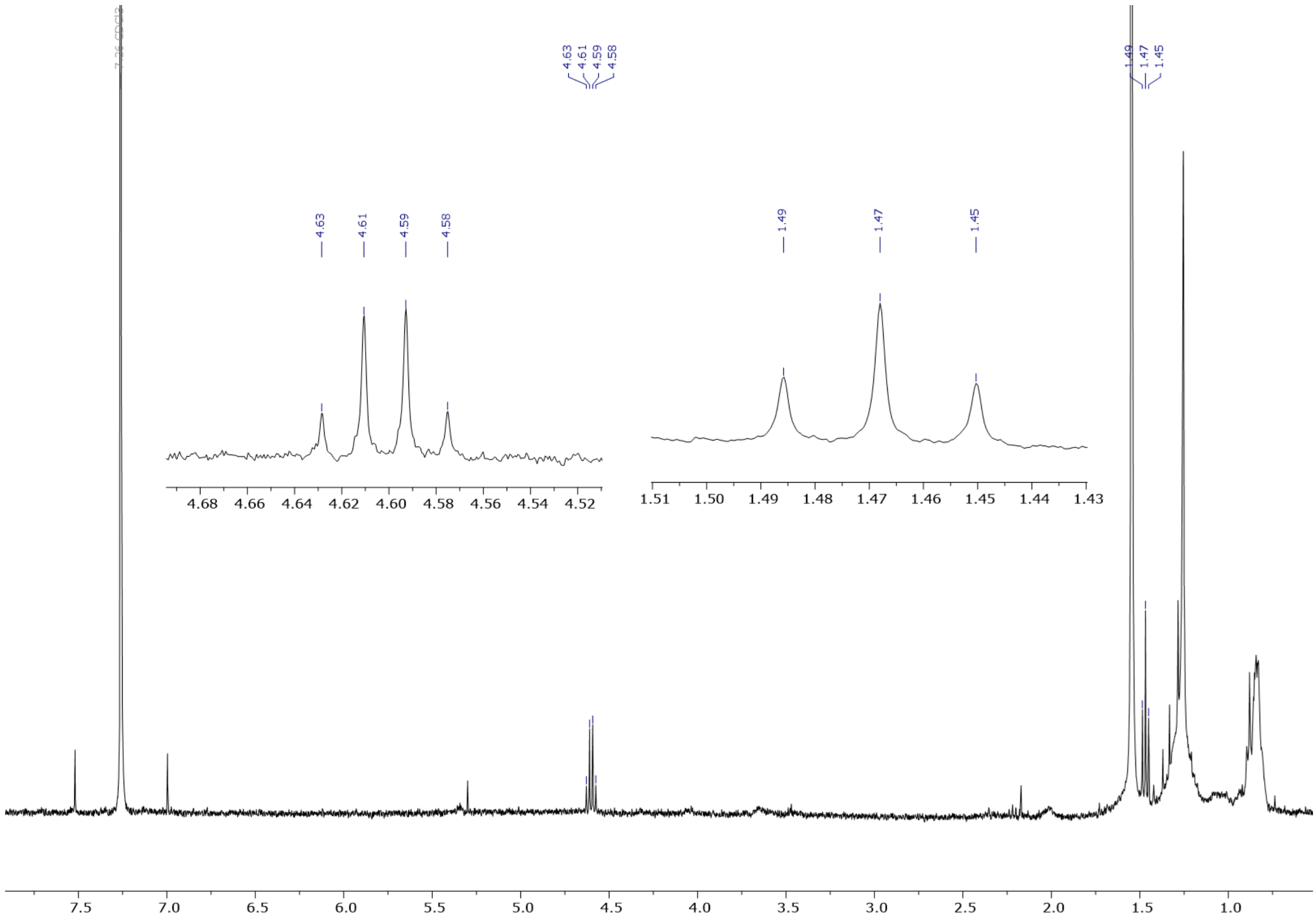

Figure S80. ^{1}H-NMR Spectrum of $Lu_3N@I_h\text{-}C_{80}C(CO_2Et)_2$ obtained by Bingel-Hirsh reaction. Experiment performed in $CDCl_3$ at 298K (400 MHz).

^{1}H-NMR (400 MHz, $CDCl_3$) δ p.p.m.: 4.60 (q, *J*=7.1 Hz, 4H, $-CH_2-$), δ 1.47 (t, *J*=7.1 Hz, 6H, $-CH_3-$).

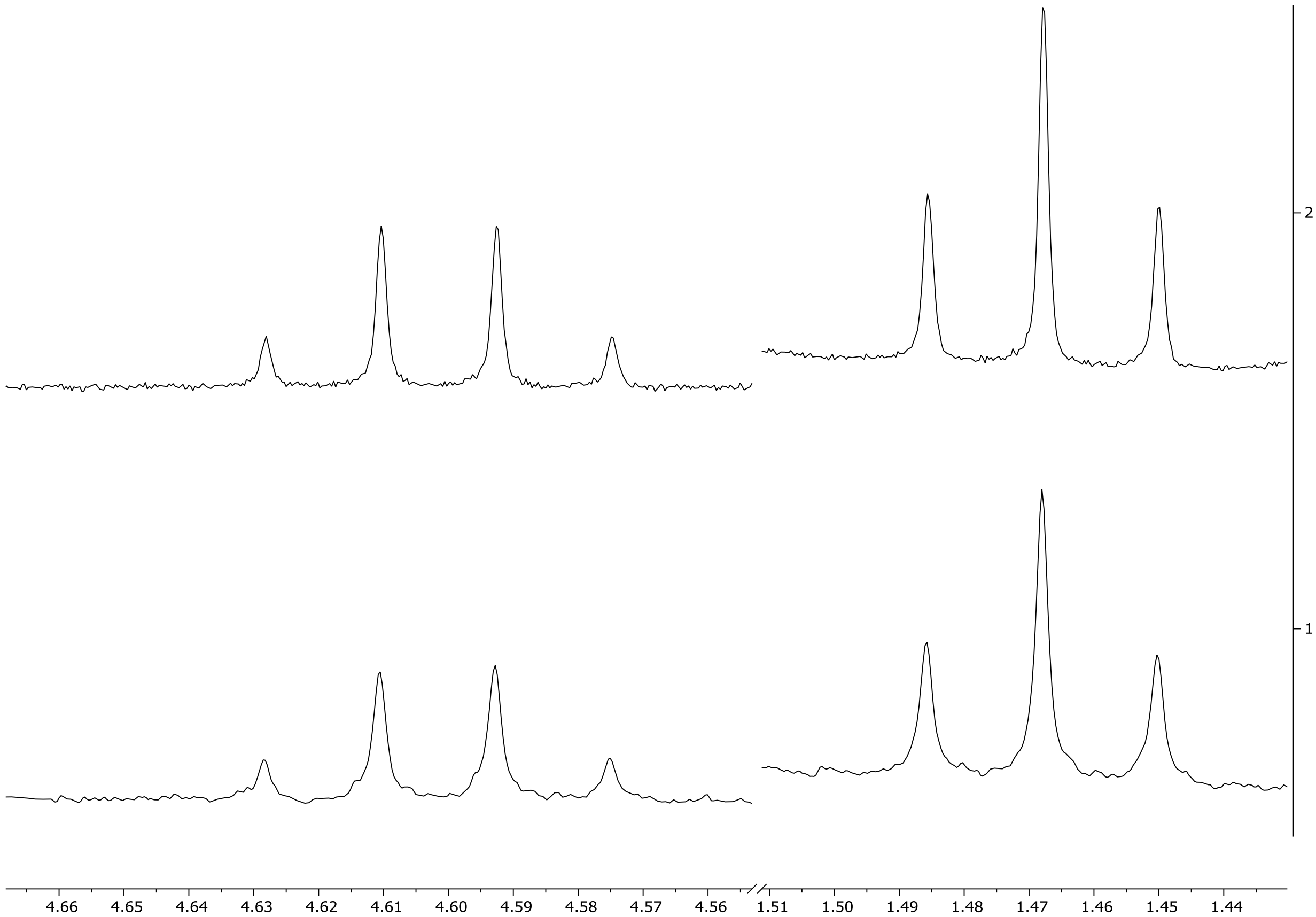

Figure S81. Comparative ^{1}H-NMR spectra ($CDCl_3$, 400 MHz, 298 K) of $Lu_3N@I_h\text{-}C_{80}C(CO_2Et)_2$ obtained by Bingel reaction (top) and Bingel-Hirsh reaction (down).

2.4.2. Synthesis and characterization of $Lu_3N@I_h$-$C_{80}[^{13}C(^{13}CO_2Et)_2]\subset$4·(BArF)$_8$ via Bingel-Hirsch Reaction

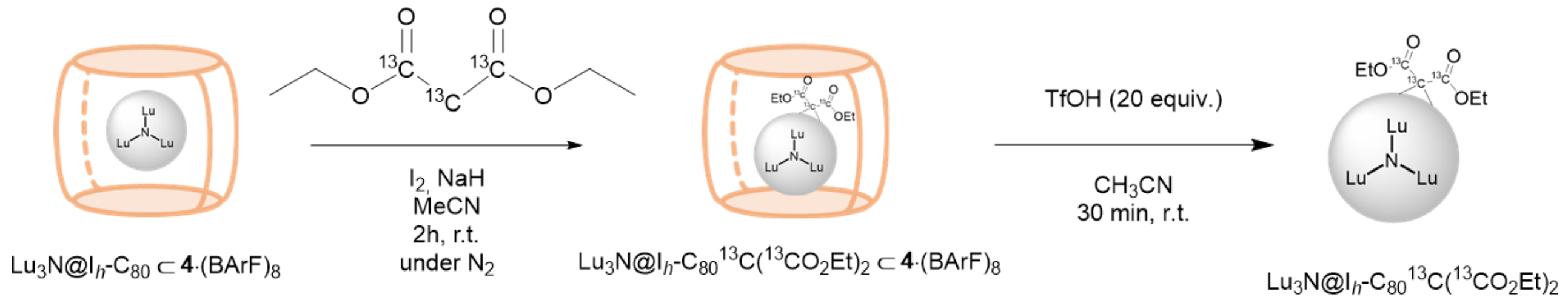

Figure S82. General procedure for Bingel-Hirsch Reaction via supramolecular mask strategy on $Lu_3N@I_h$-$C_{80}\subset$**4**·(BArF)$_8$ using a labelled malonate.

15 mg of $Lu_3N@I_h$-$C_{80}\subset$**4**·(BArF)$_8$ are totally dissolved in 0.5 mL of MeCN, then 4 equivalents of the labelled diethyl malonate, 4 equivalents of iodine and 4 equivalents of NaH as a base are added sequentially and the reaction is stirred under N_2 at room temperature for 2 hours. Once the $Lu_3N@I_h$-$C_{80}\subset$**4**·(BArF)$_8$ is completely consumed and the $Lu_3N@I_h$-$C_{80}[^{13}C(^{13}CO_2Et)_2]\subset$**4**·(BArF)$_8$ is formed (checked by HR-MS), the reaction is stopped by filtering and removing the solvent under reduced pressure.

The crude $Lu_3N@I_h$-$C_{80}{}^{13}C(^{13}CO_2Et)_2\subset$**4**·(BArF)$_8$ reaction mixture is dissolved in CH_3CN (1.5 mL) and an excess of TfOH (20 equivalents) is added to afford the disassembly of the nanocapsule. The mixture is stirred at room temperature during 30 min, then, the solution is dried with N_2 gas flow and $Lu_3N@I_h$-$C_{80}{}^{13}C(^{13}CO_2Et)_2$ is obtained after Toluene addition and filtration of the solid to remove the broken nanocapsule. Further purification of $Lu_3N@I_h$-$C_{80}{}^{13}C(^{13}CO_2Et)_2$ can be obtained by preparative TLC in Toluene 100%. $Lu_3N@I_h$-$C_{80}{}^{13}C(^{13}CO_2Et)_2$ has been characterized by ^{1}H-NMR and ^{13}C NMR.

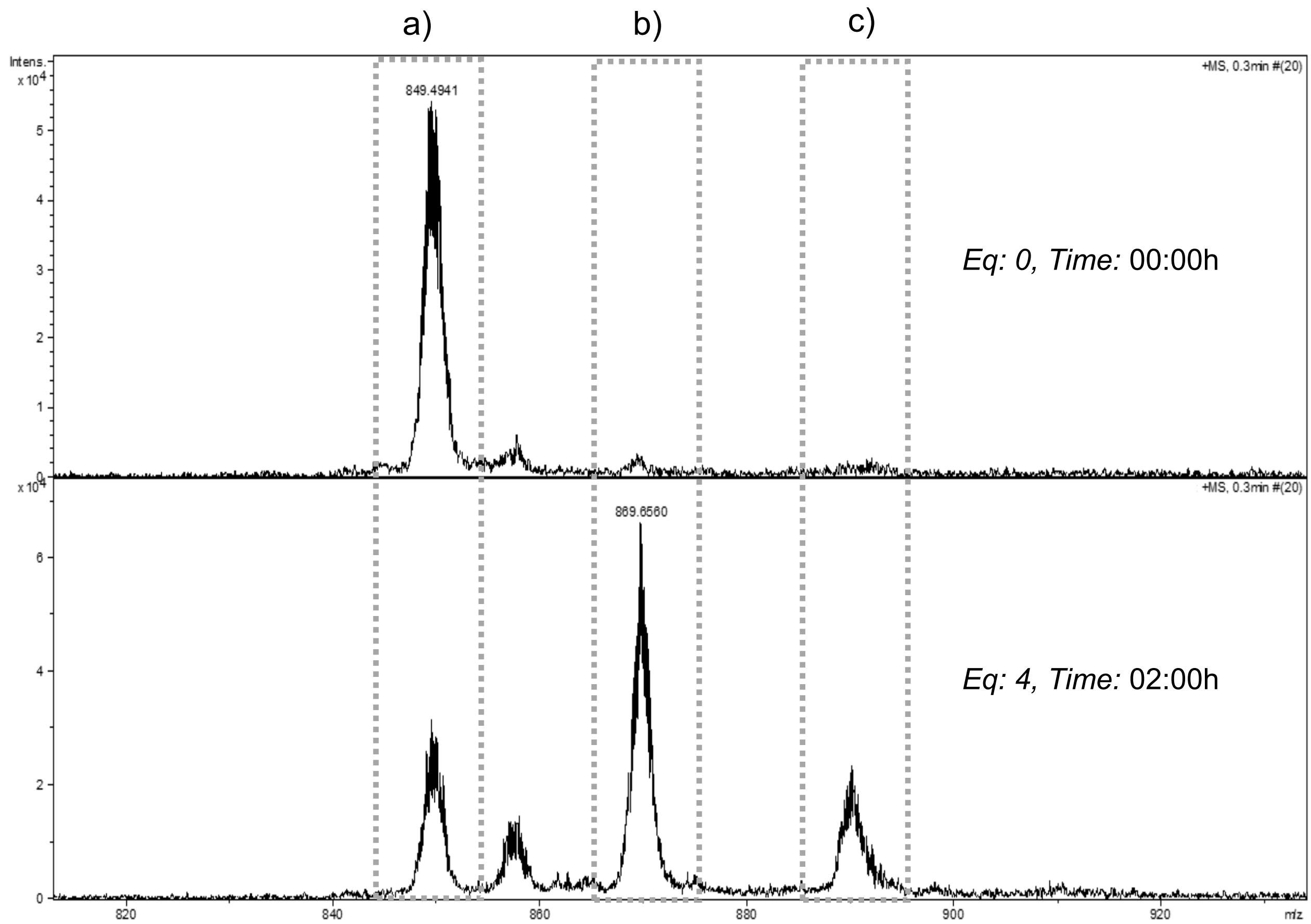

Figure S83. HR-MS monitoring of the $Lu_3N@I_h$-$C_{80}{}^{13}C({}^{13}CO_2Et)_2$⊂**4**·$(BArF)_8$ formation upon addition of 4 equivalents of diethyl malonate, I_2 and NaH in 2 hours.

a) Changes in the peak of $Lu_3N@I_h$-$C_{80}[C(CO_2Et)_2]$⊂**4**·$(BArF)_8$
b) Changes in the peak of $Lu_3N@I_h$-$C_{80}[C(CO_2Et)_2][{}^{13}C({}^{13}CO_2Et)_2]$⊂**4**·$(BArF)_8$
c) Changes in the peak of $Lu_3N@I_h$-$C_{80}[C(CO_2Et)_2][{}^{13}C({}^{13}CO_2Et)_2]_2$⊂**4**·$(BArF)_8$

Sample solved in CH_3CN and registered with a Bruker MicroTOF-Q-II mass spectrometer.

NMR Characterization

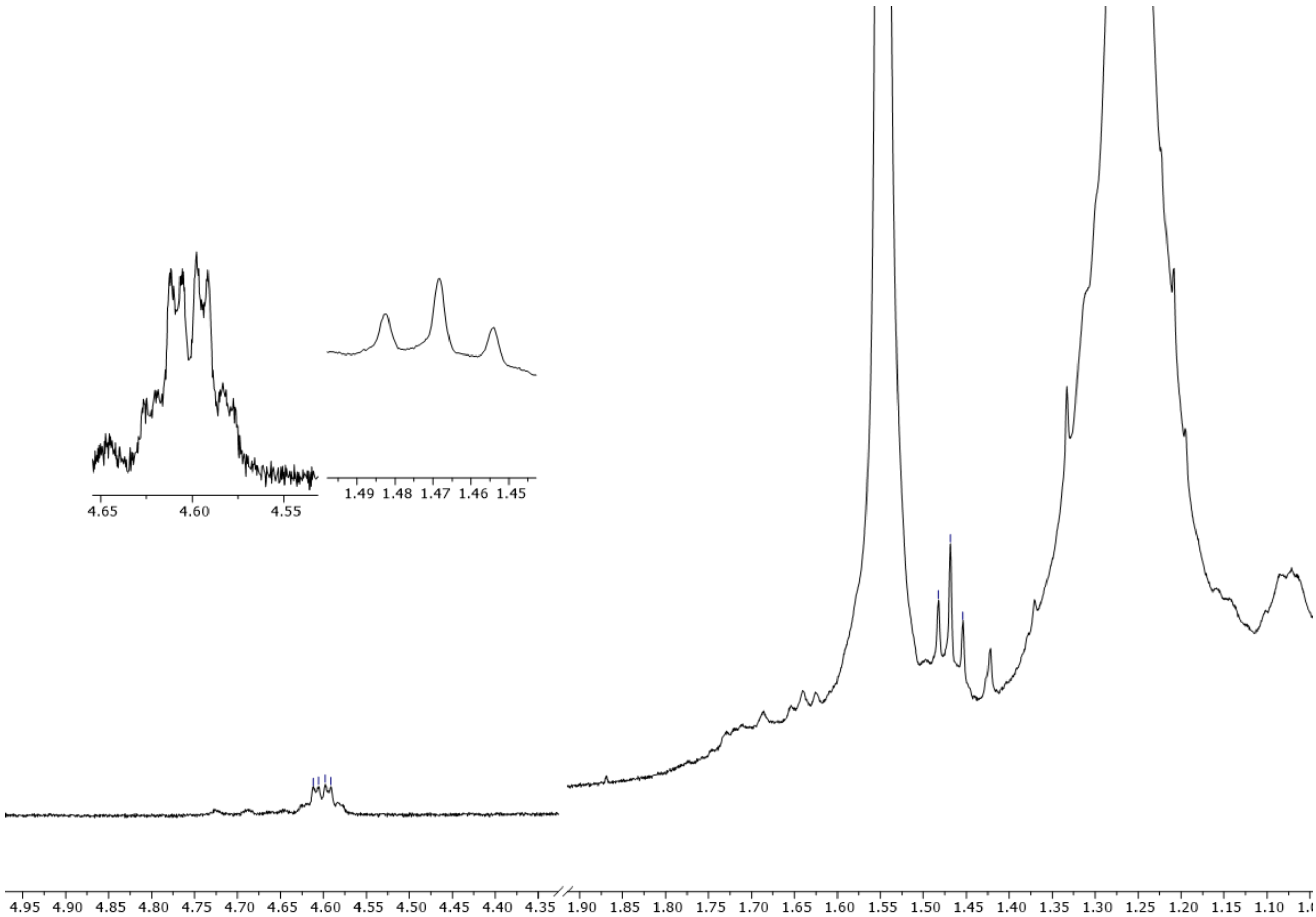

Figure S84. ^{1}H-NMR Spectrum of $Lu_3N@I_h$-$C_{80}{}^{13}C(^{13}CO_2Et)_2$ obtained by Bingel-Hirsh reaction. Experiment performed in $CDCl_3$ at 298K (400 MHz).

^{1}H-NMR (400 MHz, $CDCl_3$) δ p.p.m.: 4.60 (qd, 4H, $-CH_2-$), δ 1.47 (t, 6H, $-CH_3-$).

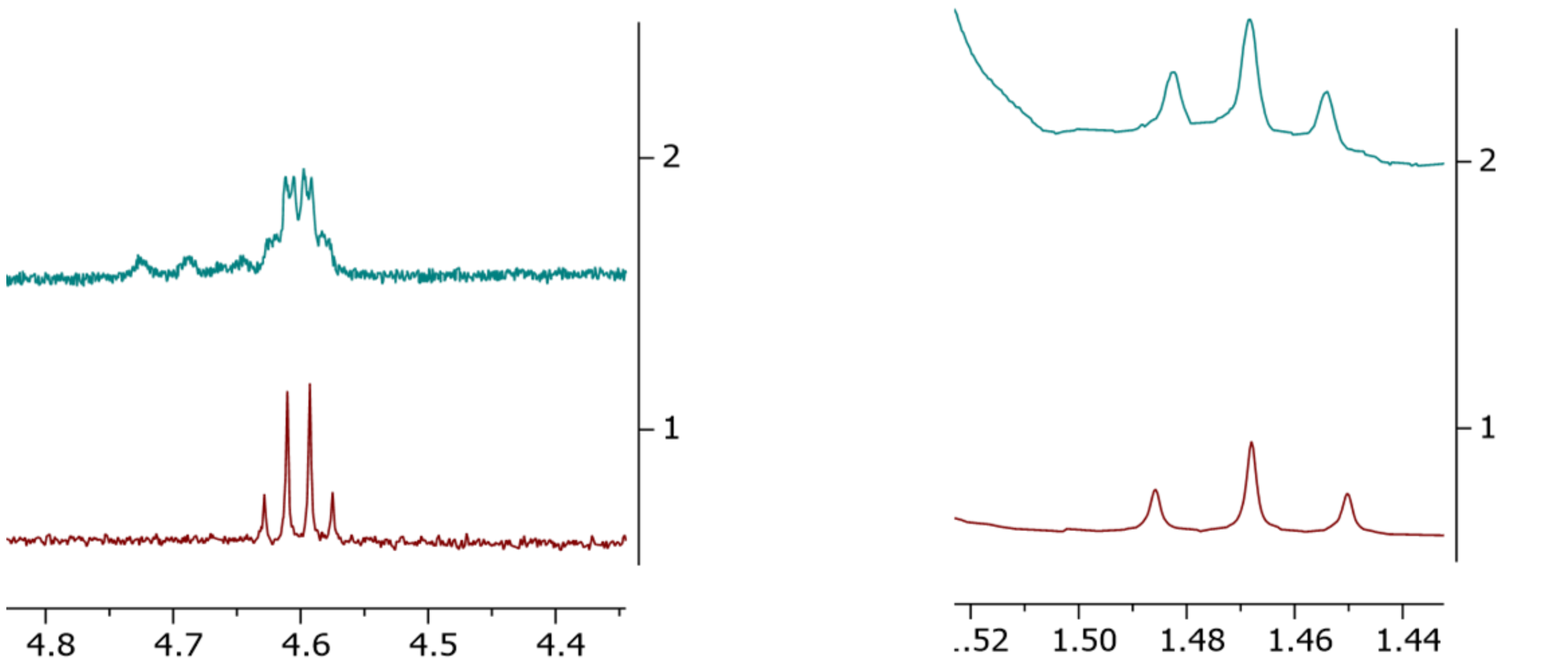

Figure S85. Comparative ^{1}H-NMR spectra ($CDCl_3$, 400 MHz, 298 K) of $Lu_3N@I_h$-$C_{80}C(CO_2Et)_2$ obtained by Bingel reaction (down, red) and $Lu_3N@I_h$-$C_{80}{}^{13}C({}^{13}CO_2Et)_2$ obtained by Bingel-Hirsh reaction with labelled diethyl malonate (top, green).

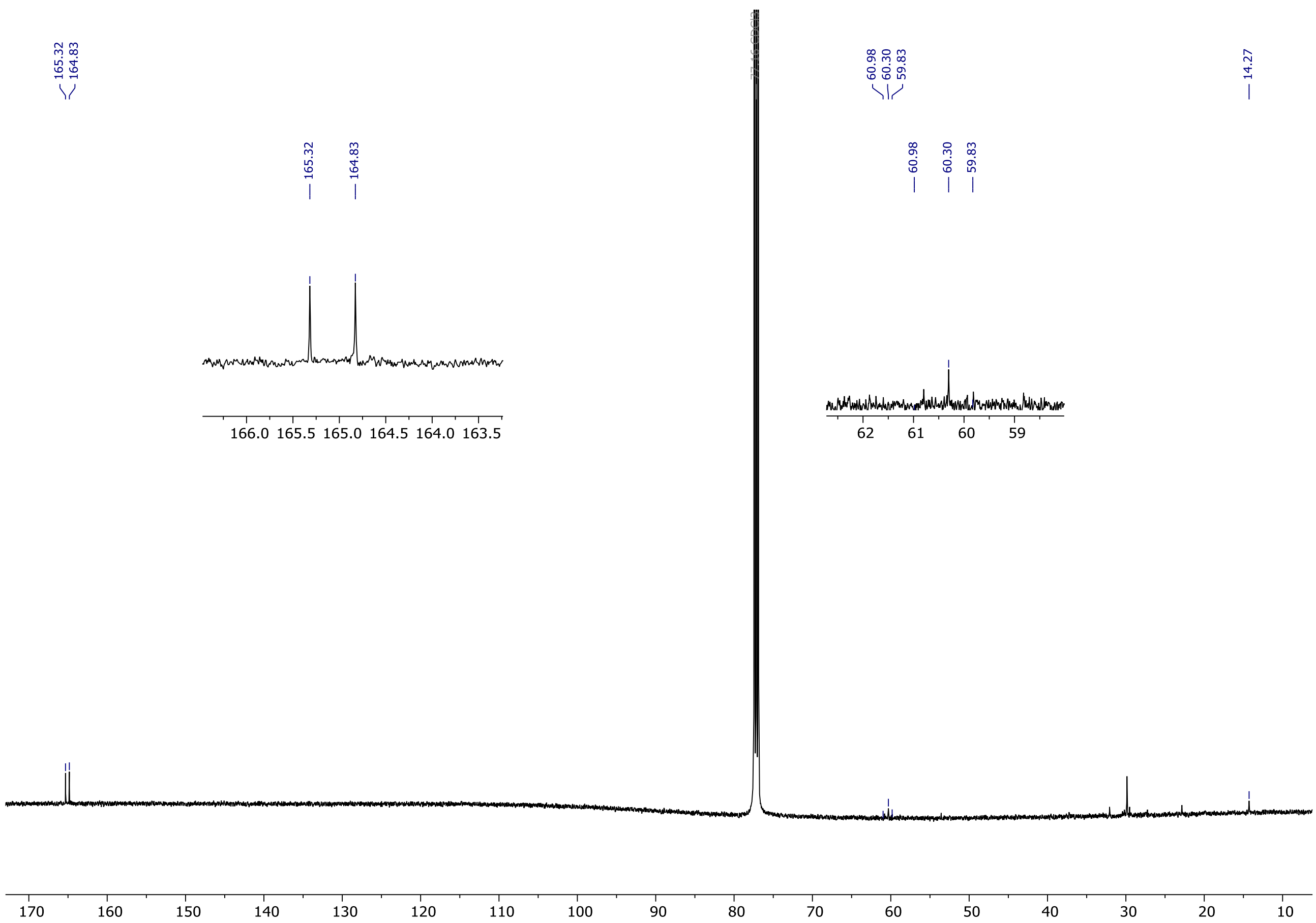

Figure S86. ^{13}C-NMR Spectrum of $Lu_3N@I_h$-$C_{80}{}^{13}C({}^{13}CO_2Et)_2$. Experiment performed in $CDCl_3$ at 298K (100 MHz).

<u>^{13}C-NMR</u> (100 MHz, $CDCl_3$) δ p.p.m.: 165.08 (^{13}C=O, d, *J*=49Hz), 60.30 (-C-^{13}C-C-, t, *J*=49Hz).

2.5. Cyclic Voltammetry of Host-guest complexes

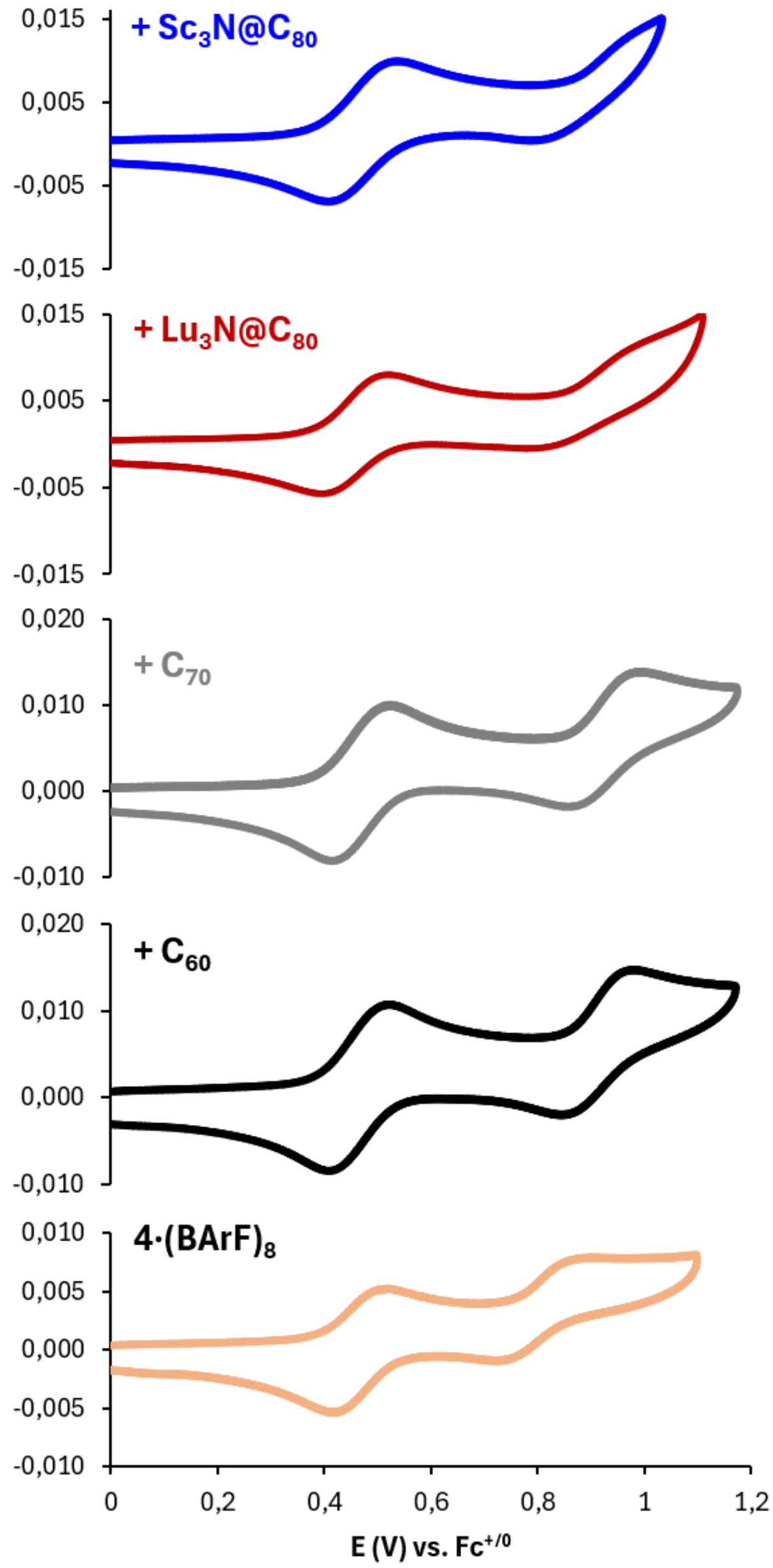

Figure S87. Cyclic voltammetry experiments were carried out inside an N_2-filled glovebox using a CHI620D potentiostat in a single-compartment, three-electrode configuration. A glassy carbon disk working electrode (3 mm diameter), a platinum counter electrode, and a silver wire pseudo-reference electrode were employed. The pseudo-reference electrode was separated from the analyte solution by a porous PTFE fritted salt bridge containing the same electrolyte solution. All potentials were externally calibrated against the ferrocene/ferrocenium ($Fc^{0/+}$) redox couple. Voltammograms were recorded with an initial anodic scan from −0.4 V toward positive potentials vs $Fc^{0/+}$. Cyclic voltammogram of **4**·$(BArF)_8$ (orange) ($E_{1/2}$ (first oxidation) = 0.47V), C_{60} ⊂ **4**·$(BArF)_8$ (black) ($E_{1/2}$ (first oxidation) = 0.46V), C_{70} ⊂ **4**·$(BArF)_8$ (gray) ($E_{1/2}$ (first oxidation) = 0.46V),Sc_3N@I_h-C_{80}⊂ **4**·$(BArF)_8$ (red) ($E_{1/2}$ (first oxidation) = 0.46V) and of Lu_3N@I_h-C_{80}⊂ **4**·$(BArF)_8$ (red) ($E_{1/2}$ (first oxidation) = 0.45V).

2.6. UV-Vis Spectra of Host-Guest complexes

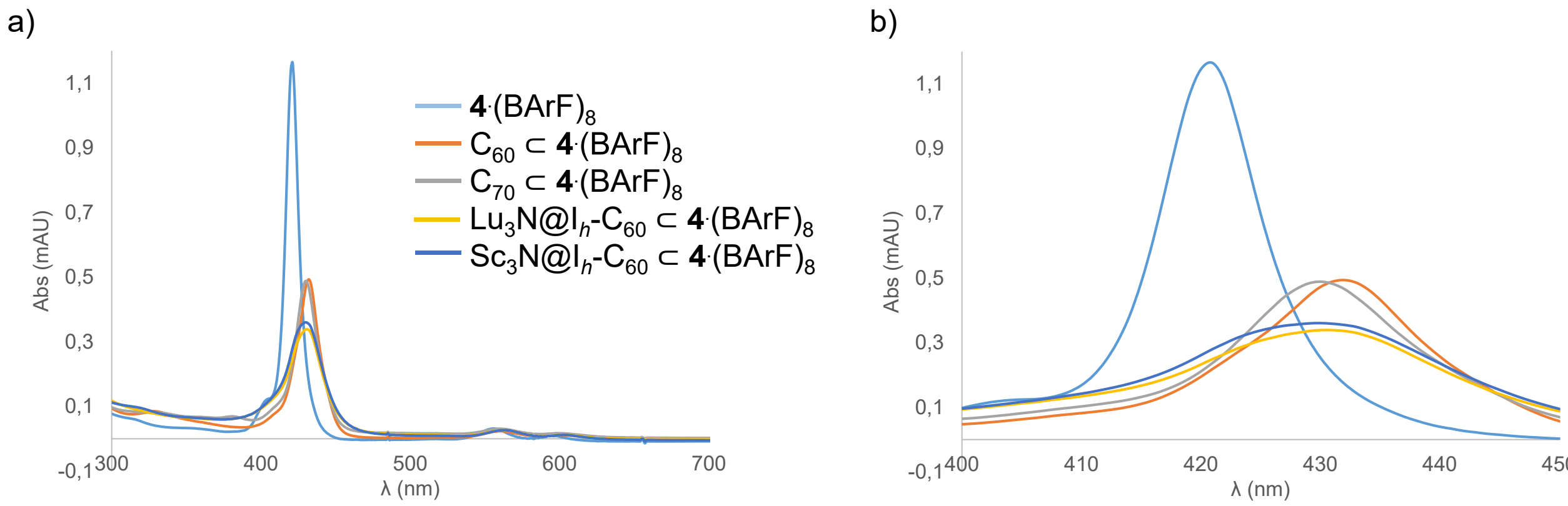

Figure S88. a) Comparison of UV-Vis spectra of nanocapsule **4**·(BArF)$_8$ and host-guest complexes with C_{60}, C_{70}, $Lu_3N@I_h$-C_{80} and $Sc_3N@I_h$-C_{80}. 1x10^{-6} M solution of compounds in CH_3CN. b) Zoom in the 400-450 nm region.

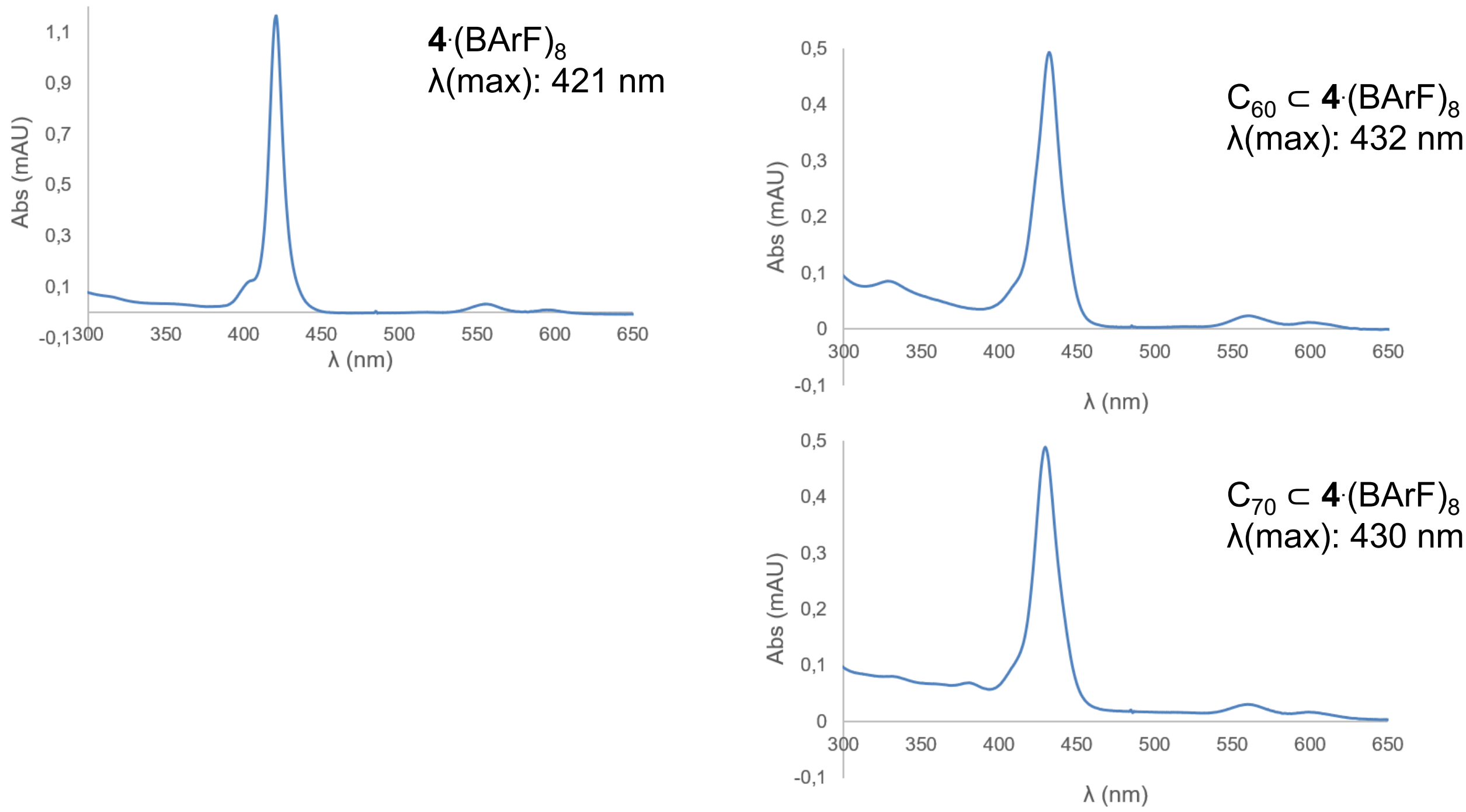

Figure S89. UV-Vis spectra of nanocapsule **4**·(BArF)$_8$ and host-guest complexes with C_{60} and C_{70}. 1x10^{-6} M solution of compounds in CH_3CN.

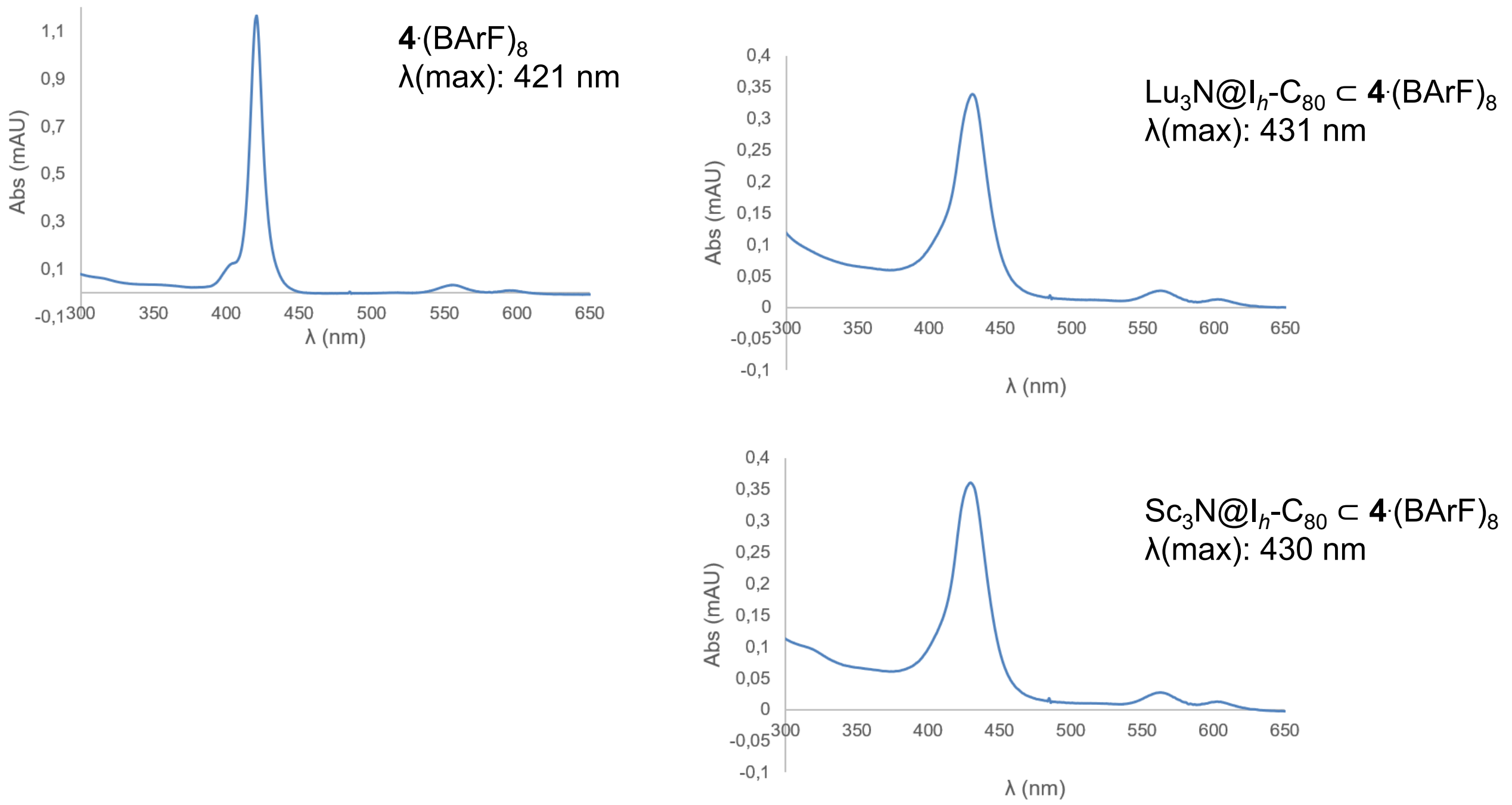

Figure S90. UV-Vis spectra of nanocapsule **4**·(BArF)$_8$ and host-guest complexes with $Lu_3N@I_h$-C_{80} and $Sc_3N@I_h$-C_{80}. $1x10^{-6}$ M solution of compounds in CH_3CN. b) Zoom in the 400-450 nm region.

2.7. Control Experiments without the nanocapsule

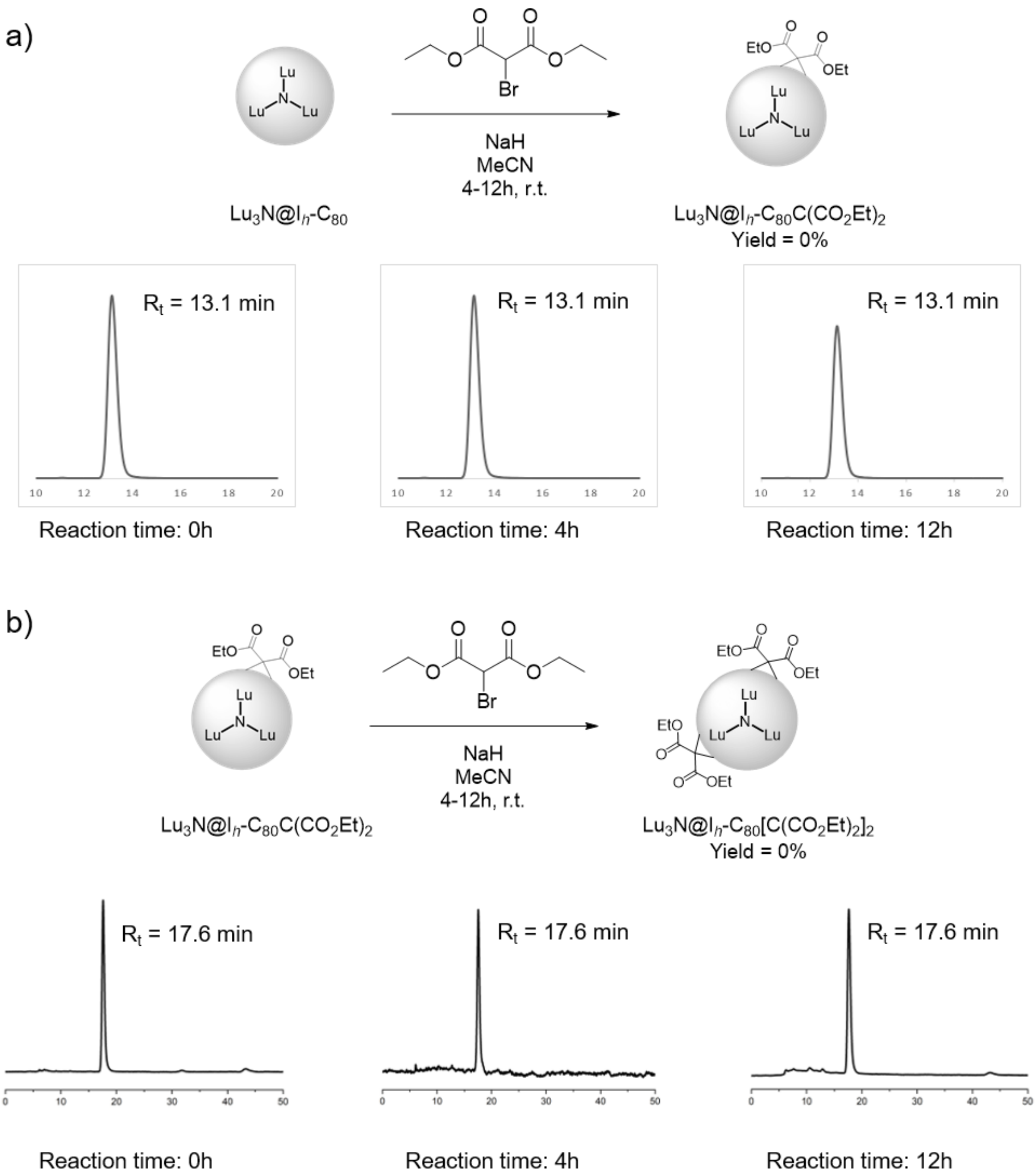

Figure S91.

a) Bingel reaction on bare $Lu_3N@I_h\text{-}C_{80}$ under identical reaction and conditions as when the reaction is performed on encapsulated $Lu_3N@I_h\text{-}C_{80}$**⊂4·**(BArF)$_8$ (MeCN, NaH, diethyl bromomalonate). No conversion to mono-adduct is observed even after 12h reaction time, probably because of lack of solubility of the TNT-EMF under these reaction conditions.

b) Bingel reaction on bare $Lu_3N@I_h\text{-}C_{80}C(CO_2Et)_2$ under identical reaction conditions as when the reaction is performed on encapsulated $Lu_3N@I_h\text{-}C_{80}C(CO_2Et)_2$**⊂4·**(BArF)$_8$ (MeCN, NaH, diethyl bromomalonate). No conversion to bis-adducts is observed even after 12h reaction time, probably because of lack of solubility of the mono-adduct under these reaction conditions.

2.8. Stability of the Host-Guest complex under basic conditions

To investigate the stability of the host-guest complex $Lu_3N@I_h$-C_{80}⊂4·$(BArF)_8$ under the basic condition employed for the Bingel functionalization, a series of experiments was performed in which increasing amounts of base were added to the nanocapsule and the stability of the supramolecular assembly and integrity of the nanocapsule was monitored by high resolution mass spectrometry.The experiments were performed under the same solvent conditions (MeCN, concentration $2x10^{-3}$ M) used for the functionalization reaction.

Table S1. Host-guest stability tests under basic conditions

✓: no signs of nanocapsule disassembly.

≈: appearance of peaks in the HR-MS accounting for nanocapsule degradation.

✗: nanocapsule mostly disassembled.

Notably, in all cases we never observe empty nanocapsules, the base is not prompting the dis-encapsulation of the guest.

NaH (Base equiv)	1h reaction	3h reaction	6h reaction	12h reaction
2	✓	✓	✓	≈
4	✓	✓	≈	✗
6	✓	≈	✗	✗
8	✓	✗	✗	✗

In the absence of, or at low amounts of base, the characteristic signals corresponding to the intact nanocapsule were observed throughout the investigated time interval. Upon increasing the amount of base, the signals associated with the intact assembly progressively decreased, accompanied by the appearance of signals attributable to partially/fully disassembled species. These results indicate that higher concentrations of base progressively compromise the stability of the nanocapsule.

It should be noted that the stability of thenanocapsule may also depend on the identity and chemical properties of the encapsulated guest. Guest–host interactions can affect the stability of the assembly and therefore the stability range determined in these experiments should be considered specific to the investigated supramolecular system and guest.

3. X-Ray Diffraction

Single crystal data were collected at ALBA synchrotron at room temperature, with samples placed inside a glass capillary, and employing a wavelength of 0.82656 Å. Data was reduced with Xia2, and the structures were solved by intrinsic phasing using SHELXT and refined against F^2 using SHELXL, as implemented in Olex2. Initial solutions readily located the atoms of the nanocages, which were subsequently refined anisotropically. Inspection of difference Fourier maps revealed electron-density maxima attributable to the Lu_3N cluster and to carbon atoms of the C_{80} cage. The occupancies of these components were fixed to reflect the symmetry-imposed (statistical) disorder. A solvent mask was applied to account for diffuse electron density arising from highly disordered solvent and counterions ($BArF^-$), which could not be modelled satisfactorily with an atomistic description.

Both structures were refined in the tetragonal *P*4/*mnc* space group. Attempts to refine the data in lower-symmetry space groups, aiming at a more explicit disorder model for $Lu_3N@I_h$-C_{80}, including the use of rigid-body restraints, did not lead to improved residuals, consistent with substantial positional/statistical disorder. For the mono-adduct, only the atoms closest to the C_{80} cage could be modelled explicitly; nevertheless, regions of residual electron density were consistently observed at positions compatible with the presence of the functional group. In addition to positional disorder, the functional group is likely affected by conformational disorder, which prevents a unique atom-by-atom assignment.

4. DFT Calculations

Table S2. Relative energies (in kcal/mol) of bis-adduct isomers; their symmetry and the angle (in degrees) between two addends, measured with respect to the center of fullerene cage (N atom).

	Bis-adduct	Symmetry	ΔE_{rel}, kcal/mol	Angle(CNC), deg
1	1-9_68-69	C_1	0.00	118.5
2	1-9_53-54	C_1	0.78	118.9
3	1-9_32-53	C_1	3.33	102.5
4	1-9_52-53	C_2	5.00	110.8
5	1-9_70-71	C_1	5.73	135.9
6	1-9_30-51	C_1	6.73	83.4
7	1-9_71-72	C_s	10.59	146
8	1-9_71-80	C_2	15.71	150.7
9	1-9_12-13	C_1	16.83	49.6
10	1-9_73-74	C_2	20.36	163
11	1-9_56-57	C_1	20.53	134
12	1-9_33-34	C_2	21.28	96.3
13	1-9_34-35	C_1	22.12	100.2
14	1-9_67-68	C_1	22.68	111.8
15	1-9_48-49	C_1	22.86	82.6
16	1-9_68-79	C_2	23.54	129.5
17	1-9_29-30	C_1	23.61	64.5
18	1-9_34-55	C_1	23.93	113.2
19	1-9_28-49	C_1	24.20	71.5
20	1-9_26-47	C_s	24.60	63.8
21	1-9_30-31	C_s	25.08	73.4
22	1-9_36-57	C_s	25.36	120.5
23	1-9_49-50	C_s	25.69	88.4
24	1-9_14-15	C_1	25.98	65.3
25	1-9_15-16	C_1	26.57	71.0
26	1-9_74-76	C_s	27.83	179.8
27	1-9_3-15	C_s	37.63	55.3

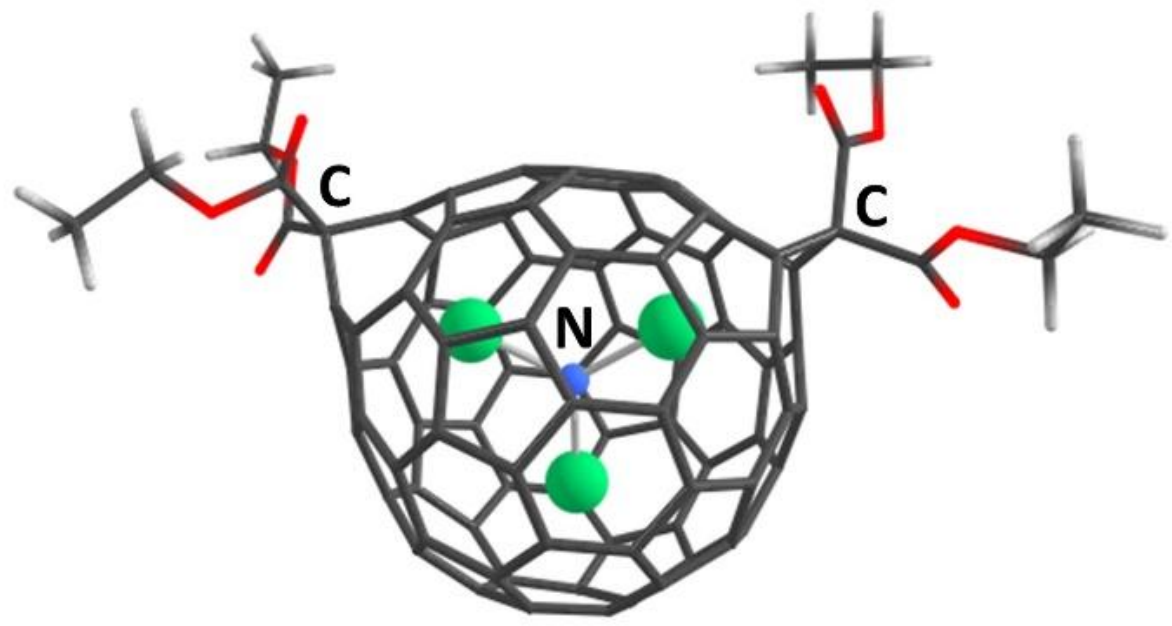

1-9_68-69
Angle(CNC)=118.5˚

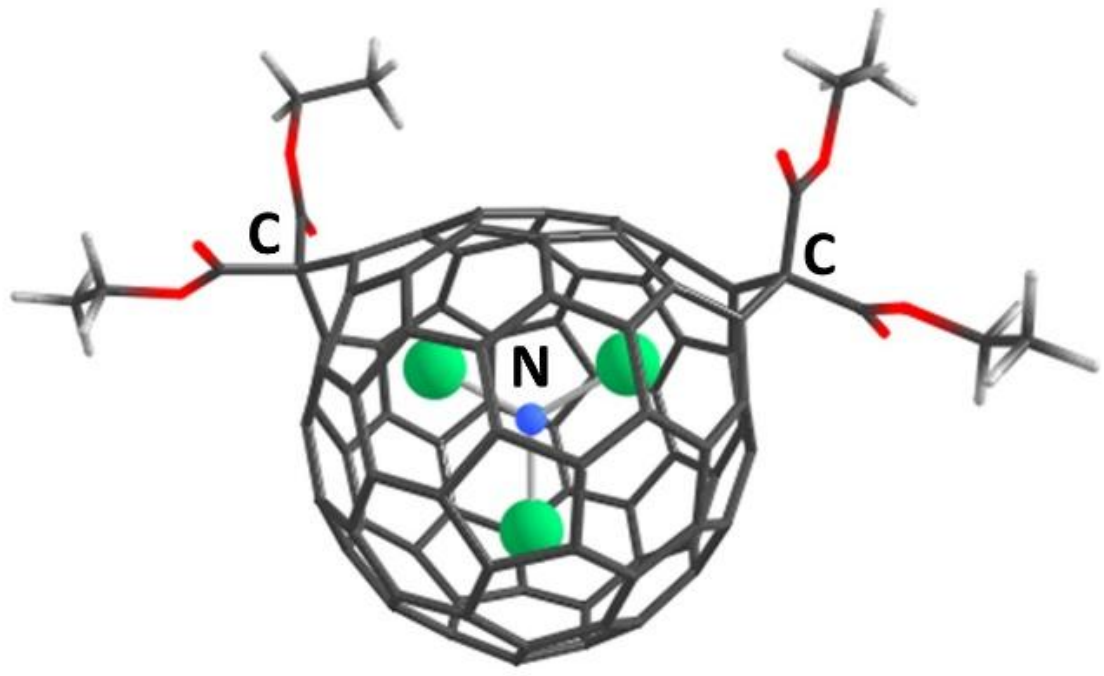

1-9_53-54
Angle(CNC)=118.9˚

Figure S92. Structure of the most stable 68-69 and 53-54 isomers of $Lu_3N@I_h$-$C_{80}C(CO_2Et)_2$.

Cartesian coordinates

Coordinates for mono- and bis-adducts of $Lu_3N@I_h$-C_{80} optimized at B3LYP-D3(BJ)/def2-SVP (charge 0, multiplicity 1)

[5,6] mono-adduct				[6,6] mono-adduct			
6	-2.811504000	-2.421720000	-3.049347000	6	3.539705000	-0.602974000	0.975267000
6	-1.366528000	-2.673895000	-3.156359000	6	2.859549000	-1.875073000	0.767795000
6	-0.735775000	-1.523652000	-3.753223000	6	2.023615000	-2.055409000	1.939391000
6	-1.742429000	-0.524592000	-3.985430000	6	2.020463000	-0.844616000	2.694724000
6	-3.016625000	-1.046959000	-3.544324000	6	2.853312000	0.131503000	2.022913000
6	-3.922582000	-0.112795000	-2.896728000	6	2.338036000	1.510593000	1.954536000
6	-4.660627000	-0.592230000	-1.767544000	6	2.373383000	2.289236000	0.723210000
6	-4.436498000	-1.922310000	-1.228509000	6	2.804230000	1.805346000	-0.608780000
6	-3.503151000	-2.848814000	-1.825881000	6	3.466270000	0.570535000	-0.928248000
6	-2.743865000	-3.611779000	-0.867246000	6	2.799780000	-0.249380000	-1.894299000
6	-1.334120000	-3.918819000	-1.049226000	6	2.367506000	-1.655085000	-1.746820000
6	-0.608552000	-3.393868000	-2.154678000	6	2.340282000	-2.419489000	-0.506198000
6	0.777044000	-3.057087000	-1.936649000	6	1.266525000	-3.390779000	-0.393773000
6	1.408293000	-1.962369000	-2.614612000	6	0.610358000	-3.701709000	0.858492000
6	0.641796000	-1.159642000	-3.516503000	6	0.943814000	-2.987990000	2.042005000
6	0.963350000	0.215314000	-3.607643000	6	-0.069310000	-2.725642000	3.000865000
6	-0.034683000	1.227214000	-3.827416000	6	-0.068612000	-1.501908000	3.765193000
6	-1.407820000	0.881858000	-3.981185000	6	0.944479000	-0.527139000	3.580504000
6	-2.383239000	1.786180000	-3.473636000	6	0.613722000	0.849729000	3.705671000
6	-3.619483000	1.288349000	-2.920715000	6	1.269166000	1.836792000	2.876706000
6	-3.990066000	2.161343000	-1.827646000	6	0.284069000	2.851159000	2.600673000
6	-4.655085000	1.653840000	-0.681457000	6	0.287051000	3.578747000	1.387198000
6	-5.041556000	0.281063000	-0.684845000	6	1.345626000	3.297650000	0.468578000
6	-5.046618000	-0.488257000	0.529683000	6	1.105967000	3.373819000	-0.945074000
6	-4.666549000	-1.839520000	0.203169000	6	1.951238000	2.426848000	-1.590789000
6	-3.916846000	-2.632242000	1.123172000	6	1.525836000	1.729157000	-2.763774000
6	-2.974681000	-3.549540000	0.563942000	6	1.946008000	0.367562000	-2.878718000
6	-1.736812000	-3.835932000	1.236729000	6	1.098708000	-0.624893000	-3.448256000
6	-0.724245000	-4.072619000	0.241176000	6	1.338371000	-1.865058000	-2.762962000
6	0.635381000	-3.683538000	0.462299000	6	0.278819000	-2.809284000	-2.609528000
6	1.399304000	-3.202151000	-0.650855000	6	0.278481000	-3.583568000	-1.425064000
6	2.469936000	-2.215419000	-0.51617900	6	-0.976061000	-4.004535000	-0.824822000

6	2.470557000	-1.418662000	-1.774257000	6	-0.754622000	-4.065211000	0.602969000
6	2.829323000	-0.005609000	-1.919196000	6	-1.797302000	-3.728856000	1.524524000
6	1.988608000	0.769258000	-2.771035000	6	-1.437010000	-3.096125000	2.751052000
6	1.610848000	2.126615000	-2.443419000	6	-2.280465000	-2.090116000	3.352330000
6	0.362549000	2.406752000	-3.100332000	6	-1.434356000	-1.112484000	3.993845000
6	-0.615863000	3.248678000	-2.487945000	6	-1.792670000	0.268593000	4.028365000
6	-1.992961000	2.957829000	-2.729193000	6	-0.748852000	1.243289000	3.926656000
6	-2.986084000	3.191332000	-1.709935000	6	-0.968532000	2.503548000	3.249212000
6	-2.623785000	3.736748000	-0.440284000	6	-2.257238000	2.927112000	2.755830000
6	-3.357378000	3.290690000	0.724640000	6	-2.273061000	3.781321000	1.563302000
6	-4.335014000	2.248700000	0.592553000	6	-0.997735000	4.031230000	0.886361000
6	-4.391094000	1.519008000	1.831926000	6	-1.217542000	4.003987000	-0.535421000
6	-4.683789000	0.100033000	1.786361000	6	-0.181978000	3.684441000	-1.479361000
6	-4.010026000	-0.724380000	2.728476000	6	-0.568666000	3.071438000	-2.702204000
6	-3.639437000	-2.079027000	2.414983000	6	0.278014000	2.094275000	-3.341698000
6	-2.397404000	-2.361361000	3.084042000	6	-0.572408000	1.095296000	-3.940870000
6	-1.417471000	-3.217613000	2.489104000	6	-0.188968000	-0.274200000	-3.956952000
6	-0.044148000	-2.931830000	2.748020000	6	-1.227792000	-1.256701000	-3.825094000
6	0.959316000	-3.172267000	1.746688000	6	-1.009453000	-2.542813000	-3.221169000
6	1.983988000	-2.181126000	1.889363000	6	-2.282191000	-3.039593000	-2.701623000
6	2.828277000	-1.742620000	0.827604000	6	-2.265647000	-3.740606000	-1.416362000
6	3.476038000	-0.458495000	1.041179000	6	-3.307242000	-3.412047000	-0.468287000
6	3.476937000	0.729861000	-0.848741000	6	-3.080696000	-3.422418000	0.966243000
6	2.779651000	1.873087000	-0.281393000	6	-3.917561000	-2.420082000	1.567951000
6	1.901780000	2.647389000	-1.147274000	6	-3.506963000	-1.706823000	2.744485000
6	0.965953000	3.548161000	-0.579480000	6	-3.916058000	-0.337144000	2.874679000
6	-0.270455000	3.866784000	-1.245215000	6	-3.074751000	0.640176000	3.510387000
6	-1.262431000	4.106447000	-0.236374000	6	-3.294140000	1.925419000	2.869717000
6	-0.641841000	3.937300000	1.061115000	6	-4.275176000	1.709771000	1.815411000
6	-1.379840000	3.489468000	2.206419000	6	-4.247386000	2.467427000	0.597392000
6	-2.811557000	3.250012000	2.069603000	6	-3.264738000	3.534365000	0.495074000
6	-3.477206000	2.137537000	2.786562000	6	-2.592170000	3.682106000	-0.780735000
6	-2.694184000	1.248937000	3.648112000	6	-2.961462000	2.967582000	-1.984783000
6	-2.997875000	-0.159068000	3.604470000	6	-1.943317000	2.703474000	-2.944831000
6	-2.003687000	-1.190151000	3.820915000	6	-1.945335000	1.477569000	-3.711866000
6	-0.628352000	-0.858790000	3.999170000	6	-2.966204000	0.501849000	-3.527964000
6	0.352370000	-1.774770000	3.509175000	6	-2.601693000	-0.894138000	-3.644002000
6	1.599830000	-1.298930000	2.972202000	6	-3.278132000	-1.976576000	-2.954478000
6	1.887519000	0.091660000	2.884625000	6	-4.263222000	-1.603886000	-1.951325000
6	2.773976000	0.541893000	1.823539000	6	-4.289956000	-2.369194000	-0.735809000
6	2.505529000	1.835173000	1.167203000	6	-4.661473000	-1.767035000	0.527049000
6	1.455301000	2.704890000	1.712959000	6	-5.000879000	-0.379520000	0.627996000
6	0.747649000	3.578757000	0.839552000	6	-4.658515000	0.315206000	1.831701000
6	0.737557000	2.289593000	2.880479000	6	-5.021002000	0.375378000	-0.577943000
6	-0.648046000	2.656108000	3.117665000	6	-4.644427000	1.769982000	-0.597576000
6	-1.293360000	1.560215000	3.828827000	6	-4.037004000	2.038279000	-1.881199000
6	-0.293389000	0.530568000	4.018683000	6	-4.037630000	0.817481000	-2.643890000
6	0.944043000	0.993206000	3.450979000	6	-4.645985000	-0.219717000	-1.840346000
6	4.469264000	0.057507000	0.048787000	6	4.493335000	0.003688000	0.001461000
6	5.337345000	-1.016072000	-0.631120000	6	5.395414000	1.106685000	0.592270000
8	5.834862000	-1.836904000	0.281006000	8	6.039456000	1.711384000	-0.395110000
6	6.723380000	-2.892233000	-0.174863000	6	6.945898000	2.794816000	-0.055142000
1	6.153156000	-3.539730000	-0.858190000	1	6.380981000	3.538368000	0.526949000
1	7.541638000	-2.427170000	-0.744562000	1	7.737512000	2.382264000	0.588403000
6	5.475653000	1.048657000	0.675928000	6	5.427195000	-1.039823000	-0.640570000
8	6.028469000	1.785935000	-0.272788000	8	6.116206000	-1.649214000	0.311236000
6	7.016617000	2.771614000	0.129325000	6	7.068294000	-2.671490000	-0.088149000

1	7.817620000	2.248778000	0.673370000	1	6.534629000	-3.407755000	-0.707475000
1	6.528225000	3.471547000	0.823957000	1	7.837784000	-2.190271000	-0.710491000
8	5.570393000	-1.057686000	-1.814317000	8	5.494371000	1.369177000	1.764204000
8	5.729461000	1.106591000	1.853708000	8	5.520494000	-1.250764000	-1.823545000
71	-1.928821000	-1.079327000	-1.358142000	71	1.262356000	-0.120295000	0.180369000
71	1.223664000	-0.061734000	-0.024891000	71	-1.790370000	1.634002000	0.777149000
71	-1.885172000	1.166938000	1.348173000	71	-1.856169000	-1.382677000	-1.095759000
7	-0.838583000	0.027992000	-0.010266000	7	-0.804117000	-0.016873000	0.027514000
6	7.219832000	-3.637314000	1.042142000	6	7.643399000	-3.286076000	1.166974000
1	6.384728000	-4.092218000	1.597206000	1	6.855396000	-3.762043000	1.771112000
1	7.900273000	-4.440384000	0.720612000	1	8.377901000	-4.056616000	0.888371000
1	7.770733000	-2.965061000	1.717824000	1	8.152901000	-2.527801000	1.781487000
6	7.519881000	3.459604000	-1.118506000	6	7.490913000	3.366697000	-1.344021000
1	6.696916000	3.958997000	-1.653279000	1	6.684060000	3.789170000	-1.962765000
1	8.262477000	4.221558000	-0.837640000	1	8.203932000	4.171685000	-1.110623000
1	8.001588000	2.740967000	-1.799242000	1	8.019394000	2.595668000	-1.925846000
**1-9_68-69 bis-adduct**				**1-9_53-54 bis-adduct**			
6	3.475175000	-0.400084000	0.816355000	6	3.519774000	-0.632183000	1.002142000
6	2.836991000	-1.698988000	0.665472000	6	2.794841000	-1.874116000	0.785359000
6	2.066766000	-1.893065000	1.875175000	6	1.930161000	-2.022916000	1.939221000
6	2.056640000	-0.671925000	2.611114000	6	1.975174000	-0.817407000	2.699356000
6	2.811163000	0.321054000	1.879588000	6	2.850726000	0.122783000	2.037186000
6	2.260451000	1.679128000	1.822275000	6	2.416937000	1.524045000	1.975681000
6	2.236425000	2.443805000	0.583994000	6	2.509462000	2.299641000	0.745526000
6	2.618169000	1.942986000	-0.758664000	6	2.932904000	1.794572000	-0.577686000
6	3.303086000	0.726901000	-1.093973000	6	3.547610000	0.536371000	-0.886247000
6	2.619738000	-0.129174000	-2.013068000	6	2.872464000	-0.249756000	-1.869492000
6	2.261778000	-1.549050000	-1.833668000	6	2.389983000	-1.639611000	-1.738452000
6	2.304129000	-2.294370000	-0.573631000	6	2.285377000	-2.389524000	-0.492873000
6	1.288500000	-3.306706000	-0.391571000	6	1.157354000	-3.301402000	-0.395009000
6	0.688623000	-3.613022000	0.895004000	6	0.443172000	-3.587589000	0.828588000
6	1.030215000	-2.861514000	2.042721000	6	0.798363000	-2.895798000	2.015146000
6	0.033846000	-2.608454000	3.033862000	6	-0.211811000	-2.585933000	2.952736000
6	0.025936000	-1.374828000	3.771666000	6	-0.149968000	-1.377753000	3.739071000
6	1.009764000	-0.377461000	3.535292000	6	0.913622000	-0.451010000	3.577558000
6	0.637346000	0.982912000	3.650165000	6	0.658058000	0.939219000	3.711437000
6	1.224347000	1.984336000	2.787784000	6	1.371041000	1.902068000	2.896516000
6	0.206605000	2.971391000	2.544047000	6	0.441641000	2.965642000	2.610987000
6	0.144143000	3.680003000	1.314389000	6	0.486796000	3.676948000	1.387752000
6	1.178504000	3.419444000	0.360776000	6	1.535847000	3.352151000	0.477375000
6	0.871199000	3.464979000	-1.038758000	6	1.318134000	3.442895000	-0.941387000
6	1.701386000	2.519987000	-1.709115000	6	2.128710000	2.457540000	-1.574234000
6	1.239040000	1.784956000	-2.844003000	6	1.696029000	1.783347000	-2.755987000
6	1.681886000	0.438733000	-2.950034000	6	2.060472000	0.404786000	-2.864301000
6	0.847622000	-0.587431000	-3.482858000	6	1.187774000	-0.552057000	-3.456186000
6	1.202725000	-1.807852000	-2.793861000	6	1.385500000	-1.805700000	-2.781219000
6	0.204304000	-2.786631000	-2.559518000	6	0.289895000	-2.701724000	-2.649673000
6	0.271480000	-3.559631000	-1.380335000	6	0.221929000	-3.473447000	-1.458940000
6	-0.930599000	-4.005901000	-0.718151000	6	-1.035691000	-3.952956000	-0.939963000
6	-0.675111000	-4.034421000	0.691702000	6	-0.935243000	-3.942821000	0.533804000
6	-1.708255000	-3.691855000	1.622117000	6	-1.997999000	-3.556723000	1.462995000
6	-1.325864000	-3.018513000	2.824942000	6	-1.590835000	-2.893956000	2.677293000
6	-2.182580000	-2.032744000	3.432286000	6	-2.378298000	-1.854232000	3.316814000
6	-1.348540000	-1.009032000	4.034071000	6	-1.490364000	-0.922525000	3.966106000
6	-1.773643000	0.358190000	4.165794000	6	-1.779654000	0.479358000	4.004344000
6	-0.739007000	1.336616000	3.924795000	6	-0.687038000	1.399628000	3.914959000
6	-0.996158000	2.578142000	3.232342000	6	-0.828781000	2.665478000	3.236101000

6	-2.296332000	2.883046000	2.724755000	6	-2.076546000	3.103273000	2.675344000
6	-2.352850000	3.664514000	1.531342000	6	-2.053976000	3.955068000	1.496521000
6	-1.147161000	4.065439000	0.840430000	6	-0.772621000	4.150897000	0.847415000
6	-1.437768000	4.107201000	-0.568231000	6	-0.981071000	4.170994000	-0.574642000
6	-0.440386000	3.753031000	-1.522377000	6	0.055495000	3.810470000	-1.501599000
6	-0.858628000	3.079750000	-2.715239000	6	-0.338154000	3.217397000	-2.732675000
6	-0.046578000	2.093449000	-3.372277000	6	0.474285000	2.202119000	-3.356190000
6	-0.908313000	1.078195000	-3.964608000	6	-0.409880000	1.236047000	-3.958426000
6	-0.469134000	-0.309466000	-4.019895000	6	-0.081618000	-0.149504000	-3.983905000
6	-1.412952000	-1.425320000	-3.986821000	6	-1.153634000	-1.086409000	-3.886794000
6	-1.087703000	-2.537183000	-3.121301000	6	-0.956208000	-2.347992000	-3.253105000
6	-2.276422000	-2.958115000	-2.455036000	6	-2.212898000	-2.805409000	-2.719045000
6	-2.217030000	-3.657881000	-1.213959000	6	-2.218564000	-3.855712000	-1.746465000
6	-3.275293000	-3.409151000	-0.300948000	6	-3.873276000	-3.376930000	-0.353224000
6	-3.018189000	-3.421815000	1.112944000	6	-3.353400000	-3.244177000	0.993787000
6	-3.874031000	-2.437221000	1.726715000	6	-3.997281000	-2.075805000	1.545121000
6	-3.462736000	-1.707563000	2.888738000	6	-3.570675000	-1.387474000	2.719634000
6	-3.938169000	-0.349386000	3.076388000	6	-3.927771000	-0.010780000	2.848359000
6	-3.134502000	0.685670000	3.744653000	6	-3.037353000	0.917929000	3.481291000
6	-3.357912000	1.929176000	2.999848000	6	-3.175088000	2.192146000	2.811404000
6	-4.297349000	1.659223000	1.940367000	6	-4.169361000	2.045275000	1.762583000
6	-4.331411000	2.399400000	0.705476000	6	-4.112807000	2.806373000	0.542761000
6	-3.380325000	3.445436000	0.537410000	6	-3.072776000	3.821678000	0.434923000
6	-2.819077000	3.740699000	-0.756821000	6	-2.374169000	3.939398000	-0.837717000
6	-3.213596000	2.990986000	-1.901994000	6	-2.742112000	3.208599000	-2.035911000
6	-2.224099000	2.715152000	-2.895896000	6	-1.725634000	2.897805000	-2.984496000
6	-2.291901000	1.537358000	-3.721890000	6	-1.766523000	1.662313000	-3.732779000
6	-3.498172000	0.761627000	-3.752688000	6	-2.815665000	0.712189000	-3.538122000
6	-2.857109000	-1.337517000	-3.988040000	6	-2.509892000	-0.679934000	-3.660687000
6	-3.373415000	-2.111395000	-2.872839000	6	-3.193334000	-1.712190000	-2.895342000
6	-4.316633000	-1.668068000	-1.831259000	6	-4.235323000	-1.339108000	-1.948760000
6	-4.281808000	-2.406839000	-0.589853000	6	-4.493633000	-2.125268000	-0.735542000
6	-4.644708000	-1.813268000	0.675859000	6	-4.691368000	-1.398929000	0.499160000
6	-5.009290000	-0.448108000	0.769389000	6	-4.965812000	-0.002404000	0.585242000
6	-4.642616000	0.263750000	1.976200000	6	-4.621377000	0.673101000	1.789862000
6	-5.096957000	0.293328000	-0.442946000	6	-4.940786000	0.717171000	-0.632043000
6	-4.776327000	1.697169000	-0.457738000	6	-4.487769000	2.090589000	-0.651380000
6	-4.234283000	2.007372000	-1.738896000	6	-3.837697000	2.302362000	-1.924076000
6	-4.295301000	0.830798000	-2.570720000	6	-3.876878000	1.066947000	-2.660374000
6	-4.768159000	-0.286022000	-1.738585000	6	-4.554432000	0.072147000	-1.868216000
6	4.382368000	0.204513000	-0.201346000	6	4.530555000	-0.069601000	0.062542000
6	5.282970000	1.344595000	0.315985000	6	5.455704000	1.004832000	0.668658000
8	5.862300000	1.933974000	-0.720894000	8	6.103896000	1.611072000	-0.315862000
6	6.749723000	3.055879000	-0.470504000	6	7.007835000	2.696946000	0.019977000
1	6.184869000	3.815837000	0.090587000	1	6.451624000	3.423643000	0.631084000
1	7.574487000	2.700038000	0.165184000	1	7.822463000	2.282476000	0.632813000
6	5.313114000	-0.836437000	-0.851354000	6	5.440393000	-1.158087000	-0.537251000
8	6.018458000	-1.433377000	0.096744000	8	6.097203000	-1.755959000	0.445677000
6	6.970522000	-2.455751000	-0.299924000	6	7.036770000	-2.807339000	0.098387000
1	6.434652000	-3.201662000	-0.905768000	1	6.490396000	-3.576121000	-0.468631000
1	7.733012000	-1.979242000	-0.934456000	1	7.802699000	-2.371334000	-0.560546000
8	5.431791000	1.642845000	1.473797000	8	5.564952000	1.250315000	1.843059000
8	5.394407000	-1.053643000	-2.034241000	8	5.545386000	-1.409554000	-1.711180000
71	1.129835000	-0.003383000	0.048945000	71	1.244801000	-0.070473000	0.144689000
71	-1.996897000	0.310977000	1.713632000	71	-1.836662000	1.789646000	0.483640000
71	-2.010453000	-0.278156000	-1.869376000	71	-2.041278000	-1.660159000	-0.423798000
7	-0.968141000	0.022862000	-0.074393000	7	-0.851149000	0.001640000	0.052367000

6	7.558731000	-3.053473000	0.957700000	6	7.622860000	-3.347266000	1.383145000
1	6.777593000	-3.525644000	1.573694000	1	6.841099000	-3.789488000	2.020019000
1	8.294783000	-3.823914000	0.683025000	1	8.358655000	-4.130323000	1.146222000
1	8.069210000	-2.285585000	1.559374000	1	8.134106000	-2.552194000	1.947932000
6	7.232289000	3.568175000	-1.809356000	6	7.507815000	3.299023000	-1.274017000
1	6.392221000	3.937000000	-2.418007000	1	6.677007000	3.718327000	-1.862591000
1	7.933959000	4.400989000	-1.651330000	1	8.214202000	4.111923000	-1.047971000
1	7.756314000	2.776983000	-2.367726000	1	8.031205000	2.546458000	-1.883867000
6	-3.649280000	-0.335703000	-4.755733000	6	-3.502664000	-4.470574000	-1.293590000
8	-5.061534000	-1.904667000	-5.752961000	8	-5.700236000	-4.991083000	-1.914084000
6	-5.093842000	-0.795662000	-5.028570000	6	-4.514336000	-4.709931000	-2.430760000
6	-6.328392000	-2.508023000	-6.132744000	6	-6.751266000	-5.421473000	-2.822656000
1	-6.928419000	-2.643495000	-5.220889000	1	-7.090701000	-4.542400000	-3.391514000
1	-6.855764000	-1.801403000	-6.791475000	1	-6.314244000	-6.141086000	-3.529565000
8	-3.649166000	1.175044000	-6.535133000	8	-2.724817000	-6.678333000	-1.437830000
6	-3.039306000	0.074028000	-6.111973000	6	-3.302985000	-5.831954000	-0.596851000
6	-3.058235000	1.919301000	-7.637113000	6	-2.316156000	-7.973375000	-0.914254000
1	-2.556902000	1.213365000	-8.312548000	1	-3.091106000	-8.328021000	-0.221831000
1	-3.909910000	2.379537000	-8.154477000	1	-2.289620000	-8.626829000	-1.795188000
8	-6.088420000	-0.225293000	-4.656857000	8	-4.241721000	-4.697056000	-3.605454000
8	-2.147416000	-0.506692000	-6.675802000	8	-3.631965000	-6.076270000	0.536455000
6	-2.103508000	2.956848000	-7.079303000	6	-0.961508000	-7.853845000	-0.241624000
1	-1.262765000	2.478468000	-6.553181000	1	-1.008674000	-7.175776000	0.624002000
1	-1.693173000	3.564417000	-7.900860000	1	-0.637093000	-8.842715000	0.118974000
1	-2.625888000	3.623736000	-6.375633000	1	-0.207879000	-7.474237000	-0.948349000
6	-6.019356000	-3.818725000	-6.818848000	6	-7.852041000	-6.032155000	-1.988303000
1	-5.493337000	-4.503025000	-6.136047000	1	-8.295665000	-5.289050000	-1.308360000
1	-6.957616000	-4.298102000	-7.137975000	1	-8.643205000	-6.414857000	-2.651027000
1	-5.392738000	-3.657714000	-7.709608000	1	-7.463008000	-6.871100000	-1.390733000

Table S3. Dipole moment (D) calculated for the six energetically favoured bis-adduct regioisomers

Functionalized bond	Symmetry	Angle CNC	ΔE_{rel} (kcal/mol)	Dipole moment, D
**68-69**	C_1	118.5°	0.00	5.63
**53-54**	C_1	118.9°	0.78	5.84
**32-53**	C_1	102.5°	3.33	9.00
**52-53**	C_2	110.8°	5.00	7.40
**70-71**	C_1	135.9°	5.73	5.77
**30-51**	C_1	83.5°	6.73	10.88

5. Molecular Dynamics simulations

$Lu_3N@I_h$-C_{80}⊂4·Cl_8

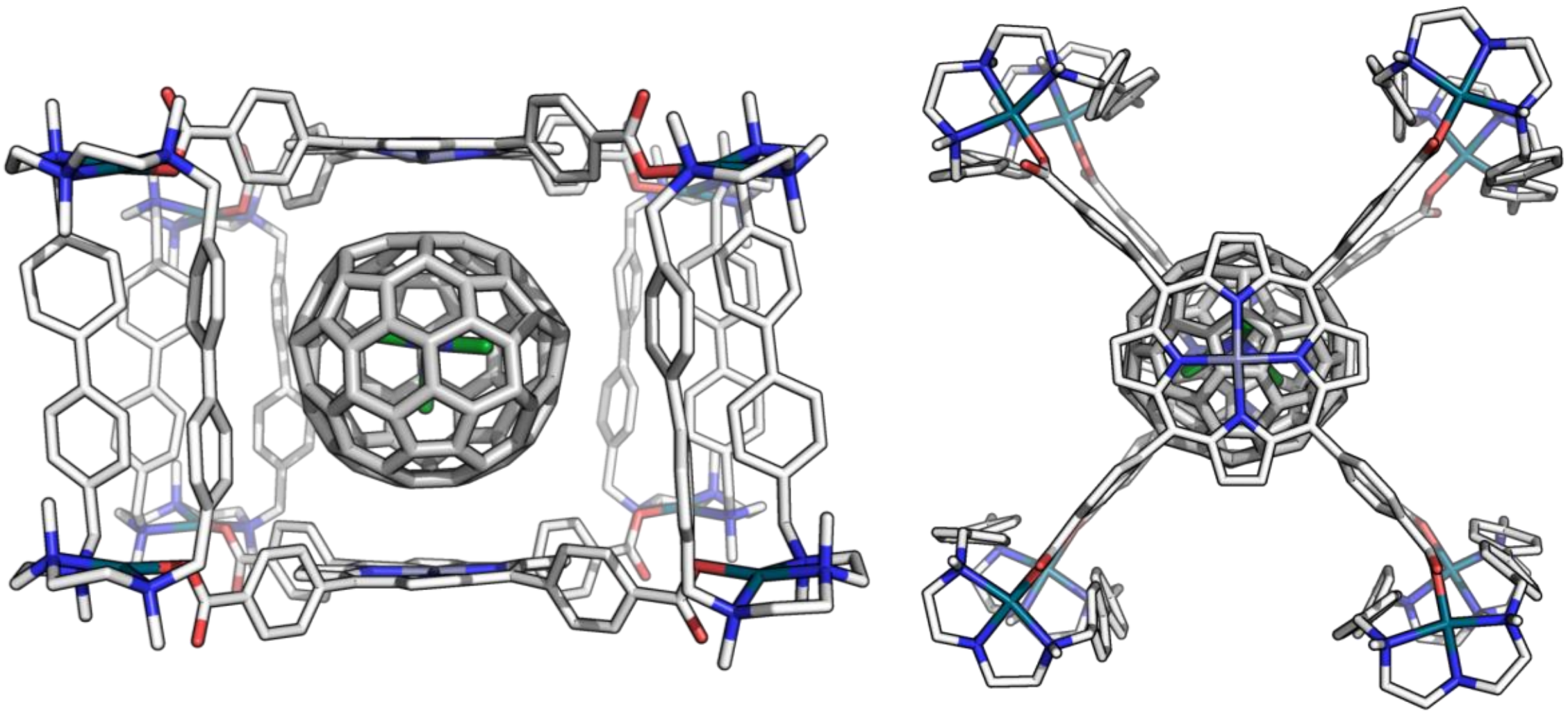

Figure S93. Snapshot of $Lu_3N@I_h$-C_{80}⊂**4**·Cl_8 during MD simulations viewed from the side and the top. Hydrogens of the cage have been omitted for clarity.

$Lu_3N@I_h\text{-}C_{80}C(CO_2Et)_2 \subset$ 4$\cdot Cl_8$

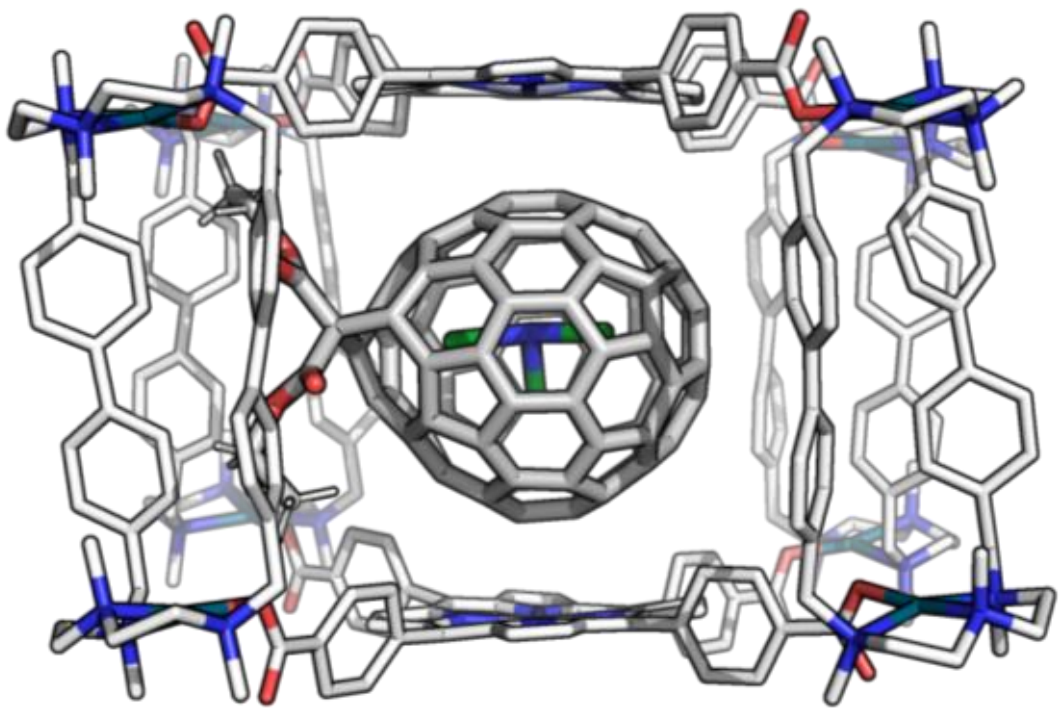

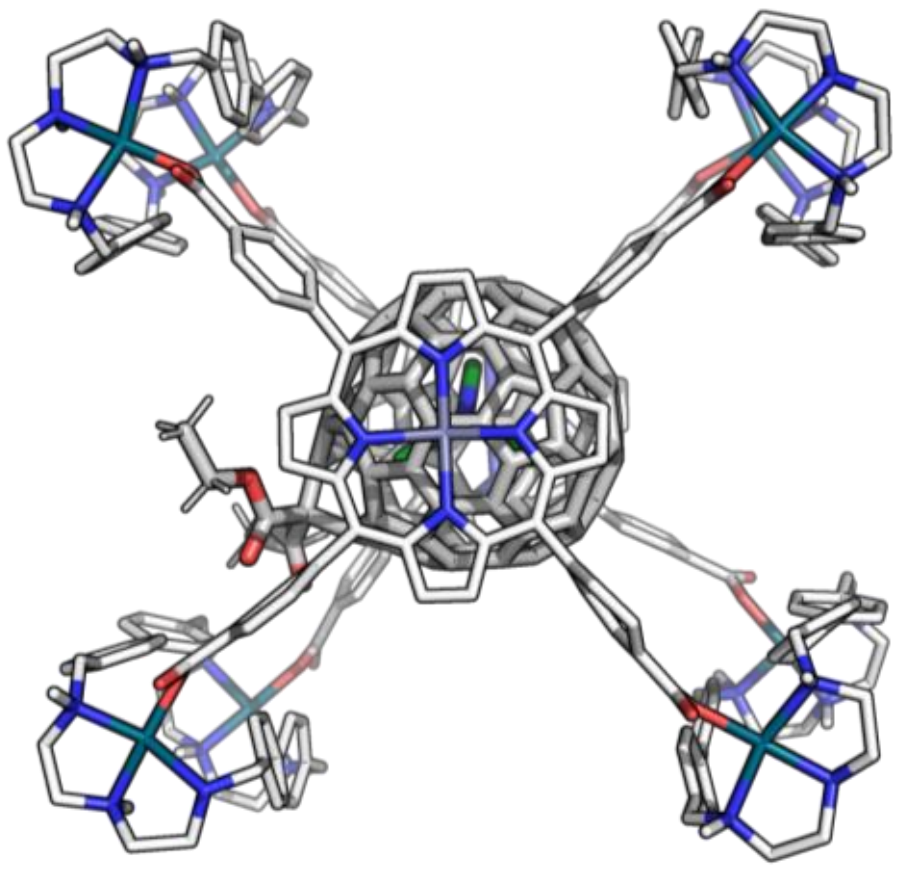

Accessibility through the window of the cage

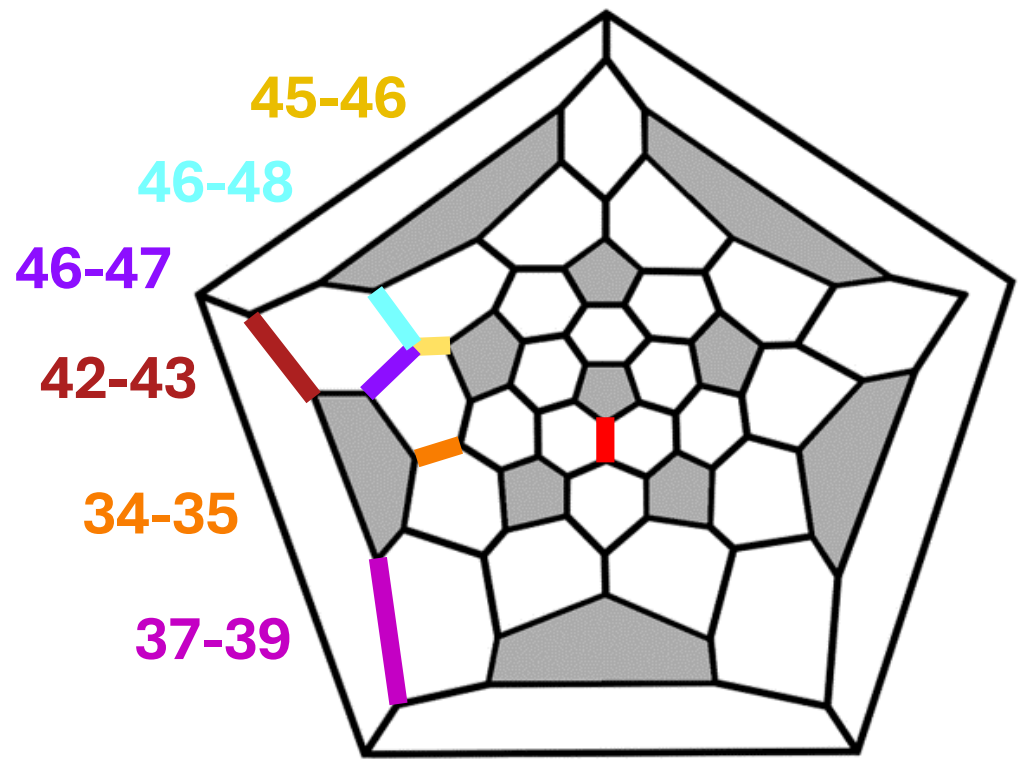

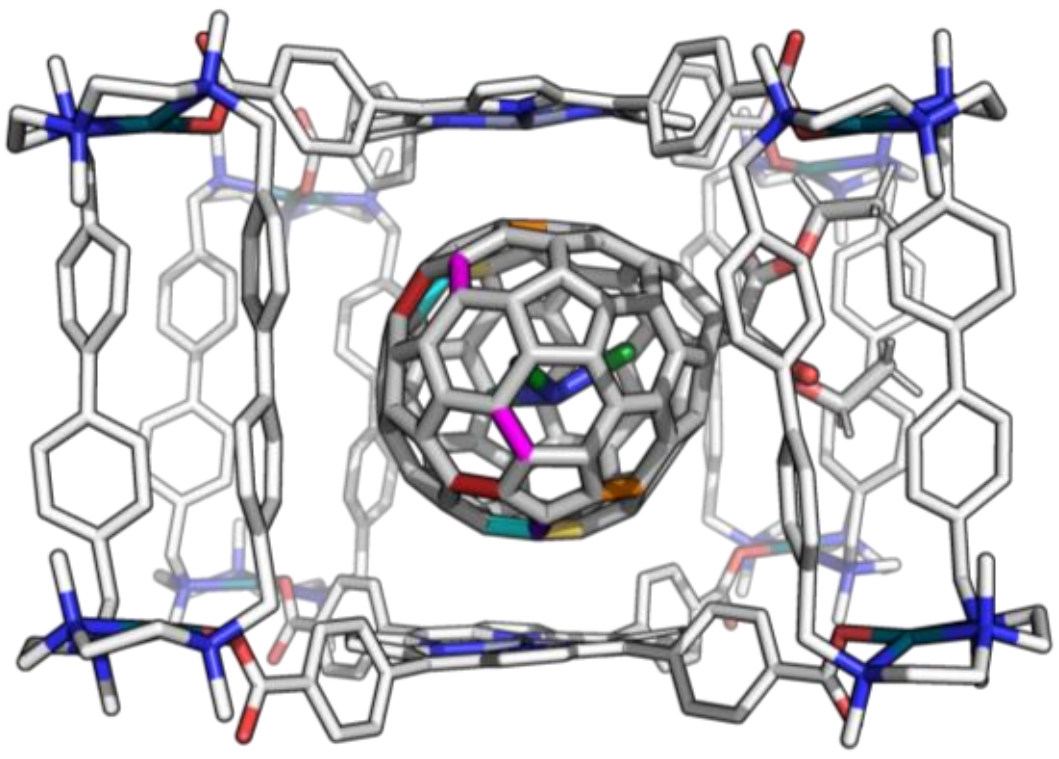

Bond 37-39 accessible

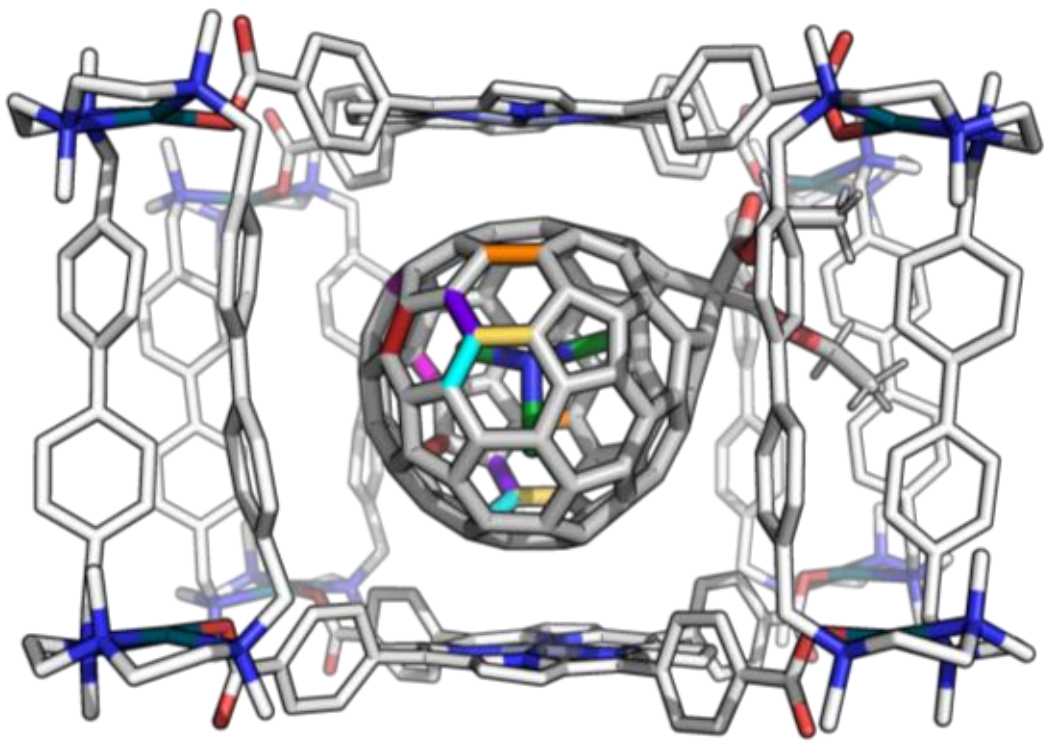

Bonds 45-46 and 46-48 accessible

Figure S94. Snapshot of $Lu_3N@I_h\text{-}C_{80}C(CO_2Et)_2 \subset$**4**$\cdot Cl_8$ during MD simulations viewed from the side and the top. Hydrogens of the cage have been omitted for clarity. Schlegel diagram of $I_h\text{-}C_{80}$ showing the bond of the first functionalization in red and the bonds of the most stable bis-adducts in different colors (32-53 in yellow, 53-54 in cyan, 52-53 in purple, 70-71 in dark red, 30-51 in orange and 68-69 in fuchsia). Accessibility of these six bonds through the window of the cage, showing the bonds 68-69, 32-53 and 53-54 accessible through contiguous windows respect of the first substituent.

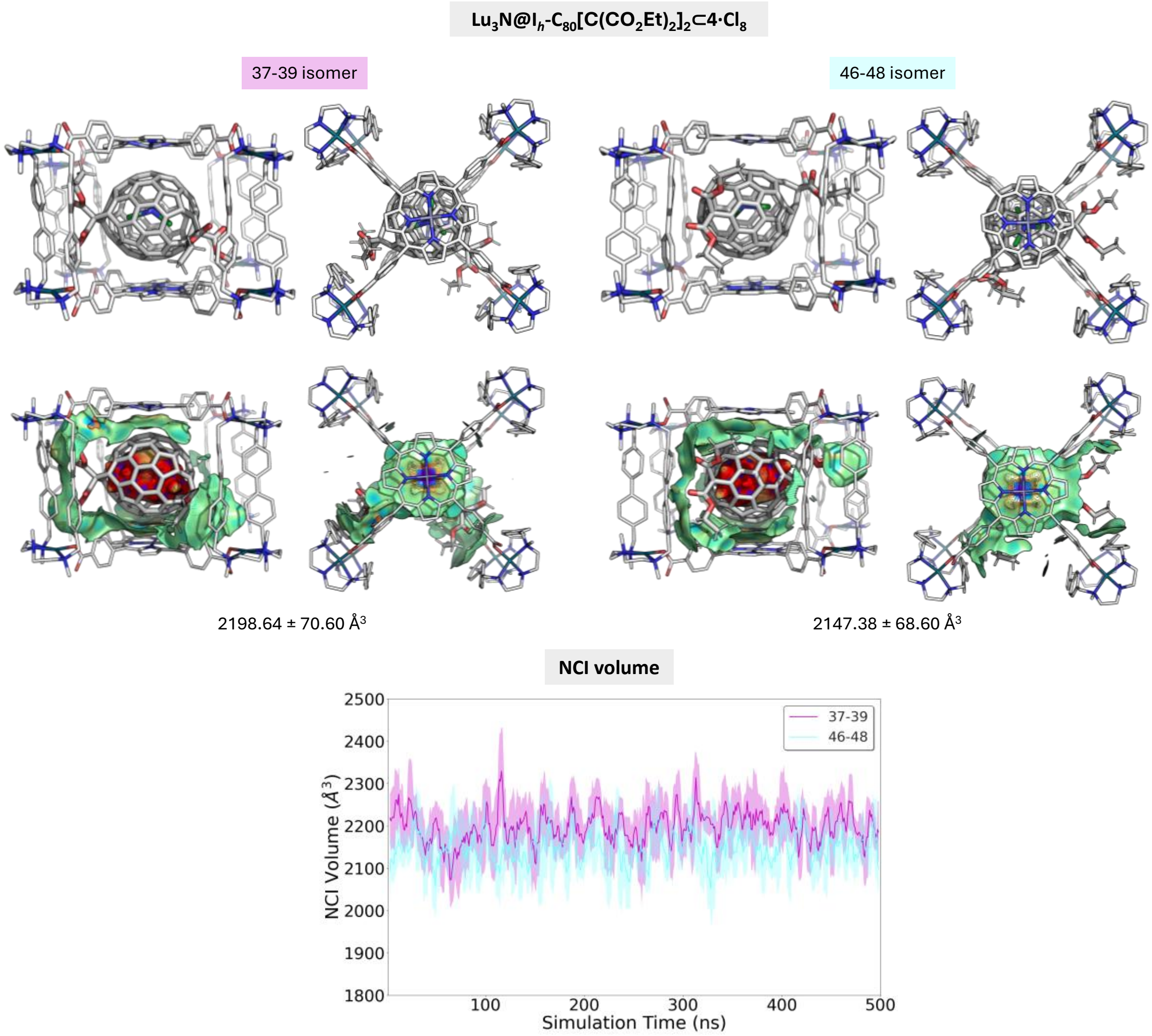

Figure S95. Snapshot of the most stable isomers of $Lu_3N@I_h$-$C_{80}[C(CO_2Et)_2]_2$⊂**4**·Cl_8 (68-69 and 53-54) during MD simulations viewed from the side and the top. Hydrogens of the cage have been omitted for clarity. Plotting of Non-Covalent Interactions (NCI) volume (in Å^3) versus the simulations time (500 ns) for one replicate of each bis-adduct inside the cage. Average of the NCI volume shown in Å^3. Visualization of the NCI using a rainbow scale. Weak van der Waals interactions in green. Gradient isosurface with s = 0.8 a.u.

6. Supporting Videos

Supplementary video S1.

Selected MD simulation replica on $Lu_3N@I_h$-$C_{80}C(CO_2Et)_2$⊂**4**·Cl_8.

Supplementary video S2.

Selected MD simulation replica on isomer 68-69 of $Lu_3N@I_h$-$C_{80}[C(CO_2Et)_2]_2$⊂**4**·Cl_8.

Supplementary video S3.

Selected MD simulation replica on isomer 53-54 of $Lu_3N@I_h$-$C_{80}[C(CO_2Et)_2]_2$⊂**4**·Cl_8.

7. References

(1) García-Simón, C.; Garcia-Borràs, M.; Gómez, L.; Parella, T.; Osuna, S.; Juanhuix, J.; Imaz, I.; Maspoch, D.; Costas, M.; Ribas, X. Sponge-like molecular cage for purification of fullerenes. *Nat. Commun.* **2014**, *5* (1), 5557. DOI: 10.1038/ncomms6557.

(2) Becke, A. D. Density-functional thermochemistry. III. The role of exact exchange. *J. Chem. Phys.* **1993**, *98* (7), 5648-5652. DOI: 10.1063/1.464913.

(3) Stephens, P. J.; Devlin, F. J.; Chabalowski, C. F.; Frisch, M. J. Ab Initio Calculation of Vibrational Absorption and Circular Dichroism Spectra Using Density Functional Force Fields. *J. Phys. Chem.* **1994**, *98* (45), 11623-11627. DOI: 10.1021/j100096a001.

(4) Grimme, S.; Antony, J.; Ehrlich, S.; Krieg, H. A consistent and accurate *ab initio* parametrization of density functional dispersion correction (DFT-D) for the 94 elements H-Pu. *J. Chem. Phys.* **2010**, *132* (15), 154104. DOI: 10.1063/1.3382344.

(5) Grimme, S.; Ehrlich, S.; Goerigk, L. Effect of the damping function in dispersion corrected density functional theory. *J. Comput. Chem.* **2011**, *32* (7), 1456-1465. DOI: 10.1002/jcc.21759.

(6) Weigend, F. Accurate Coulomb-fitting basis sets for H to Rn. *Phys. Chem. Chem. Phys.* **2006**, *8* (9), 1057. DOI: 10.1039/b515623h.

(7) Weigend, F.; Ahlrichs, R. Balanced basis sets of split valence, triple zeta valence and quadruple zeta valence quality for H to Rn: Design and assessment of accuracy. *Phys. Chem. Chem. Phys.* **2005**, 7 (18), 3297. DOI: 10.1039/b508541a.

(8) Cao, X.; Dolg, M. Valence basis sets for relativistic energy-consistent small-core lanthanide pseudopotentials. *J. Chem. Phys.* **2001**, *115* (16), 7348-7355. DOI: 10.1063/1.1406535.

(9) Neese, F. The ORCA program system. *WIREs Computational Molecular Science* **2012**, 2 (1), 73-78. DOI: 10.1002/wcms.81.

(10) Neese, F. Software update: the ORCA program system, version 4.0. *WIREs Computational Molecular Science* **2018**, *8* (1), e1327. DOI: 10.1002/wcms.1327.

(11) Berger, P. R.; Kim, M. Polymer solar cells: P3HT:PCBM and beyond. *J. Renew. Sustain. Energy* **2018**, *10* (1), 013508. DOI: 10.1063/1.5012992.

(12) *Chemcraft - Graphical Software for Visualization of Quantum Chemistry Computations*; Chemcraft: 2024. https://chemcraftprog.com (accessed.

(13) Iannace, V.; Sabrià, C.; Schmitt, R. D.; Feixas, F.; Stevenson, S.; Ribas, X. Supramolecular Length-Dependent Encapsulation of C_{90}, C_{100}, C_{120}, and C_{130}: a Shape-Selective Approach to Fullertube Purification. *J. Am. Chem. Soc.* **2025**, *147* (40), 36079-36084. DOI: 10.1021/jacs.5c12991.

(14) Wang, J.; Wolf, R. M.; Caldwell, J. W.; Kollman, P. A.; Case, D. A. Development and testing of a general amber force field. *J. Comput. Chem.* **2004**, *25* (9), 1157-1174. DOI: 10.1002/jcc.20035.

(15) Li, P.; Merz, K. M. MCPB.py: A Python Based Metal Center Parameter Builder. *J. Chem. Inf. Model.* **2016**, *56* (4), 599-604. DOI: 10.1021/acs.jcim.5b00674.

(16) Gaussian 16, Revision C.01, Frisch, M. J.; Trucks, G. W.; Schlegel, H. B.; Scuseria, G. E.; Robb, M. A.; Cheeseman, J. R.; Scalmani, G.; Barone, V.; Petersson, G. A.; Nakatsuji, H.; Li, X.; Caricato, M.; Marenich, A. V.; Bloino, J.; Janesko, B. G.; Gomperts, R.; Mennucci, B.; Hratchian, H. P.; Ortiz, J. V.; Izmaylov, A. F.; Sonnenberg, J. L.; Williams-Young, D.; Ding, F.; Lipparini, F.; Egidi, F.; Goings, J.; Peng, B.; Petrone, A.; Henderson, T.; Ranasinghe, D.; Zakrzewski, V. G.; Gao, J.; Rega, N.; Zheng, G.; Liang, W.; Hada, M.; Ehara, M.; Toyota, K.; Fukuda, R.; Hasegawa, J.; Ishida, M.; Nakajima, T.; Honda, Y.; Kitao, O.; Nakai, H.; Vreven, T.; Throssell, K.; Montgomery, J. A., Jr.; Peralta, J. E.; Ogliaro, F.; Bearpark, M. J.; Heyd, J. J.; Brothers, E. N.; Kudin, K. N.; Staroverov, V. N.; Keith, T. A.; Kobayashi, R.; Normand, J.; Raghavachari, K.; Rendell, A. P.; Burant, J. C.; Iyengar, S. S.; Tomasi, J.; Cossi, M.; Millam, J. M.; Klene, M.; Adamo, C.; Cammi, R.; Ochterski, J. W.; Martin, R. L.; Morokuma, K.; Farkas, O.; Foresman, J. B.; Fox, D. J. Gaussian, Inc., Wallingford CT, **2016**.

(17) D.A. Case, H.M. Aktulga, K. Belfon, I.Y. Ben-Shalom, J.T. Berryman, S.R. Brozell, D.S. Cerutti, T.E. Cheatham, III, G.A. Cisneros, V.W.D. Cruzeiro, T.A. Darden, R.E. Duke, G. Giambasu, M.K. Gilson, H. Gohlke, A.W. Goetz, R. Harris, S. Izadi, S.A. Izmailov, K. Kasavajhala, M.C. Kaymak, E. King, A. Kovalenko, T. Kurtzman, T.S. Lee, S. LeGrand, P. Li, C. Lin, J. Liu, T. Luchko, R. Luo, M. Machado, V. Man, M. Manathunga, K.M. Merz, Y. Miao, O. Mikhailovskii, G. Monard, H. Nguyen, K.A. O'Hearn, A. Onufriev, F. Pan, S. Pantano, R. Qi, A. Rahnamoun, D.R. Roe, A. Roitberg, C. Sagui, S. Schott-Verdugo, A. Shajan, J.

Shen, C.L. Simmerling, N.R. Skrynnikov, J. Smith, J. Swails, R.C. Walker, J. Wang, J. Wang, H. Wei, R.M. Wolf, X. Wu, Y. Xiong, Y. Xue, D.M. York, S. Zhao, and P.A. Kollman, **2022**, Amber 2022, University of California, San Francisco.

(18). D.A. Case, I.Y. Ben-Shalom, S.R. Brozell, D.S. Cerutti, T.E. Cheatham, III, V.W.D. Cruzeiro, T.A. Darden, R.E. Duke, D. Ghoreishi, M.K. Gilson, H. Gohlke, A.W. Goetz, D. Greene, R Harris, N. Homeyer, Y. Huang, S. Izadi, A. Kovalenko, T. Kurtzman, T.S. Lee, S. LeGrand, P. Li, C. Lin, J. Liu, T. Luchko, R. Luo, D.J. Mermelstein, K.M. Merz, Y. Miao, G. Monard, C. Nguyen, H. Nguyen, I. Omelyan, A. Onufriev, F. Pan, R. Qi, D.R. Roe, A. Roitberg, C. Sagui, S. Schott-Verdugo, J. Shen, C.L. Simmerling, J. Smith, R. SalomonFerrer, J. Swails, R.C. Walker, J. Wang, H. Wei, R.M. Wolf, X. Wu, L. Xiao, D.M. York and P.A. Kollman, **2018**, AMBER 2018, University of California, San Francisco. DOI: 10.13140/RG.2.2.31525.68321.

(19) García-Simón, C.; Colomban, C.; Çetin, Y. A.; Gimeno, A.; Pujals, M.; Ubasart, E.; Fuertes-Espinosa, C.; Asad, K.; Chronakis, N.; Costas, M.; et al. Complete Dynamic Reconstruction of C_{60}, C_{70}, and $(C_{59}N)_2$ Encapsulation into an Adaptable Supramolecular Nanocapsule. *J. Am. Chem. Soc.* **2020**, *142* (37), 16051-16063. DOI: 10.1021/jacs.0c07591.

(20) Contreras-García, J.; Johnson, E. R.; Keinan, S.; Chaudret, R.; Piquemal, J.-P.; Beratan, D. N.; Yang, W. NCIPLOT: A Program for Plotting Noncovalent Interaction Regions. *J. Chem. Theory Comput.* **2011**, 7 (3), 625-632. DOI: 10.1021/ct100641a.

(21) Boto, R. A.; Peccati, F.; Laplaza, R.; Quan, C.; Carbone, A.; Piquemal, J.-P.; Maday, Y.; Contreras-García, J. NCIPLOT4: Fast, Robust, and Quantitative Analysis of Noncovalent Interactions. *J. Chem. Theory Comput.* **2020**, *16* (7), 4150-4158. DOI: 10.1021/acs.jctc.0c00063.

(22) Pinzón, J. R.; Zuo, T.; Echegoyen, L. Synthesis and Electrochemical Studies of Bingel−Hirsch Derivatives of $M_3N@I_h$-C_{80} (M=Sc, Lu). *Chem. Eur. J.* **2010**, *16* (16), 4864-4869. DOI: 10.1002/chem.200903155.

(23) Camps, X.; Hirsch, A. Efficient cyclopropanation of C_{60} starting from malonates. *J. Chem. Soc., Perkin Trans. 1* **1997**, (11), 1595-1596. DOI: 10.1039/a702055d.
